# Genome-wide microRNA screening in Nile tilapia reveals pervasive isomiRs’ transcription, sex-biased arm switching and increasing complexity of expression throughout development

**DOI:** 10.1038/s41598-018-26607-x

**Published:** 2018-05-29

**Authors:** Danillo Pinhal, Luiz A. Bovolenta, Simon Moxon, Arthur C. Oliveira, Pedro G. Nachtigall, Marcio L. Acencio, James G. Patton, Alexandre W. S. Hilsdorf, Ney Lemke, Cesar Martins

**Affiliations:** 1 Department of Genetics, Institute of Biosciences of Botucatu, Sao Paulo State University (UNESP), Botucatu, SP Brazil; 2 Department of Physics and Biophysics, Institute of Biosciences of Botucatu, Sao Paulo State University (UNESP), Botucatu, SP Brazil; 3 School of Biological Sciences, University of East Anglia (UEA), Norwich Research Park, Norwich, United Kingdom; 4 Department of Clinical and Molecular Medicine, Norwegian University of Science and Technology (NTNU), Trondheim, Norway; 5 Stevenson Center, Vanderbilt University, Nashville, TN USA; 6 Unit of Biotechnology, University of Mogi das Cruzes, Mogi das Cruzes, SP, Brazil; 7 Department of Morphology, Institute of Biosciences of Botucatu, Sao Paulo State University (UNESP), Botucatu, SP, Brazil

## Abstract

MicroRNAs (miRNAs) are key regulators of gene expression in multicellular organisms. The elucidation of miRNA function and evolution depends on the identification and characterization of miRNA repertoire of strategic organisms, as the fast-evolving cichlid fishes. Using RNA-seq and comparative genomics we carried out an in-depth report of miRNAs in Nile tilapia (*Oreochromis niloticus*), an emergent model organism to investigate evo-devo mechanisms. Five hundred known miRNAs and almost one hundred putative novel vertebrate miRNAs have been identified, many of which seem to be teleost-specific, cichlid-specific or tilapia-specific. Abundant miRNA isoforms (isomiRs) were identified with modifications in both 5p and 3p miRNA transcripts. Changes in arm usage (arm switching) of nine miRNAs were detected in early development, adult stage and even between male and female samples. We found an increasing complexity of miRNA expression during ontogenetic development, revealing a remarkable synchronism between the rate of new miRNAs recruitment and morphological changes. Overall, our results enlarge vertebrate miRNA collection and reveal a notable differential ratio of miRNA arms and isoforms influenced by sex and developmental life stage, providing a better picture of the evolutionary and spatiotemporal dynamics of miRNAs.

## Introduction

MicroRNAs (miRNAs) are small non-coding RNA molecules found in animals, plants and some eukaryotic viruses, which increase the robustness of biological processes by reinforcing transcriptional regulation. They play key roles in organismal development and in tissue homeostasis, and control a wide diversity of processes including body patterning, tissue differentiation, cell cycle regulation and carcinogenesis^1,2^.

These small non-coding RNAs have a negative regulatory role on protein-coding gene expression. Gene expression in animals is inhibited by the interaction of miRNAs with specific gene transcripts. miRNAs bind to Argonaute (AGO) proteins to form an effector complex named RNA-induced silencing complex (RISC). The complex miRNA-RISC (miRISC) interacts with specific messenger RNAs (mRNAs) and promotes regulation by several inhibitory mechanisms, inducing translational repression (through inhibition of CAP recognition and premature ribosomal drop-off), mRNA deadenylation (leading to mRNAs degradation) and mRNA decay^3–6^. miRNA-target interactions occur by perfect or imperfect complementary base pairing. Although miRNA binding sites or miRNA recognition elements (MREs) are predominantly located at the 3′ untranslated region (3′ UTR) of mRNAs^7^, recent research has revealed that binding sites may also occur at 5′ UTR and in coding sequence regions^8–11^. The miRNA regulation strength in animals is greatly dependent on the amount of complementarity between miRNA seed region (a domain at the 5′ end of miRNA transcripts that spans from nucleotide position 2 to 7) and MRE at the mRNA transcript. However, there is growing evidence that the downstream nucleotides of miRNAs may also play a relevant role^12,13^.

Primary miRNA transcripts (pri-miRNAs) are transcribed during biogenesis in the nucleus and processed by a suite of proteins, including endonuclease Drosha^14^, an RNAse III-like enzyme. miRNA cleavage by Drosha initiates by recognizing hairpin structures within pri-miRNAs and it is followed by the release of ~70 nucleotide precursor miRNAs (pre-miRNA). Pre-miRNAs are then transported to the cytoplasm where Dicer, another RNAse III-like enzyme, cleaves the pre-miRNA near the terminal loop, ~22 nucleotides from the Drosha cutting site at the 5′ end^15^. The released miRNA duplex is then loaded onto one AGO protein that retains one strand while degrading the other. For some duplexes, each strand is alternatively retained into one RISC. Depending on the duplex, the two strands are retained with similar or different frequencies^15^.

Strand selection depends on the differential thermodynamic stability of the 5′ and 3′ ends of the miRNA duplex and nucleotide composition by not yet fully understood mechanisms. Drosha alternative processing may produce distinct pre-miRNAs, generating duplexes with variable stability at the 5′ position^16^. The miRNA strand harbouring the most unstable 5′ end portion will be the one associated with the AGO proteins^17,18^. Moreover, the evolutionary drift of duplicated miRNA genes is also believed to interfere in the relative abundance of strands, thereby influencing their selective incorporation into RISCs. This process of alternative strand selection or change in arm usage is named “Arm Switching” (ARS) and has been detected in studies comparing miRNA expression profiles in different tissues^19^. In fact, ARS has been proposed to be a common and imperative phenomenon of miRNA diversification^2^.

Another miRNA feature refers to the widespread generation of isoforms called ‘isomiRs’. Such variant miRNA molecules embrace single nucleotide substitutions, indels, 3′ end non-templated additions as well as 5′ and/or 3′ cleavage shifts. Their origin comes from alternative Dicer and Drosha cleavages resulting in size variants of canonical miRNAs. Furthermore isomiRs have been shown to be non-randomly distributed and functionally active as partners of canonical miRNAs in several tissues of humans^20,21^. Indeed 5′ and 3′ end isomiRs may control different targets and may be expressed in a developmental and tissue specific manner, implying coordination between miRNA biogenesis and function^22^.

Many miRNAs are highly conserved throughout metazoans in terms of structure and function^23^, whereas non-conserved or evolutionarily divergent miRNAs have been linked to the establishment and maintenance of specific cellular or organismal phenotypes^24,25^. Several miRNAs exhibit noticeable spatiotemporal and tissue-specific expression, or even cell-type specific expression signatures in distinct tissue layers within a single organ^26,27^.

The majority of current advances in miRNA discovery came from the widespread use of next generation sequencing (henceforth named RNA-seq) which successfully uncovered a substantial number of miRNAs in various species^28–30^. The RNA-seq approach is particularly suitable for accessing ancient conserved miRNAs and species-specific non-conserved miRNAs or miRNAs groups, commonly expressed at lower levels than conserved miRNAs^2^.

Presently, about 49,000 mature miRNA sequences derived from ~38,500 hairpins sequences from more than two hundred species (miRBase 22, March 2018) have been identified^31^. Although many entries for several Actinopterygii or ray-finned fishes from distinct groups have been reported, miRBase still lacks miRNA profiling from the majority of roughly 30,000 fish species, the largest group of living vertebrates.

Cichlids are evolutionarily, economically and environmentally important fish species used in aquaculture, aquarium trade, wild fisheries and science. The Nile tilapia (*Oreochromis niloticus*), an African native fish of the biodiverse Cichlid family, has been globally acknowledged as an economically valuable species owing to its ease in breeding and its high growth rate within a variety of aquaculture systems^32^. Frequently referred to as the “aquatic chicken”, the tilapia’s high endurance, easy adaptability, rapid growth rate and good consumer acceptance are some of its remarkable features^33^. The Nile tilapia is also highlighted as a scientific model in the comparative evolutionary biology of vertebrates, with significant prominence due to the recent completion of its full genome sequencing at high coverage level^34,35^.

Genomic resources for the Nile tilapia are currently at an advanced stage. However, state-of-the-art knowledge on the species miRNA compendium and its miRNA diversity, genomic context and expression patterns remains largely incomplete, in spite of the few studies available^36–43^.

In this paper we present an in-depth analysis of miRNA profiles in several tissues of both sexes and at several distinct developmental stages of Nile tilapia. We largely expand upon tilapia’s reported miRNA repertoire by identifying 368 miRNA loci expressing 567 mature miRNAs. Remarkably, we detected 178 novel tilapia miRNAs of which 90 have never been previously reported for vertebrates. Sexual dimorphism, isoform signatures and arm switching events were also characterized. Furthermore, we describe the genomic context of miRNA genes with a detailed description of their physical location and clustering patterns in the Nile tilapia’s putative chromosomes.

## Results and Discussion

### Overview of tilapia small RNA sequencing

**Figure 1:**
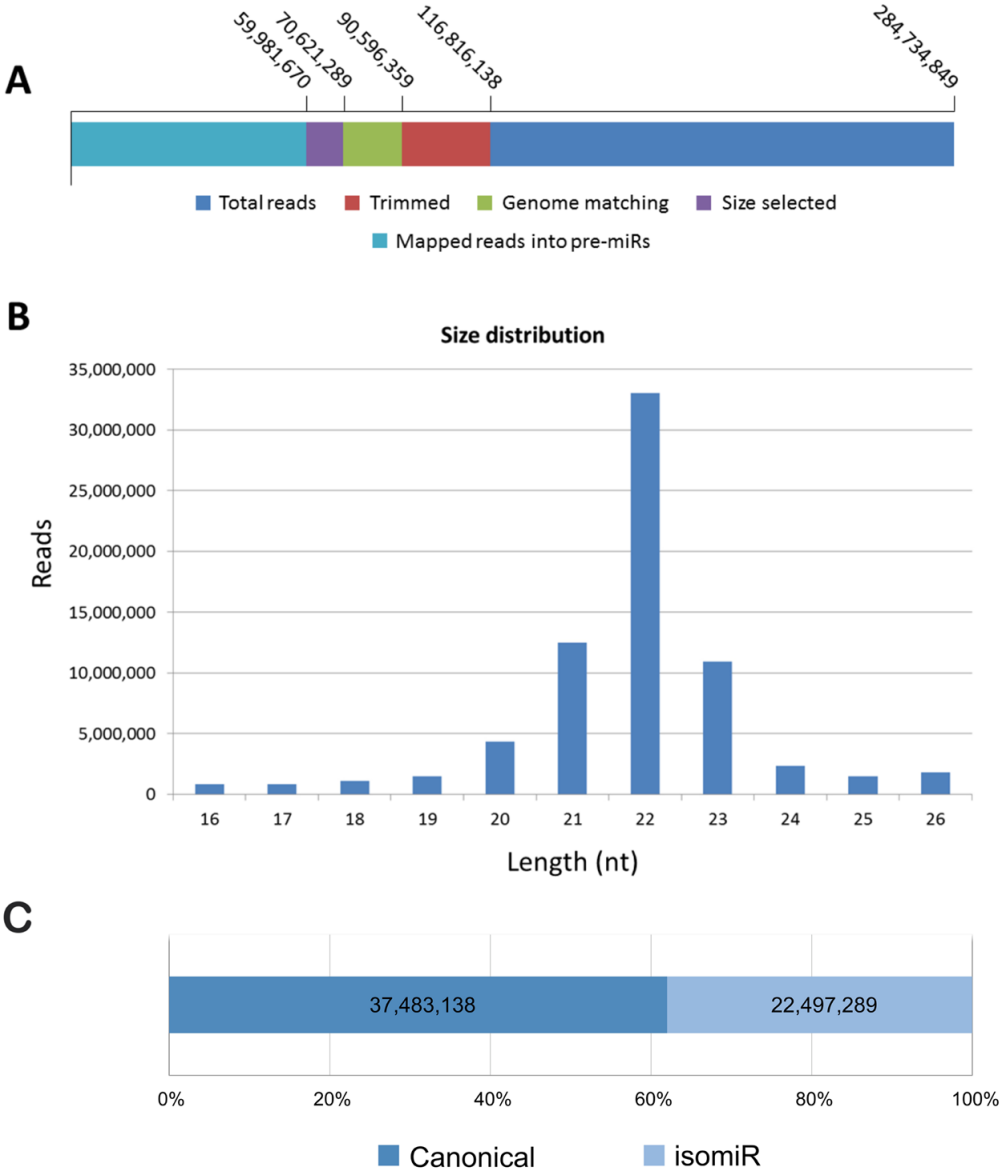
RNA-seq overview. (**A**) Reads obtained from RNA-seq. (**B**) Size distribution of “size selected” reads used for the analysis. (**C**) Read count of canonical and isomiR reads.

So that miRNA composition and expression could be investigated, samples at distinct developmental stages – embryos at 2 days post fertilization (dpf), 3 dpf, 4 dpf and 5 dpf; larvae at 10 dpf; eyes, liver (mixed sexes), heart (female), gonads, brain, red muscle and white muscle of male and female adults – were used to construct 16 individual small RNA libraries (15–40 nt) for Illumina Sequencing (HiSeq 2000). Approximately 285 million of reads were generated, subjected to quality filtering and removal of 5′ and 3′ adaptors. Other classes of small RNAs were excluded out of the ~90 million reads matching the Nile tilapia genome, whereas the remaining 60 million reads that mapped to hairpins were extracted and used for subsequent bioinformatics analysis. Figure 1A and Supplementary Table S1, provide an overview of read counts.

Reads were grouped and aligned by methodology (described in the Materials and Methods section) that combined read counts from all samples. Canonical miRNAs comprised the most abundant sequences of each alignment, whereas all non-canonical sequences were isomiRs. If a sequence was present in miRBase, it was considered a known miRNA; otherwise, it was described as a novel miRNA.

Characterization of reads revealed that roughly 80% were 21–23 nt long (Fig. 1B). Prevalent miRNA length was 22 nt for most tissues, except in liver and in 10-day post-fertilization (dpf) embryos samples, frequently 23 nt long. Muscle and brain samples generated the largest number of reads containing putative miRNAs. Gonad sample sequences length distribution showed an additional peak at 26 nt corresponding to putative piwi-RNAs (piRNAs) (see Supplementary Table S2, for more details). Evidence exists that this class of small RNAs protects germ cells by silencing transposons and retrotransposons^44^ and thus contributes towards the maintenance of genome integrity and stability^45^.

Figure 1C presents a quantitative statistics of reads from known miRNAs, novel miRNAs and isomiRs. Reads corresponding to known miRNAs were more abundant in embryos and adults when compared to those of novel miRNAs (a qualitative description of the miRNA repertoire identified is provided below). It should be underscored that isomiRs reads in certain samples were more abundant than reads of corresponding canonical miRNAs (see Supplementary Table S3).

### Extensive identification of miRNA loci

Accurate recognition of miRNAs relies on the availability of genomic resources since mapping and mining precursor sequences are a fundamental step in computational identification. Owing to the availability of the Nile tilapia genome, a search for miRNAs was systematically executed. Overall, our analysis proved to be highly sensitive, allowing for the identification of a large number of known conserved miRNAs and several novel miRNAs never before described in any vertebrate species in miRBase^31^.

In the case of the Nile tilapia, 271 pre-miRNAs, corresponding to 540 known mature miRNAs (270 miRNA-5p and 270 miRNA-3p), were detected, or rather, a similar diversity when compared to previous reports in other fishes^46–48^. When the stringency criterion of minimum read count was applied (an expression level larger than 5 raw reads in at least 25% of the samples)^49^, the number of mature miRNAs retained for analysis was reduced to 470 (243 miRNA-5p and 227 miRNA-3p) (Supplementary Tables S4, and S5).

Furthermore, 97 novel pre-miRNAs loci, producing 194 putative novel mature miRNAs (97 miRNA-5p and 97 miRNA-3p), were discovered. When the same read count stringency criterion was applied, as above, 97 novel miRNAs (60 miRNA-5p and 37 miRNA-3p) were retained (Supplementary Tables S4 and S5) In addition to read abundance, novel miRNAs tended towards a stable secondary hairpin structures, consistent with requirements for the biogenesis of mature and functional miRNAs. All hairpin sequences showed very low Minimum Free Energy rates (MFE ≤ -15), which clearly suggested bona fide miRNAs (Supplementary Fig. S1).

**Figure 2:**
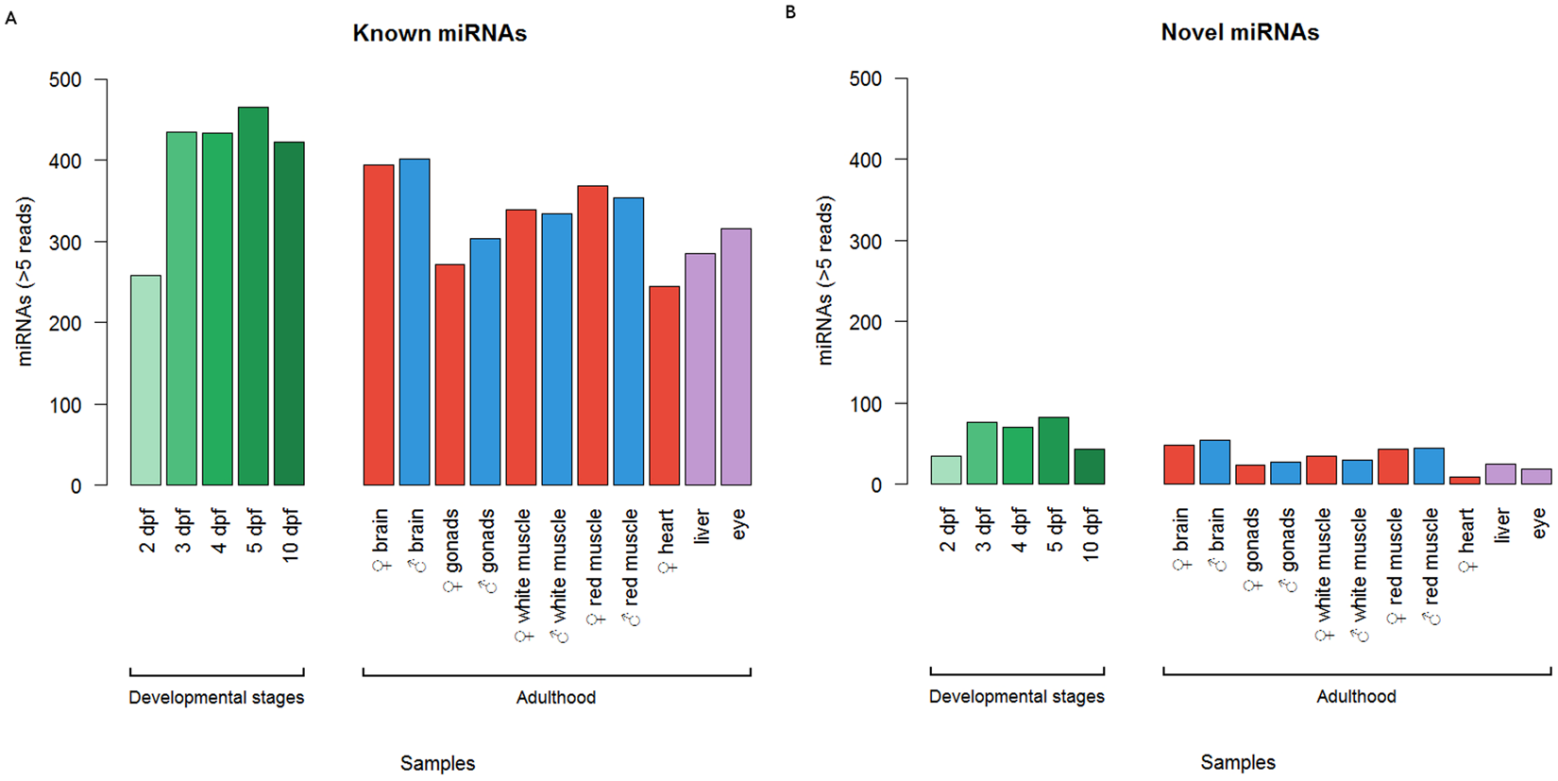
Patterns of miRNAs distribution. Known (**A**) and Novel (**B**) miRNAs detected (>5 reads) among developmental stages and adult tissues samples. *dpf = days post-fertilization; developmental stages, liver and eye correspond to pool samples.

Known mature miRNAs presented a heterogeneous qualitative distribution in adult tissues (Fig. 2). For instance, known miRNAs were almost one quarter more diverse in male and female brain samples than in gonad samples (Fig. 2A). In embryos, the number of known miRNAs incresed along developmental stages, with a ~1-fold up in global expressed miRNAs, from 2 to 10 dpf. Similarly, the number of novel miRNAs expressed in embryos was concentrated (74.5%) at 3 to 5 dpf, whereas novel miRNAs in adults were particularly abundant in the brain (28.4%) and red muscle (24.5%) of both sexes. The liver sample showed a relative reduced diversity of novel miRNAs in comparison to other tissues, however liver miRNAs stand for 40.6% of the total number of novel miRNA reads expressed in adult tissues, suggesting these novel miRNAs are putative enriched or tissue-specific miRNAs (Fig. 2B; Supplementary Tables S1 and S6).

miRNA sequences from the two ends of the precursor (i.e., 5p or 3p) were identified and assigned to miRNA families based on seed sequence identity. Through a detailed analysis, known miRNAs could be arranged into 249 families. Contrastingly, novel miRNA sequences presented 84 variable seeds within the precursor sequences, suggesting that some novel miRNAs belonged to new families and that some novel precursors were paralogous copies or were expressed at low levels (i.e., <5 raw reads). Interestingly, three novel tilapia miRNAs identified in current study were assigned to known miRNA families due to the fact that their seed sequence perfectly matched known miRNAs families (Supplementary Table S7), even though their precursor transcripts had enough nucleotide differences to be categorized as novel miRNAs.

The diversity of Nile tilapia miRNAs was compared to available data from other cichlidae species reported by Brawand *et al*.^39^: *Oreochromis niloticus*, *Neolamprologus brichardi/pulcher*, *Metriaclima zebra*, *Pundamilia nyererei* and *Astatotilapia burtoni*; by Loh, Yi and Streelman^50^: *Labeotropheus fuelleborni*, *Melanochromis auratus*, *Maylandia zebra*, *Tyrannochromis maculiceps*, *Docimodus evelynae*, *Nimbochromis polystigma*, *Mchenga conophoros* and *Rhamphochromis esox*; by Huang *et al*.^36^, Yan *et al*.^40^ and Tao *et al*.^51^: *Oreochromis niloticus*; by Franchini *et al*.^52^: *Amphilophus citrinellus*, *A*. *astorquii*, *A*. *zaliosus*, *A*. *amarillo*, *A*. *sagittae*). For instance, Brawand *et al*.^39^ identified 259–286 miRNA loci per cichlidae species (*O*. *niloticus* included), whereas our study detected 271 known miRNAs loci. The sensitivity of our analysis made possible the discovery of additional miRNAs. Further, 368 miRNAs loci were identified by taking into account putative novel miRNAs, a significant increase over Brawand *et al*.^39^ who discovered only 40 novel putative novel miRNAs. Such difference may be due to a richer set of tissues and developmental stages considered in this study. Current novel miRNAs could not be checked to detect whether they were also present in Brawand’s results since the latter were not present in miRBase v.21.

Results here reported contribute towards a better annotation of miRNAs in cichlidae. Since miRNAs are highly conserved in cichlidae species, the number of bona-fide miRNAs from *O*. *niloticus* could be increased. Loh *et al*.^50^ detected 114 miRNA loci, corresponding to 100 miRNA genes, distributed in the 5 cichlidae species: *Labeotropheus fuelleborni* (26 miRNAs), *Rhamphochromis esox* (18 miRNAs), *Maylandia zebra* (32 miRNAs), *Melanochromis auratus* (10 miRNAs) and *Mchenga conophorus* (28 miRNAs). Further, Franchini *et al*.^52^ detected 236 known miRNA genes within six *Amphilophus species* (cichlids from Central America). Combining publicly available data to current new comprehensive approach, the number of annotated miRNAs could be increased by 178 in the Nile tilapia and by 106 in cichlids.

### Genomic organization of miRNA loci

**Figure 3:**
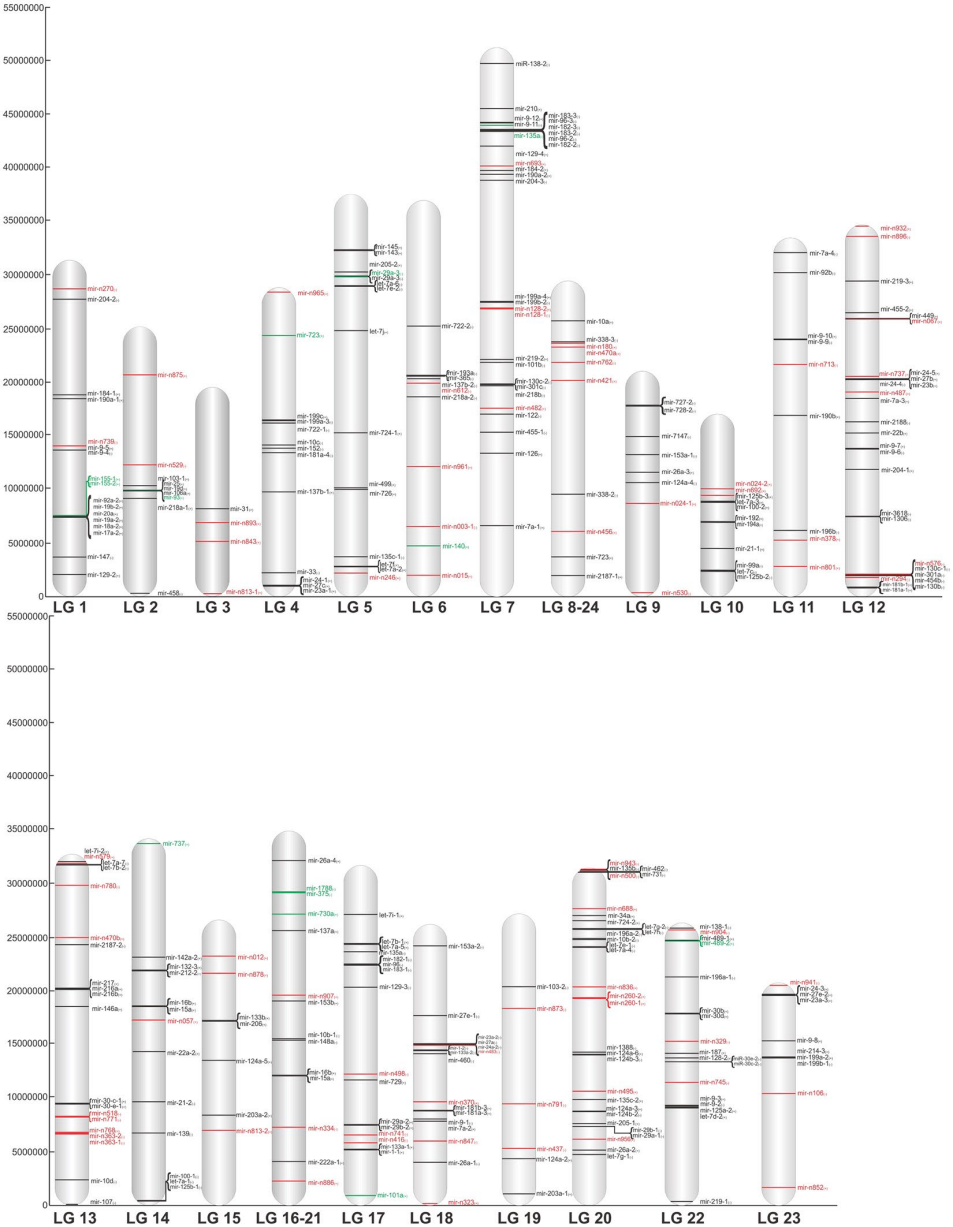
Genome organization of miRNAs genes in Nile tilapia genome. Black: known miRNAs. Red: novel miRNAs described in current study. Green: novel miRNAs described previously by Huang *et al*.^36^ and Yan *et al*.^40^; MiRNAs in brackets: clustered miRNAs.

Sequences of miRNA precursors were mapped on the genome of Nile tilapia, currently organized in linkage groups (LGs), corresponding to 22 putative chromosomes. Figure 3 shows the physical location and strand orientation of precursors of known and novel miRNAs in LGs. It should be underscored that 65 precursors were mapped to scaffolds not yet anchored to any of the LGs, because the tilapia’s genome is not fully assembled owing to its recent completion. Nevertheless, 90 mature miRNAs originating from the 65 precursors were mapped (Supplementary Table S4).

Some LG-assigned miRNAs could be mapped on a single locus, although most had paralogs at several genome locations. For instance, let-7, the most abundant miRNA, was represented by 21 paralogs distributed across seven LGs, whereas 51 miRNAs were mapped on a single locus. The occurrence of miRNA paralogs at multiple genomic locations might be directly linked to a third round of whole genome duplication, known as the teleost-specific genome duplication (TGD)^53^. Accordingly, several paralogs have been consistently reported in the rainbow trout genome that retains most miRNA genes in duplicated genomic copies^54^.

In tilapia LGs, miRNA genes are organized either sparsely or grouped in 46 clusters (Fig. 3), many of which encode novel miRNAs. At least one novel miRNA gene was mapped on each LG. Eight novel miRNAs were discovered on LG20. Interestingly, a pair of novel clustered miRNAs (miR-n518 and miR-n771) separated by only ~200 bp were identified in LG13 (Supplementary Table S4). Although situated in the larger chromosome of *O*. *niloticus*^55^, LG3 had the smallest number of miRNAs (only four), corroborating data by Tao *et al*.^51^. In contrast, LG10, with approximately the same size of LG3, showed 11 miRNAs. The above may be due to the lack of full anchoring of the LG3 chromosome. In fact, some of the identified but not yet mapped miRNAs could be located at this chromosome. Another potentially relevant fact is that *in situ* hybridization chromosome mapping has evidenced a huge amount of heterochromatin and repeated DNAs anchored on the LG3 chromosome^56^. Consequently, predominance of heterochromatin over euchromatin on this region may also account for the small number of LG3 miRNAs.

Regarding the genomic characterization, miRNA genomic context was classified as intergenic, intronic or exonic and as encoded or not encoded as part of a gene cluster. Our analysis determined that 179 (48.6%) precursors could be classified as intergenic, whereas 142 (38.6%) were intronic and 47 (12.8%) exonic in tilapia. MiRNA host genes were distributed across 15 distinct LGs and 7 within unanchored genome regions not yet fully annotated. Interestingly, 13 novel exonic miRNAs were identified (Supplementary Table S4).

Differences in the organization of miRNA genes were detected between Nile tilapia and zebrafish genomes. For instance, in the zebrafish most miRNAs (~86%) were found in intergenic regions but only 12% within introns^46^, whereas there was an equivalent percentage of intergenic (48.6%) and intragenic (51.4%) miRNAs in the tilapia. A comparison between vertebrate species and the tilapia revealed that the cichlid fish had a similar rate of intergenic miRNAs as mammals (humans, 52.1% Hinske *et al*.^57^ and 49.9% Paczynska *et al*.^58^; mouse, 58.4% Hinske *et al*.^57^ and 44.8% Paczynska *et al*.^58^; pig, 66.9% Paczynska *et al*.^58^ and bovine 59.5% Romao *et al*.^59^) and crustaceans (49% Xu *et al*.^60^). However, the trout^48^, the dog, and probably the chicken^57^ have a higher composition of intergenic miRNAs than *O*. *niloticus*. In the case of the intronic miRNAs fraction, tilapia resembles mammals since both have fewer fractions than *Xenopus tropicalis*, with 71.1%^61^. When compared to other vertebrates, there is a higher number of exonic miRNAs in *O*. *niloticus* (12.8%), which, in turn, is similar to amphibians (13.4%)^61^ (Supplementary Table S8). Results may indicate a exclusive attribute of these species or it may be due to poor gene annotation.

We also found intronic novel miRNAs (46%) predominated over intronic known miRNAs (36%) in the tilapia, corroborating previous data for canine^62^ and other mammalian species^63^. Results are consistent with the observation that the birth of miRNAs genes predominantly occurs in introns^63^, following a parsimonious evolutionary route^2^.

### Identification and quantification of isomiRs

**Figure 4:**
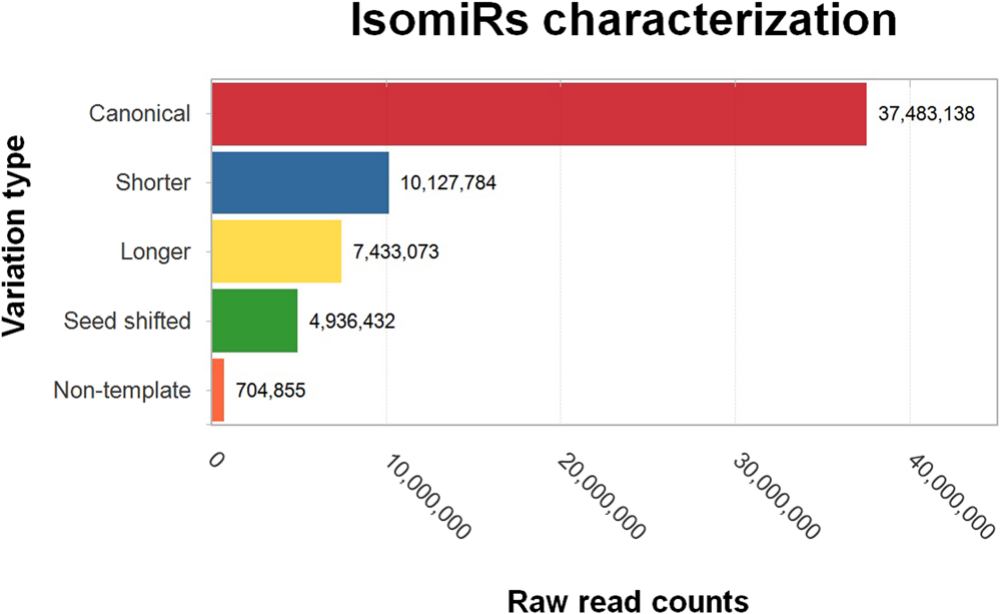
Categorization and quantification of isomiRs sequences. Isoforms were categorized into canonical sequences; Shorter – isomiR sequences with reduced length when compared to canonical sequence; Longer – isomiR sequences with increasing nucleotide base; Seed shifted – isomiRs with any modification at 5′ end (nucleotide gain or loss) that changed seed region (2–7 nt region); and Non-template - IsomiRs with any nucleotide mismatch with the precursor sequence. Henceforth canonical sequences were defined as the most miRNA 5p and 3p expressed sequences of combined samples.

Several miRNA isoforms were detected in tilapia embryos and adult tissues (Fig. 4; Supplementary Table S9 and Fig. S2). Their expression levels were extensive in several samples. In fact, individual isomiRs were more frequent in some samples than their respective canonical forms (Supplementary Table S3). These findings suggest that isomiRs have a biological function and may play relevant roles in sharpening gene regulation in tilapia, as previously reported for other species^64,65^.

Most isomiRs derived from single nucleotide modifications at the 3′ end, or rather, 12.57% were generated by a single nucleotide deletion (3p − 1) and 10.34% by a single nucleotide insertion (3p + 1). Double deletions at the 3′ end (3p − 2) accounted for 2.82% of isoforms. IsomiRs containing changes at the 5′ end (5p − 1) were much less common (2.1%), which was expected since changes in 5′ end alter the seed region (Supplementary Fig. S3) and therefore, modifiy miRNA-mRNA pair interactions. The distribution of Nile tilapia isomiRs was similar to previous data on rat heart samples^65^ and current results demonstrate that the expression of a rich collection of isomiRs seems to be a feature common to all tissues.

Several biological contexts from multiple *O*. *niloticus* individuals were analysed and the consistent presence of isomiRs indicate that they cooperate with canonical miRNAs to control biological processes^20^. The abundance of highly expressed isomiRs foregrounds the idea that they are not randomly generated and they do have important biological functions^65,66^. *In vitro* experiments have shown that isomiRs could be more efficient on target recognition and repression than their respective canonical form^20^. Moreover, 5′ end isomiRs may be important for the regulation of gene expression because shifts in seed sequence could lead to differential recognition of targets, thus making possible new miRNA-mRNA interactions in the cells, which could result in new regulatory pathways^21^. Divergence at the 3′ end should not be so critical because only occasionally such changes modify the target repertoire of a given miRNA. Consequently, the discrepancy between expression levels of 3′ end and 5′ end isomiRs may be due to selective constraints on the efficiency of the Drosha and Dicer cleavage which seems to be more disruptive at the 5′ rather than at the 3′ end. This could maintain cellular homeostasis and prevent abnormalities in primary pathways regulated by miRNAs.

### Identification of miRNA arm switching

Arm switching (ARS) refers to a shift in the ratio of mature 5p and 3p strands that get loaded into the miRNA-induced silencing complex (miRISC)^67^. Variable incorporation of the strands provides raw material for the evolution of new targets, extending the repertoire of post-transcriptional gene regulation. Previous studies have shown that miRNAs are able to switch the dominant arm loaded in miRISCs and that arm usage varies between tissues and developmental stages. ARS may result in distinct miRNAs regulatory effects over an enlarged set of cellular targets. Switches in the incorporation of the two arms are a well-documented phenomenon in diverse miRNAs families of fruit flies and worms^68,69^ and ARS has also been recently identified in teleostei^39^. Such ARS events are likely to be explained by distinctive miRNA processing by Drosha, which modifies the relative thermodynamic stability of the miRNA duplex ends (reviewed by Ha and Kim^15^). In current study, arm switching in embryos and in adult tissues for eight known and one novel miRNAs were detected (Supplementary Table S10).

**Figure 5:**
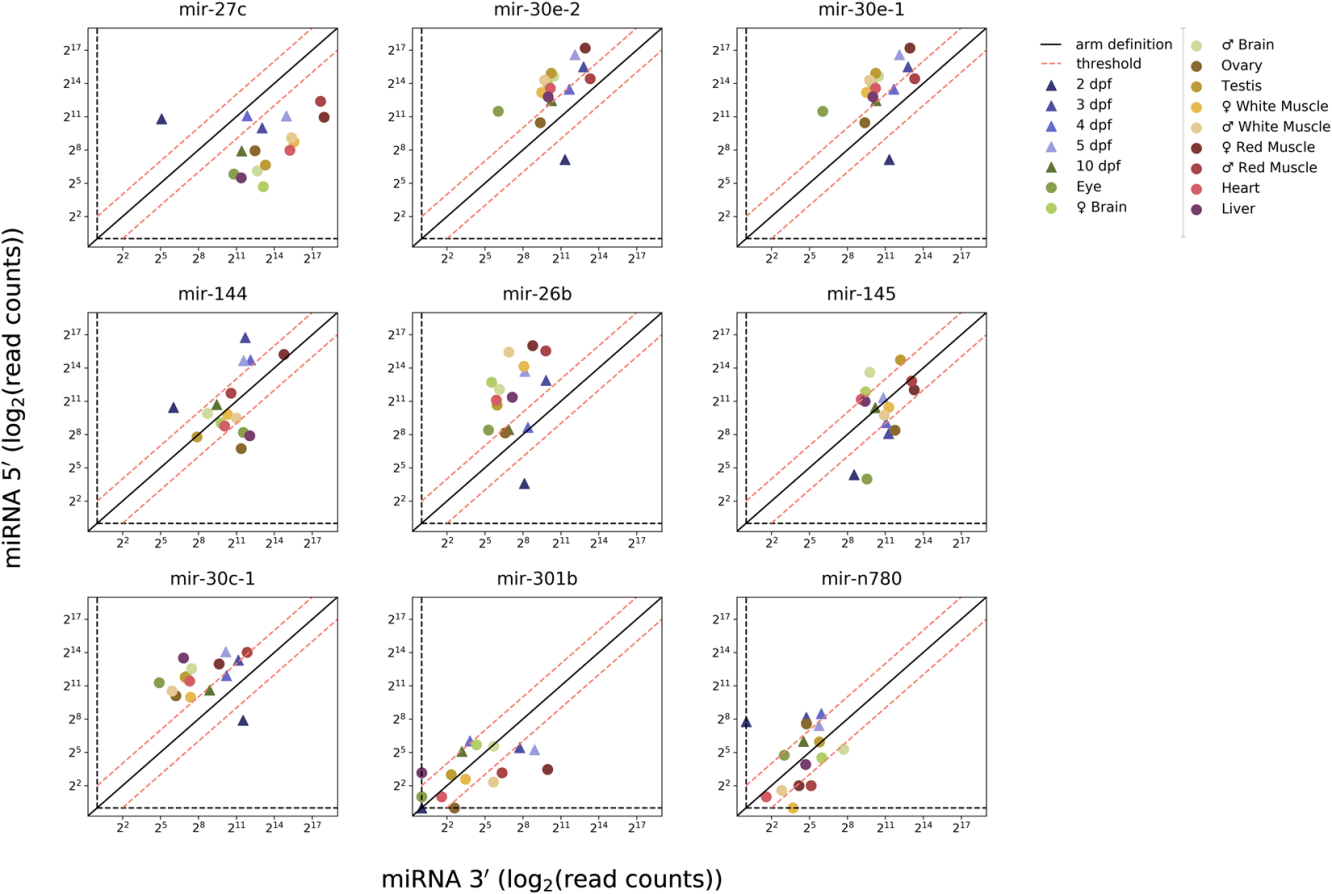
Individual profiles of miRNAs showing switches in arm counts at early and adult stages. X-axis represents 3p miRNA mapped raw read counts (log_2_) and Y-axis represents 5p miRNA mapped raw read counts (log_2_). Diagonal line represents same expression rates from 5p and 3p. Data series on the right side of the line shows greater proportion of 5p over 3p, while data series on the left side of the line shows a greater proportion of 3p over 5p.Threshould (dashed red line) represents Fold change ≥2 or ≤−2.

Figure 5 shows the scatter plot for the nine miRNAs with the highest expression variation between 5p and 3p arms. The 5p and the 3p expressions are respectively shown on the y and x axes. The line represents a case of equal expression for 5p and 3p. Elements located far from this line represent extreme cases in which the expression is concentrated on a single arm. ARS occurs for miRNAs in which a bias change for 5p or 3p expression develops.

We analysed miRNAs revealing either (i) a tissue specific difference with regard to relative abundance between 5p and 3p arms (fold change ≤−2 or ≥2) or (ii) developmental changes during embryonic stages compared to adult tissues. Using Fisher’s exact test, statistically relevant variable arm expression for several miRNAs was detected among tissues and developmental stages (Supplementary Table S10 provides a detailed comparison of ARS for different miRNAs). As examples, we highlight miR-144 and miR-145 (Fig. 5).

**Figure 6:**
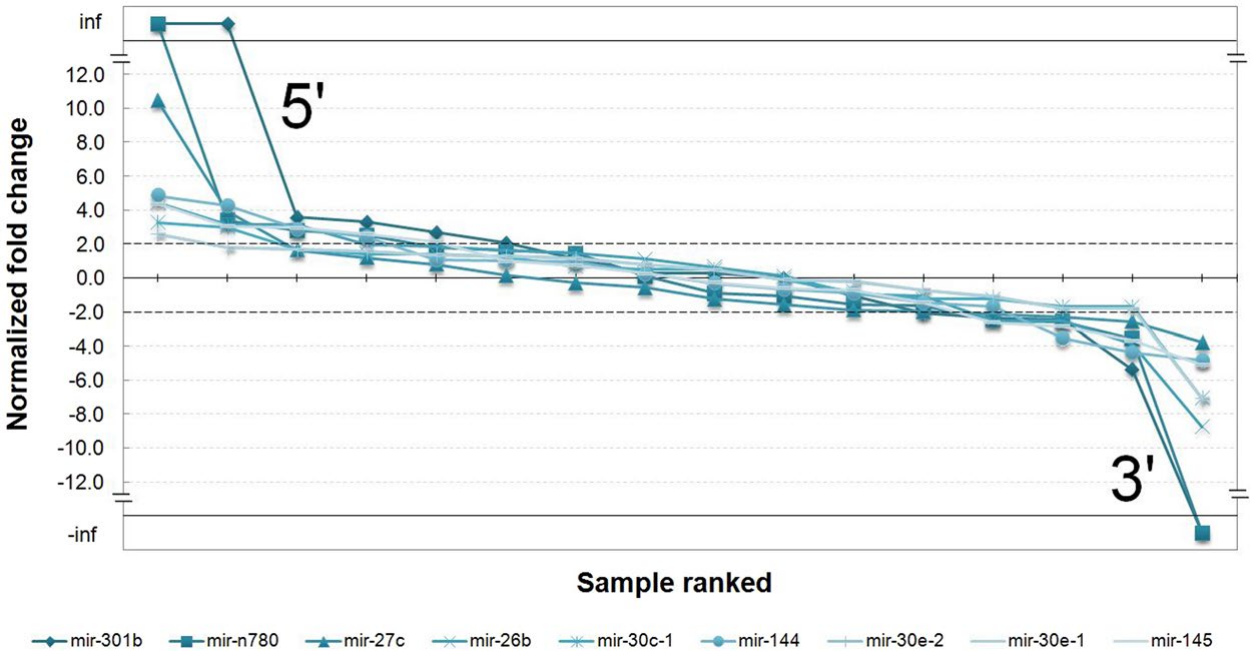
miRNA arm switching events detected and ranked in Nile tilapia. The miRNA 5p and 3p expression prevalence in each sample is represented by normalized fold change rates. Rates were normalized by the difference between the miRNA fold change from each sample and the mean of miRNA fold change of samples to collapse the series curves aiming at the improvement of data visualization. The 5p and 3p variations were ranked among samples organized from the greatest (prevalent 5p) to the smallest (prevalent 3p) fold rates per each pre-miRNA. The pre-miRNAs were also ranked from the greatest to the smallest distance between max and min fold change among samples (from dark to light blue series).

Figure 6 represents the normalized and ranked fold change for nine ARS detected, and integrates on a single plot the data on Fig. 5. In this representation, a flat curve represents a uniform case with no switching event. The curve’s declivity represents the strength of the ARS event; strong events are shown in bold blue. Current data reveal that the strongest ARS occurred for mir-27-c. In the case of mir-27-c, the fold-change varied between 5.7 for the sample 2 dpf and -8.4 for the sample female brain. The curve also demonstrates that in certain cases a strong variation exists on a given tissue, whereas in other cases the variation is distributed among different samples.

The 3p arm in miR-144 was dominant in adult tissues, whereas the 5p arm was more highly expressed in developmental stages. In adult tissues, miR-144 is linked to the control of the chloride channel in the human lung and to a key transcription factor (nuclear factor-erythroid 2-related factor 2 - NRF2) responsible for modulating the response to oxidative stress in primary erythroid progenitor cells in mammals^70^. In zebrafish, a similar association with erythroid cells has been detected for miR-144^71^. In fact, miR-144 dysfunction has been associated to such diseases as Cystic Fibrosis^72^, Alzheimer’s Disease^73^ and premature ovarian failure in rats^74^. Consequently, miR-144 function during developmental stages is associated with hematopoiesis and vascular development regulation through the repression of meis1 expression^75^. When 5p and 3p arms are considered separately, miR-144–5p has been reported as down-regulated in the ovary in response to hypoxia in marine medaka (*Oryzias melastigma*)^76^, and miR-144–3p was registered as essential for the regulation of cholesterol homeostasis and inflammatory reactions causing atherosclerosis in humans^74^.

Another noticeable example of ARS was miR-145. We detected that miR-145 arm switching is sex-dependent, with miR-145–3p expressed at 3.4-fold higher levels in ovaries and miR-145–5p expressed at 2.5-fold higher levels in testis. Similarly, miR-145–5p in the Atlantic halibut (*Hippoglossus hippoglossus*) proved to be sex-dependent, with higher levels in male testis, where it controls the differentiation and renewal rate of germ stem cell^77^.

### Increasing complexity of miRNA expression during development and differentiation

**Figure 7:**
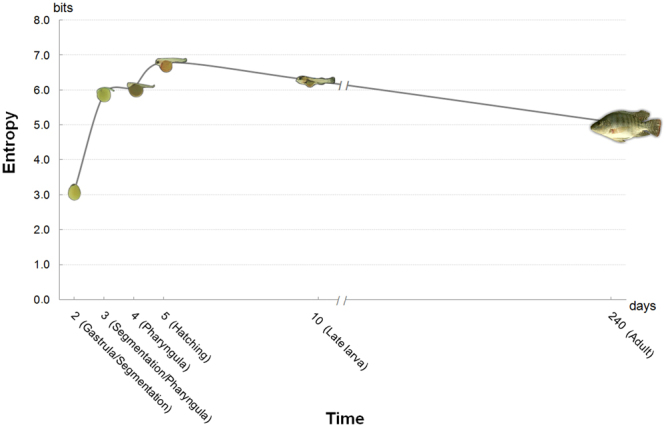
Entropy analysis of miRNAs. Shannon’s entropy was used to quantify miRNA diversity for developmental stages through adulthood. We observed an increase of diversity at early developmental stages (2 to 5 dpf), where cell differentiation and organ formation occurs, followed by stabilization in late larval (10 dpf) and adult (6 month years old) stages. Developmental stage labels and images were obtained from Fujimura and Okada, 2007 under Creative Commons Attribution 4.0 International (CC BY 4.0 - authors “Koji Fujimura and Norihiro Okada”, title “Fig. 1. Overview of the developmental stages of Nile tilapia *O*. *niloticus*.”, date “14 March 2007”, url “http://onlinelibrary.wiley.com/doi/10.1111/j.1440-169X.2007.00926.X/full”, no endorsement, only esthetical adaptations: image vectorized, background removed, shadow included and image rotated. Adult *Oreochromis niloticus* image from https://www.usgs.gov/ under the Public domain.

Compared to later development and adult stages, a much more limited number of miRNAs is expressed during the early development stage. A significant difference (3 standard deviations; Supplementary Fig. S4) in expression patterns between embryos at 2, 3 and 5 days post-fertilization (dpf) has been reported when compared to adult patterns (Fig. 7). In other words, at an early stage (i.e. 2 dpf), qualitative (diversity) and quantitative (expression) levels of miRNAs were low, whereas increasing complexity occurred in later stages (i.e., augmented entropy). It seems that miRNAs only refines gene expression patterns although current data suggest that increasing complexity of expression may trigger development and differentiation of specific cells and tissues.

Heimberg *et al*.^29^ proposed a relationship between acquisition rate of new miRNAs and morphological changes throughout the evolutionary history of vertebrate species, chiefly during the appearance and at the peak of the diversification rate of vertebrate classes. The emergence of vertebrates was followed by two major genome duplications that might be associated with increasing complexity of miRNA expression and the emergence of new species.

In tilapia, the increasing miRNA complexity during the transition from the embryonic to the adult stage (Fig. 7) may be paralleled to the correlation between the peak of miRNA acquisition and increased morphological complexity observed at the dawn of vertebrates described by Heimberg *et al*.^29^. Results suggest that high miRNA entropy observed at the early developmental stages might be related to the increased diversity of protein-responsive miRNA circuits which trigger cells to differentiate into distinct cell types, tissues and organs. By contrast, the average entropy level was slightly smaller in the adult body, where differentiated cells predominate, indicating that a simpler regulatory program is required to maintain homeostasis.

When exclusively analysing the expression of novel miRNAs, particularly those that are species-specific, it may be observed that the expression of the majority was concentrated at the 3 dpf and 5 dpf developmental stages, featuring high entropy levels. Since a high percentage of cells differentiate and actively produce great quantities of transcripts at these stages, we envision that this is the perfect scenario for a newly emerged miRNA to acquire functions in a specific pathway. Moreover, several novel miRNAs expressed at early developmental stages may be involved in key regulatory mechanisms during subsequent stages, because the individual miRNAs and miRNA families remain active after their earliest expression, thus maintaining their functionality in the control of gene expression in succeeding cell stages^78^.

### Tracing miRNAs evolution

**Figure 8:**
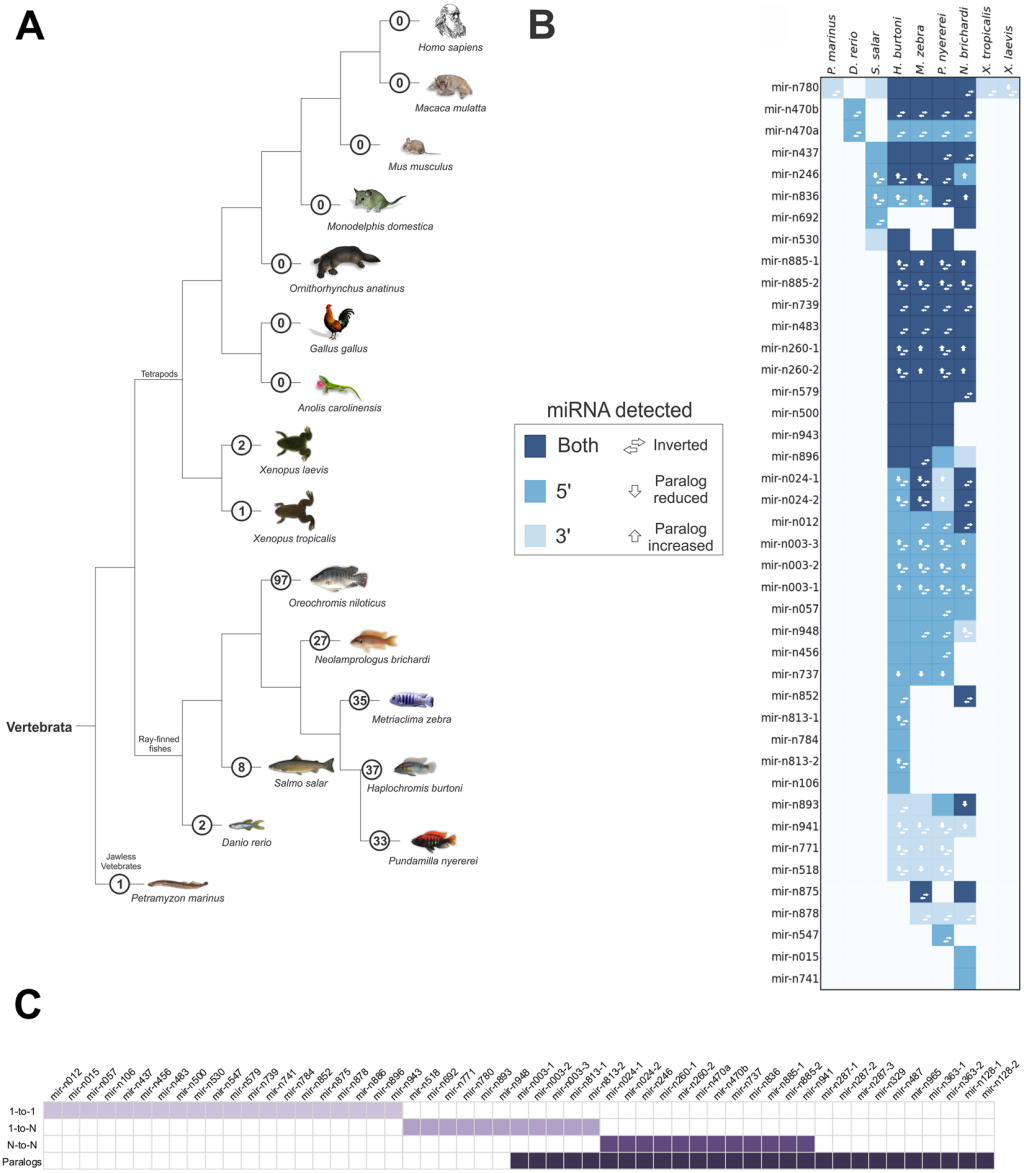
Detection of novel Nile tilapia miRNAs in diverse vertebrate species by miRDeep2 approach and ortholog/paralog annotation. (**A**) Vertebrate evolutionary tree with the number of novel miRNAs (inside circles) detected in each species through genome-wide matching searches by miRDeep2 software. Phylogenetic tree is a handmade tree derived by merging published trees by Brawand *et al*.^39^ and Amemiya *et al*.^94^. *Homo sapiens*, *Ornithorhynchus anatinus*, *Petromyzon marinus*, *Danio rerio*, *Salmo salar*, *Gallus gallus* and *Monodelphis domestica* images were obtained from https://commons.wikimedia.org/wiki/Main_Page under the Public Domain; *Macaca mulata* and *Mus musculus* images from https://pixabay.com under the Public Domain; *Pundamilia nyererei*, *Haplochromis burtoni* and *Neolamprologus brichardi* adapted by permission from Macmillan Publishers Ltd: Nature (Brawand *et al*.^39^), copyright 2014, https://www.nature.com/; *Xenopus tropicalis* and *Xenopus laevis* images from http://journals.plos.org/plosbiology/ under Creative Commons Attribution 4.0 International (CC BY 4.0 - authors “Patrick Narbonne, David E. Simpson and John B. Gurdon”, title “Figure S1. Characterization of the hybrids formed by the cross-fertilization of *X*. *laevis* eggs with *X*. *tropicalis* sperm.”, date “15 November, 2011”, url “https://doi.org/10.1371/journal.pbio.1001197.s001”, no endorsement, only esthetical adaptations: image vectorized, background removed, shadow included and image rotated); *Metriaclima zebra or Maylandia zebra* and *Anolis carolinensis* images from http://www.publicdomainpictures.net under CC0 1.0 Universal: CC0 1.0 Public Domain Dedication; *Oreochromis niloticus* image from https://www.usgs.gov/ under the Public domain. (**B**) Comparative features of 42 novel miRNAs among species, including sense orientation, paralogs abundance, and arms detection. Other 55 novel miRNAs were tilapia-specific, and therefore, excluded from this comparative analysis. (**C**) Orthologs and paralogs relationship cardinalities considering common anscestral and the “young” gene copies from novel Nile tilapia miRNA genes. Orthologs were infered from reconciled gene and species trees obtained with RAxML and TreeBest using predicted data and homologs miRNA data from miRBase (see methodology).

A predictive-based evolutionary analysis of novel mature miRNAs of the Nile tilapia was performed for putative orthologs in 16 other vertebrate genomes (from agnathans to mammals). Several of the 97 novel miRNA of tilapia genes were predicted in other cichlid genomes, such as *Haplochromis burtoni* (37 genes), *Metraclima zebra* (35), *Neolamprologus brichardi* (27) and *Pundamilla nyererei* (33). A few miRNAs were also predicted in *Salmo salar* (8) and *Danio rerio* (2), two representatives of Teleostei genomes. As expected, a smaller number of novel miRNAs was predicted in more distantly related species, such as in the frogs *Xenopus laevis* (2), *X*. *tropicalis* (1) and in the lamprey *Petromyzon marinus* (1) (see Fig. 8A,B for more details).

An additional BLAST search analysis also identified several other homolog sequences (orthologs and paralogs) of novel miRNAs from annotated miRNA genes (pre-miRNAs) of phylogenetically related groups, such as teleostei *Cyprinus carpio* (2 sequences), *Fugu rubripes* (1), *Ictalurus punctatus* (2), *Tetraodon nigroviridis* (2), *Oryzias latipes* (2) and from more distantly related species, such as *Branchiostoma belcheri* (1), *Caenorhabditis remanei* (1), *Anolis carolinensis* (1), *Ophiophagus hannah* (2), *Ornithorhynchus anatinus* (3), *Monodelphis domestica* (2), *Eptesicus fuscus* (1), *Apis mellifera* (1), *Daphnia pulex* (1) and *Ixodes scapularis* (1) (Supplementary Table S12 and S13; Supplementary Fig. S5). The above foregrounds the hypothesis that several novel miRNAs discovered are likely to be ancient miRNAs that accumulated mutations and originated new group-specific variants.

We also inferred orthology and paralogy relationships by comparing miRNA genes in reconciled phylogenetic trees. Among the possible homology relationships (described in detail in the M&M section), this analysis revealed that 1-to-1 orthologs (46.5%) are the most predominant type of homologs within the recently evolved novel miRNAs (Fig. 8C and Supplementary Table S14). This means that only a single putative functional novel miRNA gene copy exists in the genome of the majority of species so far studied. A few species-specific novel miRNA genes with paralogs were exclusively detected in the genome of tilapia, as the three copies of mir-n287, located in regions not yet assigned to LG groups, and the two copies of mir-n363, arranged as clustered copies in the LG13 (Fig. 3C). Other N to N ortologous miRNAs (i.e., miRNAs present as several duplicated copies in distinct species) had paralogs only in ciclhids, as for instance mir-n024, which was predicted to contain two copies in *O*. *niloticus* and *N*. *brichardi*, four copies in *H*. burtoni and five copies in *M*. *zebra*, whereas *P*. *nyererei* have lost one paralog and harbours a unique mir-024 copy. This findings clearly evidence the great dynamism driving the molecular evolution of miRNA paralogs in fish genomes.

The large-scale comparison of homologous genes revealed that novel miRNAs from tilapia emerged at distinct moments in evolutionary history, since they were teleost-specific (96), cichlid-specific (89) or tilapia-specific (55). Duplication and de novo origin are common birth mechanisms driving miRNA genes emergence and accumulation in the genome of metazoans^2,63^. In this sense, novel tilapia-specific or cichlid-specific miRNA genes, with non homologous genes so far, likely have emerged by de novo origination, accumulated mutations, and their maintenance might be tied to the development of lineage specific phenotypic traits and lineage adaptation^1,2,39^. In other words, once a novel miRNA emerged and is expressed, selective forces could feasibly have an effect on mutations that change miRNA biogenesis efficiency and modify the correspondence between the de novo generated miRNA and its target gene, particularly if this new interaction impacts on extant gene regulatory networks.

**Figure 9:**
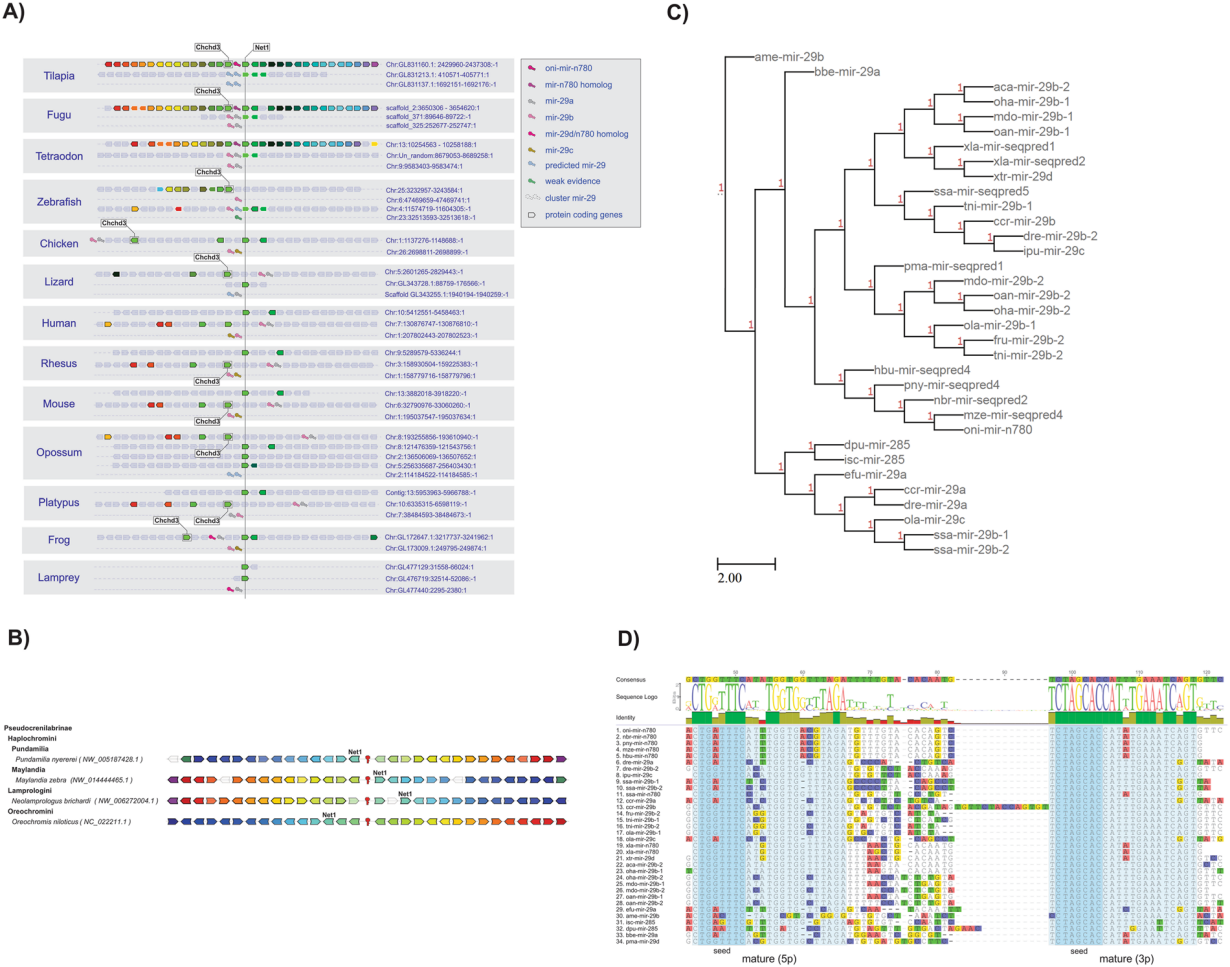
Tracing the evolution of oni-miRNA-n780. (**A**) Syntheny block shows a high conservation among teleost genes. The origin of oni-miRNA-n780 paralogs is associated with extra rounds of genome duplication between in teleosts, since the lambrey presents only one copy of the miRNA cluster 29d/a and cichlids show three copies. In amphibians, reptiles and mammals, oni-miR-n780 paralog could have been lost, while the cluster 29b/a present in mammals or 29d/a present in amphibians might have emerged from a same ancestor paralogs found in lampreys. Syntheny blocks were obtained from Genomicus^95^ and altered with homology data from Ensembl about oni-mir-n780 paralogs from other vertebrate species. (**B**) A manual check using the gene annotation in cichlids and the homology level among genes shows a high conservation of the synthetic block among the cichlid species. However, among *M*. *zebra* and *N*. *brichardi* there was an inversion of the genomic block. Each color represents a different gene and its transparency level represents the homology level (high homology/low transparency to low homology/high transparency). The oni-mir-n780 is represented by the red symbol in the center of the image. (**C**) A reconciled gene and species tree of oni-miR-n780 using RAxML and TreeBest to reconstructing the historical evolutive events among species. We found that the mir-29b from *Apis mellifera* is the closest common miRNA ancestor of oni-miR-n780. (**D**) Alignment of novel mir-n780 precursor sequences with homologs found in lamprey, amphibians and other teleost fish. Sequence similarity of oni-mir-n780 within all species is around 68.8%, although inside cichlid clade it has 100% of identity. The mature miRNA 3p is highly conserved, showing 95.2% of similarity among vertebrats and 100% of similarity when compared among cichlid fishes, *B*. *belcheri*, *X*. *laevis*, *X*. *tropicalis* and *S*. *salar;* by contrast the mature miRNA 5p form is more variable presenting 80.8% of similarity within vertebrates, but still highly conserved in the cichlid genomes where they show of 100% of identity. Another features include a G-to-A mutation in the seed region of the mature miRNA 5p sequences exclusively detected in cichlids, which might refer to a possible specific functional regulatory role of oni-miR-n780 in the genome of cichlids. Alignment data was retrieved from Geneious^96^.

Conversely, the novel oni-miR-n780 is a clear candidate of ancient miRNA with homology to the genome of tunicates, agnathans, fishes, amphibians, reptiles, mammals, and two insect species. Interestingly, we identified arm switching for oni-miR-n780 5p and 3p forms both in an embrionic (2 dpf) and adult stage (muscle), suggesting that this novel miRNA play multiple regulatory roles throughout Nile tilapias’ lifetime (Fig. 5 and Supplementary Table S10). Indeed, oni-miR-n780 may be considered a variant of miR-29 in vertebrates and *B*. *belcheri*, or miR-285 in invertebrates (reported at miRBase in several species) and may act in similar biological processes likewise miR-29, due to its high level of sequence conservation (Fig. 9D). Members of miR-29 family play functional roles in heart development and kidney diseases, as previously verified in humans and mice^79^. Furthermore, the miR-29 expression has been detected in the locomotion apparatus of larvae and worms, being considered important for the evolution of this mechanism among protostomes and deuterostomes^30^.

Taking sequence homology into consideration, the nucleotide similarity at the pre-miR-n780 in all species is approximately 68.8% (Fig. 9D). When miRNAs 5p and 3p were examined separately, it was found that the 3p form of oni-miR-n780 is highly conserved (similarity 95.2%), whereas 5p form had accumulated more variable sites (similarity 80.8%). Further, 5p and 3p are 100% similar to their cichlids’ ortholog. With regard to the genomic context, miR-n780 is located within a syntenic block conserved in the genome of several species and was detected to be highly conserved in cichlidae and in a few teleostei (Fig. 9A,B). Remarkably, the 5p form of miR-n780 carry a G to A mutation in the seed region, common to all cichlidae, and to some tunicates, teleosteans and amphibians. It is interesting to note that sequences matching the zebrafish *D*. *rerio* were not located within the same syntenic block of the oni-miR-n780, and carry several mutations in the 5′ portion of its pre-miRNA.

Additionally, inspection of the phylogenetic relationships reveals that both the oni-miR-n780 and its ortholog in *D*. *rerio* are related to an ancestral miRNA form extant in *Apis mellifera*. However, differently from the copies of *D*. *rerio*, the oni-miR-n780 appears to have evolved from miR-29 and diverged into a clade-specific functional form in the genome of cichlids (Fig. 9C).

### Comparative evolutionary rates of miRNA sequences

In order to verify nucleotide conservation and gain insight into miRNA evolutionary rates, a BLAST search was undertaken matching all tilapia pre-miRNAs against all animal pre-miRNAs annotated in miRBase. Then we focused on the whole pre-miRNA, the mature, seed, cap, loop and tail pre-miRNA regions, to determine whether these regions were under neutral, fast or slow evolutionary rate.

Results revealed that the loop region was the unique miRNA region with a high density of fast-evolving nucleotides (Supplementary Table S11 and Fig. S6). We identified 145 miRNAs with at least one fast-evolving nucleotide (see Supplementary Fig. S7). Five of these miRNAs were associated with arm switching events (Supplementary Fig. S8) and 47 are novel miRNAs, which indicates that these modified mature miRNA transcripts might be either under positive selection or subjected to non-adaptive forces as undergone by young miRNAs^63^. None miRNA within the group of fast-evolving miRNAs (25% of data with higher density sites) presented significant change in arm usage. This fact fails to indicate any correlation between fast evolutionary rate and arm switching. However, 25 novel mature miRNAs were observed among the fast-evolving miRNAs (Fisher’s Exact Test: odd ratio 27.07 and p-value 9.13 × 10^−19^), which indicates a clear correlation between miRNAs age and evolutionary rate.

This comparative analysis of metazoan miRNAs actually corroborate previous findings^37^, reinforcing the expectation of a high percentage of fast-evolving sites at the loop region due to lack of functional constraint or sequence-dependent features for miRNA biogenesis. On the other hand, regions of mature miRNAs or seed regions had a lower density of fast-evolving nucleotides, since changes in these regions may either compromise miRNA-MREs recognition or may induce recognition of other MREs from different target genes, potentially modifying a miRNA network (reviewed by Berezikov, 2011)^2^. This means that mature and seed regions of miRNAs might still accumulate mutations at lower rates, especially nonlethal or neutral mutations that originate variant phenotypes without selective drawback and loss of gene networks evolvability (i.e., the continuous generation of selective genetic variety^80^).

The clear correlation between miRNAs age and evolutionary rate in tilapia also corroborates data from primates^81^ reinforcing the value of this fish as model organism to explore the functional relevance of miRNA evolutionary dynamics in vertebrates.

## Conclusions

Using large-scale sequencing, expression analysis and bioinformatic searches unrestricted to conserved sequences, we discovered several novel miRNAs in tilapia and some novel miRNAs in vertebrates. Genomic context results were consistent with previously published papers showing that novel miRNAs predominantly occur in introns following a parsimonious evolutionary route.

A notable differential ratio and variable prevalence in miRNA arm usage throughout major developmental transitions and adult tissues have been demonstrated. Further, a great number of variant forms of miRNA, highly abundant in early and late life stages in specimens of both sexes, was also identified and characterized, strongly evidencing relevant biological functions of isomiRs. We observed that the complexity of miRNA expression profile depends on the developmental stage, and peaks at 5 dpf. This outcome is an interesting observation of the relationship between evolution and embryology, providing evidence that miRNAs could have played an important role on the onset of vertebrate organisms. Moreover, our evolutionary analysis indicates that novel miRNAs may be ancient miRNAs evolving at a fast pace and originating new group-specific variants.

Finally, we expanded the catalog of miRNAs in the cichlid lineage by “fishing” novel molecules in the Nile tilapia genome. Our findings can be helpful for uncovering molecular biomarkers to assist in Nile tilapia breeding programs^82^, as well as by allowing for a better annotation and analysis of the evolutionary dynamics of the vertebrate miRNome.

## Material and Methods

### Animals and sampling

Specimens used in current study were obtained at Royal Fish farm (Jundiaí, Brazil). Animals were killed by an overdose of MS-222 anesthetic (50 mg/L tricaine-methanensulfonate; Sigma-Aldrich, USA).

Samples for Illumina-sequencing were collected from heart (female), brain (male and female), gonads (male and female), red and white skeletal muscle (male and female), eyes and liver (mixed sexes) of Nile tilapia adults (N = 1 with each sample being a pool of 3 individuals). Additionally, whole naturally spawned embryos (N = 1 with each sample being a pool of 3 samples corresponding to 5 individuals) were sampled at five distinct early developmental stages (2, 3, 4, 5 and 10 dpf). That is, five embryos were pooled together to compose a single sample, and three samples were pooled per sequencing mix (in a total of 15 individuals sequenced per developmental stage). Embryos and adult samples were immediately cooled in liquid nitrogen (for RNA stabilization) and stored at −80 °C until use.

### RNA isolation and quality analysis

Total RNA was extracted with Trizol Reagent® (Invitrogen, USA), following manufacturer’s protocol. RNA samples were treated with TURBO DNase (Ambion, USA) to remove genomic DNA contamination. Final RNA concentration was measured by UV absorbance with NanoDrop 1000 (Thermo Scientific ©, USA), whereas RNA integrity was assessed in the equipment Agilent 2100 Bioanalyzer (Agilent Technologies, USA). All samples presented a good RIN rate (RIN ≥ 8).

### Small RNA library preparation and sequencing

Small RNA library was generated at LC Sciences (Houston, USA) using Illumina Truseq^TM^ Small RNA Preparation kit, following Illumina’s TruSeq^TM^ Small RNA Sample Preparation Guide (Illumina).

Purified cDNA library was used for cluster generation on Illumina’s Cluster Station and then sequenced on Illumina GAIIx instrument. Raw sequencing reads (40 nt) were obtained with Illumina’s Sequencing Control Studio 2.8 (SCS v2.8) following real-time sequencing image analysis and base-calling by Illumina’s RealTime Analysis 1.8.70 (RTA v1.8.70). Extracted sequencing reads were used in deep data analysis.

### Sequence analysis and miRNA identification

Adaptor sequences were trimmed by matching the first 8 bases of the adaptor sequence “TGGAATTC”. FASTQ files were converted into FASTA with a custom Perl script. Reads were counted, collapsed to unique reads and filtered by sequence length (16–26 nts), by sequence complexity (≥3 distinct nucleotides) and sequences matching to other known non-coding RNAs (rRNAs and tRNAs) were removed using the Filter module of UEA small RNA Workbench^83^. Further, reads were exactly matched to tilapia genome (Orenil1.1; MapAssembly.anchored.assembly.fasta downloaded from Ensembl) with PatMaN^84^. Only mapped reads were used for further analysis.

Precursors from miRBase were searched against the tilapia genome using BLAST (e-value ≤ 10^−3^). Reads from all tilapia samples were matched against orthologue precursors, while alignments with sequence coverage were established.

Novel tilapia miRNA loci were predicted with both miRCat^83^ and miRDeep2^85^. Small RNA reads were aligned back to the predicted precursor hairpins and checked manually to ensure that miRNAs were consistent with Dicer and Drosha processing. Line plots were established, showing base coverage by small RNAs across the accepted precursors.

An in-house Python pipeline selected the respective matched reads from each precursor locus and estimated the read counts supposedly originated from two or more precursor loci by weight count (raw read count divided by number of precursor locus aligned), as similarly implemented in miRProf^83^. However, normalization was not applied to raw or weight read counts among samples because it was not necessary for two reasons: (i) we did not perform any Differential Expression analysis (DE) in this study; and (ii) we just evaluated intra-sample ratios among 5p and 3p miRNA reads counts (Fold change values), followed by an indirect comparison using the Fisher’s Exact test and also evaluated the intra-sample miRNA read distribution using Shannon entropy.

Further, an accurate nomenclature was applied to mature miRNA transcripts. The authors took into account small nucleotides variations to identify different members within the same miRNA family (e.g., allocation of let-7 to let-7a and let-7b). miRBase mature sequences were matched against putative tilapia miRNAs and the occurrence rate of orthologous miRNA names was calculated among species. The more common observed name was attributed to each tilapia miRNA, based on the highest degree of similarity to miRBase annotation, followed by the highest occurrence rate among the species. Additionally, mature and precursor miRNA names were manually checked to attribute an accurate annotation. For novel miRNAs, random enumeration was applied and incorporated to the name, followed by “n”, meaning novel miRNA (e.g. mir-n024–1).

Additionally, published papers describing tilapia miRNAs not yet included in miRBase were identified and our reads were matched against their datasets searching for previously detected miRNAs.

### Analysis of IsomiRs

Sequences with highest counts within each arm of pre-miRNA from combined sample datasets were considered canonical mature miRNA reference. After identification, canonical sequences were set apart and variant sequences retained for further analysis.

A polymorphism quantifier based on the alignment of isomiR to canonical sequences was used. First step comprised the identification of nucleotide additions in sequences (named 5p + N, 3p + N or 5p + N/3p + N; where N is the quantity of extra nucleotides in relation to canonical reference sequence). Sequences were aligned to canonical miRNA sequences identified in the previous step. In cases of 100% alignment identity, the isomiR sequence was checked for extra nucleotides at both ends. IsomiR sequences classified in this step were marked to avoid a second classification. Only isomiR sequences with loss of nucleotides remained at the end of the process.

For the classification and quantification of isomiRs, canonical reference sequences were gradually reduced at each extremity (independently at 5′ and 3′ ends) until a maximum of 40% of their original size. Unclassified isomiRs in each reduction were matched to reference trimmed sequences. When a 100% (identity and coverage) hit was detected, sequences were selected for additional verification. This approach confirmed whether there were isomiRs with deletion plus addition of nucleotides in opposite extremities, with subsequent classification per category (5p − N/3p + N or 3p − N/5p + N). If no addition was observed, isomiRs were classified according with deletion and their nucleotide quantity reduced (5p − N or 3p − N). IsomiRs not yet categorized in the previous steps were then processed by considering a gradual reduction at both ends of the canonical miRNA reference sequences (keeping 40% of their original size; 5p − N/3p − N). Finally, isomiRs not assigned to any of the previous categories were classified as “others”, encompassing isoforms that probably match to the loop region of hairpin precursors or were shorter than 40% of the canonical reference sequence.

Results were grouped into additional categories to simplify the classification process of isomiR variants: shorter than the canonical (sequences with nucleotide reductions in 3′ end miRNA sequence extremity); longer than the canonical (sequences with nucleotide addition in 3′ end miRNA sequence extremity), seed shifted miRNAs (any nucleotide variation in 5′ end miRNA sequence extremity disregarding changes in 3′ end); non-template (any nucleotide variation not matched in precursor sequence). An extra identification method was performed to detect non-template sequences. Raw data were reprocessed using data processing steps previously described. However, the sequences were subjected to a filtering to exclude identified miRNAs data, including canonical and isomiRs sequences (shorter, longer, seed shifted) with 100% similarity to pre-miRNA sequences (novel and conserved). Filtered data were then subjected to an additional identification step. A gradual classification (0-to-2 mismatches) was applied to three rounds of alignment processes to pre-miRNAs. Workflow was implemented by an in-house set of Python scripts and Patman software^83^.

### Arm switching

ARS events are significant alterations in the expression levels between 5p and 3p miRNA transcripts. A priori, miRNAs transcripts should be equally expressed from the 5p or 3p miRNAs of the hairpin, although proportions may be altered due to degradation linked to functional constraint. An arm switch may indicate that the same pre-miRNA sequence may play different roles on a specific tissue or developmental stage.

A comparative analysis for each miRNA was undertaken between 5p and 3p miRNA ratios log_2_(ratio(total mapped raw reads 5p and 3p)) for only high confidence miRNAs (≥10 mapped raw read counts in both 5p and 3p) to guarantee reproducibility. In-house scripts in Python were employed.

As criterion for bona fide miRNAs with arm switching events, the fold change measure was employed to calculate the differential prevalence of 5p and 3p transcripts among samples. Fold rates ≥2 and ≤−2 among samples were used so that the clear prevalence for 5p and 3p transcripts detected in at least two samples would be taken into account. Moreover, both 5p and 3p were checked to minimal read counts in the arms (≥10 mapped raw read counts). In addition, Fisher’s statistical exact test (p-value ≤ 0.05) was applied to 5p and 3p miRNA total mapped raw read counts among samples to filter high confidence ARS events.

True positive cases were represented as scatter plots in which each point corresponded to a given sample and 5p and 3p expression levels (log_2_) shown on y-axis and x-axis respectively.

### Entropy

miRNA expression level diversity was accessed by measuring Shannon entropy (Eqs 1 and 2) which quantified the complexity of miRNAs expression profile. Entropy is given by

1 $${\rm{S}}=-\,\sum _{i=1}^{n}{p}_{i}{\mathrm{log}}_{2}{p}_{i}$$

where

2 $${p}_{i}=\frac{{e}_{i}}{{\sum }_{i=1}^{n}{e}_{i}}$$

*e*_*i*_ is the expression of miRNA_*i*_ present in the samples (weight count); *p*_*i*_ corresponds to the probability of selecting miRNA_*i*_ from a given sample; −log_2_ *p*_*i*_ is the information (measured in bits) obtained by observing the event; *S* is the information expected value. In current context, *S* is a measure for the miRNA sample expression profile diversity or, equivalently, the expression profile complexity.

Minimum entropy is obtained if all miRNAs in the sample are qualitatively equal (*p* = *1* for a given miRNA and *p* = *0* for all the others); conversely, maximum value is obtained if all miRNAs are observed with the same frequency (*p*_*i*_ = 1/*n*). In this case, *S* = log_2_ *n*, or rather, maximum entropy depends on the number *n* of available miRNAs. Consequently, there are two main sources for entropy increase: the number of miRNAs and the dispersion of miRNAs expression. Low entropy rates indicate a simple regulated mechanism, either by concentrated expression or by occurring in few miRNAs, while larger entropy rates indicate a complex regulated process caused by the diffuse expression of several miRNAs.

A statistical approach of resample through bootstrapping was also applied. One hundred repetitions were employed and deviated means were calculated over results obtained, although the interval of confidence obtained was small (not shown in the results).

A z-score metrics, which attributed deviation means by confidential inference for comparison of results, was used among samples for comparison (Eq. 3).

3 $$z=\frac{{x}_{i}-\bar{x}}{{\sigma }_{x}}$$

where *x*_*i*_ = sample entropy rate; *X* = average entropy among samples; σ_*x*_ = entropy standard deviation among samples.

### Genomic organization

We characterized and categorized regions as intergenic, exonic or intronic in the tilapia genome hosting miRNA genes. Some rules were applied for the classification. In the case of intergenic miRNAs, no region of the pre-miRNA should overlap a gene region. For intronic miRNAs, the pre-miRNA portion should be located within an intron region. For exonic miRNAs, at least the 5′ pre-miRNA portion should overlap an annotated exon. For both intronic and exonic miRNAs, the pre-miRNA has to be at the same orientation as the annotated gene. Genomic coordinates of miRNAs were obtained from Blast alignment results (previously described), while other genes coordinates were obtained from pre-computed results available at Ensembl web site (Ensembl; *Oreochromis niloticus* gene annotation file; 2015; ftp://ftp.ensembl.org/pub/release-78/gtf/oreochromis_niloticus/Oreochromis_niloticus.Orenil1.0.78.gtf.gz). Coordinates identified and characterized genomic regions. The intersect regions were generated by using in-house Python scripts and checked by UCSC Genome Browser^86^.

A portion of classified data (10%) was manually checked for verification. The coordinates from gene and miRNAs were used in the visualization process with UCSC Genome Browser by Galaxy^87^ to provide a customized visualization.

### Evolutionary analysis

Putative novel species-specific tilapia miRNAs were inspected with regard to their evolutionary conservation in other vertebrates by miRDeep2^85^. miRNAs were tentatively matched against genome sequences of 16 representatives from main vertebrates groups downloaded at NCBI (Genome Database; Bethesda (MD): National Library of Medicine (US), National Center for Biotechnology Information; 2004; http://www.ncbi.nlm.nih.gov/genome/). Novel miRNAs had their canonical and variant sequences mapped and their location determined in the genomes of vertebrates with the module Mapper. After pre-processing, miRDeep2 generated miRNA prediction based on the identification of precursors ability to fold into hairpin structures and their stability according to the minimum free energy of the stem-looped molecule.

So that Nile tilapia miRNA evolutionary rate could be compared to other vertebrates, a Blast search (e-value <= 10^−1^ and coverage >= 70%) was performed against animal miRNAs annotated in miRBase to obtain all homologs for each tilapia miRNA. We also took into account pre-miRNAs predicted previously by miRDeep2 based on novel tilapia annotated miRNAs, including Cichlidae pre-miRNAs.

Multiple alignments were performed with MUSCLE (Edgar, R.C; MUSCLE: multiple sequence alignment with high accuracy and high throughput; 2004; http://www.ebi.ac.uk/Tools/msa/muscle/)^88^. The coordinates of cap 5′, mature 5p, seed 5p, loop, mature 3p, seed 3p and tail 3′ regions were delimited from alignments to compare differential evolution rate separately for each region.

Phylogenetic trees were obtained from whole aligned pre-miRNAs with RAxML from CIPRES portal (Miller, M.A., Pfeiffer W. and Schwartz T; The CIPRES Science Gateway V. 3.1; 2010; http://www.phylo.org/sub_sections/portal/). Default parameters were employed, excepting bootstrap option set at 1000. Multiple alignment and tree data were fitted by PhyloFit, followed by a phyloP score analysis from PHAST package (Hubisz M.J., Pollard K.S. and Siepel A; PHAST: Phylogenetic Analysis with Space/Time Models; 2011; http://compgen.cshl.edu/phast/)^89^.

Nucleotides were classified as slow, neutral and fast-evolving categories based on phyloP scores (−log_10_(p-value)). Positive values were employed to indicate slow evolving categories whilst negative values indicated fast-evolving ones. Absolute value ranged as from −log_10_(p-value), where p-value = 0.05 was used as a threshold to discriminate miRNAs belonging to neutral and fast/slow categories. Each nucleotide on each genomic region was classified under these categories.

Mature miRNAs containing fast-evolving nucleotides were selected and ranked (from a higher density of fast-evolving nucleotides to lower densities of fast-evolving). Sequences containing gaps larger than 3 nts were excluded from the analysis. The top 25% sequences with a higher density of higher evolving nucleotides were classified as fast-evolving miRNAs.

We also investigated whether novel miRNAs were also fast-evolving. Fisher’s Exact test was employed with a cut-off p-value of 0.05.

Orthology and parology relationships were annotated using a phylogenetic tree approach. We followed a pipeline described at Ensembl (Pignatelli, M. *et al*.; ncRNA orthology determination; 2016; https://www.ensembl.org/info/genome/compara/ncRNA_methods.html)^90^ designed for the annotation of orthologs and paralogs non-coding RNAs.

First, we built an initial secondary structure consensus in the RNAalifold^91^ using previously identified homologous miRNAs. Secondary structure outputs alongside with multiple miRNA alignments were used in INFERNAL^92^ to construct and refine the covariance models. After, we built miRNA trees with RAxML using 16 different secondary structure models following a fast (-f a and -x parameters) and a slow strategy (-b parameter) with 1000 bootstrap runs each one. In addtion, we build miRNA multiple alignments with PRANK^93^ and estimated a neighbour-joining (NJ) and a maximum-likelihood (ML) tree using the TreeBeST (Jean-Karim; TreeBeST: Tree Building guided by Species Tree; 2006; http://treesoft.sourceforge.net/treebest.shtml) with 10 bootstrap runs. Species trees were obtained from NCBI database (Taxonomy) and were reconciled with all trees using TreeBeST. Finally, we inferred orthology and paralogy relationships by manually comparing each pair of relative “old” and “young” genes in each miRNA tree^92^. Then, miRNA genes were annotated as follows: paralogs – referring to a pair of genes within a species, emerged though a gene duplication event in their common ancestor or through a de novo origin and subsequent duplicatation in the genome; 1 to 1 orthologs – comprising a pair of genes from distinct species emerged after a speciation event and that correspond to the unique copies of the ancestral gene in their respective species; 1 to N orthologs – representing a pair of genes from distinct species, being one them the unique remaining copy of the ancestor in its species, whereas the other gene has two or more copies in its species; N to N orthologs – naming gene copies in the genome of distinct species, and these genes have been subjeted to duplication events during evolution^92^.

### Ethical note

Methods were carried out in accordance with the guidelines of the Brazilian College for Animal Experimentation (COBEA; http://www.sbcal.org.br/). Assays were approved by the Ethics Committee in Animal Use (CEUA) of UNESP, Botucatu SP Brazil (Protocol#731/2015-CEUA, May 2015).

## Electronic supplementary material

Supplementary Files

Supplementary Dataset 1
