# Supplementary material for: Genome-wide microRNA screening in Nile tilapia reveals pervasive isomiRs’ transcription, sex-biased arm switching and increasing complexity of expression throughout development
Source: Sci Rep. 2018 May 29;8:8248. doi: 10.1038/s41598-018-26607-x (PMC5974277; doi:10.1038/s41598-018-26607-x)
Supplement: Supplementary file 1 — Supplementary Files [file 41598_2018_26607_MOESM1_ESM.pdf]

Genome-wide microRNA screening in Nile tilapia reveals pervasive isomiRs' transcription, sex-biased arm switching and increasing complexity of expression throughout development

Danillo Pinhal[1*#], Luiz A. Bovolenta[2#], Simon Moxon[3], Arthur C. Oliveira[1], Pedro G. Nachtigall[1], Marcio L. Acencio[4], James G. Patton[5], Alexandre W.S. Hilsdorf[6], Ney Lemke[2] and Cesar Martins[7]

Supplementary Files

Supplementary Figure S1. Putative novel Nile tilapia miRNAs compilation.

Compilation of putative novel Nile tilapia miRNAs. The novel pre-miRs loci were predicted using miRDeep[2sr] and miRCat[3sr] tools combination. The predicted mature miRNAs were aligned to pre-miRNAs, and their secondary structure and Minimum Free Energy (MFE) were predicted using Randfold[4sr]. Line plots showed the read alignment coverage indicating the bona-fide Drosha and Dicer pre-miRNA cleavages. The predicted secondary structure were represented by Vienna RNA package[20sr] highlighting mature miRNA-5p and -3p regions. Color scale represents the minimum free energy scale.

mir-n003-1

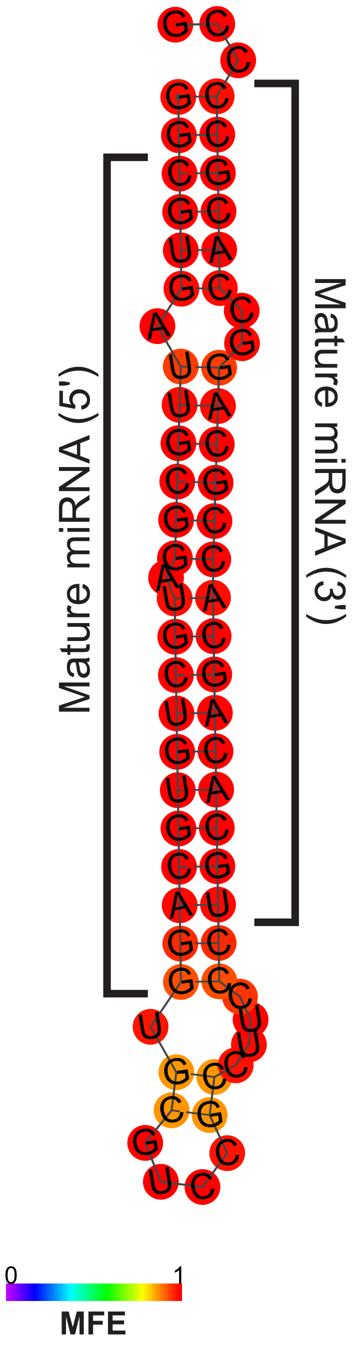

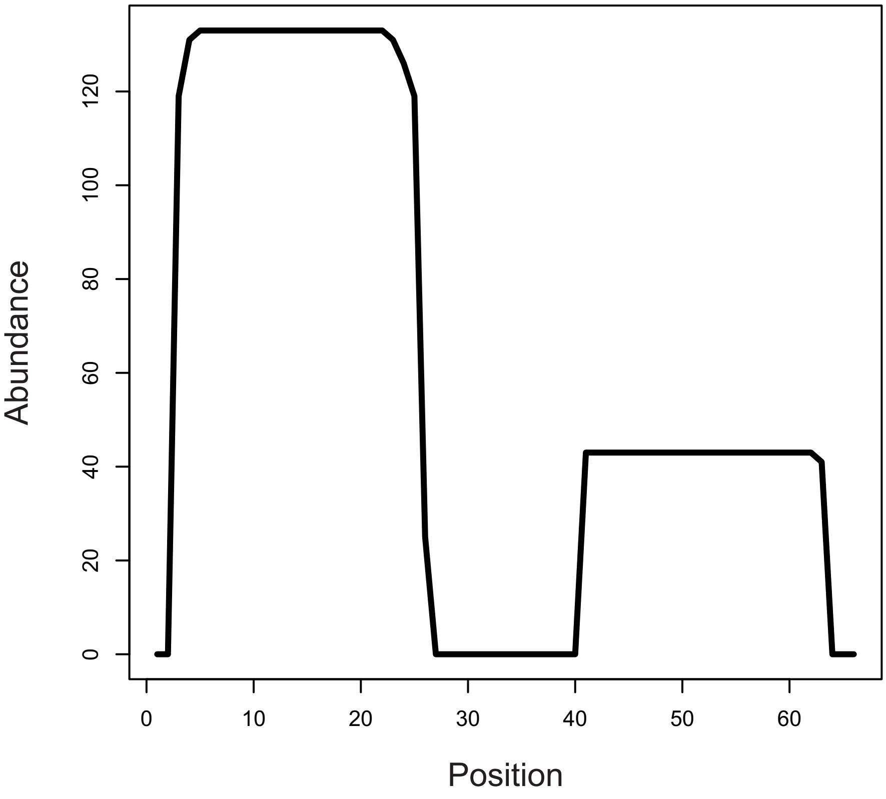


```
GGCGTGATTGCGGATGCTGTGCAGGTGCGTCCGCCTTCCCTGCACAGCACCGCAGGCCACGCCCCG   Raw reads
..CGTGATTGCGGATGCTGTGCAGG.........................................   85
........................................TGCACAGCACCGCAGGCCACGCC...   41
..CGTGATTGCGGATGCTGTGCAGGT........................................   20
...GTGATTGCGGATGCTGTGCAGG.........................................   9
..CGTGATTGCGGATGCTGTGCAG..........................................   7
..CGTGATTGCGGATGCTGTGCA...........................................   5
...GTGATTGCGGATGCTGTGCAGGT........................................   3
..CGTGATTGCGGATGCTGTGC............................................   2
....TGATTGCGGATGCTGTGCAGGT........................................   2
........................................TGCACAGCACCGCAGGCCACGC....   2

((((((.((((((.(((((((((((.((....))....)))))))))))))))))..))))))...   (-44.40 MFE)
```

mir-n012

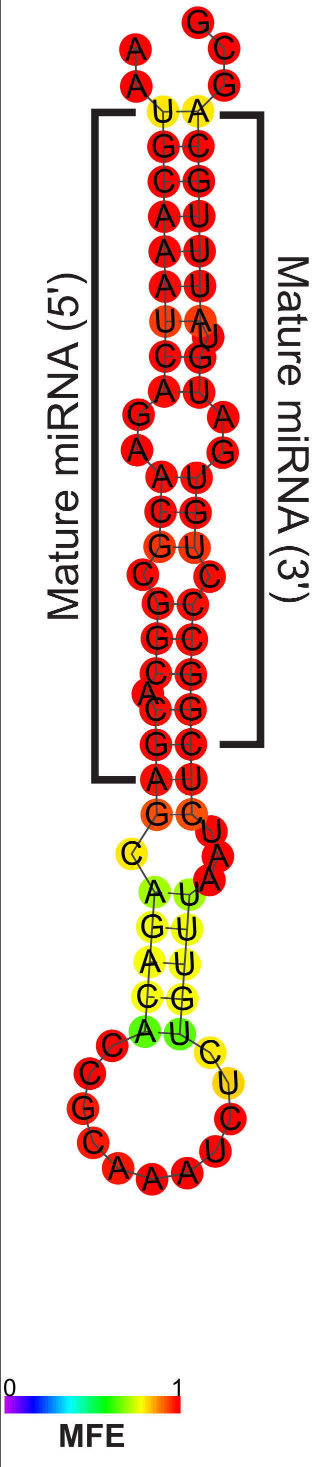

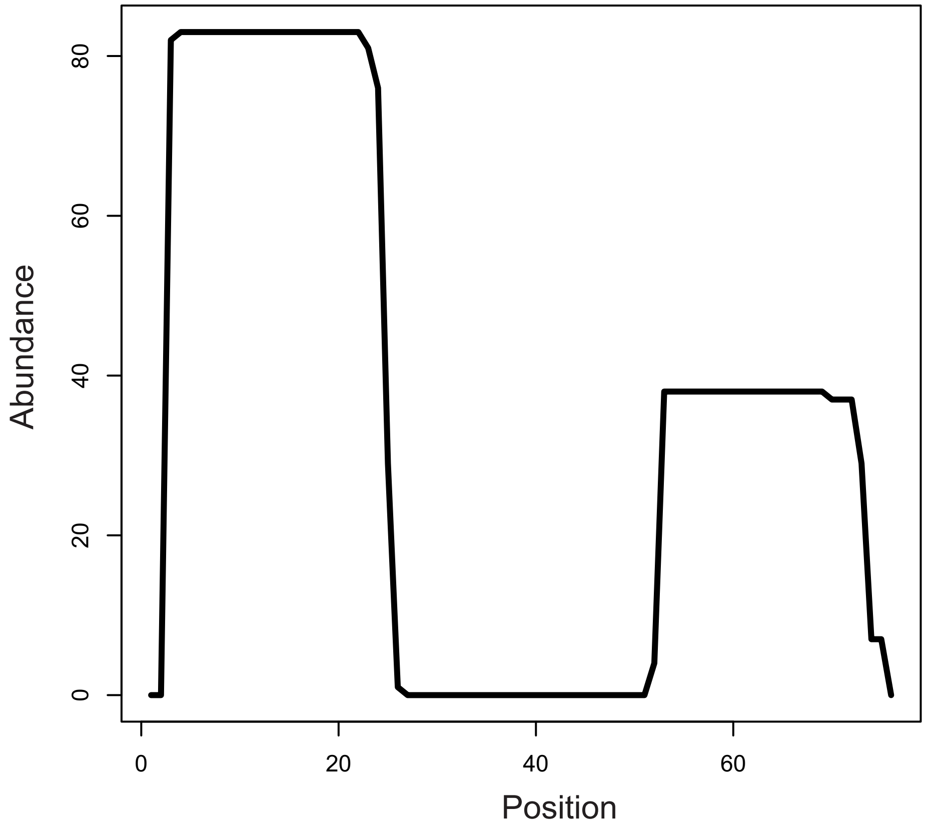


```
AATGCAAATCAGAACGCGGCACGAGCAGACACCGCAAATCTCTGTTTAATCTCGGCCCTGTGATGTATTTGCAGCG      Raw Reads
..TGCAAATCAGAACGCGGCACGA....................................................      47
..TGCAAATCAGAACGCGGCACGAG...................................................      27
....................................................CGGCCCTGTGATGTATTTGCA...      19
....................................................CGGCCCTGTGATGTATTTGC....      7
....................................................CGGCCCTGTGATGTATTTGCAGC.      7
..TGCAAATCAGAACGCGGCACG.....................................................      5
...................................................TCGGCCCTGTGATGTATTTGCA...      3
..TGCAAATCAGAACGCGGCAC......................................................      2
...GCAAATCAGAACGCGGCACGAG...................................................      1
..TGCAAATCAGAACGCGGCACGAGC..................................................      1
....................................................CGGCCCTGTGATGTATT.......      1
...................................................TCGGCCCTGTGATGTATTTGC....      1

..(((((((((..(((.(((.((((.(((((...........)))))...))))))).)))..)).)))))))... (-23.30 MFE)
```

mir-n024-1

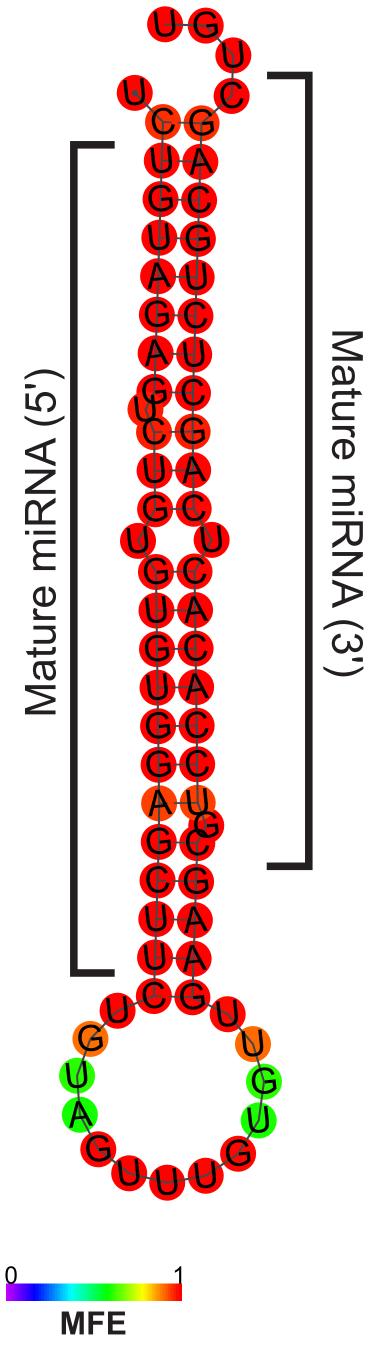

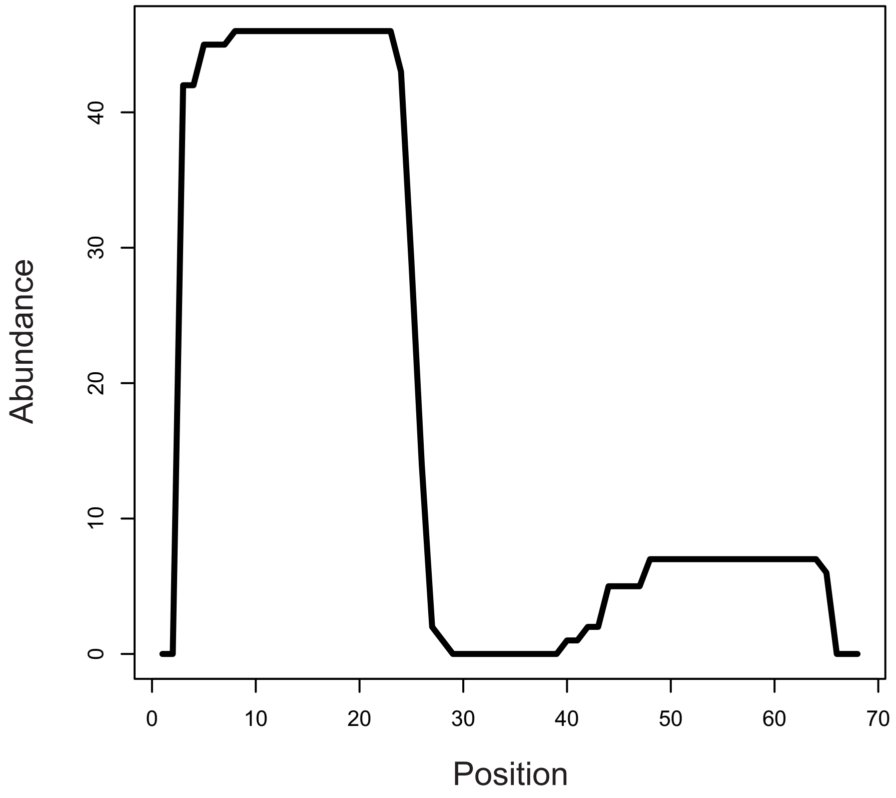


```
TCTGTAGAGTCTGTGTGTGGAGCTTCTGTAGTTTGTGTTGAAGCGTCCACACTCAGCTCTGCAGCTGT      Raw reads
..TGTAGAGTCTGTGTGTGGAGCTT...........................................      15
..TGTAGAGTCTGTGTGTGGAGCT............................................      14
..TGTAGAGTCTGTGTGTGGAGCTTC..........................................      9
....TAGAGTCTGTGTGTGGAGCTTC..........................................      3
...........................................CGTCCACACTCAGCTCTGCAGC...      3
..TGTAGAGTCTGTGTGTGGAGC.............................................      2
.......AGTCTGTGTGTGGAGC.............................................      1
..TGTAGAGTCTGTGTGTGGAGCTTCT.........................................      1
..TGTAGAGTCTGTGTGTGGAGCTTCTG........................................      1
...............................................CACACTCAGCTCTGCAG....      1
.......................................GAAGCGTCCACACTCAGCTCTGCAGC...      1
.........................................AGCGTCCACACTCAGCTCTGCAGC...      1
...............................................CACACTCAGCTCTGCAGC...      1

.((((((((.(((.((((((((((((.............))))).))))))).)))))))))))....      (-35.52  MFE)
```

mir-n024-2

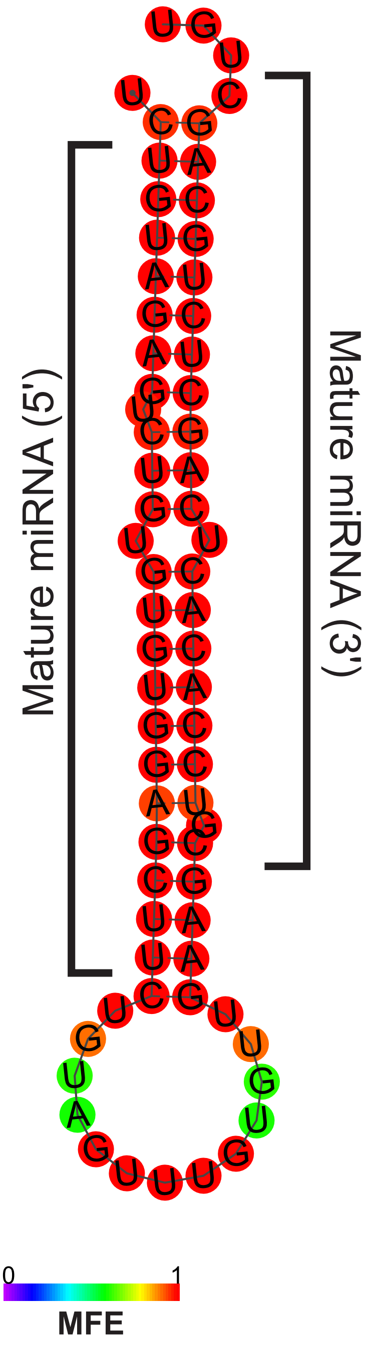

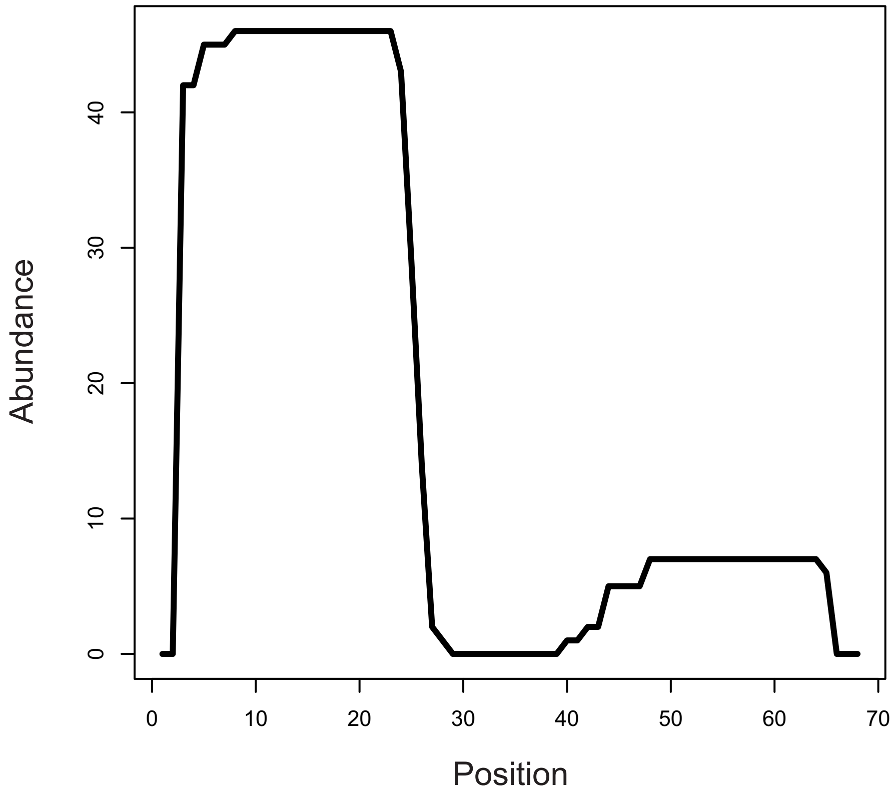


```
TCTGTAGAGTCTGTGTGTGGAGCTTCTGTAGTTTGTGTTGAAGCGTCCACACTCAGCTCTGCAGCTGT       Raw reads
..TGTAGAGTCTGTGTGTGGAGCTT...........................................       15
..TGTAGAGTCTGTGTGTGGAGCT............................................       14
..TGTAGAGTCTGTGTGTGGAGCTTC..........................................       9
....TAGAGTCTGTGTGTGGAGCTTC..........................................       3
...........................................CGTCCACACTCAGCTCTGCAGC...       3
..TGTAGAGTCTGTGTGTGGAGC.............................................       2
.......AGTCTGTGTGTGGAGC.............................................       1
..TGTAGAGTCTGTGTGTGGAGCTTCT.........................................       1
..TGTAGAGTCTGTGTGTGGAGCTTCTG........................................       1
...............................................CACACTCAGCTCTGCAG....       1
.......................................GAAGCGTCCACACTCAGCTCTGCAGC...       1
.........................................AGCGTCCACACTCAGCTCTGCAGC...       1
...............................................CACACTCAGCTCTGCAGC...       1

.((((((((.(((.((((((((((((.............))))).))))))).)))))))))))....       (-35.52 MFE)
```

mir-n057

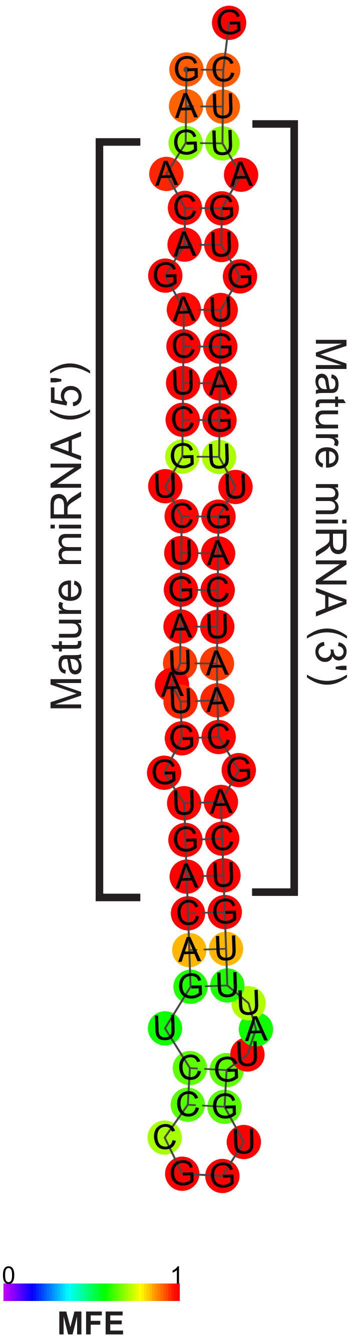

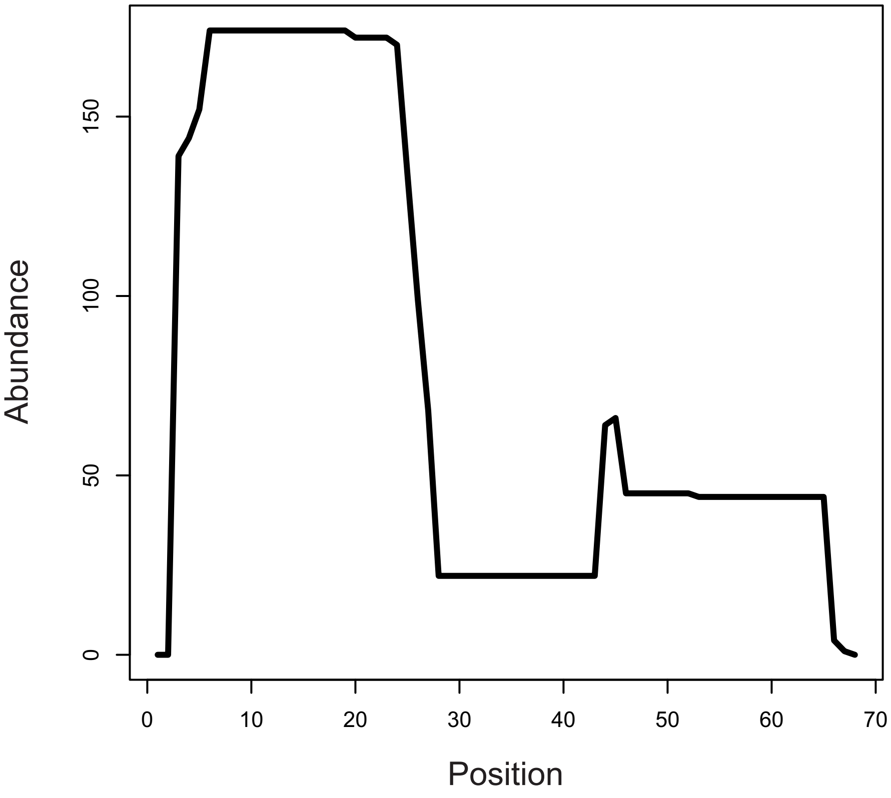


```
GAGACAGACTCGTCTGATATGGTGACAGTCCCGGTGGTATTTGTCAGCAATCAGTTGAGTGTGATTCG      Raw reads
..GACAGACTCGTCTGATATGGTGA...........................................      51
...........................................TCAGCAATCAGTTGAGTGTGAT...      40
..GACAGACTCGTCTGATATGGTG............................................      35
..GACAGACTCGTCTGATATGGTGAC..........................................      29
.........................CAGTCCCGGTGGTATTTGTC.......................      21
..GACAGACTCGTCTGATATGGTGACA.........................................      20
.....AGACTCGTCTGATATGGTGACA.........................................      20
....CAGACTCGTCTGATATGGTGACA.........................................      6
...ACAGACTCGTCTGATATGGTGA...........................................      3
..GACAGACTCGTCTGATA.................................................      2
..GACAGACTCGTCTGATATGGT.............................................      2
.....AGACTCGTCTGATATGGTGA...........................................      2
...ACAGACTCGTCTGATATGGTGAC..........................................      2
...........................................TCAGCAATCAGTTGAGTGTGATT..      2
....CAGACTCGTCTGATATGGTG............................................      1
....CAGACTCGTCTGATATGGTGAC..........................................      1
..........................AGTCCCGGTGGTATTTGTCAGCAATC................      1
............................................CAGCAATCAGTTGAGTGTGATT..      1
............................................CAGCAATCAGTTGAGTGTGATTC.      1

(((.((.(((((.(((((.((.((((((.((....))...)))))).))))))).))))).)).))).      (-26.90 MFE)
```

mir-n067

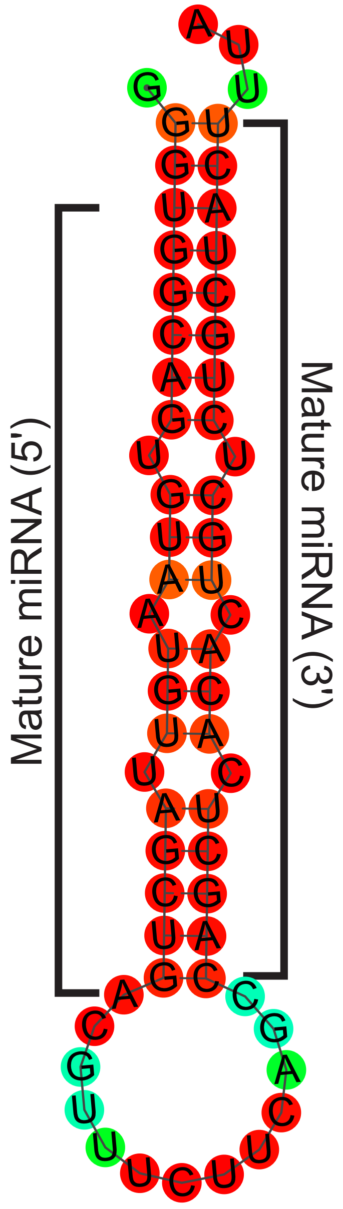

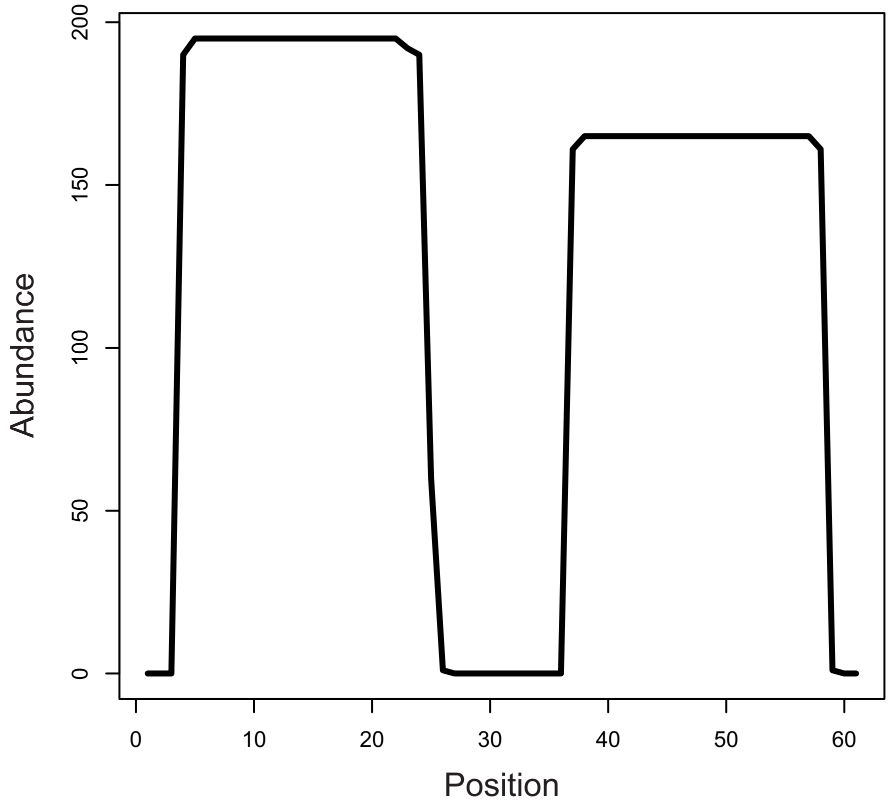


```
GGGTGGCAGTGTAATGTTAGCTGACGTTTCTTCAGCCAGCTCACACTGCTCTGCTACTTTA    Raw reads
....................................CAGCTCACACTGCTCTGCTACT...    156
...TGGCAGTGTAATGTTAGCTGA.....................................    126
...TGGCAGTGTAATGTTAGCTGAC....................................    58
....GGCAGTGTAATGTTAGCTGA.....................................    4
....................................CAGCTCACACTGCTCTGCTAC....    4
.....................................AGCTCACACTGCTCTGCTACT...    4
...TGGCAGTGTAATGTTAGCT.......................................    3
...TGGCAGTGTAATGTTAGCTG......................................    2
....GGCAGTGTAATGTTAGCTGAC....................................    1
...TGGCAGTGTAATGTTAGCTGACG...................................    1
....................................CAGCTCACACTGCTCTGCTACTT..    1
(((((((((.(((.(((.(((((.(.........).))))).))).))).))))))))).. (-24.90 MFE)
```

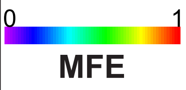

mir-n106

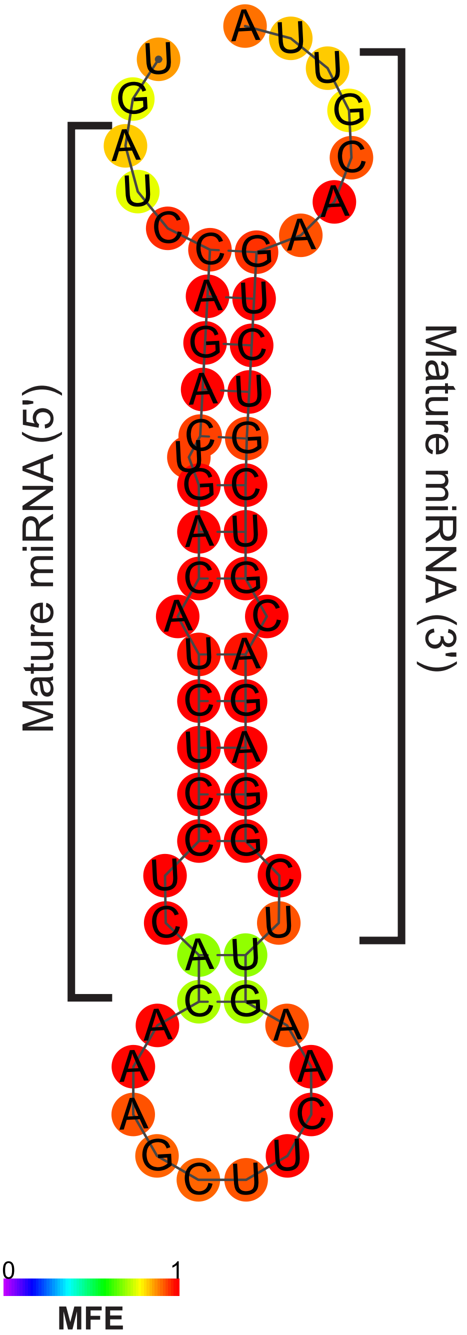

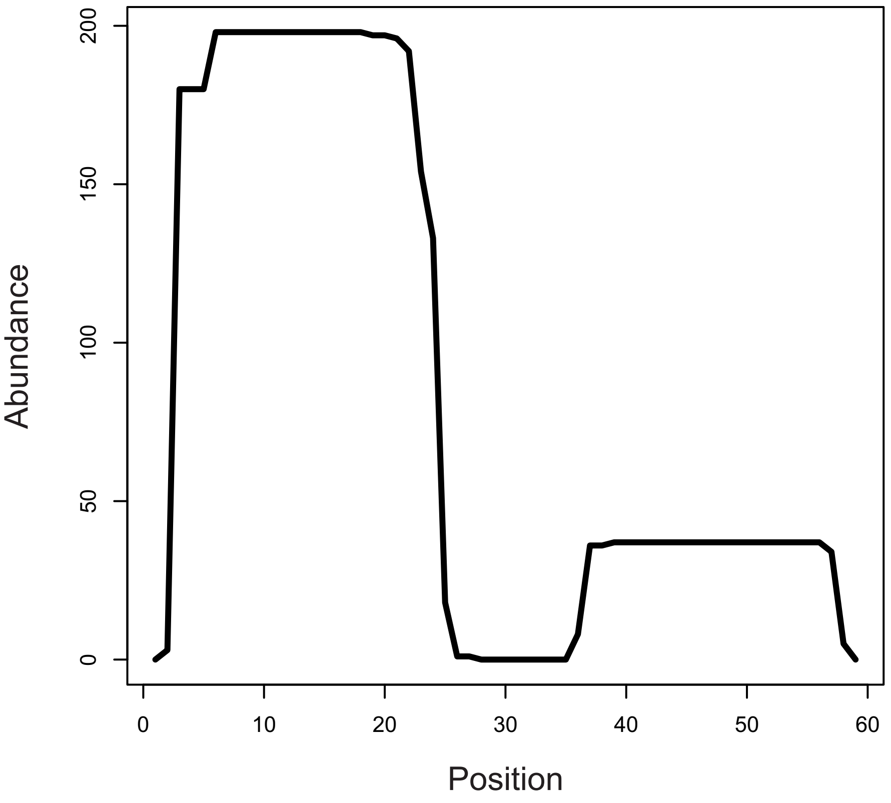


```
TGATCCAGACTGACATCTCCTCACAAAGCTTCAAGTTCGGAGACGTCGTCTGAACGTTA      Raw reads
..ATCCAGACTGACATCTCCTCAC...................................      105
..ATCCAGACTGACATCTCCTC.....................................      31
....................................TCGGAGACGTCGTCTGAACGT..      26
..ATCCAGACTGACATCTCCTCA....................................      19
..ATCCAGACTGACATCTCCTCACA..................................      15
.....CAGACTGACATCTCCTCAC...................................      9
.....CAGACTGACATCTCCTC.....................................      5
..ATCCAGACTGACATCTCCT......................................      4
...................................TTCGGAGACGTCGTCTGAACGTT.      4
.GATCCAGACTGACATCTCCTC.....................................      2
.....CAGACTGACATCTCCTCA....................................      2
.....CAGACTGACATCTCCTCACA..................................      2
...................................TTCGGAGACGTCGTCTGAACG...      2
...................................TTCGGAGACGTCGTCTGAACGT..      2
..ATCCAGACTGACATCT.........................................      1
..ATCCAGACTGACATCTCC.......................................      1
.GATCCAGACTGACATCTCCTCAC...................................      1
..ATCCAGACTGACATCTCCTCACAAA...............................      1
....................................TCGGAGACGTCGTCTGAACG...      1
......................................GGAGACGTCGTCTGAACGT..      1
....................................TCGGAGACGTCGTCTGAACGTT.      1

.....(((((.(((.(((((..((..........))..))))).)))))))).......      (-17.60 MFE)
```

mir-n128-1

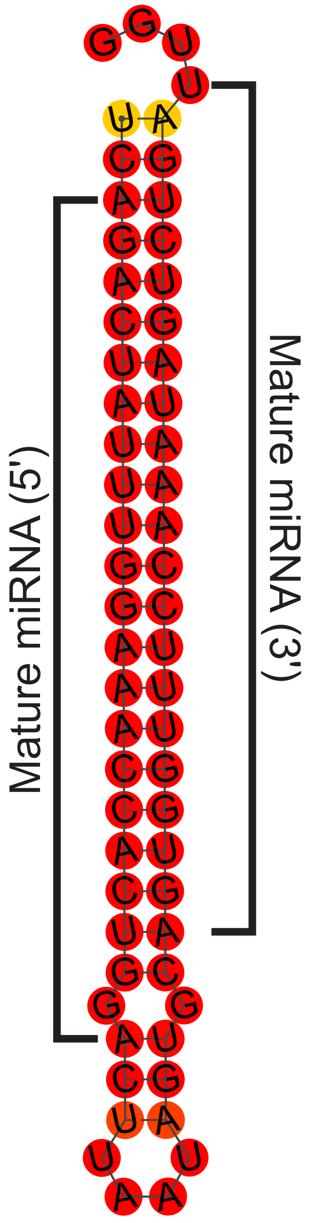

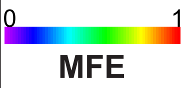

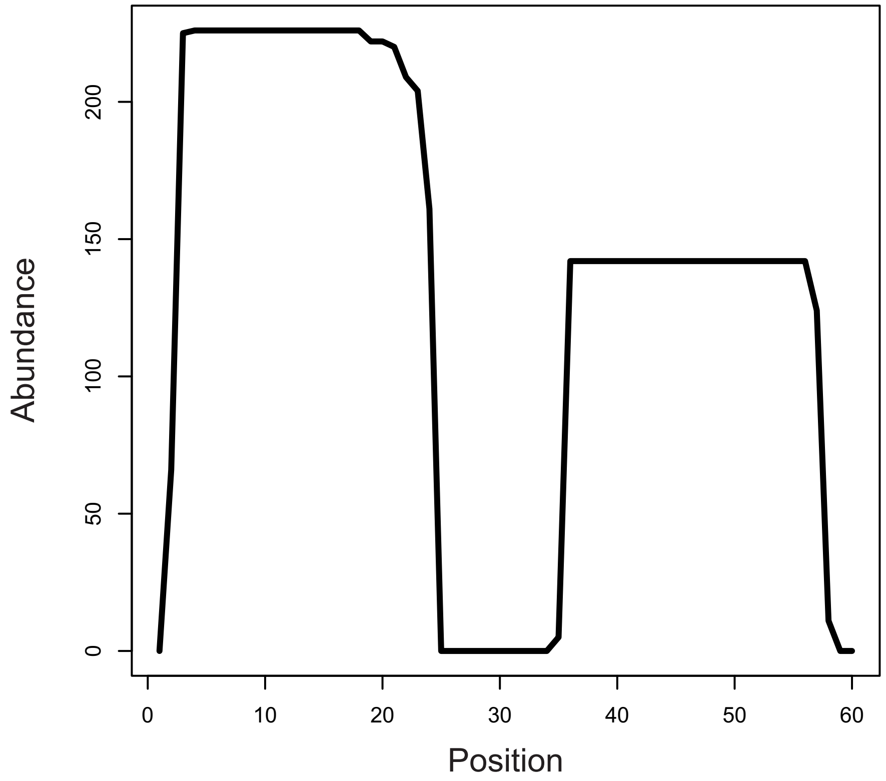


```
TCAGACTATTTGGAAACCACTGGACTTAATAGTGCAGTGGTTTCCAAATAGTCTGATTGG      Raw read
..AGACTATTTGGAAACCACTGGA....................................      145
...................................AGTGGTTTCCAAATAGTCTGAT...      113
.CAGACTATTTGGAAACCACTGG.....................................      34
.CAGACTATTTGGAAACCACTGGA....................................      15
...................................AGTGGTTTCCAAATAGTCTGA....      13
...................................AGTGGTTTCCAAATAGTCTGATT..      11
.CAGACTATTTGGAAACCACT.......................................      9
..AGACTATTTGGAAACCACTGG.....................................      9
..................................CAGTGGTTTCCAAATAGTCTGA....      5
.CAGACTATTTGGAAACCACTG......................................      4
.CAGACTATTTGGAAACC..........................................      3
..AGACTATTTGGAAACCACT.......................................      2
..AGACTATTTGGAAACC..........................................      1
.CAGACTATTTGGAAACCAC........................................      1
..AGACTATTTGGAAACCAC........................................      1
..AGACTATTTGGAAACCACTG......................................      1
...GACTATTTGGAAACCACTGGA....................................      1


((((((((((((((((((((((.(((....))).)))))))))))))))))))))).... (-41.70 MFE)
```

mir-n128-2

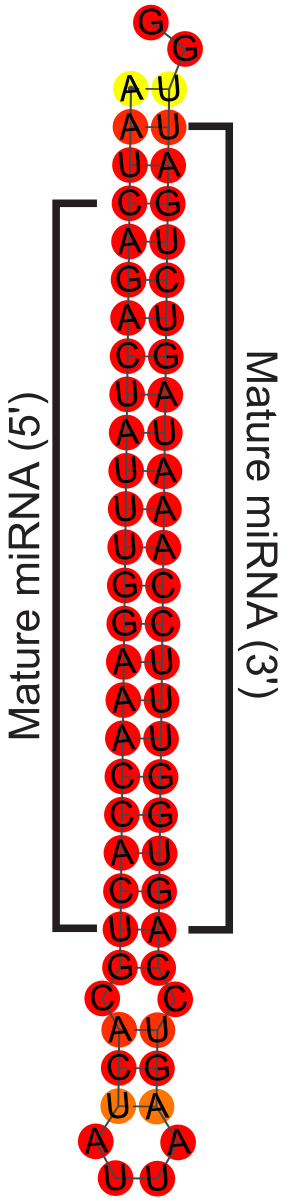

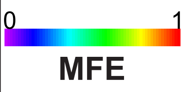

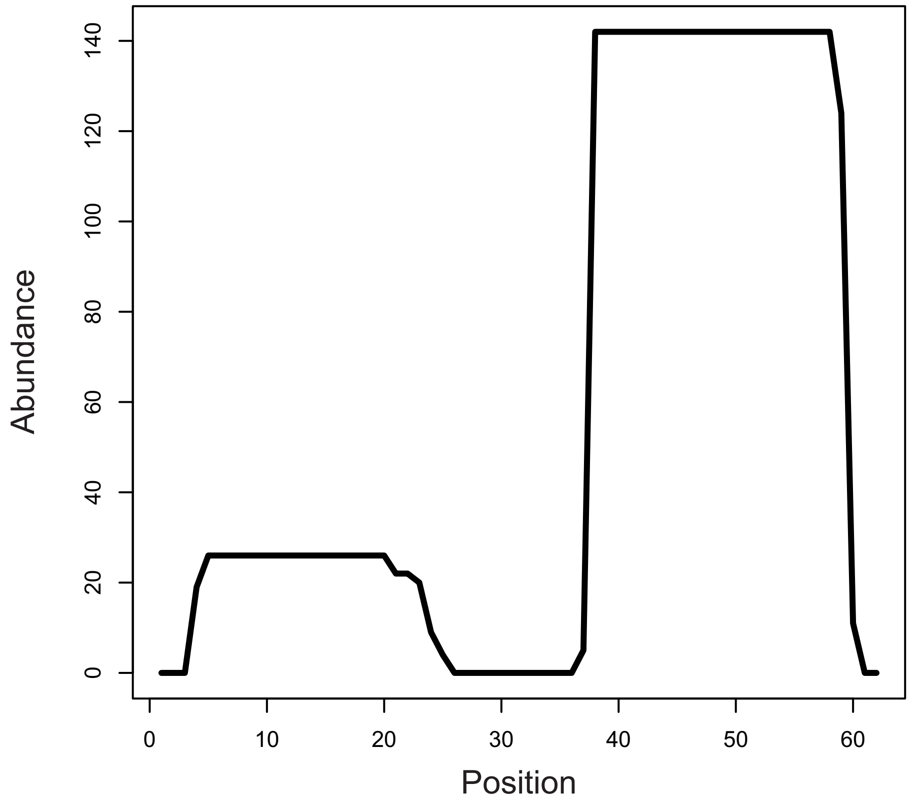


```
AATCAGACTATTTGGAAACCACTGCACTATTAAGTCCAGTGGTTTCCAAATAGTCTGATTGG   Raw reads
.....................................AGTGGTTTCCAAATAGTCTGAT...   113
.....................................AGTGGTTTCCAAATAGTCTGA....   13
.....................................AGTGGTTTCCAAATAGTCTGATT..   11
...CAGACTATTTGGAAACCACT.......................................   9
....................................CAGTGGTTTCCAAATAGTCTGA....   5
...CAGACTATTTGGAAACCACTG......................................   4
...CAGACTATTTGGAAACC..........................................   3
....AGACTATTTGGAAACCACT.......................................   2
...CAGACTATTTGGAAACCACTGC.....................................   2
....AGACTATTTGGAAACCACTGC.....................................   2
....AGACTATTTGGAAACC..........................................   1
...CAGACTATTTGGAAACCAC........................................   1
....AGACTATTTGGAAACCAC........................................   1
....AGACTATTTGGAAACCACTG......................................   1

((((((((((((((((((((((((.(((....))).)))))))))))))))))))))))).. (-41.40 MFE
```

mir-n180

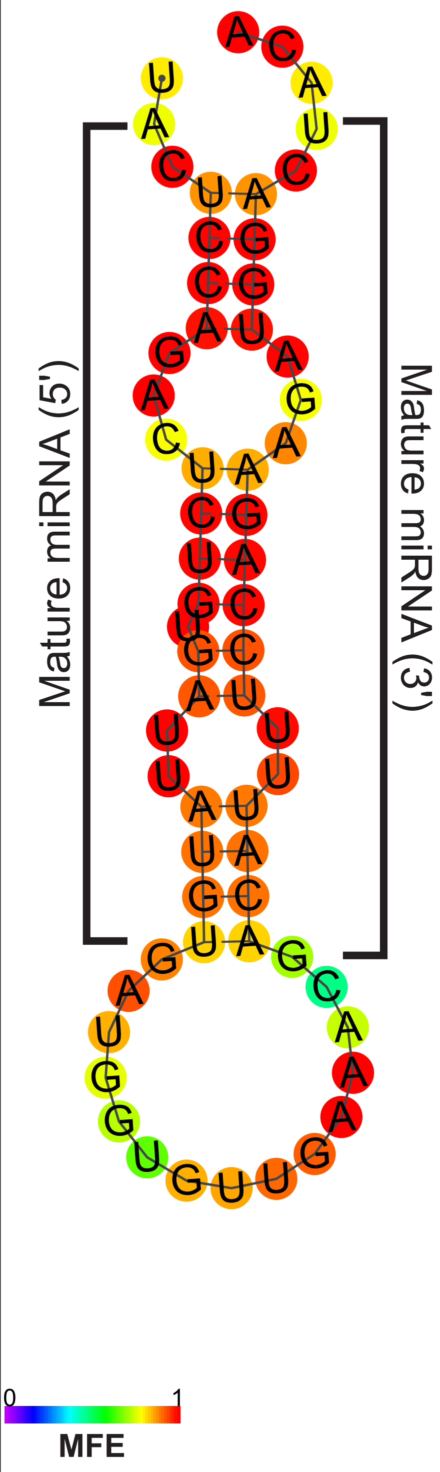

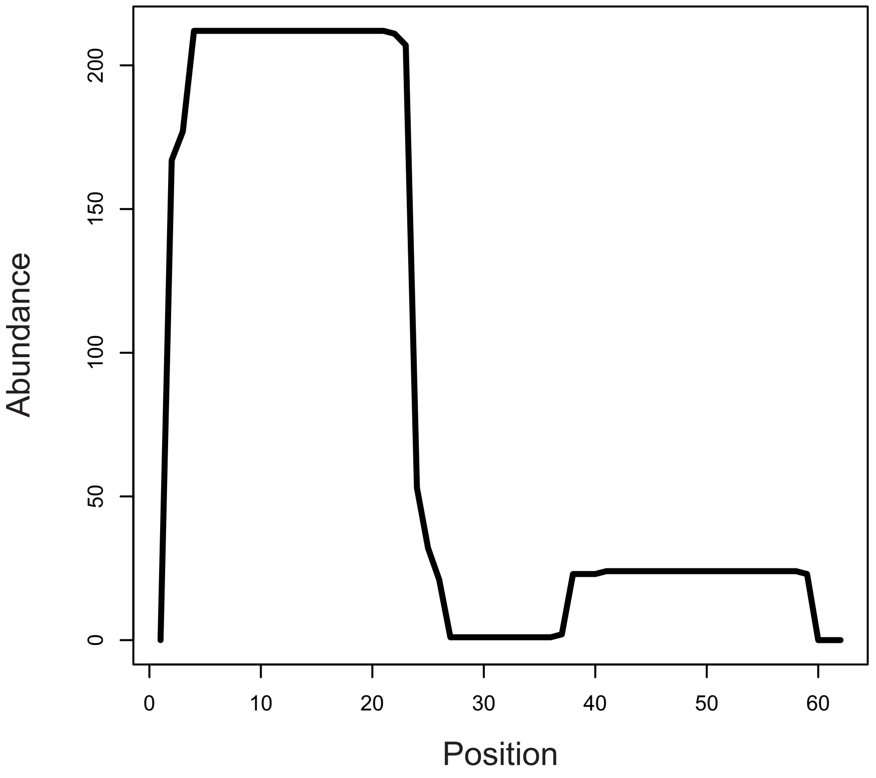


```
TACTCCAGACTCTGTGATTATGTGATGGTGTTGAAACGACATTTTCCAGAAGATGGACTACA   Raw Reads
.ACTCCAGACTCTGTGATTATGT.......................................   152
...TCCAGACTCTGTGATTATGTGAT....................................   20
.....................................GACATTTTCCAGAAGATGGACT...   20
.ACTCCAGACTCTGTGATTATGTG......................................   11
...TCCAGACTCTGTGATTATGTGA.....................................   11
..CTCCAGACTCTGTGATTATGTG......................................   8
.ACTCCAGACTCTGTGATTATG........................................   4
...TCCAGACTCTGTGATTATGT.......................................   2
...TCCAGACTCTGTGATTATGTG......................................   2
........................................ATTTTCCAGAAGATGGACT...   2
..CTCCAGACTCTGTGATTAT.........................................   1
..CTCCAGACTCTGTGATTATGT.......................................   1
.......................GATGGTGTTGAAACGAC......................   1
.....................................GACATTTTCCAGAAGATGGAC....   1
....................................CGACATTTTCCAGAAGATGGACT...   1

...((((...((((.((..((((....((......)).))))..))))))...)))).....  (-12.90 MFE)
```

mir-n260-1

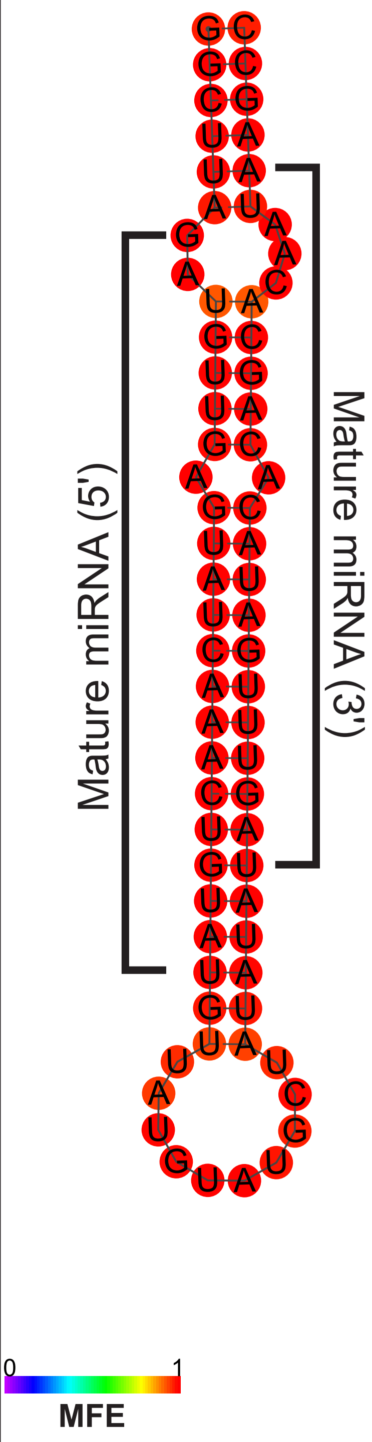

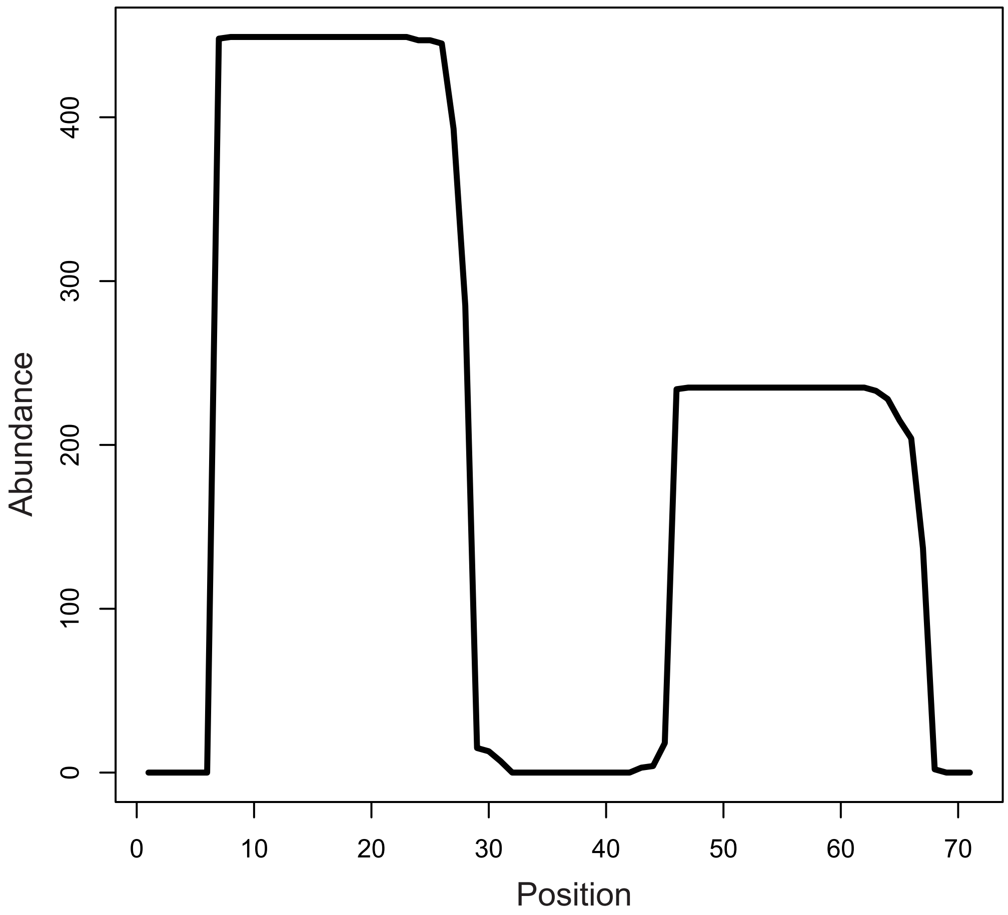


```
GGCTTAGATGTTGAGTATCAAACTGTATGTTATGTATGCTATATATAGTTTGATACACAGCACAATAAGCC   Raw reads
......GATGTTGAGTATCAAACTGTAT...........................................   270
.............................................TAGTTTGATACACAGCACAATA....   133
......GATGTTGAGTATCAAACTGTA............................................   108
.............................................TAGTTTGATACACAGCACAAT.....   56
......GATGTTGAGTATCAAACTGT.............................................   52
.............................................TAGTTTGATACACAGCACA.......   10
.............................................TAGTTTGATACACAGCACAA......   10
............................................ATAGTTTGATACACAGCACAAT.....   9
......GATGTTGAGTATCAAACTGTATGTT........................................   7
......GATGTTGAGTATCAAACTGTATGT.........................................   6
.............................................TAGTTTGATACACAGCAC........   5
............................................ATAGTTTGATACACAGCACA.......   3
......GATGTTGAGTATCAAACTG..............................................   2
......GATGTTGAGTATCAAACTGTATG..........................................   2
..........................................ATATAGTTTGATACACAGCA.........   2
............................................ATAGTTTGATACACAGCACAATA....   2
.............................................TAGTTTGATACACAGCACAATAA...   2
......GATGTTGAGTATCAAAC................................................   1
.......ATGTTGAGTATCAAAC................................................   1
..........................................ATATAGTTTGATACACAGCACAA......   1
...........................................TATAGTTTGATACACAGCACAAT.....   1
..............................................AGTTTGATACACAGCACAAT.....   1

((((((..(((((.((((((((((((((((...(....).))))))))))))))))).)))))...)))))) (-32.30 MFE)
```

mir-n260-2

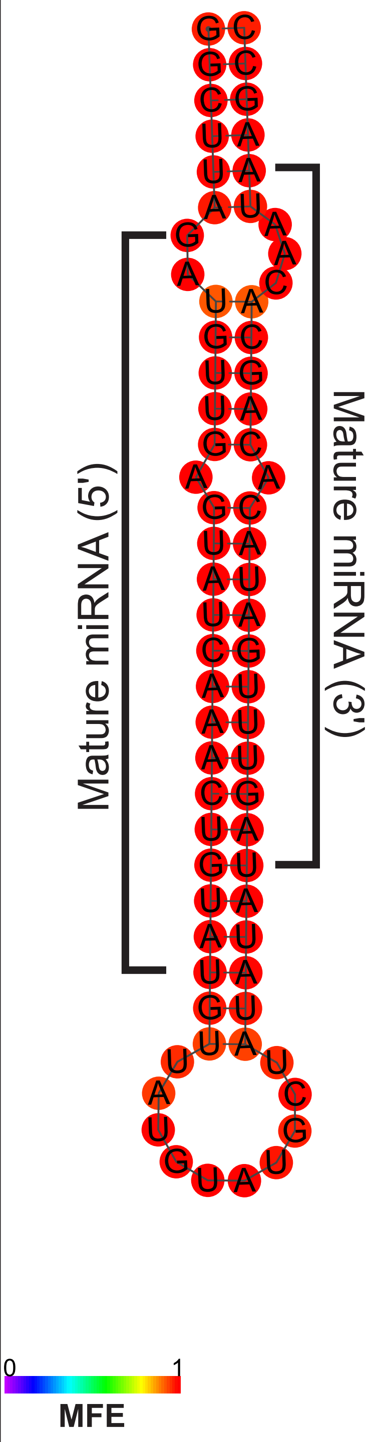

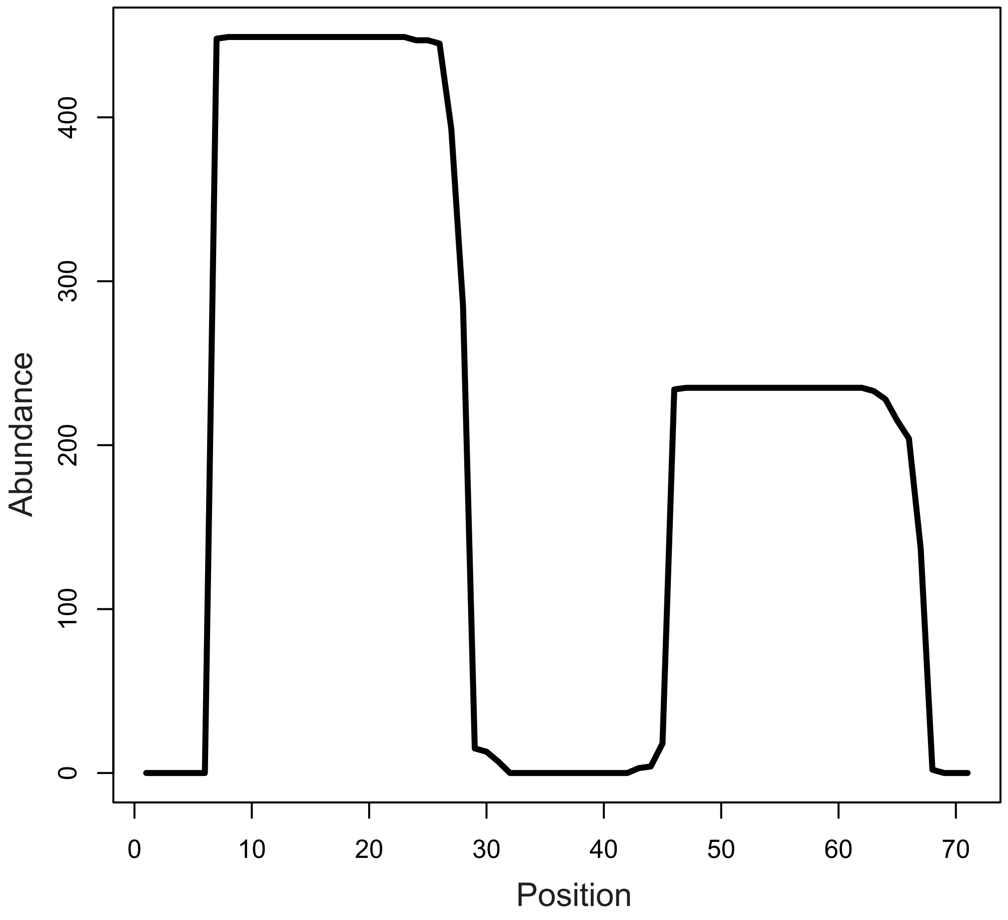


```
GGCTTAGATGTTGAGTATCAAACTGTATGTTATGTATGCTATATATAGTTTGATACACAGCACAATAAGCC  Raw reads
......GATGTTGAGTATCAAACTGTAT...........................................  270
.............................................TAGTTTGATACACAGCACAATA....  133
......GATGTTGAGTATCAAACTGTA............................................  108
.............................................TAGTTTGATACACAGCACAAT.....  56
......GATGTTGAGTATCAAACTGT.............................................  52
.............................................TAGTTTGATACACAGCACA.......  10
.............................................TAGTTTGATACACAGCACAA......  10
............................................ATAGTTTGATACACAGCACAAT.....  9
......GATGTTGAGTATCAAACTGTATGTT........................................  7
......GATGTTGAGTATCAAACTGTATGT.........................................  6
.............................................TAGTTTGATACACAGCAC........  5
............................................ATAGTTTGATACACAGCACA.......  3
......GATGTTGAGTATCAAACTG..............................................  2
......GATGTTGAGTATCAAACTGTATG..........................................  2
..........................................ATATAGTTTGATACACAGCA.........  2
............................................ATAGTTTGATACACAGCACAATA....  2
.............................................TAGTTTGATACACAGCACAATAA...  2
......GATGTTGAGTATCAAAC................................................  1
.......ATGTTGAGTATCAAAC................................................  1
..........................................ATATAGTTTGATACACAGCACAA......  1
...........................................TATAGTTTGATACACAGCACAAT.....  1
..............................................AGTTTGATACACAGCACAAT.....  1

((((((..(((((.((((((((((((((((...(....).)))))))))))))))).)))))...))))))  (-32.30 MFE
```

mir-n270

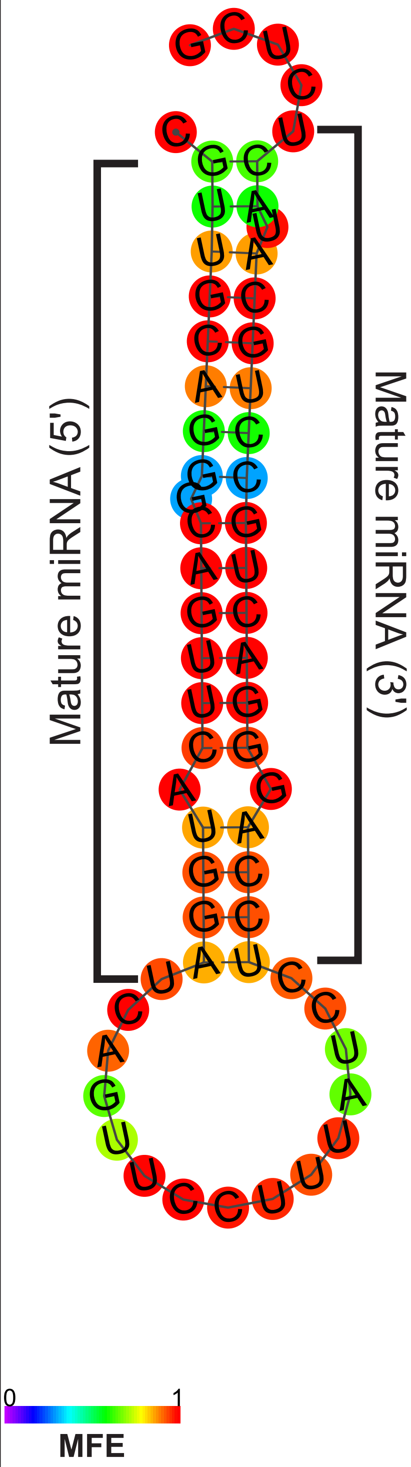

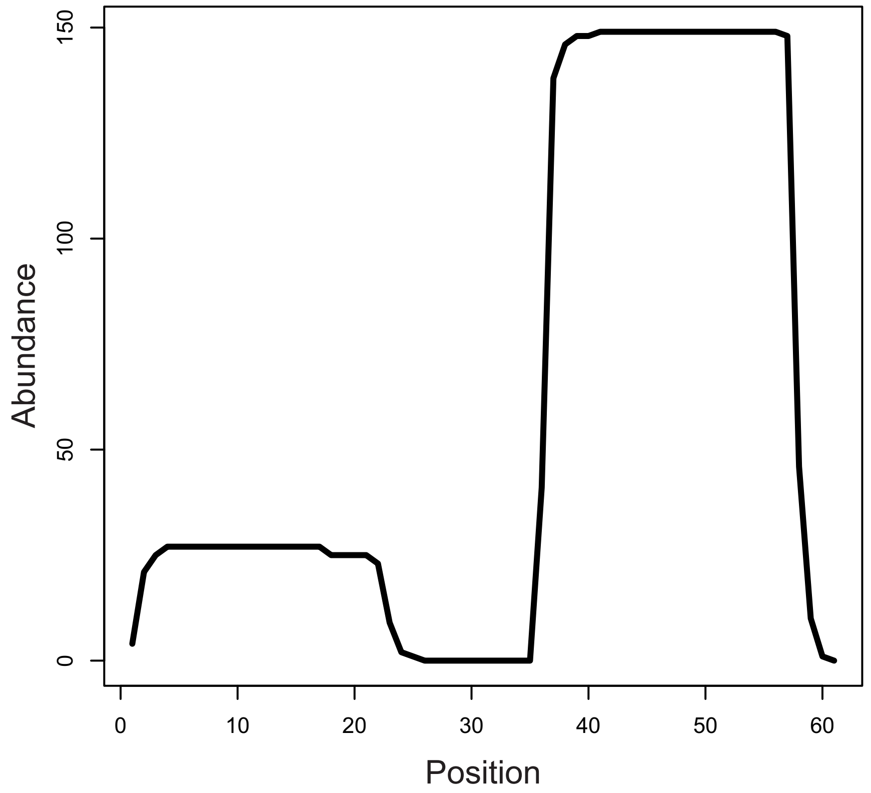


```
CGTTGCAGGGCAGTTCATGGATCAGTTCCTTTATCCTCCAGGGACTGCCTGCATACTCTCG     Raw read
....................................TCCAGGGACTGCCTGCATACT....     60
...................................CTCCAGGGACTGCCTGCATACT....     40
....................................TCCAGGGACTGCCTGCATACTC...     31
.GTTGCAGGGCAGTTCATGGAT.......................................     11
....................................TCCAGGGACTGCCTGCATACTCT..     6
.....................................CCAGGGACTGCCTGCATACTC...     4
.GTTGCAGGGCAGTTCATGGATC......................................     3
.GTTGCAGGGCAGTTCATGGA........................................     2
CGTTGCAGGGCAGTTCATGGAT.......................................     2
..TTGCAGGGCAGTTCATGGATC......................................     2
.....................................CCAGGGACTGCCTGCATACT....     2
.....................................CCAGGGACTGCCTGCATACTCT..     2
CGTTGCAGGGCAGTTCA............................................     1
.GTTGCAGGGCAGTTCA............................................     1
..TTGCAGGGCAGTTCATGGAT.......................................     1
CGTTGCAGGGCAGTTCATGGATC......................................     1
...TGCAGGGCAGTTCATGGATC......................................     1
...TGCAGGGCAGTTCATGGATCA.....................................     1
..TTGCAGGGCAGTTCATGGATCAG....................................     1
...................................CTCCAGGGACTGCCTGCATAC.....     1
........................................GGGACTGCCTGCATACTC...     1
......................................CAGGGACTGCCTGCATACTCT..     1
......................................CAGGGACTGCCTGCATACTCTC.     1

.((((((((.((((((.((((...............)))).)))))))))))).)).....  (-22.66 MFE)
```

mir-n329

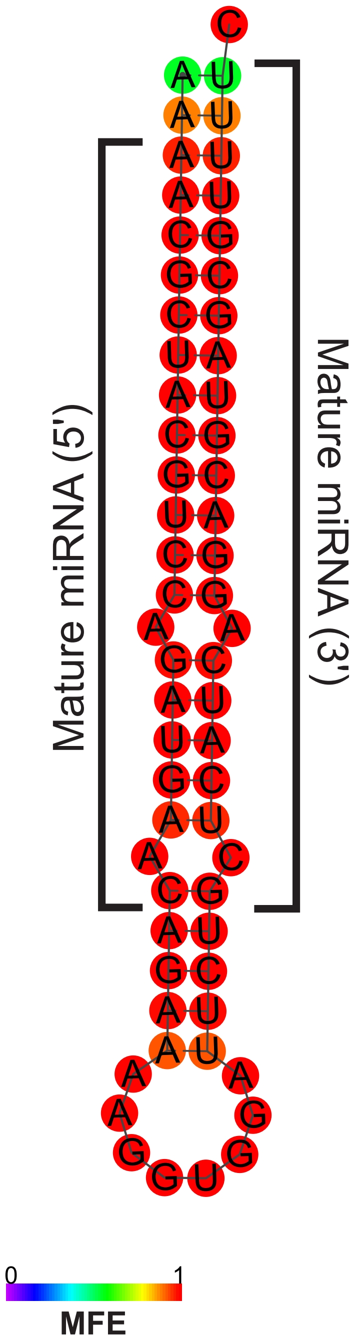

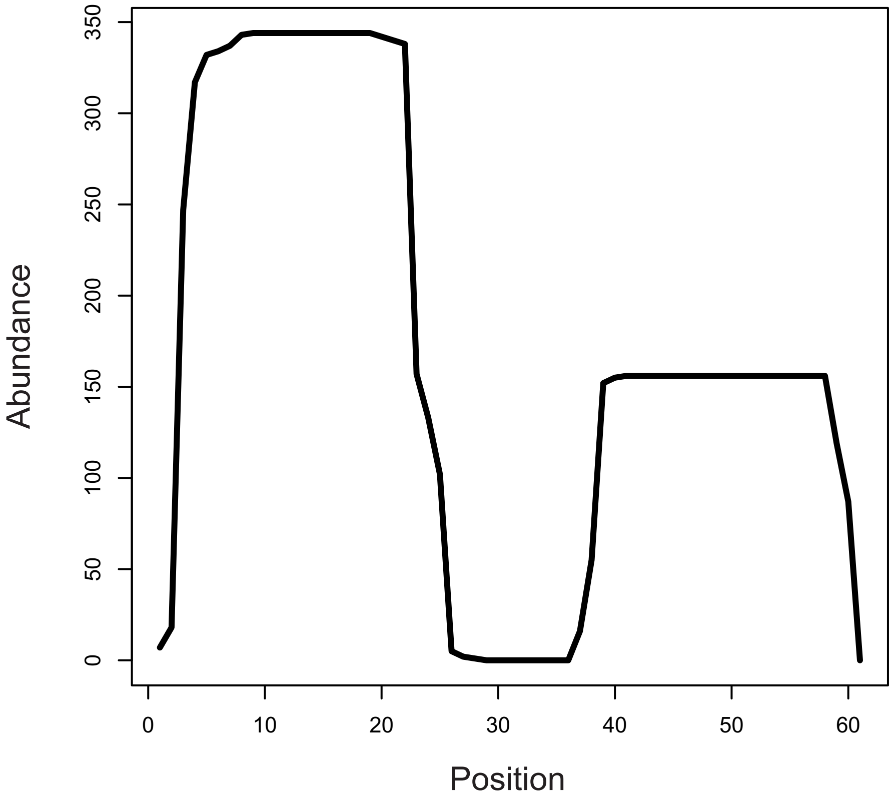


```
AAAACGCTACGTCCAGATGAACAGAAAAGGTGGATTCTGCTCATCAGGACGTAGCGTTTTC     Raw reads
..AACGCTACGTCCAGATGAAC.......................................     168
......................................GCTCATCAGGACGTAGCGTTTT.     83
...ACGCTACGTCCAGATGAACAGA....................................     64
..AACGCTACGTCCAGATGAACAG.....................................     26
..AACGCTACGTCCAGATGAACA......................................     22
.....................................TGCTCATCAGGACGTAGCGTTT..     21
.....................................TGCTCATCAGGACGTAGCGTT...     17
....................................CTGCTCATCAGGACGTAGCGTT...     16
....CGCTACGTCCAGATGAACAGA....................................     12
..AACGCTACGTCCAGATGAACAGA....................................     11
......................................GCTCATCAGGACGTAGCGTTT..     10
.AAACGCTACGTCCAGATGAAC.......................................     6
AAAACGCTACGTCCAGATGAAC.......................................     5
.......TACGTCCAGATGAACAGA....................................     5
......................................GCTCATCAGGACGTAGCGTT...     4
...ACGCTACGTCCAGATGAACAG.....................................     3
......CTACGTCCAGATGAACAGA....................................     3
.......................................CTCATCAGGACGTAGCGTTTT.     3
..AACGCTACGTCCAGATGAA........................................     2
...ACGCTACGTCCAGATGAACA......................................     2
.AAACGCTACGTCCAGATGAACAG.....................................     2
....CGCTACGTCCAGATGAACAGAA...................................     2
AAAACGCTACGTCCAGATG..........................................     1
.AAACGCTACGTCCAGATG..........................................     1
AAAACGCTACGTCCAGATGA.........................................     1
.AAACGCTACGTCCAGATGA.........................................     1
....CGCTACGTCCAGATGAAC.......................................     1
.....GCTACGTCCAGATGAAC.......................................     1
.AAACGCTACGTCCAGATGAACAGA....................................     1
.....GCTACGTCCAGATGAACAGA....................................     1
...ACGCTACGTCCAGATGAACAGAA...................................     1
.......TACGTCCAGATGAACAGAAA..................................     1
........ACGTCCAGATGAACAGAAAA.................................     1
........................................TCATCAGGACGTAGCGTTT..     1
.....................................TGCTCATCAGGACGTAGCGTTTT.     1

((((((((((((((.(((((.(((((........))))).))))).)))))))))))))).    (-34.20 MFE)
```

mir-n334

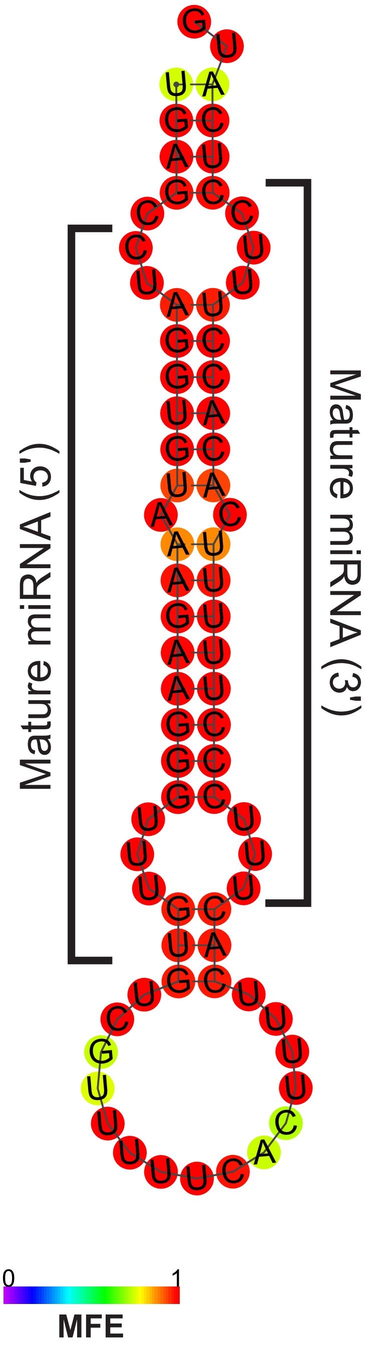

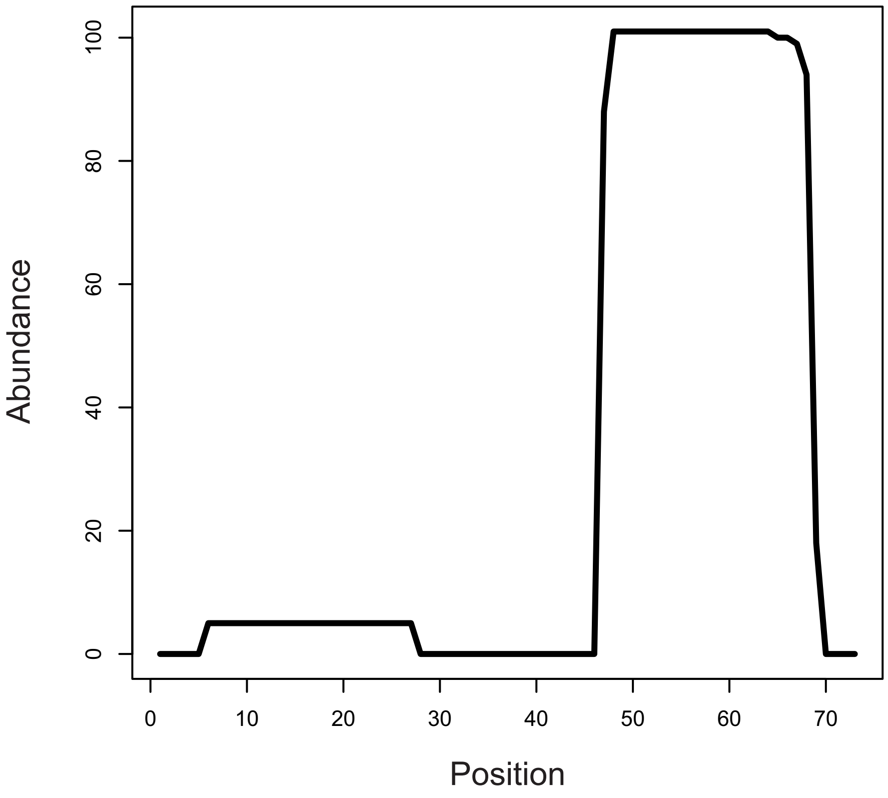


```
TGAGCCTAGGTGTAAAGAAGGGTTTGTGTCGTTTTTCACTTTTCACTTTCCCTTTTTCACACCTTTCCTCATG  Raw reads
..............................................TTTCCCTTTTTCACACCTTTCC.....  75
...............................................TTCCCTTTTTCACACCTTTCCT....  12
..............................................TTTCCCTTTTTCACACCTTTCCT....  6
.....CTAGGTGTAAAGAAGGGTTTGT..............................................  5
..............................................TTTCCCTTTTTCACACCTTTC......  5
..............................................TTTCCCTTTTTCACACCT.........  1
..............................................TTTCCCTTTTTCACACCTTT.......  1
...............................................TTCCCTTTTTCACACCTTTCC.....  1

((((...((((((.((((((((...(((...............)))...)))))))).))))))...)))).. (-25.56 MFE)
```

mir-n363-1

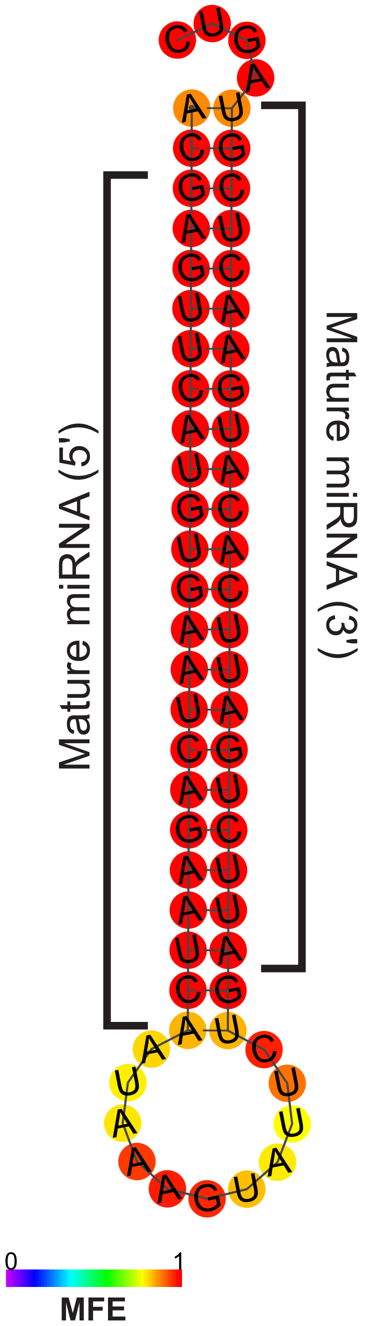

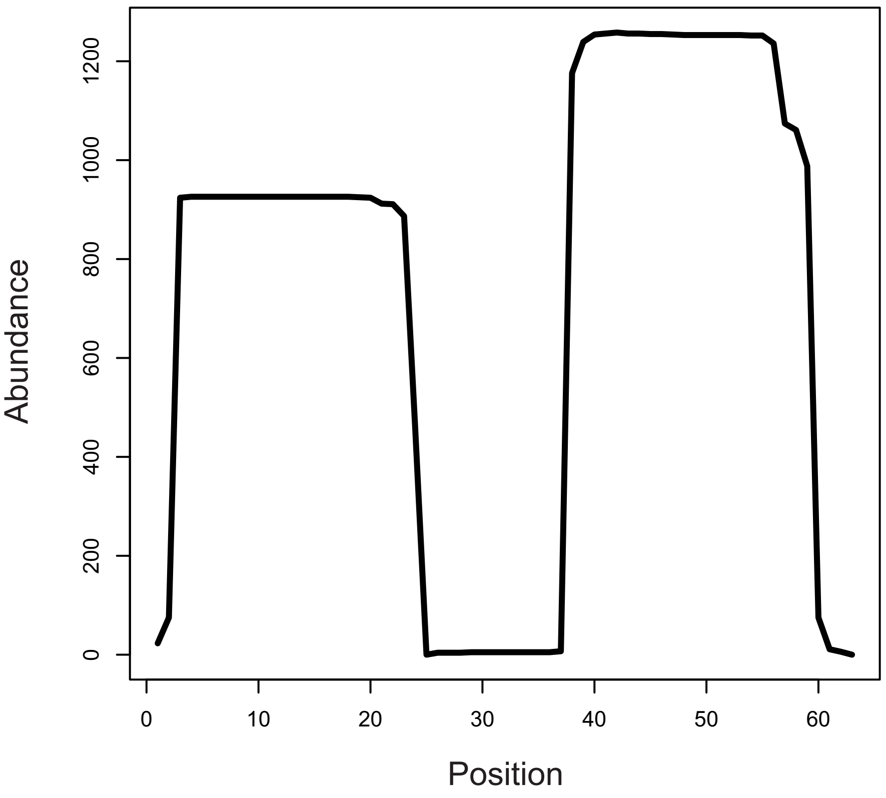


```
ACGAGTTCATGTGAATCAGAATCAATAAAGTATTCTGATTCTGATTCACATGAACTCGTAGTC   Raw reads
.....................................ATTCTGATTCACATGAACTCGT....   889
..GAGTTCATGTGAATCAGAATCA.......................................   452
..GAGTTCATGTGAATCAGAATC........................................   384
.....................................ATTCTGATTCACATGAACT.......   156
.....................................ATTCTGATTCACATGAACTCG.....   66
.CGAGTTCATGTGAATCAGAATC........................................   45
......................................TTCTGATTCACATGAACTCGTA...   32
.....................................ATTCTGATTCACATGAACTCGTA...   25
......................................TTCTGATTCACATGAACTCGT....   19
.....................................ATTCTGATTCACATGAAC........   16
..GAGTTCATGTGAATCAGAAT.........................................   13
ACGAGTTCATGTGAATCAGA...........................................   12
.....................................ATTCTGATTCACATGAACTC......   10
ACGAGTTCATGTGAATCAGAAT.........................................   9
.......................................TCTGATTCACATGAACTCGTA...   7
......................................TTCTGATTCACATGAACT.......   6
.....................................ATTCTGATTCACATGAACTCGTAGT.   6
.......................................TCTGATTCACATGAACTCGTAG..   5
......................................TTCTGATTCACATGAACTCG.....   4
.CGAGTTCATGTGAATCAGAAT.........................................   2
ACGAGTTCATGTGAATCAGAATC........................................   2
.CGAGTTCATGTGAATCAGAATCA.......................................   2
.........................TAAAGTATTCTGATTCT.....................   2
......................................TTCTGATTCACATGAACTC......   2
....................................GATTCTGATTCACATGAACTCG.....   2
.......................................TCTGATTCACATGAACTCGT....   2
........................................CTGATTCACATGAACTCGT....   2
.CGAGTTCATGTGAATCA.............................................   1
.CGAGTTCATGTGAATCAG............................................   1
.CGAGTTCATGTGAATCAGAA..........................................   1
...AGTTCATGTGAATCAGAATC........................................   1
...AGTTCATGTGAATCAGAATCA.......................................   1
............................AGTATTCTGATTCTGA...................   1
.........................TAAAGTATTCTGATTCTGATT.................   1
.........................TAAAGTATTCTGATTCTGATTC................   1
.....................................ATTCTGATTCACATGA..........   1
.......................................TCTGATTCACATGAACTC......   1
.........................................TGATTCACATGAACTCG.....   1
.........................................TGATTCACATGAACTCGT....   1

((((((((((((((((((((((((...........)))))))))))))))))))))))).... (-38.20 MFE)
```

mir-n363-2

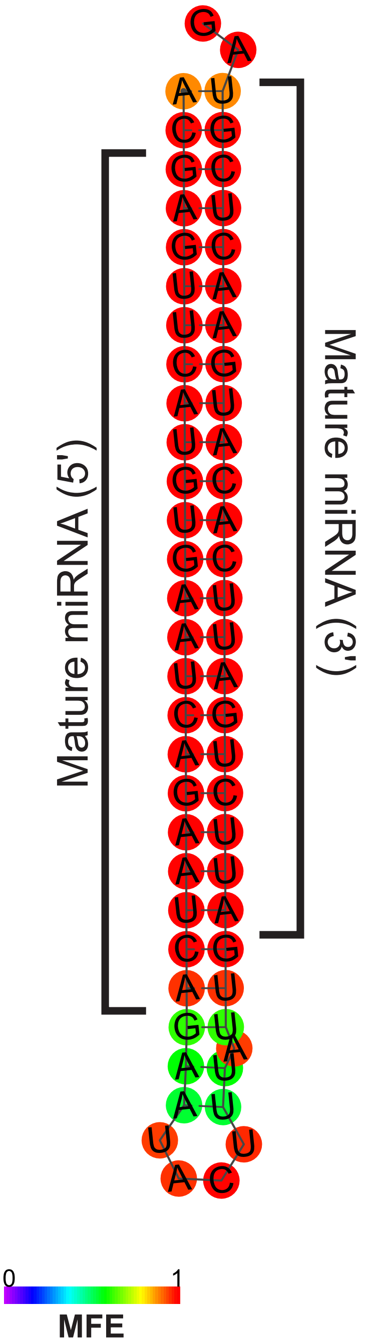

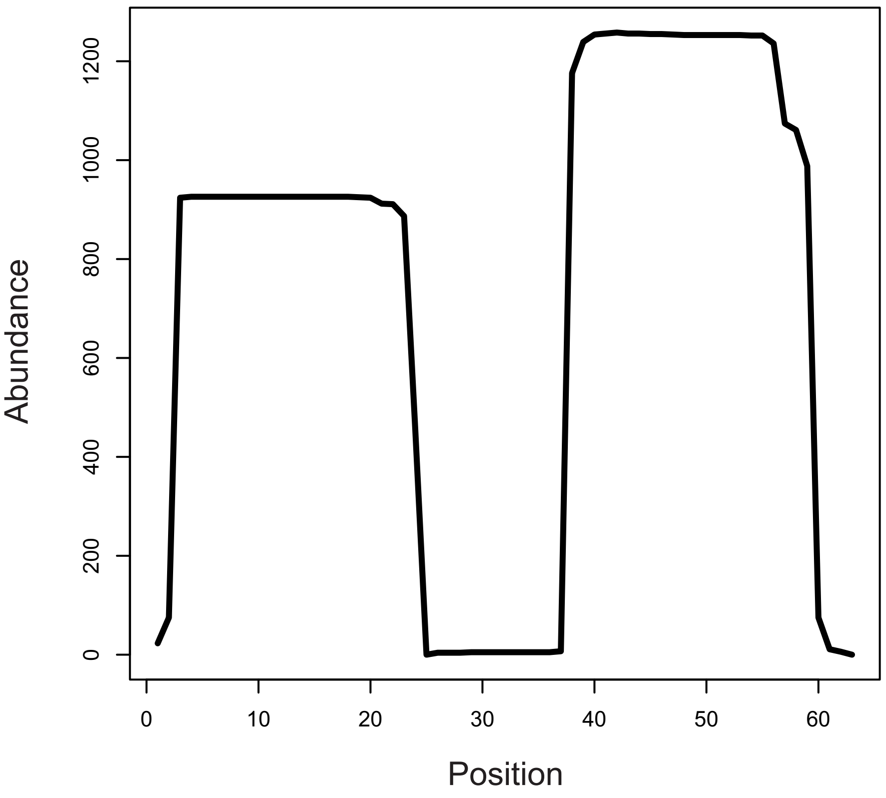


```
ACGAGTTCATGTGAATCAGAATCAGAATACTTTATTGATTCTGATTCACATGAACTCGTAG     Raw reads
.....................................ATTCTGATTCACATGAACTCGT..     889
..GAGTTCATGTGAATCAGAATCA.....................................     452
..GAGTTCATGTGAATCAGAATC......................................     384
.....................................ATTCTGATTCACATGAACT.....     156
.....................................ATTCTGATTCACATGAACTCG...     66
.CGAGTTCATGTGAATCAGAATC......................................     45
......................................TTCTGATTCACATGAACTCGTA.     32
.....................................ATTCTGATTCACATGAACTCGTA.     25
......................................TTCTGATTCACATGAACTCGT..     19
.....................................ATTCTGATTCACATGAAC......     16
..GAGTTCATGTGAATCAGAAT.......................................     13
ACGAGTTCATGTGAATCAGA.........................................     12
.....................................ATTCTGATTCACATGAACTC....     10
ACGAGTTCATGTGAATCAGAAT.......................................     9
.......................................TCTGATTCACATGAACTCGTA.     7
......................................TTCTGATTCACATGAACT.....     6
.......................................TCTGATTCACATGAACTCGTAG     5
......................................TTCTGATTCACATGAACTCG...     4
.CGAGTTCATGTGAATCAGAAT.......................................     2
ACGAGTTCATGTGAATCAGAATC......................................     2
.CGAGTTCATGTGAATCAGAATCA.....................................     2
..................................TTGATTCTGATTCACATGAACT.....     2
......................................TTCTGATTCACATGAACTC....     2
....................................GATTCTGATTCACATGAACTCG...     2
.......................................TCTGATTCACATGAACTCGT..     2
........................................CTGATTCACATGAACTCGT..     2
.CGAGTTCATGTGAATCA...........................................     1
.CGAGTTCATGTGAATCAG..........................................     1
.CGAGTTCATGTGAATCAGAA........................................     1
...AGTTCATGTGAATCAGAATC......................................     1
...AGTTCATGTGAATCAGAATCA.....................................     1
........ATGTGAATCAGAATCAGAATACT..............................     1
...............TCAGAATCAGAATACTTTA...........................     1
.....................................ATTCTGATTCACATGA........     1
.................................ATTGATTCTGATTCACATGAAC......     1
.......................................TCTGATTCACATGAACTC....     1
.........................................TGATTCACATGAACTCG...     1
.........................................TGATTCACATGAACTCGT..     1

(((((((((((((((((((((((((((....)).))))))))))))))))))))))))).. (-38.80 MFE)
```

mir-n370

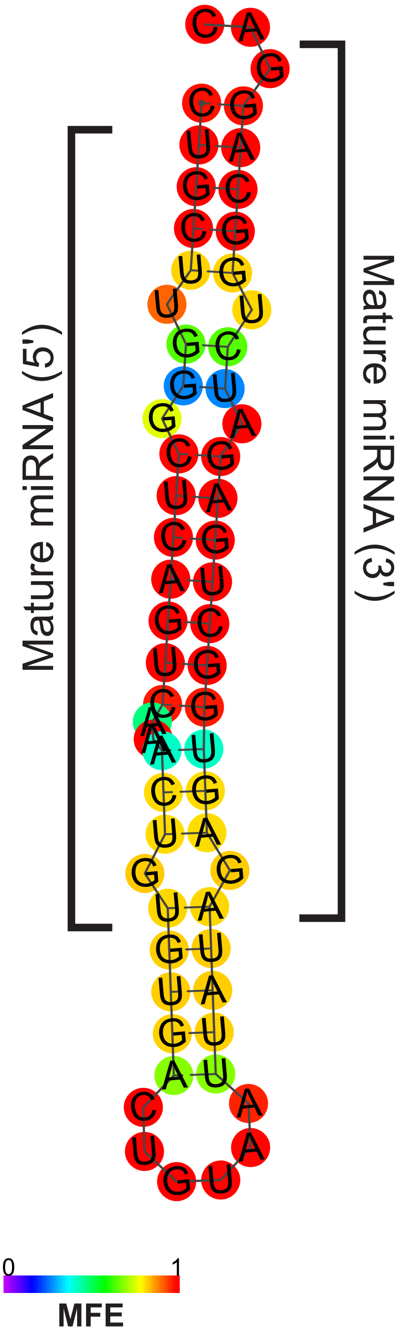

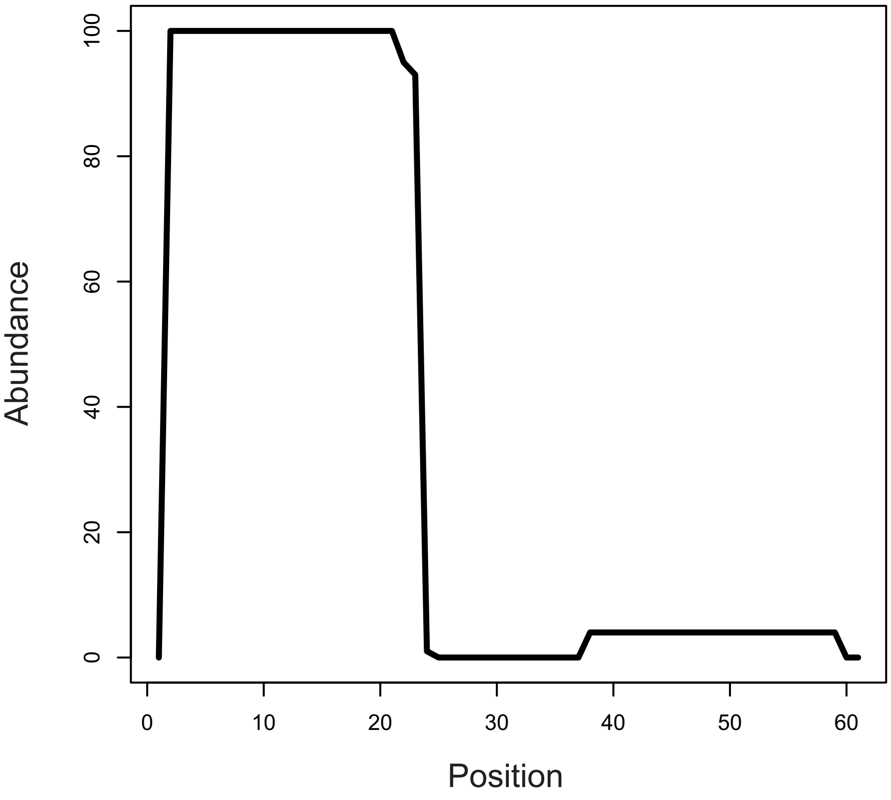


```
CTGCTTGGGCTCAGTCAAACTGTGTGACTGTAATTATAGAGTGGCTGAGATCTGGCAGGAC    Raw reads
.TGCTTGGGCTCAGTCAAACTGT......................................    92
.TGCTTGGGCTCAGTCAAACT........................................    5
.....................................AGAGTGGCTGAGATCTGGCAGG..    4
.TGCTTGGGCTCAGTCAAACTG.......................................    2
.TGCTTGGGCTCAGTCAAACTGTG.....................................    1

(((((.(..((((((((..(((((..........)))))..))))))))..).)))))...    (-24.80 MFE)
```

mir-n378

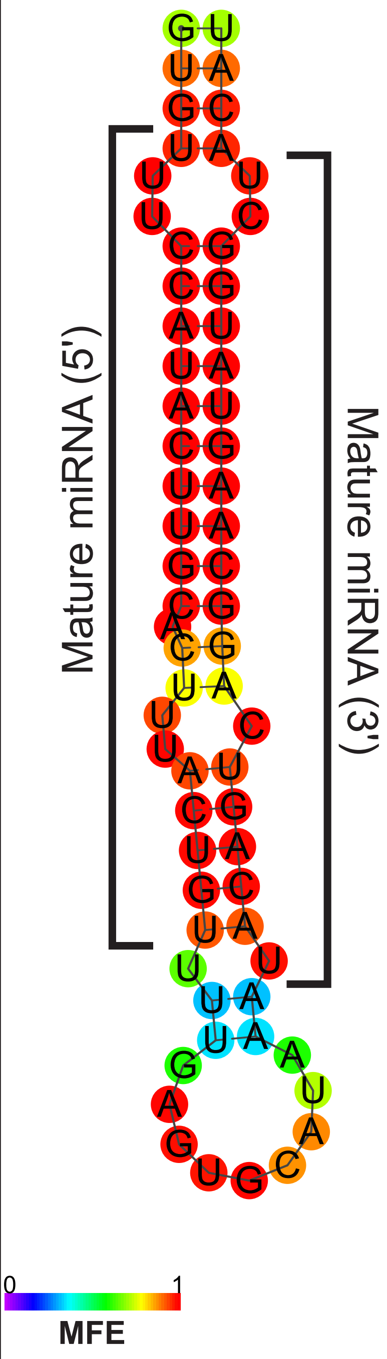

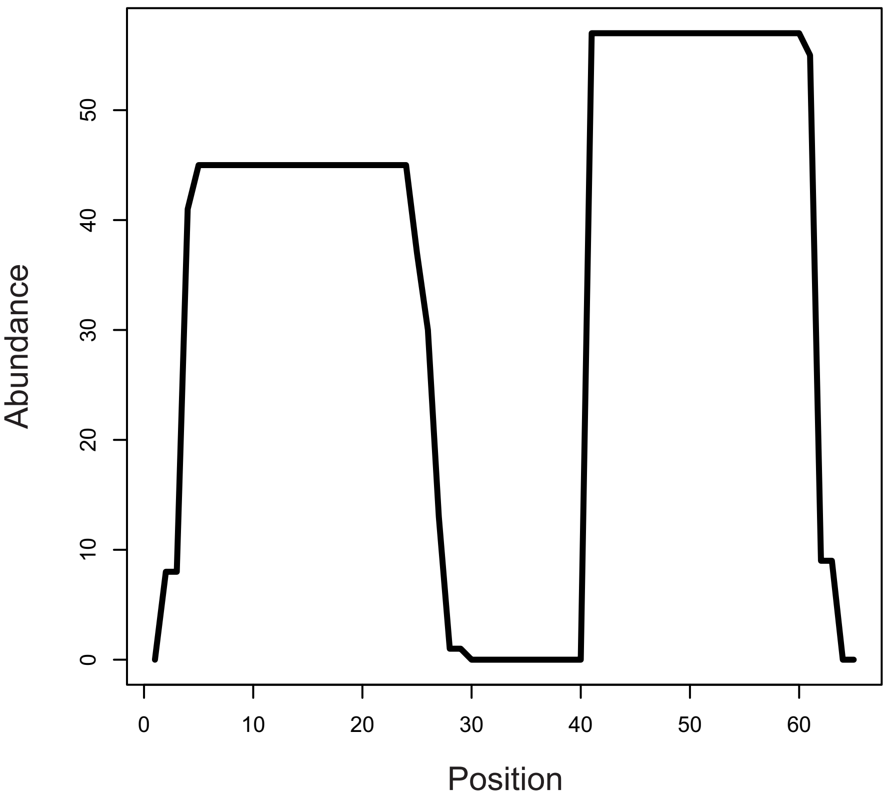


```
GTGTTTCCATACTTGCACTTTACTGTTTTGAGTGCATAAATACAGTCAGGCAAGTATGGCTACAT   Raw reads
........................................TACAGTCAGGCAAGTATGGCT....   46
...TTTCCATACTTGCACTTTACTGT.......................................   12
...TTTCCATACTTGCACTTTACTGTT......................................   10
........................................TACAGTCAGGCAAGTATGGCTAC..   9
...TTTCCATACTTGCACTTTACTG........................................   7
.TGTTTCCATACTTGCACTTTACT.........................................   5
...TTTCCATACTTGCACTTTACT.........................................   3
....TTCCATACTTGCACTTTACTGT.......................................   3
.TGTTTCCATACTTGCACTTTACTGT.......................................   2
........................................TACAGTCAGGCAAGTATGGC.....   2
.TGTTTCCATACTTGCACTTTACTGTT......................................   1
....TTCCATACTTGCACTTTACTGTT......................................   1
...TTTCCATACTTGCACTTTACTGTTTT....................................   1

((((..((((((((((.((..(((((.((.........)).))))).))))))))))))..))))   (-23.50 MFE)
```

mir-n416

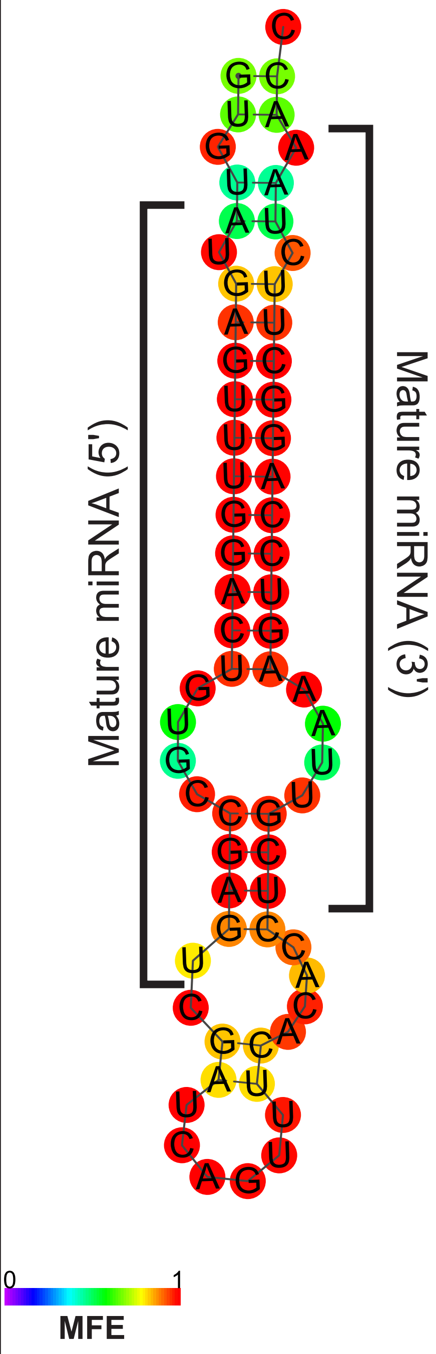

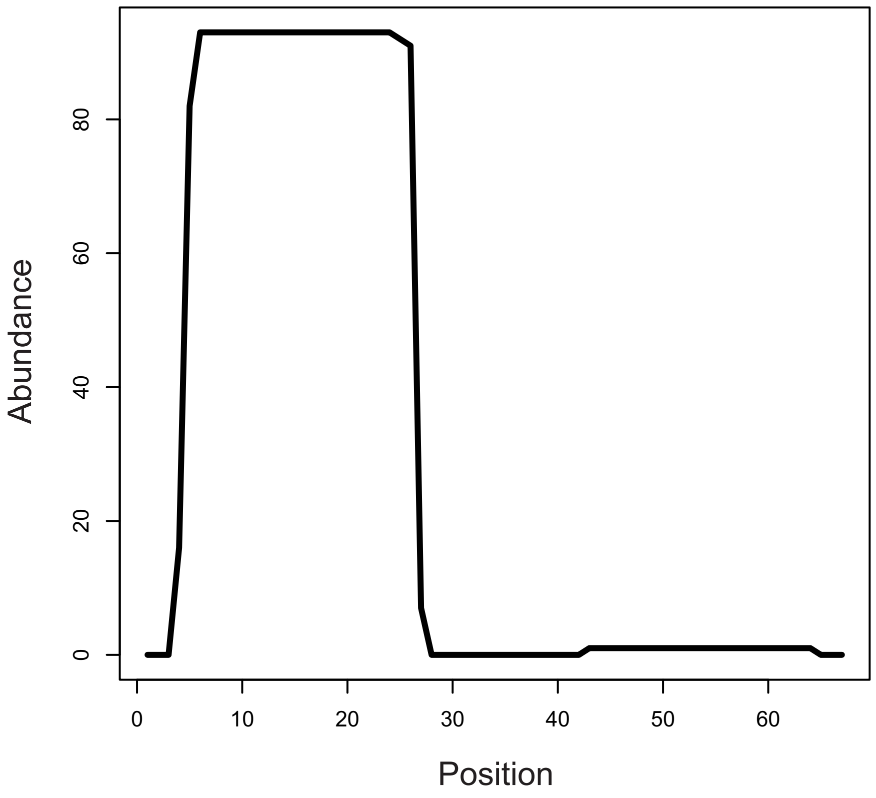


```
GTGTATGAGTTTGGACTGTGCCGAGTCGATCAGTTTCACACCTCGTTAAAGTCCAGGCTTCTAAACC  Raw reads
....ATGAGTTTGGACTGTGCCGAGT.........................................  64
...TATGAGTTTGGACTGTGCCGAGT.........................................  16
.....TGAGTTTGGACTGTGCCGAGTC........................................  7
.....TGAGTTTGGACTGTGCCGAGT.........................................  4
....ATGAGTTTGGACTGTGCCGA...........................................  1
....ATGAGTTTGGACTGTGCCGAG..........................................  1
..........................................TCGTTAAAGTCCAGGCTTCTAA...  1

((.((.(((((((((((.((.((((..((......))....)))).)).))))))))))).)).)).  (-20.20 MFE)
```

mir-n421

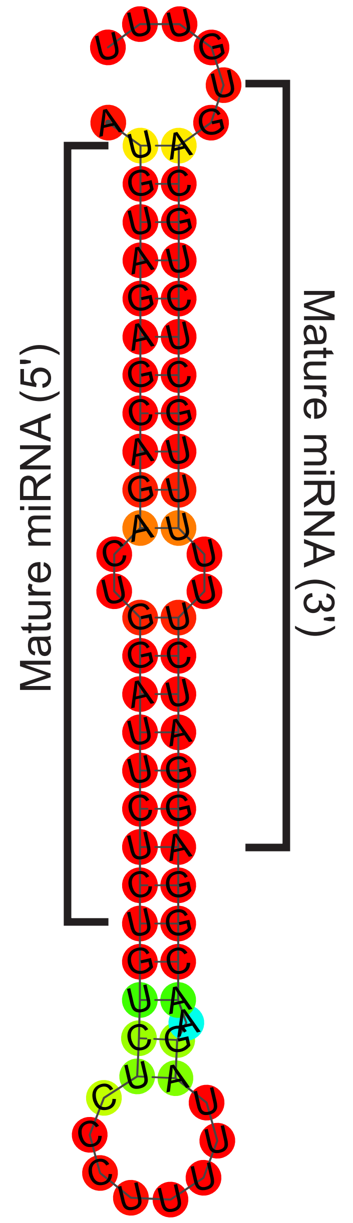

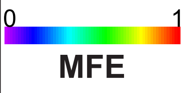

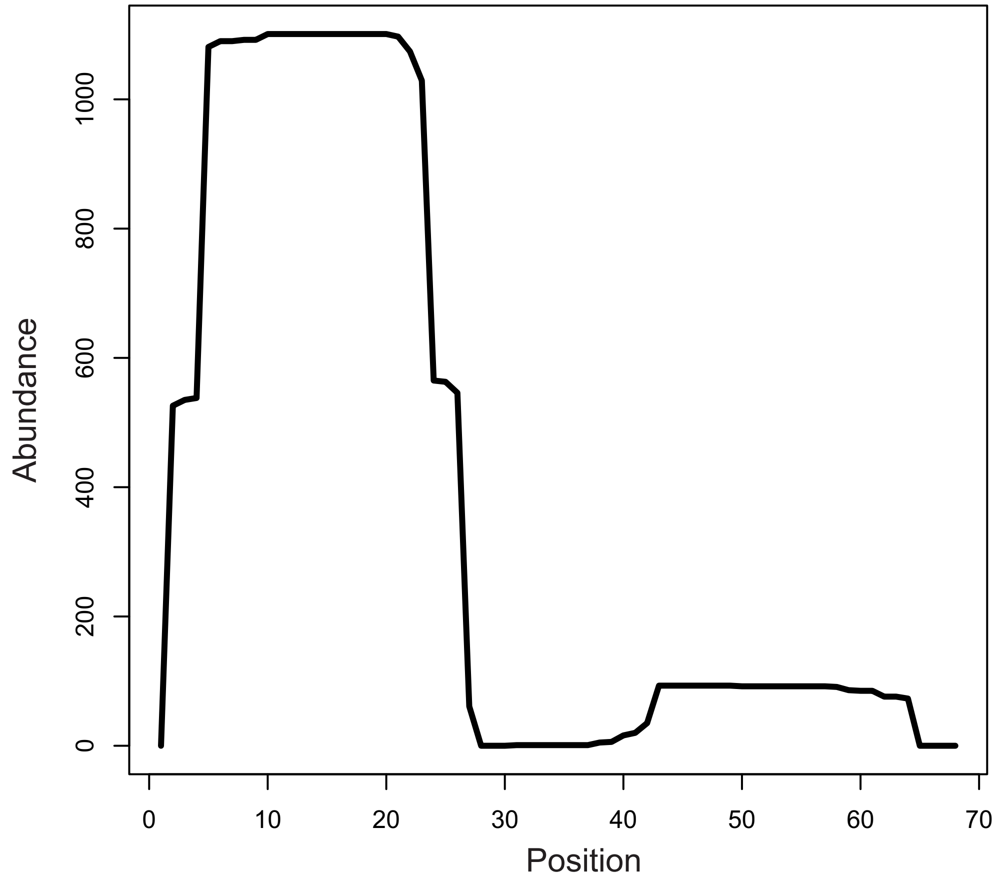


```
ATGTAGAGCAGACTGGATTCTCTGTCTCCCTTTTTAGAACGGAGGATCTTTTTTGCTCTGCAGTGTTT     Raw reads
.TGTAGAGCAGACTGGATTCTCT.............................................     452
....AGAGCAGACTGGATTCTCTGTC..........................................     468
....AGAGCAGACTGGATTCTCTGTCT.........................................     58
..........................................AGGATCTTTTTTGCTCTGCAGT....     55
.TGTAGAGCAGACTGGATTCTC..............................................     42
.TGTAGAGCAGACTGGATTCT...............................................     23
.........................................GAGGATCTTTTTTGCTCTGCAGT....     15
....AGAGCAGACTGGATTCTCTGT...........................................     11
..GTAGAGCAGACTGGATTCTCT.............................................     9
.....GAGCAGACTGGATTCTCTGTC..........................................     9
.........AGACTGGATTCTCTGTC..........................................     8
.......................................CGGAGGATCTTTTTTGCTCTGC.......     8
.TGTAGAGCAGACTGGATTC................................................     4
.TGTAGAGCAGACTGGATTCTCTGT...........................................     4
....AGAGCAGACTGGATTCTCT.............................................     3
..........................................AGGATCTTTTTTGCTCTGCAG.....     3
........................................GGAGGATCTTTTTTGCTCTGCAGT....     3
....AGAGCAGACTGGATTCTC..............................................     2
...TAGAGCAGACTGGATTCTCTGT...........................................     2
.......GCAGACTGGATTCTCTGTCT.........................................     2
.....................................AACGGAGGATCTTTTTTGCTC..........     2
.......................................CGGAGGATCTTTTTTGCTC..........     2
...TAGAGCAGACTGGATTCTC..............................................     1
.TGTAGAGCAGACTGGATTCTCTG............................................     1
....AGAGCAGACTGGATTCTCTG............................................     1
.........AGACTGGATTCTCTGTCT.........................................     1
..............................TTTTTAGAACGGAGGATCT...................     1
.....................................AACGGAGGATCTTTTTTGCT...........     1
......................................ACGGAGGATCTTTTTTGCTC..........     1
.....................................AACGGAGGATCTTTTTTGCTCT.........     1
........................................GGAGGATCTTTTTTGCTCTGC.......     1

.(((((((((((..(((((((((((((........)).)))))))))))..)))))))))))......   (-32.30 MFE)
```

mir-n437

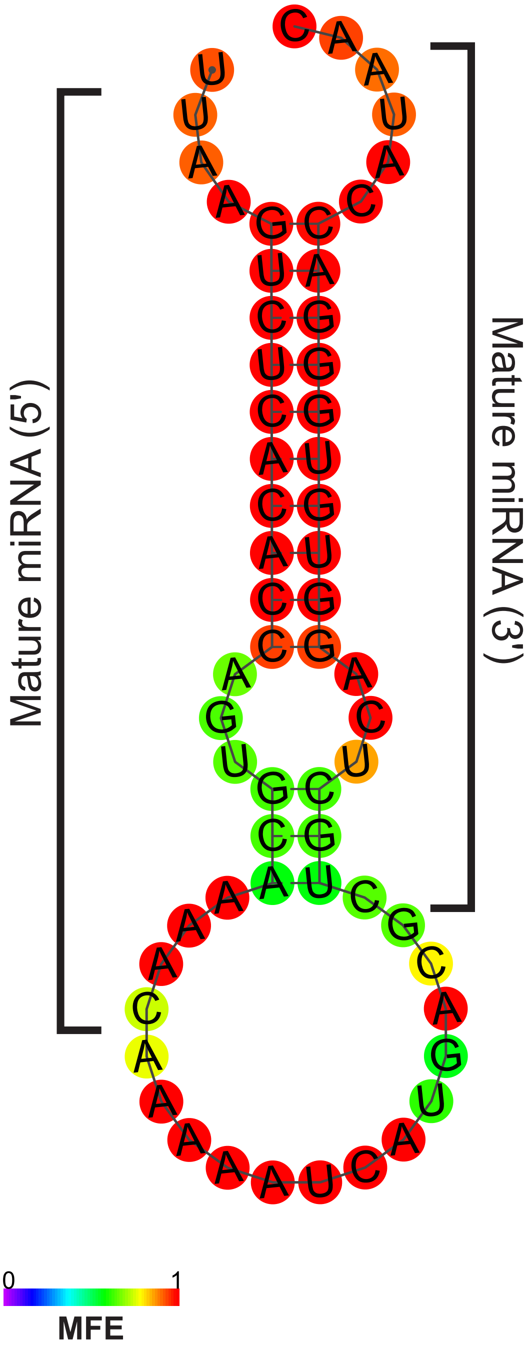

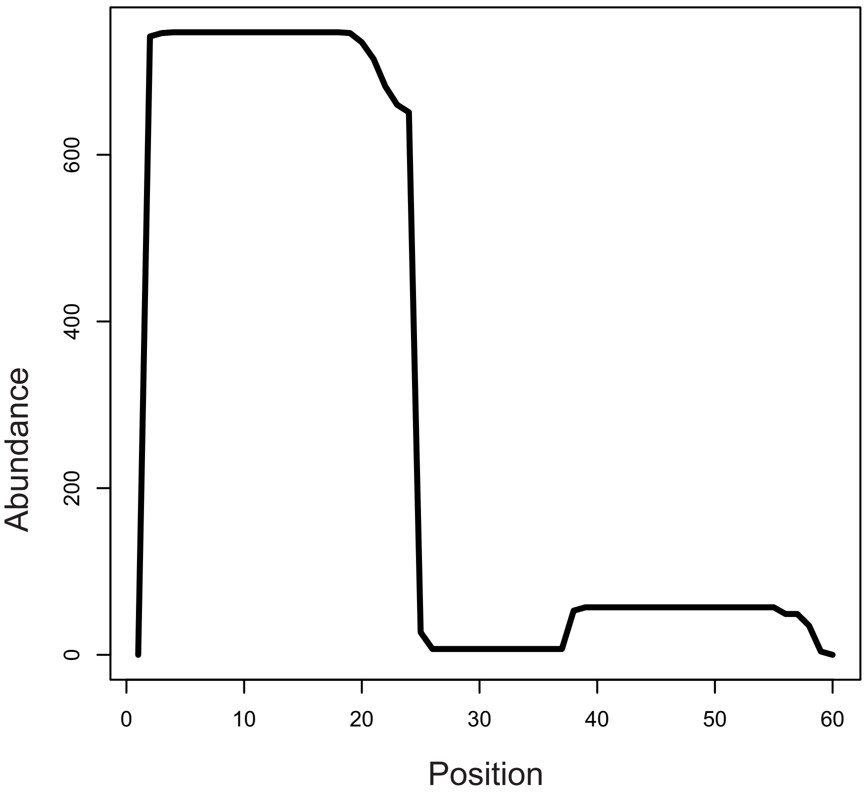


```
TTAAGTCTCACACCAGTGCAAAACAAAAATCATGACGCTGCTCAGGTGTGGGACCATAAC      Raw reads
.TAAGTCTCACACCAGTGCAAAAC....................................      626
.TAAGTCTCACACCAGTGCAA.......................................      33
.TAAGTCTCACACCAGTGCAAA......................................      22
.....................................CTGCTCAGGTGTGGGACCATA..      22
.TAAGTCTCACACCAGTGCA........................................      20
.TAAGTCTCACACCAGTGCAAAACA...................................      20
.....................................CTGCTCAGGTGTGGGACCAT...      12
.TAAGTCTCACACCAGTGC.........................................      11
.TAAGTCTCACACCAGTGCAAAA.....................................      9
.....................................CTGCTCAGGTGTGGGACC.....      8
........................AAAAATCATGACGCTGCTCAGGTGTGGGACCATA..      7
..AAGTCTCACACCAGTGCAAAAC....................................      4
.....................................CTGCTCAGGTGTGGGACCATAA.      4
......................................TGCTCAGGTGTGGGACCAT...      2
......................................TGCTCAGGTGTGGGACCATA..      2
.TAAGTCTCACACCAGTG..........................................      1
...AGTCTCACACCAGTGCAAAAC....................................      1

....((((((((((.(.(((..................))).).))))))))))......     (-19.17 MFE)
```

mir-n456

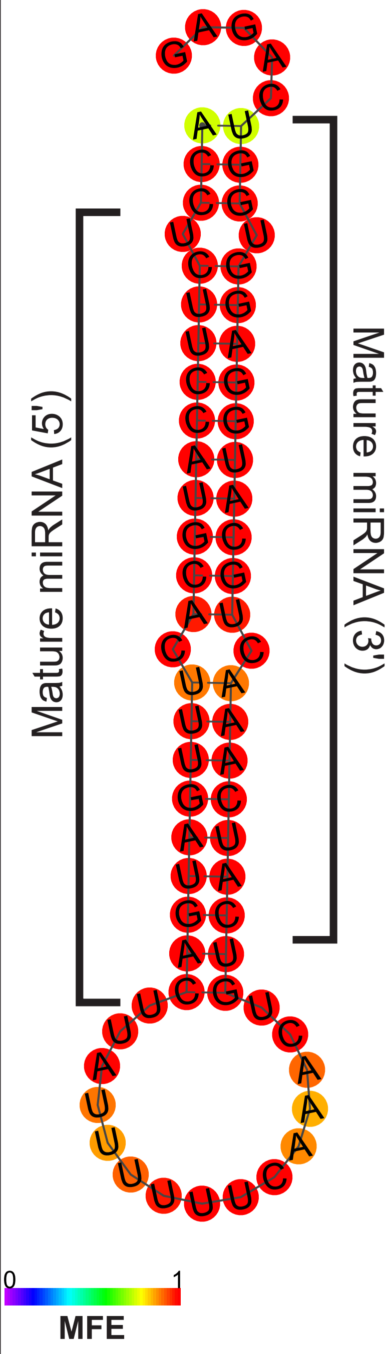

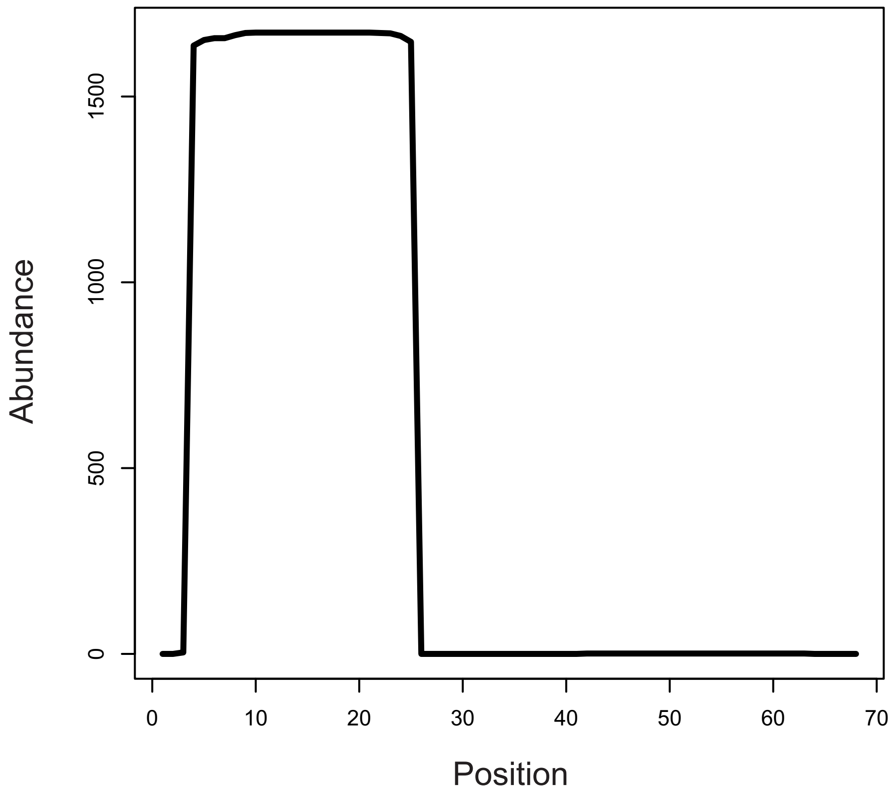


```
ACCTCTTCCATGCACTTTGATGACTTATTTTTTCAAACTGTCATCAAACTGCATGGAGGTGGTCAGAG Raw reads
...TCTTCCATGCACTTTGATGACT........................................... 1610
...TCTTCCATGCACTTTGATGAC............................................ 16
....CTTCCATGCACTTTGATGACT........................................... 15
.......CCATGCACTTTGATGACT........................................... 8
...TCTTCCATGCACTTTGATGA............................................. 6
........CATGCACTTTGATGACT........................................... 6
.....TTCCATGCACTTTGATGACT........................................... 5
..CTCTTCCATGCACTTTGATGACT........................................... 2
..CTCTTCCATGCACTTTGAT............................................... 1
...TCTTCCATGCACTTTGATG.............................................. 1
..CTCTTCCATGCACTTTGATGA............................................. 1
.........ATGCACTTTGATGACT........................................... 1
.........................................CATCAAACTGCATGGAGGTGGT..... 1

(((.((((((((((.(((((((((...............))))))))).)))))))))).))).....  (-32.06 MFE)
```

mir-n470a

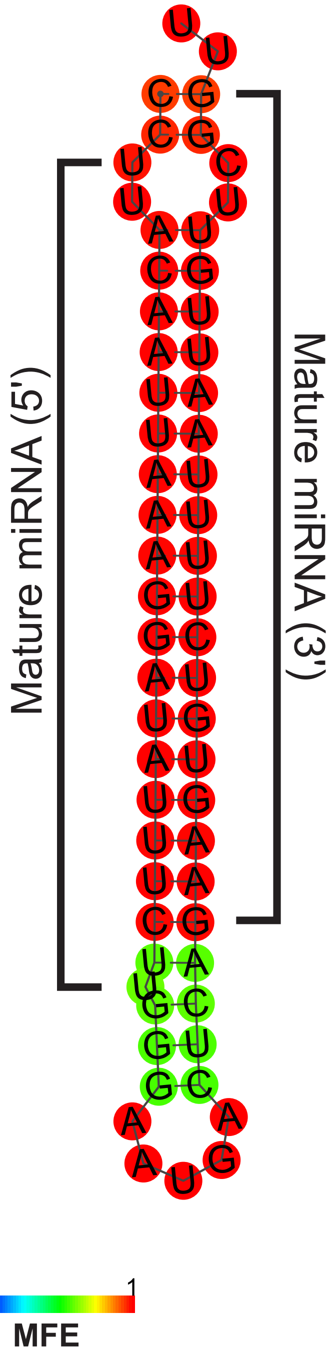

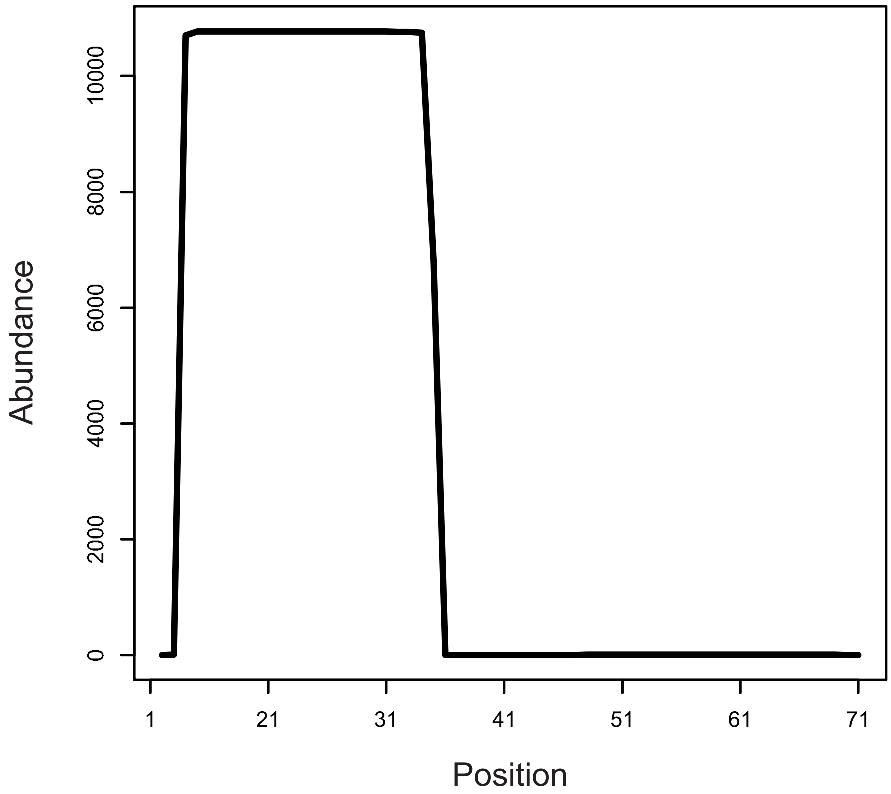


```
CCTTACAATTAAAGGATATTTCTTGGGAATGACTCAGAAGTGTCTTTTAATTGTTCGGTT    Raw reads
..TTACAATTAAAGGATATTTCTT....................................    6741
..TTACAATTAAAGGATATTTCT.....................................    3926
...TACAATTAAAGGATATTTCTT....................................    39
...TACAATTAAAGGATATTTCT.....................................    27
..TTACAATTAAAGGATATTTC......................................    10
.CTTACAATTAAAGGATATTTCT.....................................    8
..TTACAATTAAAGGATATT........................................    7
....................................GAAGTGTCTTTTAATTGTTCGG..    7
..TTACAATTAAAGGATATTT.......................................    3
.CTTACAATTAAAGGATATTTCTT....................................    3
.......ATTAAAGGATATTTCTT....................................    2
..TTACAATTAAAGGATAT.........................................    1
...TACAATTAAAGGATATTTC......................................    1
....ACAATTAAAGGATATTTCT.....................................    1
....ACAATTAAAGGATATTTCTT....................................    1
..TTACAATTAAAGGATATTTCTTG...................................    1
.....................................AAGTGTCTTTTAATTGTTCGGT.    1
......................................AGTGTCTTTTAATTGTTCGGT.    1

((..(((((((((((((((((((.(((.....))))))))))))))))))))))..))..    (-25.30 MFE)
```

mir-n470b

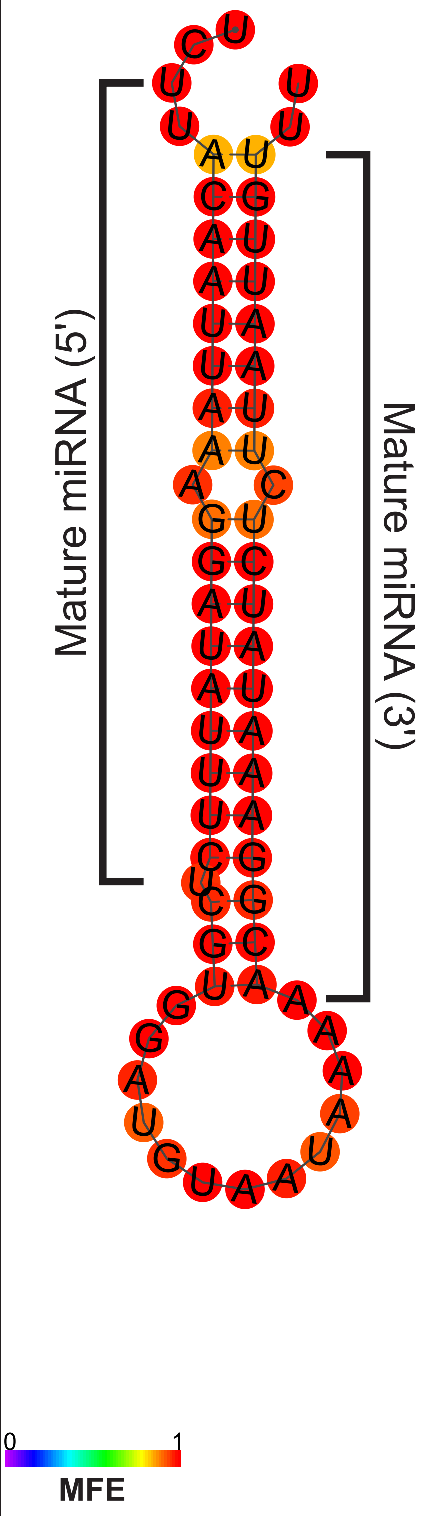

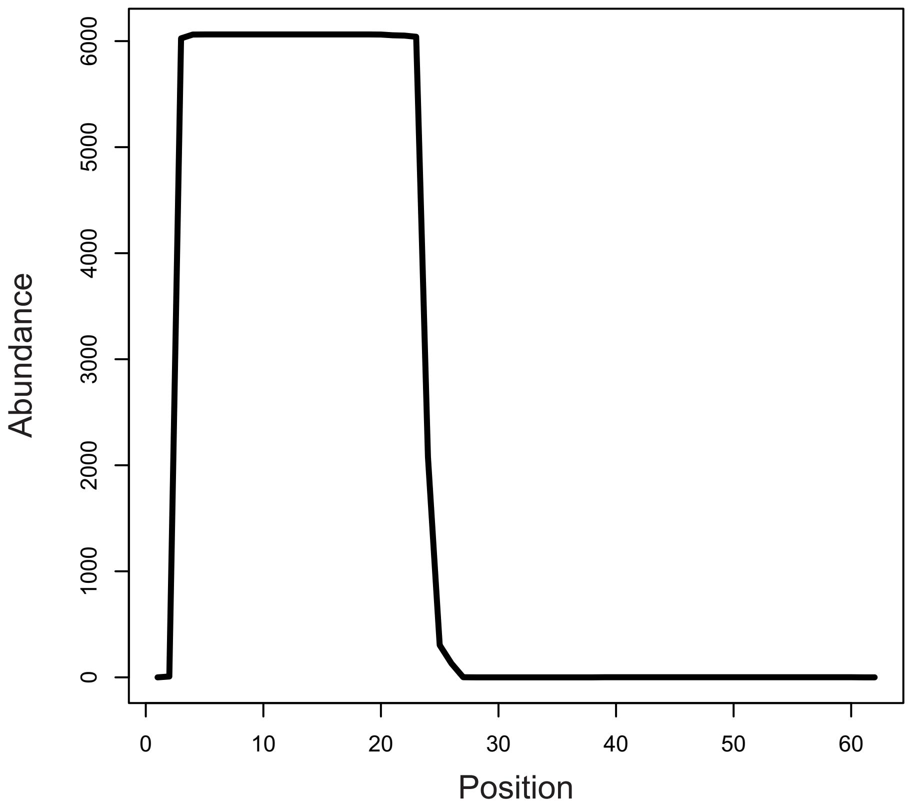


```
TCTTACAATTAAAGGATATTTCTCGTGGATGTAATAAAAACGGAAATATCTCTTAATTGTTT    Raw reads
..TTACAATTAAAGGATATTTCT.......................................    3926
..TTACAATTAAAGGATATTTCTC......................................    1769
..TTACAATTAAAGGATATTTCTCG.....................................    172
..TTACAATTAAAGGATATTTCTCGT....................................    129
...TACAATTAAAGGATATTTCT.......................................    27
..TTACAATTAAAGGATATTTC........................................    10
.CTTACAATTAAAGGATATTTCT.......................................    8
..TTACAATTAAAGGATATT..........................................    7
...TACAATTAAAGGATATTTCTC......................................    6
..TTACAATTAAAGGATATTT.........................................    3
..TTACAATTAAAGGATAT...........................................    1
...TACAATTAAAGGATATTTC........................................    1
....ACAATTAAAGGATATTTCT.......................................    1
...TACAATTAAAGGATATTTCTCG.....................................    1
...TACAATTAAAGGATATTTCTCGT....................................    1
..TTACAATTAAAGGATATTTCTCGTG...................................    1
......................................AACGGAAATATCTCTTAATTGT..    1

....((((((((..((((((((.(((.............)))))))))))..)))))))).. (-18.82 MFE)
```

mir-n482

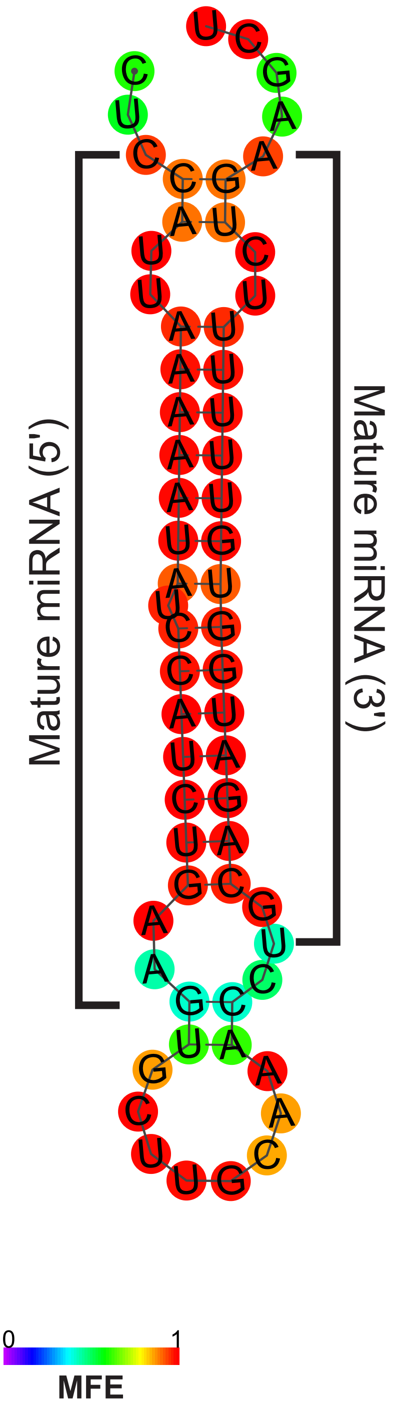

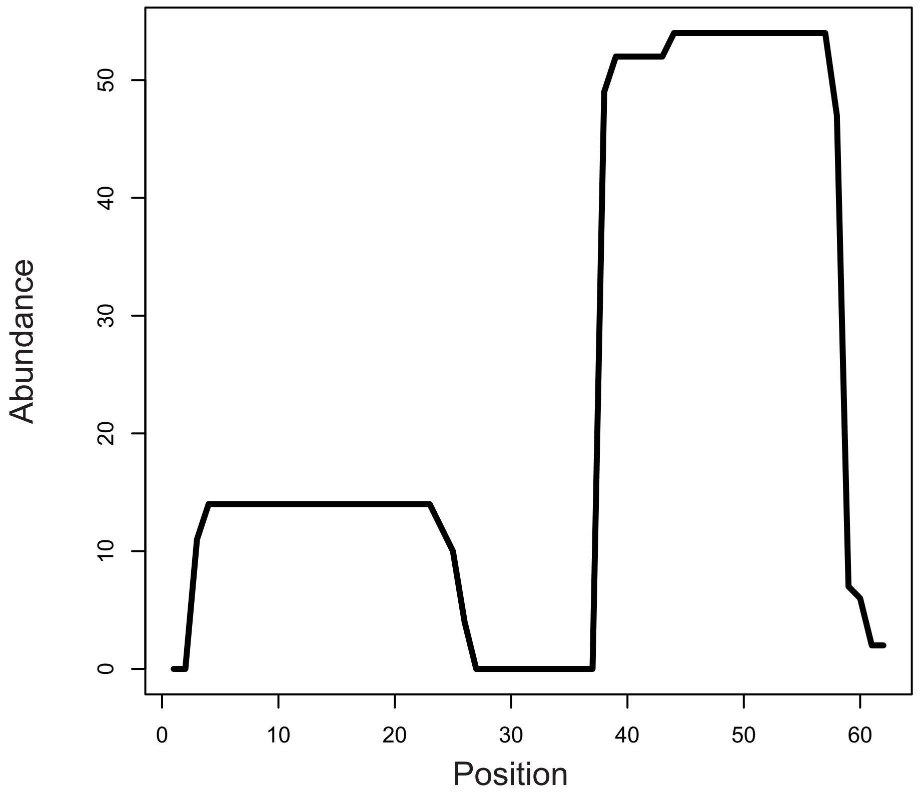


```
CTCCATTAAAAATATCCATCTGAAGTGCTTGCAAACCTGCAGATGGTGTTTTTTCTGAAGCT    Raw reads
.....................................TGCAGATGGTGTTTTTTCTGA....    40
.....................................TGCAGATGGTGTTTTTTCTG.....    7
..CCATTAAAAATATCCATCTGAAG.....................................    4
..CCATTAAAAATATCCATCTGAAGT....................................    3
......................................GCAGATGGTGTTTTTTCTGAAG..    3
..CCATTAAAAATATCCATCTGA.......................................    2
..CCATTAAAAATATCCATCTGAA......................................    2
...CATTAAAAATATCCATCTGAAG.....................................    2
...........................................TGGTGTTTTTTCTGAAGCT    2
...CATTAAAAATATCCATCTGAAGT....................................    1
.....................................TGCAGATGGTGTTTTTTCTGAA...    1
.....................................TGCAGATGGTGTTTTTTCTGAAG..    1
...((..(((((((.(((((((..((........))...))))))))))))))..))..... (-16.20 MFE)
```

mir-n483

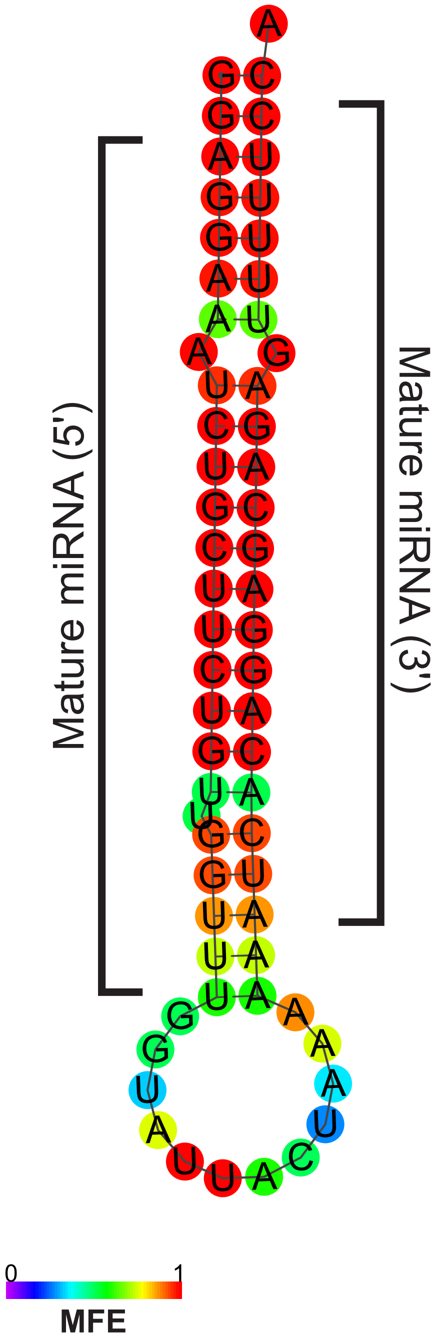

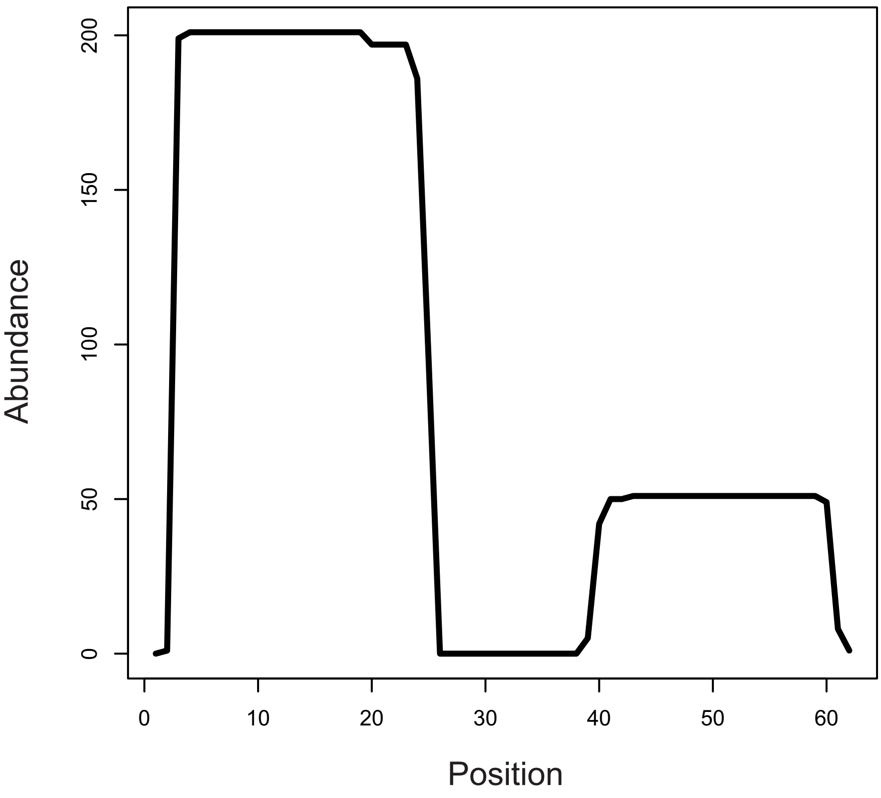


```
GGAGGAAATCTGCTTCTGTTGGTTTGGTATTACTAAAAAATCACAGGAGCAGAGTTTTTCCA   Raw reads
..AGGAAATCTGCTTCTGTTGGTTT.....................................   94
..AGGAAATCTGCTTCTGTTGGTT......................................   90
.......................................ATCACAGGAGCAGAGTTTTTC..   32
..AGGAAATCTGCTTCTGTTGGT.......................................   10
......................................AATCACAGGAGCAGAGTTTTTC..   5
..AGGAAATCTGCTTCTGT............................................   4
.......................................ATCACAGGAGCAGAGTTTTTCC.   4
........................................TCACAGGAGCAGAGTTTTTC..   3
........................................TCACAGGAGCAGAGTTTTTCC.   3
.GAGGAAATCTGCTTCTGTTGGT.......................................   1
...GGAAATCTGCTTCTGTTGGTT......................................   1
...GGAAATCTGCTTCTGTTGGTTT.....................................   1
.......................................ATCACAGGAGCAGAGTTTTT...   1
........................................TCACAGGAGCAGAGTTTTT...   1
..........................................ACAGGAGCAGAGTTTTTC..   1
........................................TCACAGGAGCAGAGTTTTTCCA   1

(((((((.(((((((((((.(((((..((....))..)))))))))))))))).))))))).   (-28.80 MFE)
```

mir-n495

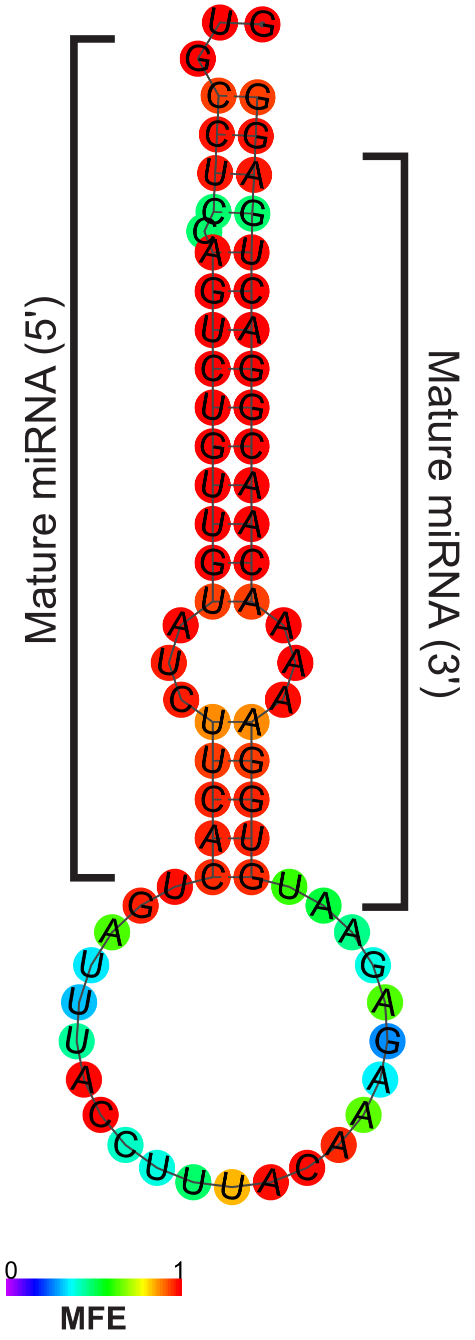

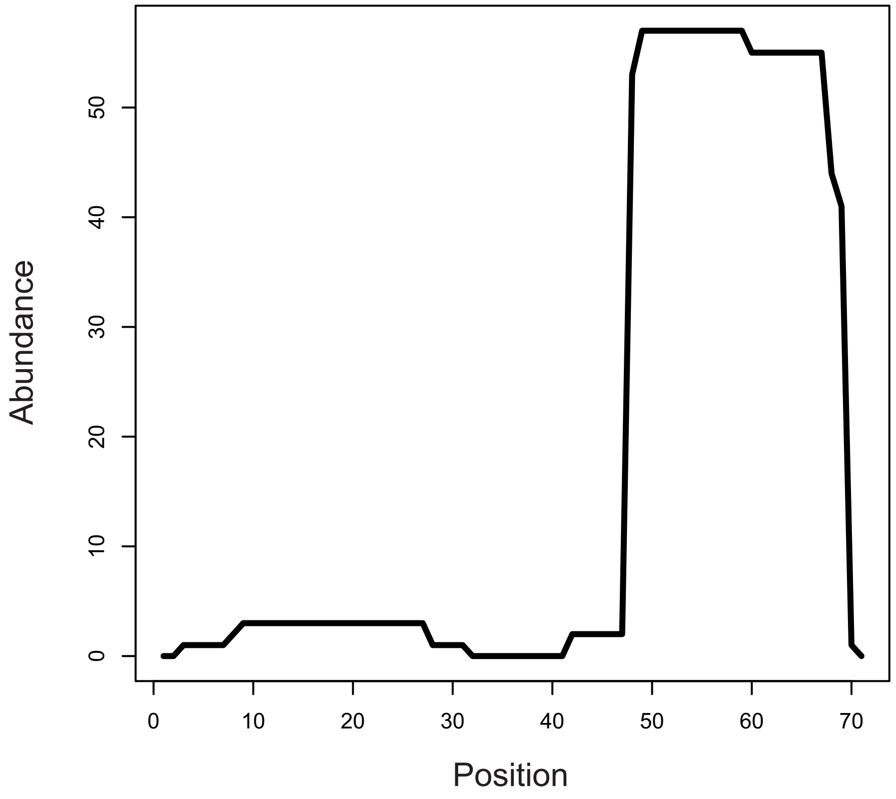


```
GTGCCTCCAGTCTGTTGTATCTTCACTGATTTACCTTTACAAAGAGAATGTGGAAAAACAACGGACTGAGG    Raw reads
...............................................ATGTGGAAAAACAACGGACTGA..    37
...............................................ATGTGGAAAAACAACGGACT....    11
...............................................ATGTGGAAAAACAACGGACTG...    3
................................................TGTGGAAAAACAACGGACTGA..    3
.........................................AAGAGAATGTGGAAAAAC............    2
..GCCTCCAGTCTGTTGTATCTTCACT............................................    1
.......CAGTCTGTTGTATCTTCACT............................................    1
........AGTCTGTTGTATCTTCACTGATT........................................    1
................................................TGTGGAAAAACAACGGACTGAG.    1

...((((.((((((((((.(.(((((....((..(((....)))..)).))))).).)))))))))))))) (-22.80 MFE
```

mir-n498

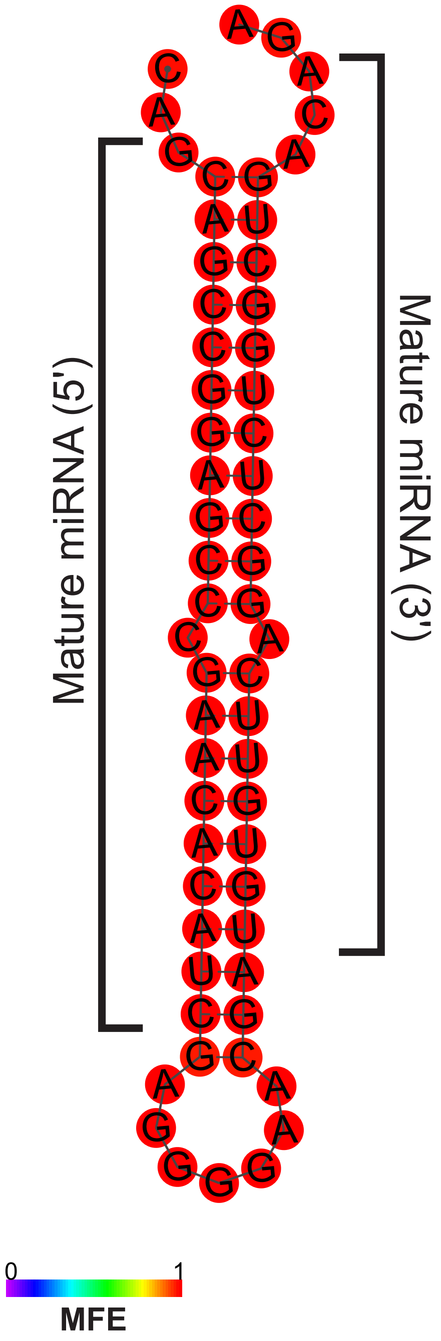

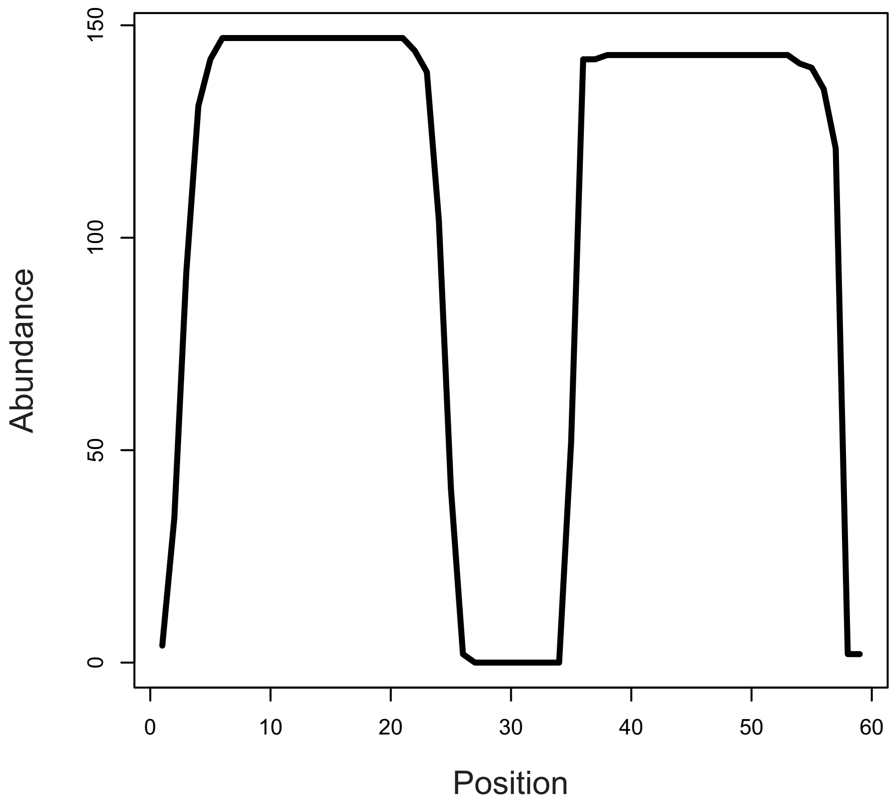


```
CAGCAGCCGGAGCCCGAACACATCGAGGGGAACGATGTGTTCAGGCTCTGGCTGACAGA      Raw reads
...................................TGTGTTCAGGCTCTGGCTGACA..      84
..GCAGCCGGAGCCCGAACACATC...................................      40
..................................ATGTGTTCAGGCTCTGGCTGACA..      35
...CAGCCGGAGCCCGAACACATCG..................................      28
..GCAGCCGGAGCCCGAACACAT....................................      17
.AGCAGCCGGAGCCCGAACACAT....................................      15
..................................ATGTGTTCAGGCTCTGGCTGAC...      12
.AGCAGCCGGAGCCCGAACACATC...................................      10
...CAGCCGGAGCCCGAACACATC...................................      9
....AGCCGGAGCCCGAACACATCG..................................      6
CAGCAGCCGGAGCCCGAACACA.....................................      4
.AGCAGCCGGAGCCCGAACAC......................................      3
.....GCCGGAGCCCGAACACATCG..................................      3
...................................TGTGTTCAGGCTCTGGCTGA....      3
...CAGCCGGAGCCCGAACACAT....................................      2
....AGCCGGAGCCCGAACACATC...................................      2
.....GCCGGAGCCCGAACACATC...................................      2
....AGCCGGAGCCCGAACACATCGA.................................      2
..................................ATGTGTTCAGGCTCTGGCT......      2
..................................ATGTGTTCAGGCTCTGGCTGA....      2
...................................TGTGTTCAGGCTCTGGCTGAC...      2
.AGCAGCCGGAGCCCGAACACA.....................................      1
....AGCCGGAGCCCGAACACAT....................................      1
.AGCAGCCGGAGCCCGAACACATCG..................................      1
..GCAGCCGGAGCCCGAACACATCG..................................      1
..................................ATGTGTTCAGGCTCTGGCTG.....      1
...................................TGTGTTCAGGCTCTGGCTGACAGA      1
.....................................TGTTCAGGCTCTGGCTGACAGA      1

..((((((((((((.((((((((((.......)))))))))).))))))))))).)...      (-39.70 MFE)
```

mir-n500

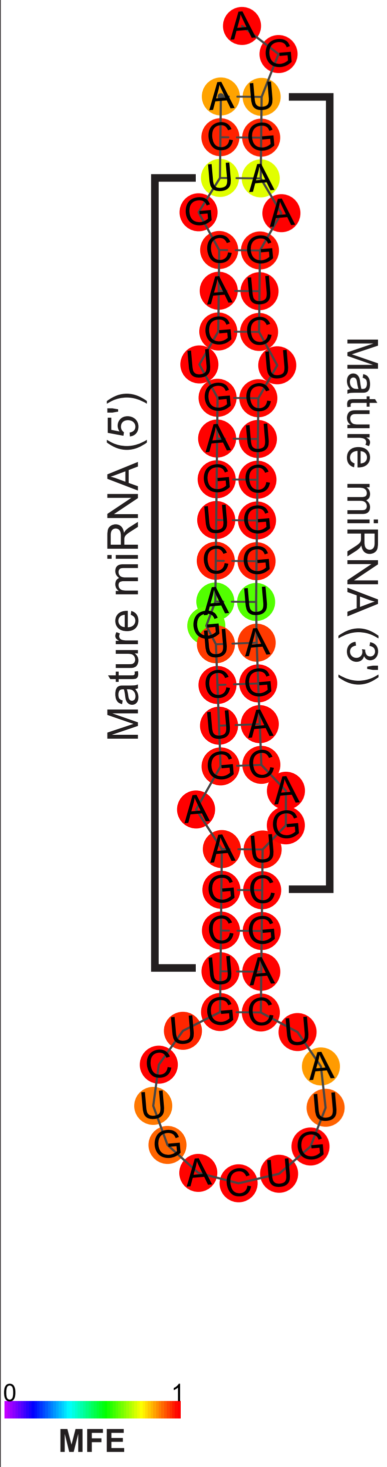

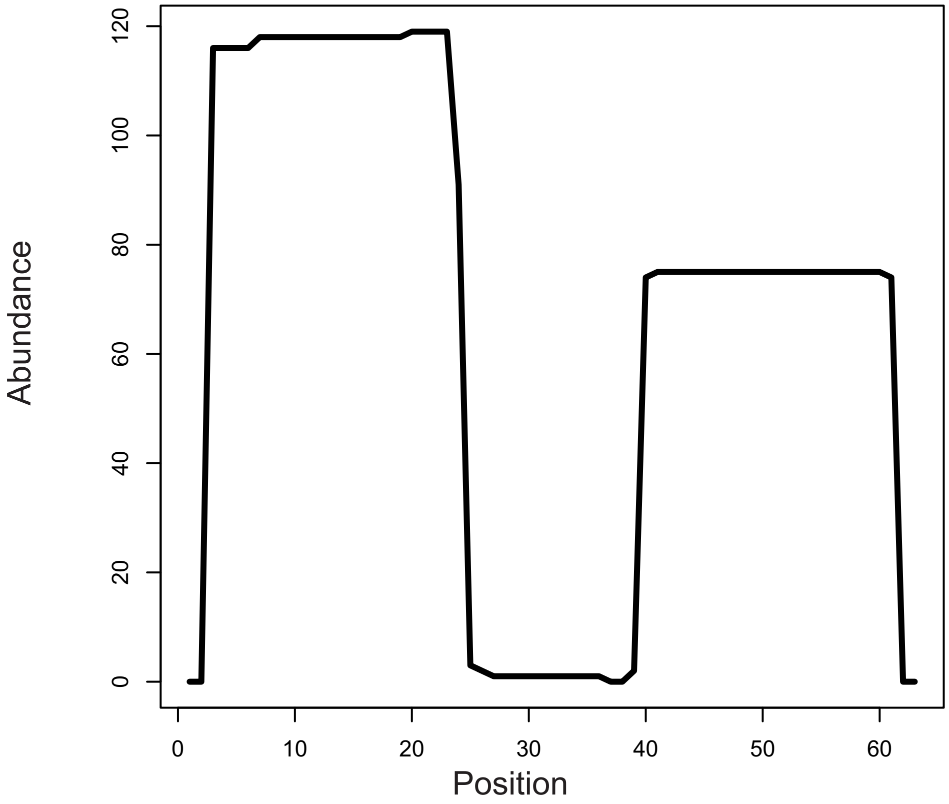


```
ACTGCAGTGAGTCAGTCTGAAGCTGTCTGACTGTATCAGCTGACAGATGGCTCTCTGAAGTGA   Raw reads
..TGCAGTGAGTCAGTCTGAAGCT.......................................   88
.......................................CTGACAGATGGCTCTCTGAAGT..   71
..TGCAGTGAGTCAGTCTGAAGC........................................   26
......GTGAGTCAGTCTGAAGC........................................   2
......................................GCTGACAGATGGCTCTCTGAAGT..   2
..TGCAGTGAGTCAGTCTGAAGCTG......................................   1
..TGCAGTGAGTCAGTCTGAAGCTGT.....................................   1
...................AAGCTGTCTGACTGTAT...........................   1
.......................................CTGACAGATGGCTCTCTGAAG...   1
........................................TGACAGATGGCTCTCTGAAGT..   1

(((.(((.((((((.((((.(((((.(.....)...)))))..)))))))))).))).))).. (-25.40 MFE
```

mir-n518

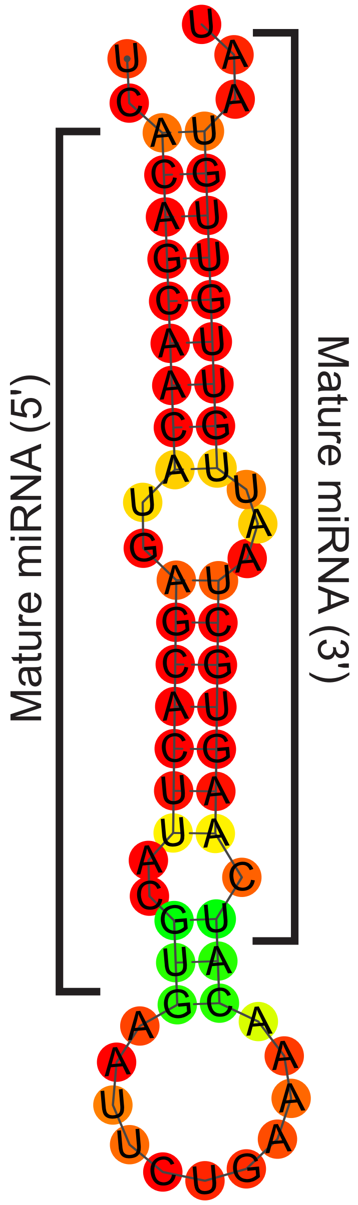

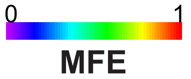

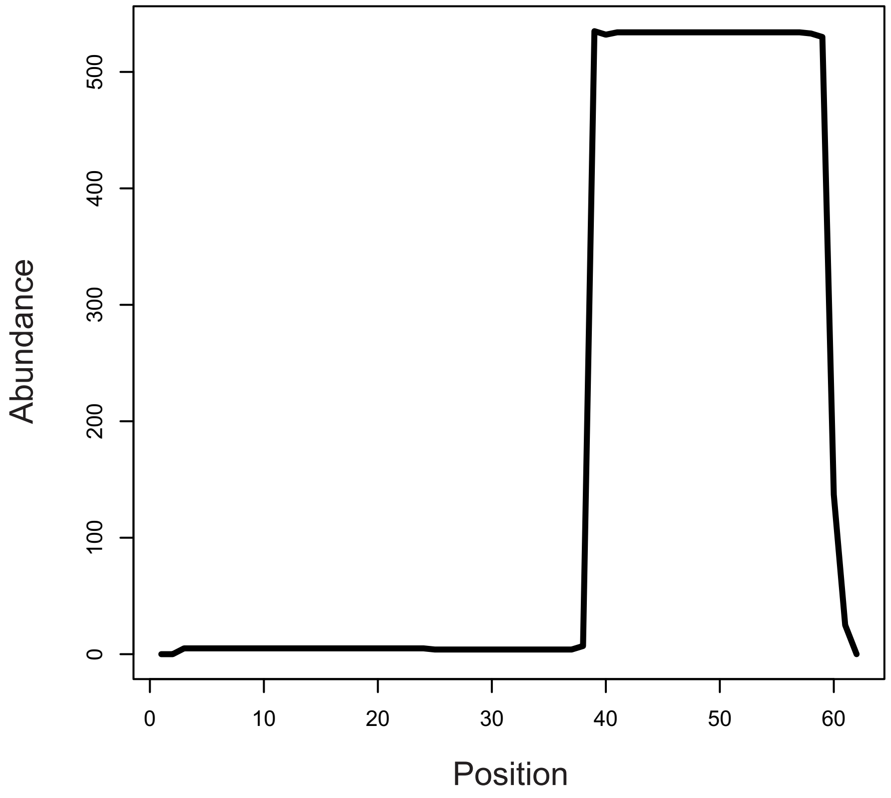


```
TCACAGCAACATGAGCACTTACGTGAATTCTGAAAACATCAAGTGCTAATTGTTGTTGTAAT   Raw reads
......................................TCAAGTGCTAATTGTTGTTGT...   389
......................................TCAAGTGCTAATTGTTGTTGTA..   110
......................................TCAAGTGCTAATTGTTGTTGTAA.   25
..ACAGCAACATGAGCACTTACGTGAATTCTGAAAACAT.......................   4
......................................TCAAGTGCTAATTGTTGTTG....   3
.....................................ATCAAGTGCTAATTGTTGTTGT...   3
..ACAGCAACATGAGCACTTACGT......................................   1
......................................TCAAGTGCTAATTGTTGTT.....   1
........................................AAGTGCTAATTGTTGTTGT...   1
.......................................CAAGTGCTAATTGTTGTTGTA..   1
........................................AAGTGCTAATTGTTGTTGTA..   1

..(((((((((..(((((((..(((...........))).)))))))...)))))))))...   (-20.40 MFE)
```

mir-n529

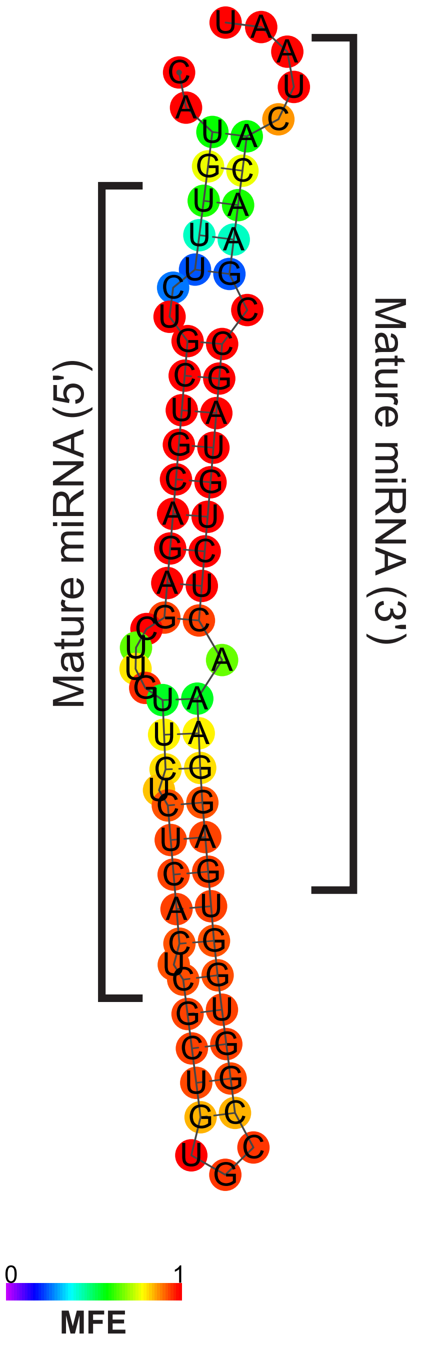

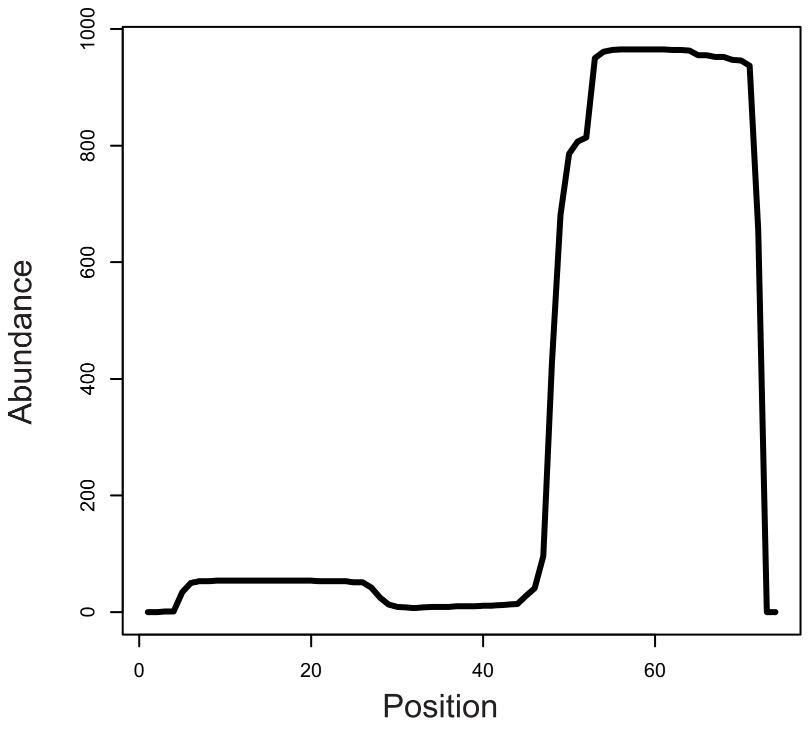


```
CATGTTTCTGCTGCAGAGCTTGTTCTCTCACTCGCTGTGCCGGTGGTGAGGAAACTCTGTAGCCGAACACTAAT  Raw reads
...............................................GAGGAAACTCTGTAGCCGAACACTA..  260
................................................AGGAAACTCTGTAGCCGAACACTA..  191
....................................................AACTCTGTAGCCGAACACT...  96
.................................................GGAAACTCTGTAGCCGAACACTA..  86
...............................................GAGGAAACTCTGTAGCCGAACACT...  66
................................................AGGAAACTCTGTAGCCGAACACT...  60
....................................................AACTCTGTAGCCGAACACTA..  38
..............................................TGAGGAAACTCTGTAGCCGAACACTA..  36
.................................................GGAAACTCTGTAGCCGAACACT...  19
..................................................GAAACTCTGTAGCCGAACACTA..  15
....TTTCTGCTGCAGAGCTTGTTCTC...............................................  14
.............................................GTGAGGAAACTCTGTAGCCGAACACT...  10
..............................................TGAGGAAACTCTGTAGCCGAACACT...  9
............................................GGTGAGGAAACTCTGTAGCCGAACACTA..  9
....TTTCTGCTGCAGAGCTTGTTCT................................................  6
....TTTCTGCTGCAGAGCTTGTTCTCT..............................................  6
.....TTCTGCTGCAGAGCTTGTTCTCT..............................................  6
..................................................GAAACTCTGTAGCCGAACACT...  6
.....................................................ACTCTGTAGCCGAACACTA..  6
..............................................TGAGGAAACTCTGTAGCCGAAC......  5
............................................GGTGAGGAAACTCTGTAGCCGAACACT...  5
.....................................................ACTCTGTAGCCGAACACT...  5
...................................................AAACTCTGTAGCCGAACACTA..  5
....TTTCTGCTGCAGAGCTTGTTCTCTCA............................................  4
...............................................GAGGAAACTCTGTAGCCGAACAC....  4
.....TTCTGCTGCAGAGCTTGTTCT................................................  3
................................CGCTGTGCCGGTGGTGAGGAAACTCTGTAGCC..........  3
..............................................TGAGGAAACTCTGTAGCCGA........  3
.....TTCTGCTGCAGAGCTTGTTCTC...............................................  2
....TTTCTGCTGCAGAGCTTGTTCTCTC.............................................  2
.....TTCTGCTGCAGAGCTTGTTCTCTCACT..........................................  2
..............................CTCGCTGTGCCGGTGGTGAGGAAACTCTGTAGCC..........  2
................................................AGGAAACTCTGTAGCC..........  2
................................................AGGAAACTCTGTAGCCGAACAC....  2
....................................................AACTCTGTAGCCGAACAC....  2
...................................................AAACTCTGTAGCCGAACACT...  2
......................................................CTCTGTAGCCGAACACT...  2
..............................CTCGCTGTGCCGGTGGTGAGGAAACTCTGTAGCCGAACACTA..  2
.............................................GTGAGGAAACTCTGTAGCCGAACACTA..  2
..TGTTTCTGCTGCAGAGCT......................................................  1
....TTTCTGCTGCAGAGCTTGTT..................................................  1
........TGCTGCAGAGCTTGTT..................................................  1
......TCTGCTGCAGAGCTTGTTCTC...............................................  1
.....TTCTGCTGCAGAGCTTGTTCTCTC.............................................  1
......TCTGCTGCAGAGCTTGTTCTCTC.............................................  1
.....TTCTGCTGCAGAGCTTGTTCTCTCA............................................  1
.....TTCTGCTGCAGAGCTTGTTCTCTCAC...........................................  1
......TCTGCTGCAGAGCTTGTTCTCTCACT..........................................  1
.................................GCTGTGCCGGTGGTGAGGAAACTCTGTA.............  1
....................................GTGCCGGTGGTGAGGAAACTCTGTAGC...........  1
..............................................TGAGGAAACTCTGTAGCC..........  1
.............................................GTGAGGAAACTCTGTAGCCGAACA.....  1
..............................................TGAGGAAACTCTGTAGCCGAACAC....  1
.......................................CCGGTGGTGAGGAAACTCTGTAGCCGAACACT...  1
.......................................................TCTGTAGCCGAACACT...  1
................................CGCTGTGCCGGTGGTGAGGAAACTCTGTAGCCGAACACTA..  1
.........................................GGTGGTGAGGAAACTCTGTAGCCGAACACTA..  1
..........................................GTGGTGAGGAAACTCTGTAGCCGAACACTA..  1
...........................................TGGTGAGGAAACTCTGTAGCCGAACACTA..  1
......................................................CTCTGTAGCCGAACACTA..  1

..(((((..((((((((....(((.(((((.(((((...))))))))))))).))))))))).))))).....  (-31.40 MFE)
```

mir-n579

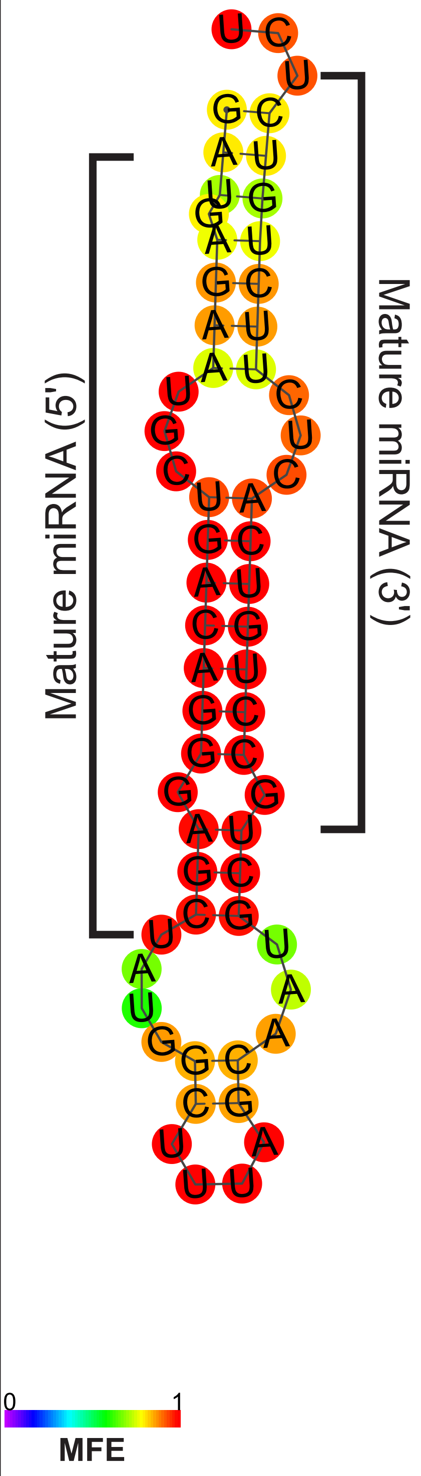

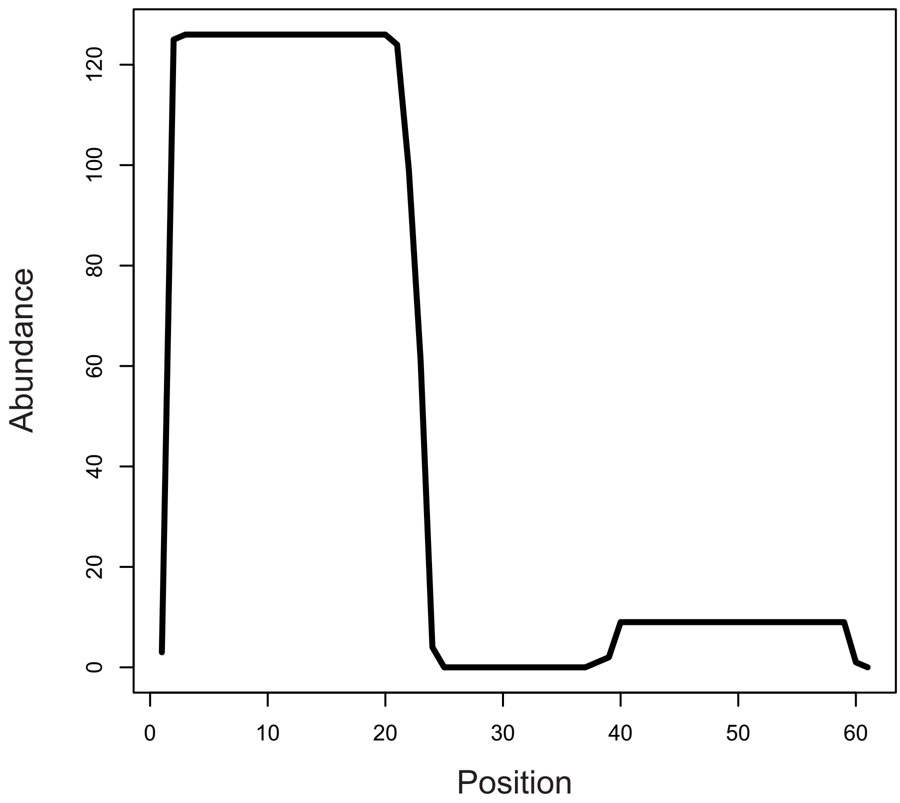


```
GATGAGAATGCTGACAGGGAGCTATGGCTTTAGCAATGCTGCCTGTCACTCTTCTGTCTCT     Raw read
.ATGAGAATGCTGACAGGGAGCT......................................     55
.ATGAGAATGCTGACAGGGAGC.......................................     37
.ATGAGAATGCTGACAGGGAG........................................     24
.......................................TGCCTGTCACTCTTCTGTCT..     6
.ATGAGAATGCTGACAGGGAGCTA.....................................     4
.ATGAGAATGCTGACAGGGA.........................................     2
GATGAGAATGCTGACAGGGAG........................................     1
GATGAGAATGCTGACAGGGAGC.......................................     1
GATGAGAATGCTGACAGGGAGCT......................................     1
..TGAGAATGCTGACAGGGAGCT......................................     1
.....................................GCTGCCTGTCACTCTTCTGTCT..     1
......................................CTGCCTGTCACTCTTCTGTCT..     1
.......................................TGCCTGTCACTCTTCTGTCTC.     1

(((.((((.(.(((((((.(((....((....))...))).))))))).).)))))))... (-23.50 MFE)
```

mir-n612

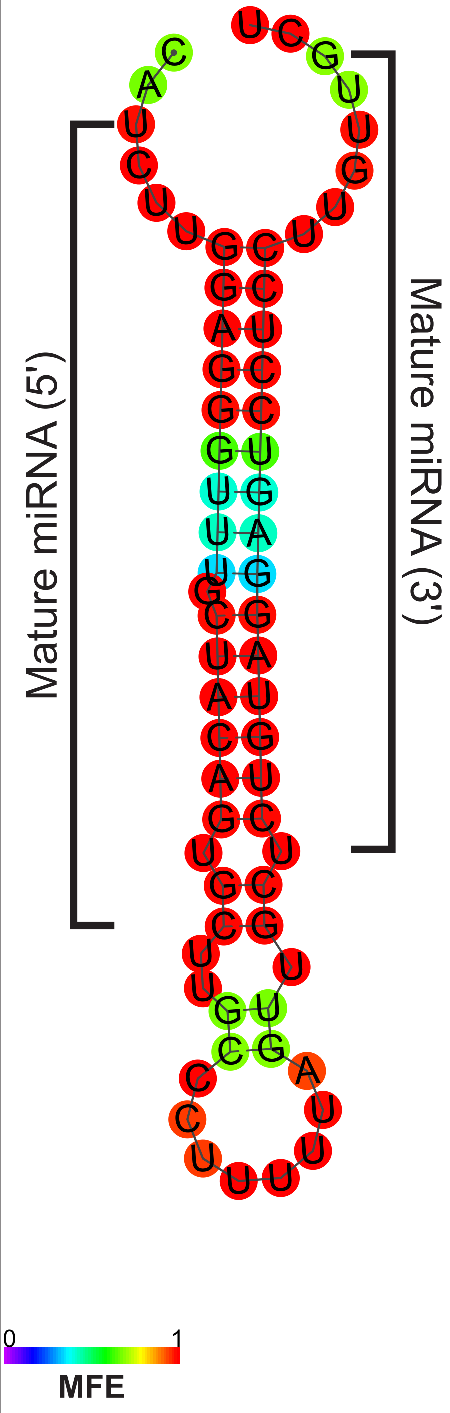

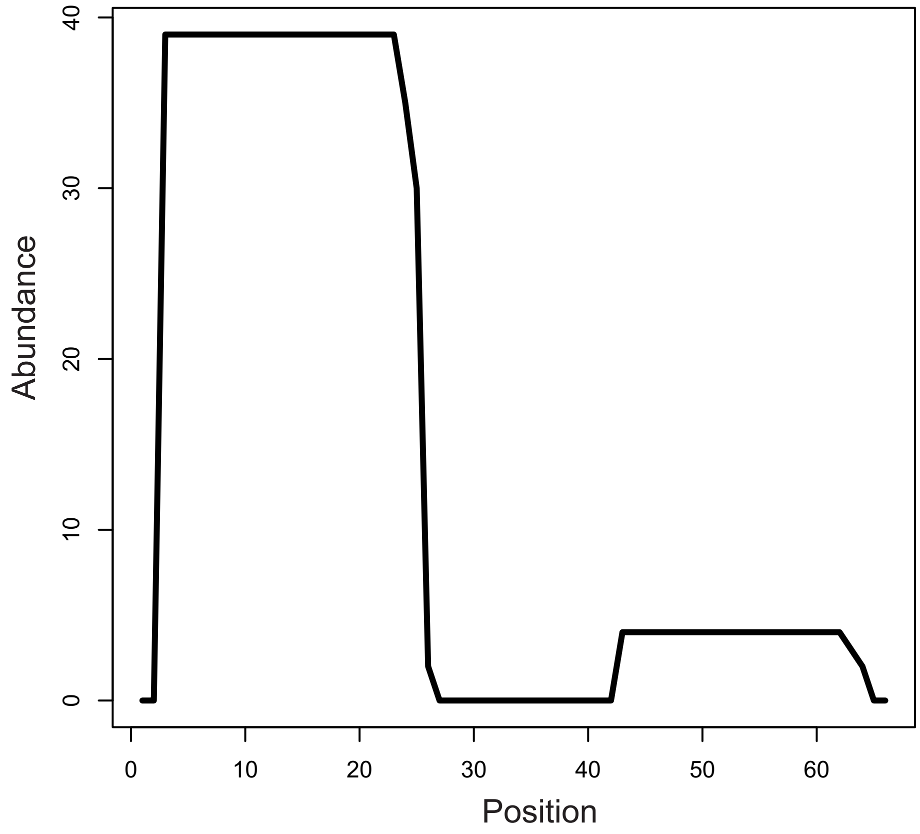


```
CATCTTGGAGGGTTTGCTACAGTGCTTGCCCTTTTTAGTTGCTCTGTAGGAGTCCTCCTTGTTGCT    Raw reads
..TCTTGGAGGGTTTGCTACAGTGC.........................................    28
..TCTTGGAGGGTTTGCTACAGTG..........................................    5
..TCTTGGAGGGTTTGCTACAGT...........................................    4
..TCTTGGAGGGTTTGCTACAGTGCT........................................    2
..........................................TCTGTAGGAGTCCTCCTTGTTG..    2
..........................................TCTGTAGGAGTCCTCCTTGT....    1
..........................................TCTGTAGGAGTCCTCCTTGTT...    1

((.(..(((((..((.((((((.((..((........)).)).))))))))..))))).).))..    (-23.50 MFE)
```

mir-n688

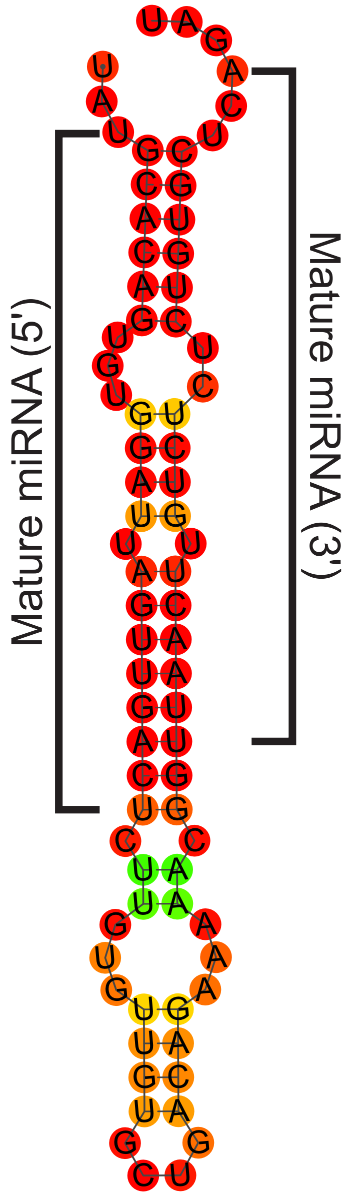

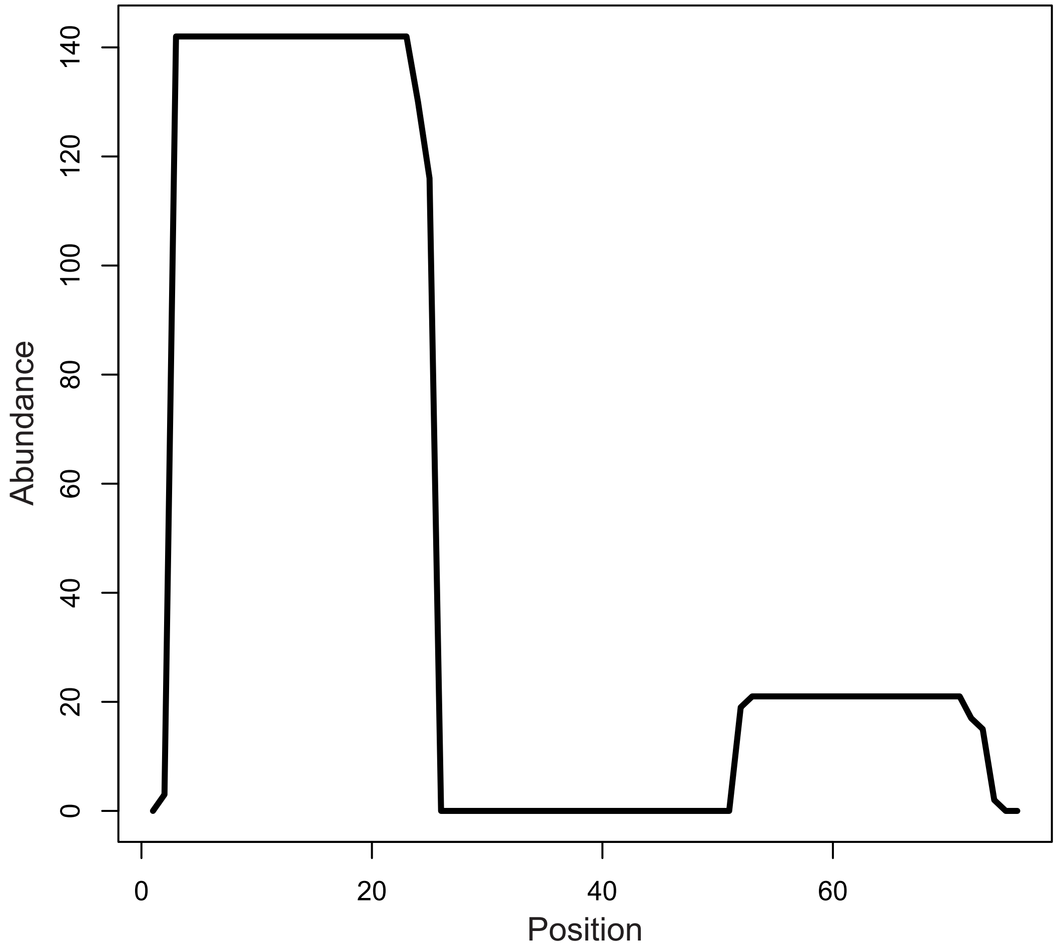


```
TATGCACAGTGTGGATTAGTTGACTCTTGTGTTGTGCTGACAGAAAAACGGTTAACTTGTCTCTCTGTGCTCAGAT      Raw read
..TGCACAGTGTGGATTAGTTGACT...................................................      115
..TGCACAGTGTGGATTAGTTGAC....................................................      13
...................................................TTAACTTGTCTCTCTGTGCTCA...      12
..TGCACAGTGTGGATTAGTTGA.....................................................      11
...................................................TTAACTTGTCTCTCTGTGCT.....      4
...................................................TTAACTTGTCTCTCTGTGCTC....      2
.ATGCACAGTGTGGATTAGTTGA.....................................................      1
.ATGCACAGTGTGGATTAGTTGAC....................................................      1
.ATGCACAGTGTGGATTAGTTGACT...................................................      1
....................................................TAACTTGTCTCTCTGTGCTCA...      1
...................................................TTAACTTGTCTCTCTGTGCTCAG..      1
....................................................TAACTTGTCTCTCTGTGCTCAG..      1

...((((((.(.((((.((((((((.((...((((....))))...)).)))))))).))))).))))))...... (-25.10 MFE)
```

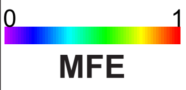

mir-n692

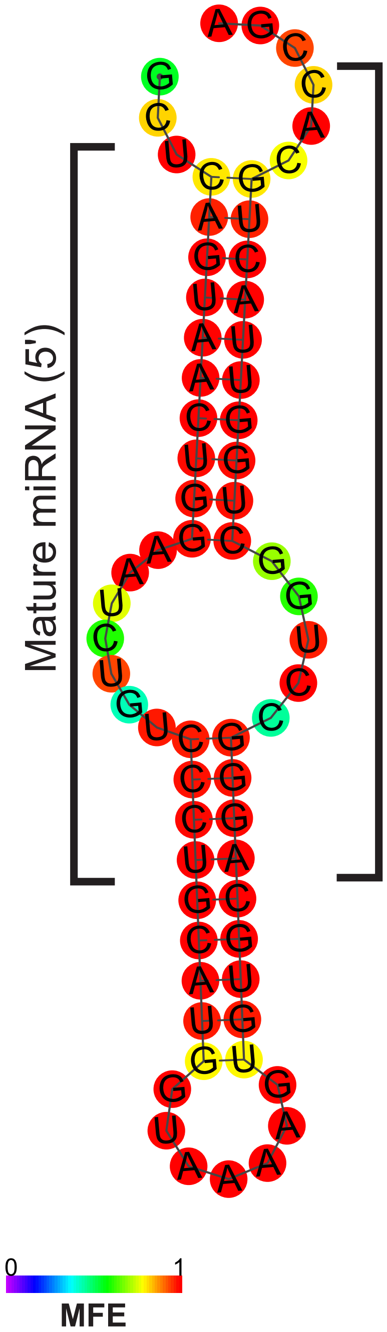

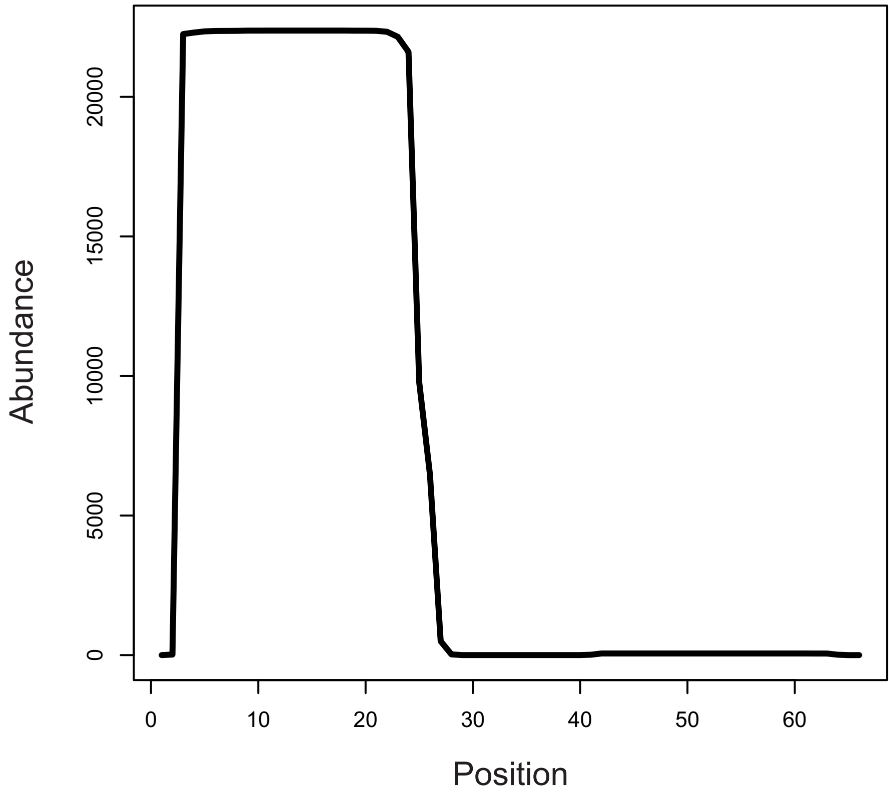


```
GCTCAGTAACTGGAATCTGTCCCTGCATGGTAAAAGTGTGCAGGGCCTGGCTGGTTACTGCACCGA        Raw reads
..TCAGTAACTGGAATCTGTCCCT..........................................        11772
..TCAGTAACTGGAATCTGTCCCTGC........................................        5914
..TCAGTAACTGGAATCTGTCCCTG.........................................        3287
..TCAGTAACTGGAATCTGTCCC...........................................        539
..TCAGTAACTGGAATCTGTCCCTGCA.......................................        468
..TCAGTAACTGGAATCTGTCC............................................        180
..TCAGTAACTGGAATCTGTC.............................................        33
.........................................AGGGCCTGGCTGGTTACTGCAC...        31
...CAGTAACTGGAATCTGTCCCT..........................................        27
..TCAGTAACTGGAATCTGTCCCTGCAT......................................        26
....AGTAACTGGAATCTGTCCCT..........................................        22
...CAGTAACTGGAATCTGTCCCTGC........................................        19
.....GTAACTGGAATCTGTCCCTGC........................................        14
.........................................AGGGCCTGGCTGGTTACTGCACC..        14
.CTCAGTAACTGGAATCTGTCCCT..........................................        11
........................................CAGGGCCTGGCTGGTTACTGCAC...        11
....AGTAACTGGAATCTGTCCCTGC........................................        9
.CTCAGTAACTGGAATCTGTCCCTGC........................................        8
...CAGTAACTGGAATCTGTCCCTG.........................................        7
....AGTAACTGGAATCTGTCCCTG.........................................        6
..TCAGTAACTGGAATCT................................................        4
..TCAGTAACTGGAATCTGT..............................................        4
........ACTGGAATCTGTCCCT..........................................        3
........ACTGGAATCTGTCCCTGC........................................        3
....AGTAACTGGAATCTGTCCCTGCA.......................................        3
.CTCAGTAACTGGAATCTGTCCCTG.........................................        2
........ACTGGAATCTGTCCCTG.........................................        2
........................................CAGGGCCTGGCTGGTTACTGC.....        2
....AGTAACTGGAATCTGTC.............................................        1
....AGTAACTGGAATCTGTCC............................................        1
....AGTAACTGGAATCTGTCCC...........................................        1
.....GTAACTGGAATCTGTCCCTG.........................................        1
......TAACTGGAATCTGTCCCTG.........................................        1
.......AACTGGAATCTGTCCCTG.........................................        1
......TAACTGGAATCTGTCCCTGC........................................        1
.......AACTGGAATCTGTCCCTGC........................................        1
..........TGGAATCTGTCCCTGC........................................        1
..TCAGTAACTGGAATCTGTCCCTGCATGGTAAAAGTGTG..........................        1
.........................................AGGGCCTGGCTGGTTACTGCA....        1
........................................CAGGGCCTGGCTGGTTACTGCACC..        1

...(((((((..(.....(.((((((((.........)))))))).)...)..)))))))......        (-25.60 MFE)
```

mir-n693

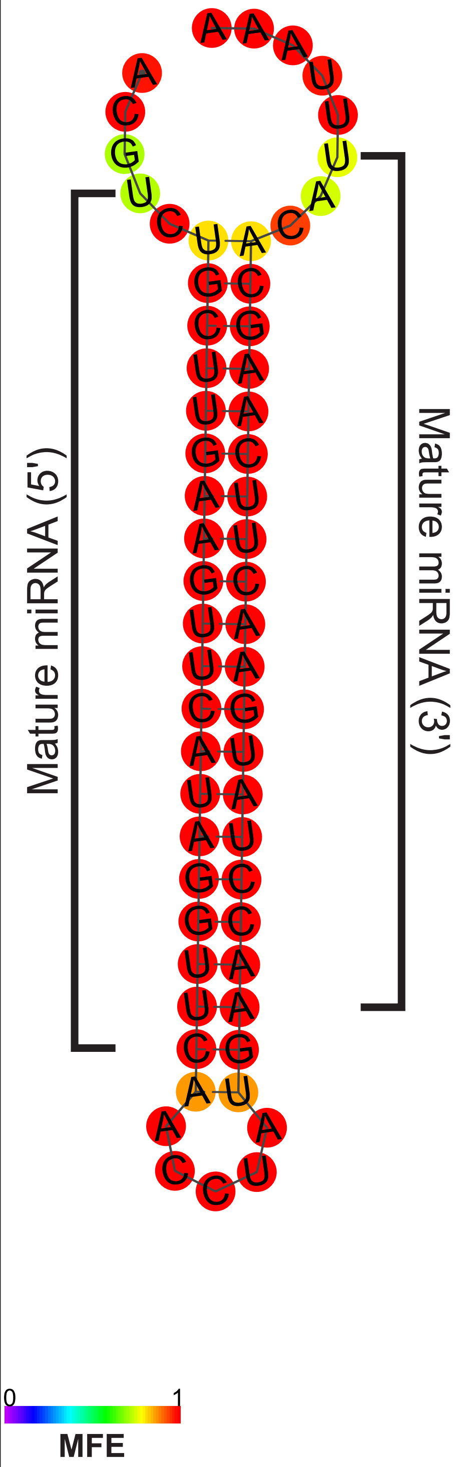

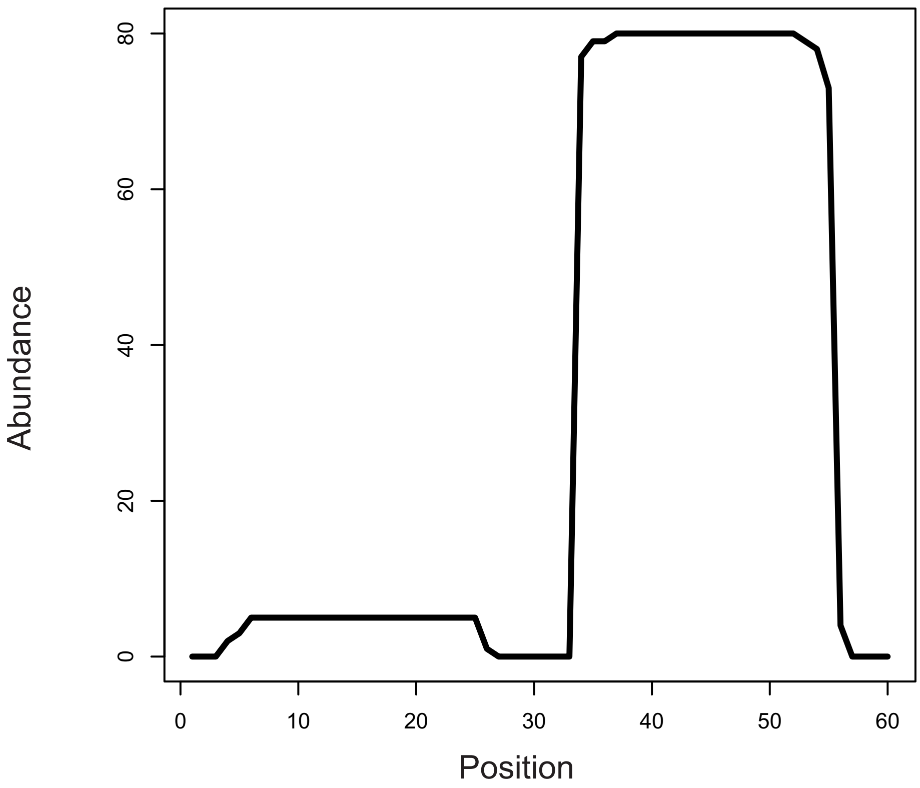


```
ACGTCTGCTTGAAGTTCATAGGTTCAACCTATGAACCTATGAACTTCAAGCACATTTAAA     Raw reads
.................................AACCTATGAACTTCAAGCACAT.....     67
.................................AACCTATGAACTTCAAGCACA......     5
.................................AACCTATGAACTTCAAGCACATT....     3
...TCTGCTTGAAGTTCATAGGTTC...................................     2
....CTGCTTGAAGTTCATAGGTTC...................................     1
.....TGCTTGAAGTTCATAGGTTC...................................     1
.....TGCTTGAAGTTCATAGGTTCA..................................     1
.................................AACCTATGAACTTCAAGCA........     1
.................................AACCTATGAACTTCAAGCAC.......     1
...................................ACCTATGAACTTCAAGCACAT.....     1
....................................CTATGAACTTCAAGCACAT.....     1
..................................ACCTATGAACTTCAAGCACATT....     1

.....(((((((((((((((((((((.....)))))))))))))))))))))........ (-33.80 MFE)
```

mir-n713

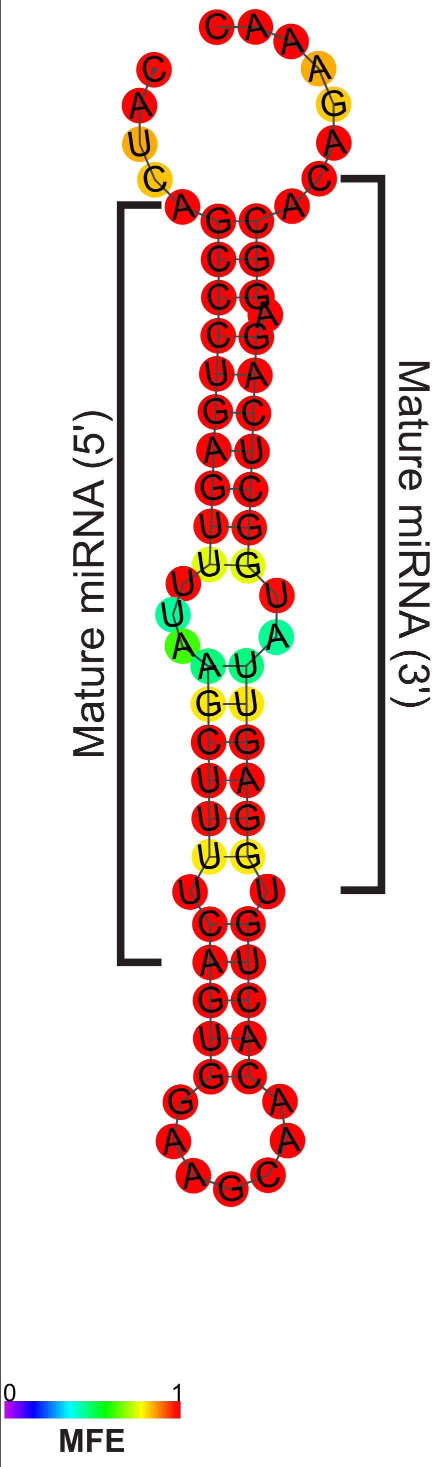

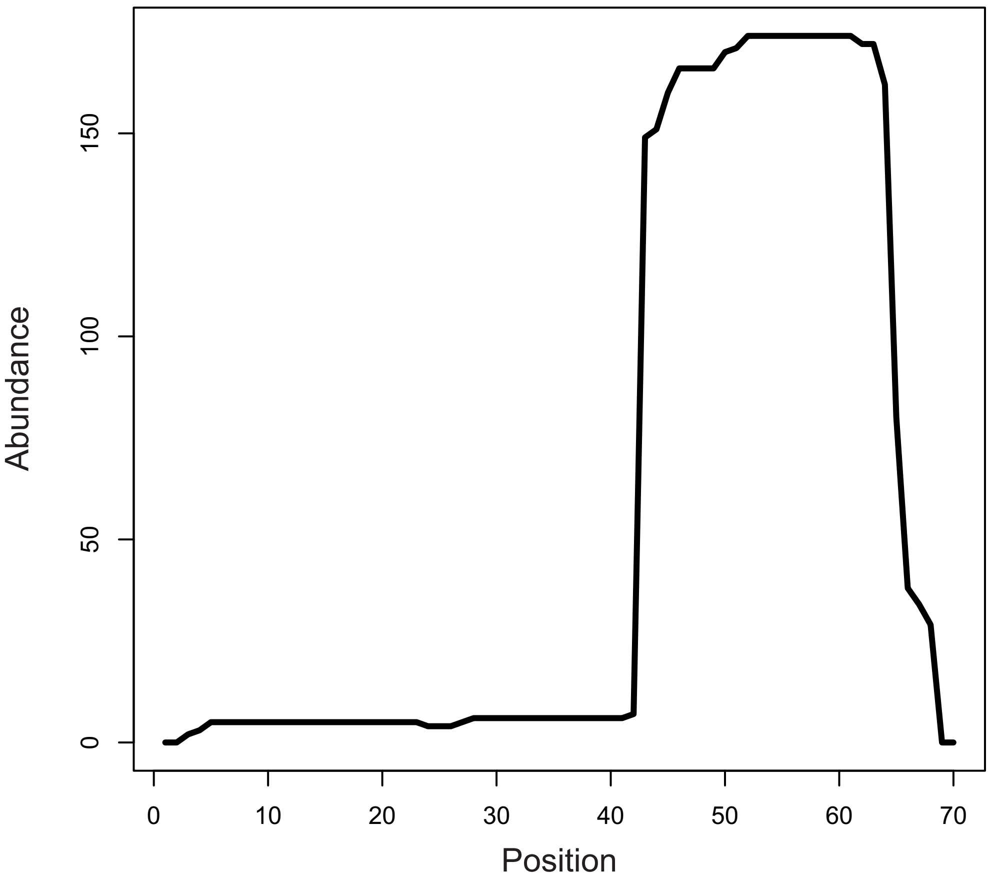


```
CATCAGCCCTGAGTTTTAAGCTTTTCAGTGGAAGCAACACTGTGGAGTTATGGCTCAGAGGCACAGAAAC    Raw read
..........................................TGGAGTTATGGCTCAGAGGCAC......    80
..........................................TGGAGTTATGGCTCAGAGGCACA.....    42
..........................................TGGAGTTATGGCTCAGAGGCA.......    10
............................................GAGTTATGGCTCAGAGGCACAGAA..    9
.............................................AGTTATGGCTCAGAGGCACAGAA..    6
..........................................TGGAGTTATGGCTCAGAGGCACAGA...    4
.................................................ATGGCTCAGAGGCACAGAA..    4
..........................................TGGAGTTATGGCTCAGAGGCACAG....    3
..........................AGTGGAAGCAACACTGTGGAGTTATGGCTCAGAGGCACAGAA..    3
....AGCCCTGAGTTTTAAGCTTTTCA...........................................    2
..........................................TGGAGTTATGGCTCAGAGG.........    2
...........................GTGGAAGCAACACTGTGGAGTTATGGCTCAGAGGCACAGAA..    2
...................................................GGCTCAGAGGCACAGAA..    2
..TCAGCCCTGAGTTTTAAGCTT...............................................    1
..TCAGCCCTGAGTTTTAAGCTTTTC............................................    1
...CAGCCCTGAGTTTTAAGCTTTTC............................................    1
...........................GTGGAAGCAACACTGTGGAGTTATGGCTCAGAGGCAC......    1
...........................................GGAGTTATGGCTCAGAGGCAC......    1
...........................................GGAGTTATGGCTCAGAGGCACAG....    1
...................................................GGCTCAGAGGCACAGA...    1
.........................................GTGGAGTTATGGCTCAGAGGCACAGAA..    1
..........................................TGGAGTTATGGCTCAGAGGCACAGAA..    1
..................................................TGGCTCAGAGGCACAGAA..    1

.....(((((((((...(((((..(((((.......)))))..)))))..))))))).)))........ (-28.20
```

mir-n737

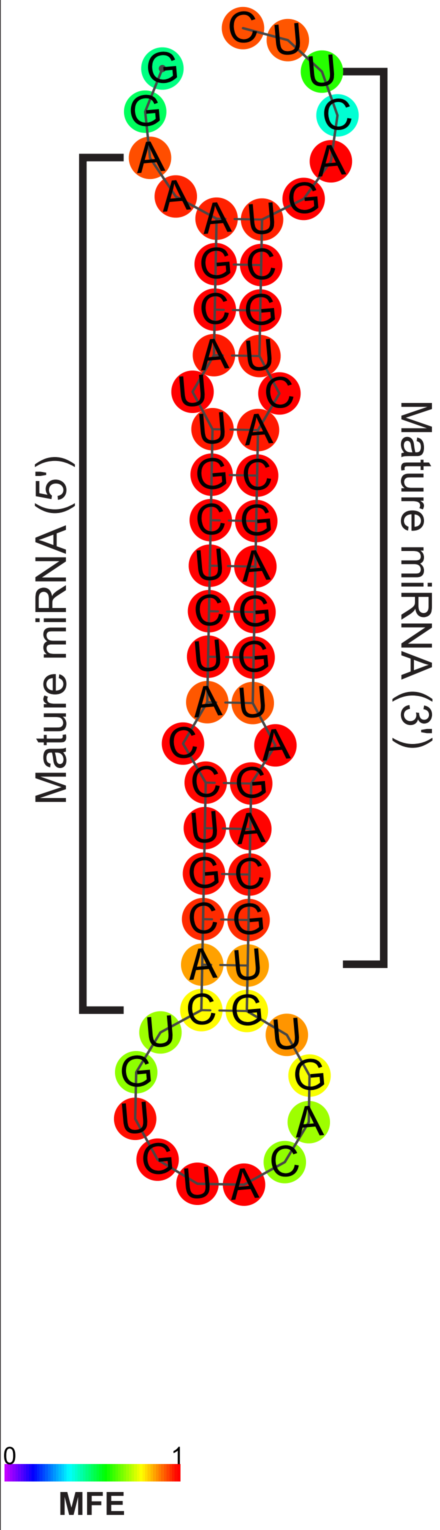

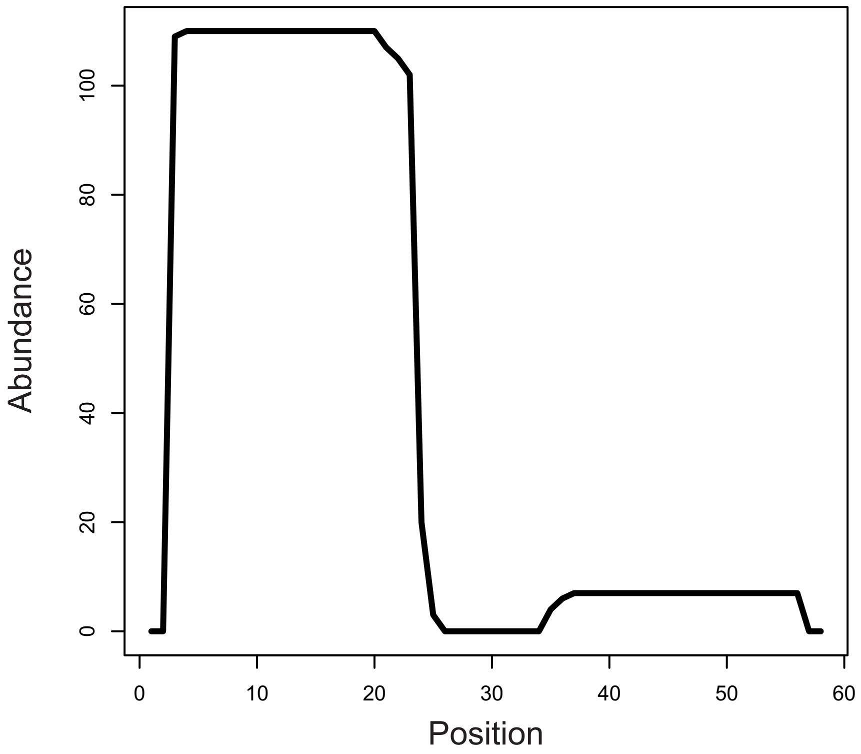


```
GGAAAGCATTGCTCTACCTGCACTGTGTACAGTGTGCAGATGGAGCACTGCTGACTTC        Raw reads
..AAAGCATTGCTCTACCTGCAC...................................        81
..AAAGCATTGCTCTACCTGCACT..................................        17
..................................TGCAGATGGAGCACTGCTGACT..        4
..AAAGCATTGCTCTACCTG......................................        3
..AAAGCATTGCTCTACCTGCA....................................        3
..AAAGCATTGCTCTACCTGCACTG.................................        3
..AAAGCATTGCTCTACCTGC.....................................        2
...................................GCAGATGGAGCACTGCTGACT..        2
...AAGCATTGCTCTACCTGCAC...................................        1
....................................CAGATGGAGCACTGCTGACT..        1

((..((((.(((((((.((((((..........)))))).))))))).))))..))..        (-25.90 MFE)
```

mir-n739

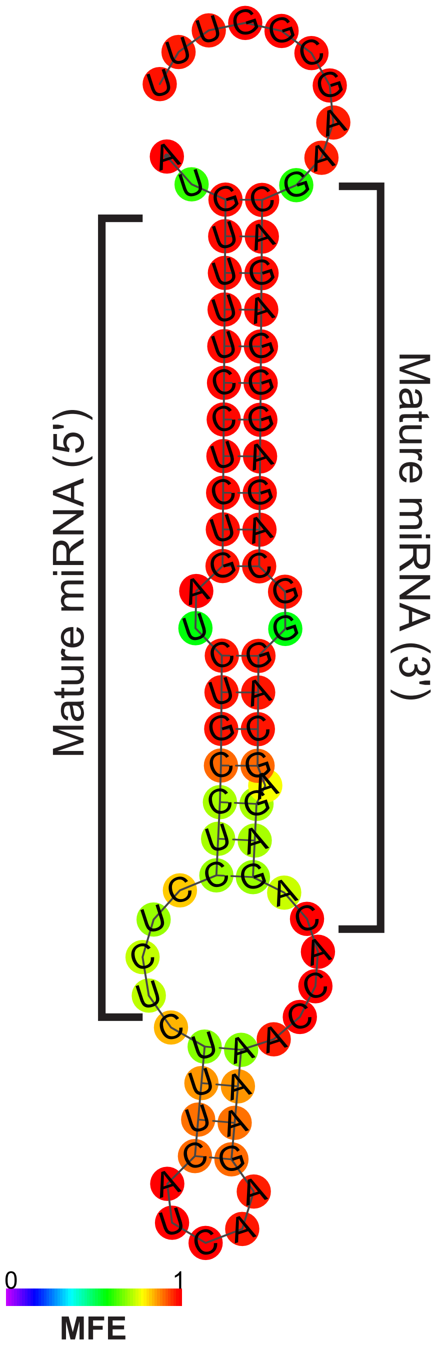

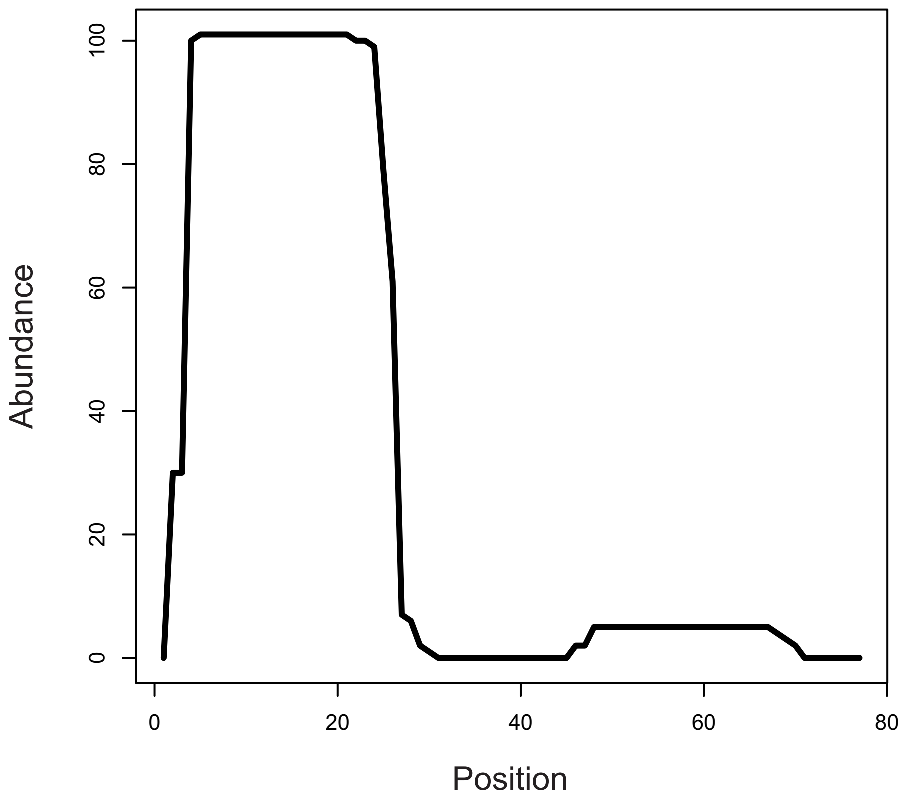


```
ATGTTTTCCTCTGATCTGCCTCCTCTCTTTCATCAAGAAAACCACAGAGAGCAGGGCAGAGGGAGACGAAGCGGTTT        Raw reads
...TTTTCCTCTGATCTGCCTCCTCT...................................................        42
.TGTTTTCCTCTGATCTGCCTCCT.....................................................        13
...TTTTCCTCTGATCTGCCTCCTC....................................................        13
.TGTTTTCCTCTGATCTGCCTCCTCT...................................................        11
...TTTTCCTCTGATCTGCCTCCT.....................................................        7
.TGTTTTCCTCTGATCTGCCTCCTC....................................................        5
...TTTTCCTCTGATCTGCCTCCTCTCT.................................................        4
.............................................AGAGAGCAGGGCAGAGGGAGACG.........        1
...............................................AGAGCAGGGCAGAGGGAGACGAA.......        2
...TTTTCCTCTGATCTGCCT........................................................        1
.TGTTTTCCTCTGATCTGCCTCC......................................................        1
....TTTCCTCTGATCTGCCTCCTCT...................................................        1
...TTTTCCTCTGATCTGCCTCCTCTC..................................................        1
...TTTTCCTCTGATCTGCCTCCTCTCTT................................................        1
...TTTTCCTCTGATCTGCCTCCTCTCTTT...............................................        1
.............................................AGAGAGCAGGGCAGAGGGAGAC..........        1
...............................................AGAGCAGGGCAGAGGGAGACGA........        1

.((((((((((((..(((((((.....((((.....))))......))).))))..)))))))))))).........        (-29.70 MFE)
```

mir-n741

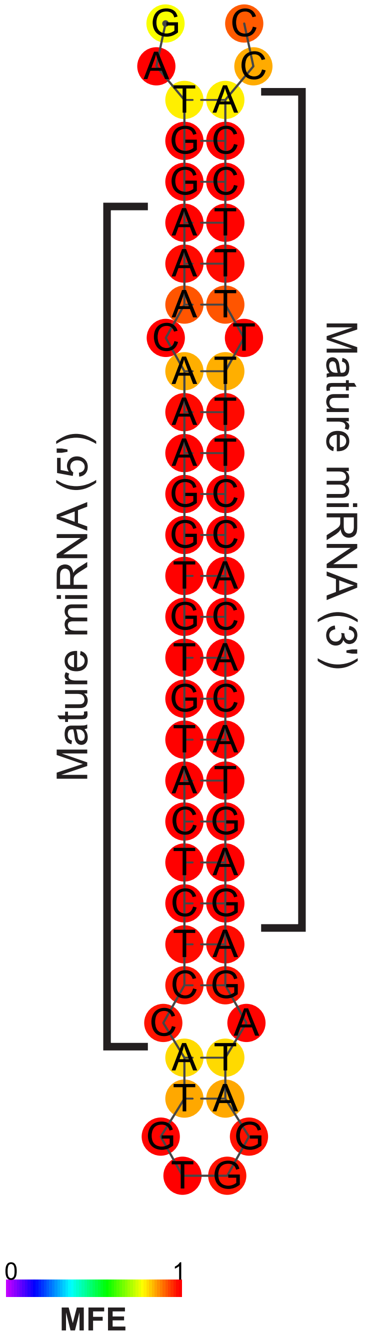

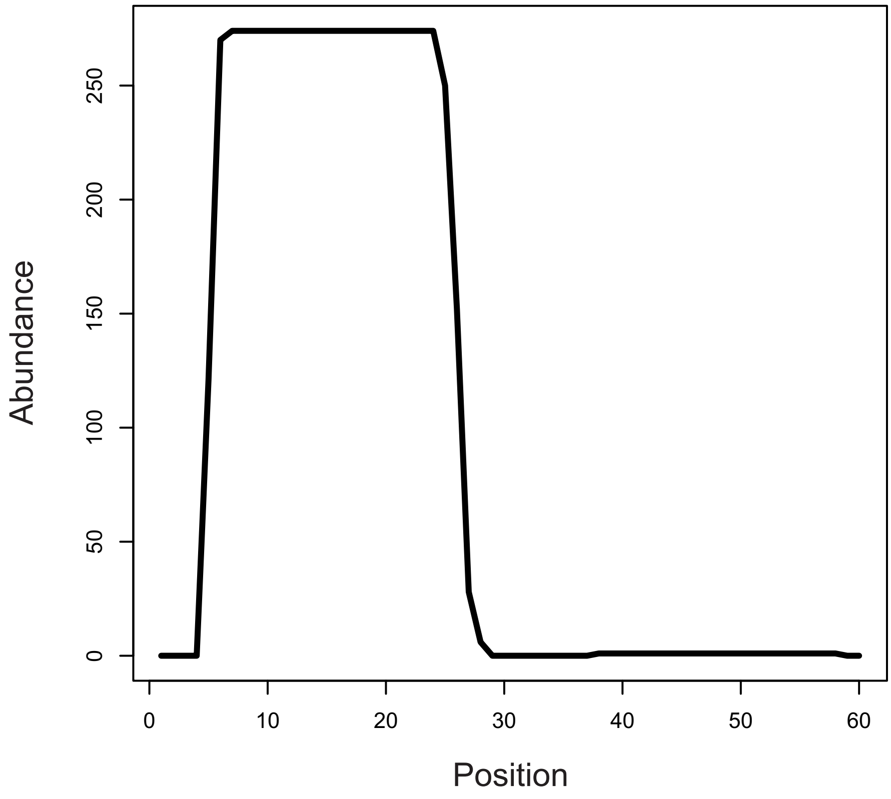


```
GATGGAAACAAAGGTGTGTACTCTCCATGTGGATAGAGAGTACACACCTTTTTTTCCACC     Raw reads
.....AAACAAAGGTGTGTACTCTCC..................................     106
....GAAACAAAGGTGTGTACTCTC...................................     91
.....AAACAAAGGTGTGTACTCTCCA.................................     22
....GAAACAAAGGTGTGTACTCT....................................     16
....GAAACAAAGGTGTGTACTCTCC..................................     14
.....AAACAAAGGTGTGTACTCT....................................     8
.....AAACAAAGGTGTGTACTCTC...................................     7
.....AAACAAAGGTGTGTACTCTCCAT................................     6
......AACAAAGGTGTGTACTCTCC..................................     4
.....................................GAGTACACACCTTTTTTTCCA..     1

..((((((.((((((((((((((((.((....)).)))))))))))))))).))))))..     (-33.80 MFE)
```

mir-n745

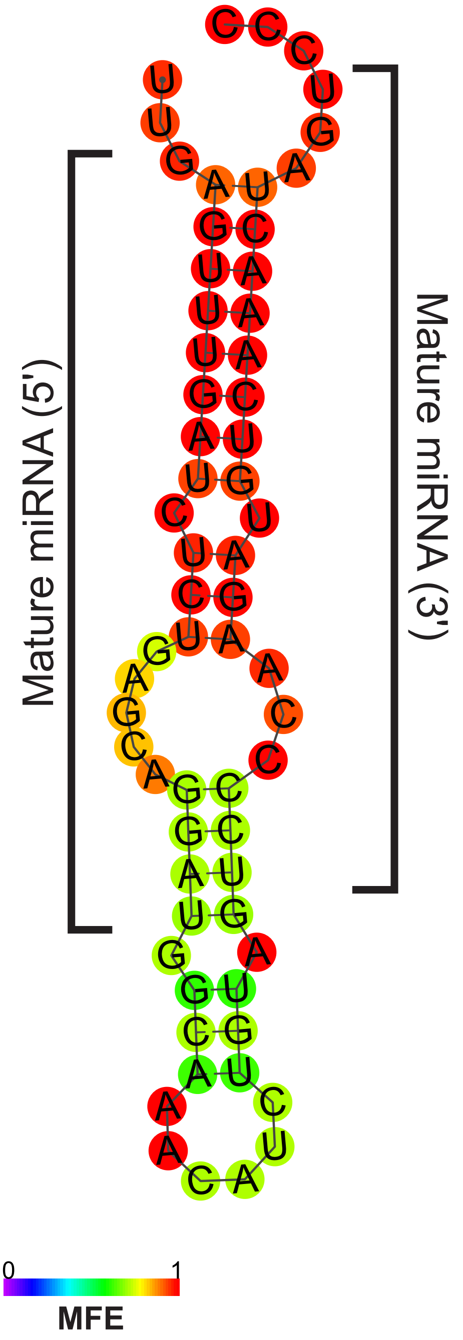

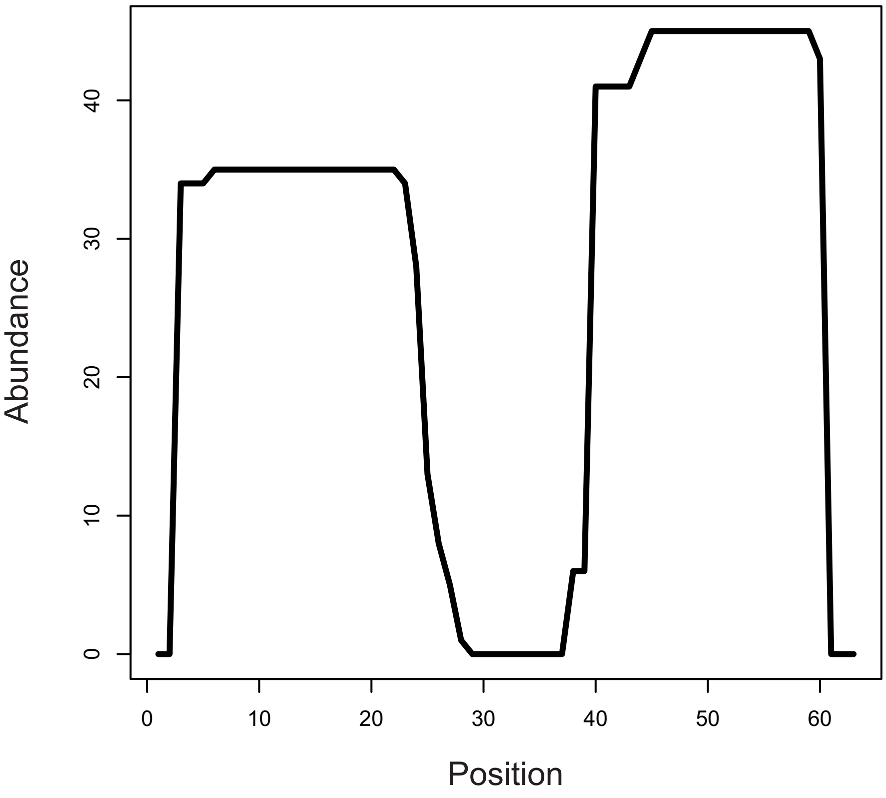


```
TTGAGTTTGATCTCTGAGCAGGATGGCAAACATCTGTAGTCCCCAAGATGTCAAACTAGTCCC   Raw reads
.......................................TCCCCAAGATGTCAAACTAGT...   33
..GAGTTTGATCTCTGAGCAGGAT.......................................   15
..GAGTTTGATCTCTGAGCAGGA........................................   6
.....................................AGTCCCCAAGATGTCAAACTAGT...   6
..GAGTTTGATCTCTGAGCAGGATG......................................   5
..GAGTTTGATCTCTGAGCAGGATGGC....................................   4
..GAGTTTGATCTCTGAGCAGGATGG.....................................   3
.......................................TCCCCAAGATGTCAAACTAG....   2
...........................................CAAGATGTCAAACTAGT...   2
............................................AAGATGTCAAACTAGT...   2
..GAGTTTGATCTCTGAGCAGG.........................................   1
.....TTTGATCTCTGAGCAGGATGGCA...................................   1

...((((((((.(((.....((((.(((......))).))))...))).))))))))......   (-17.60 MFE)
```

mir-n762

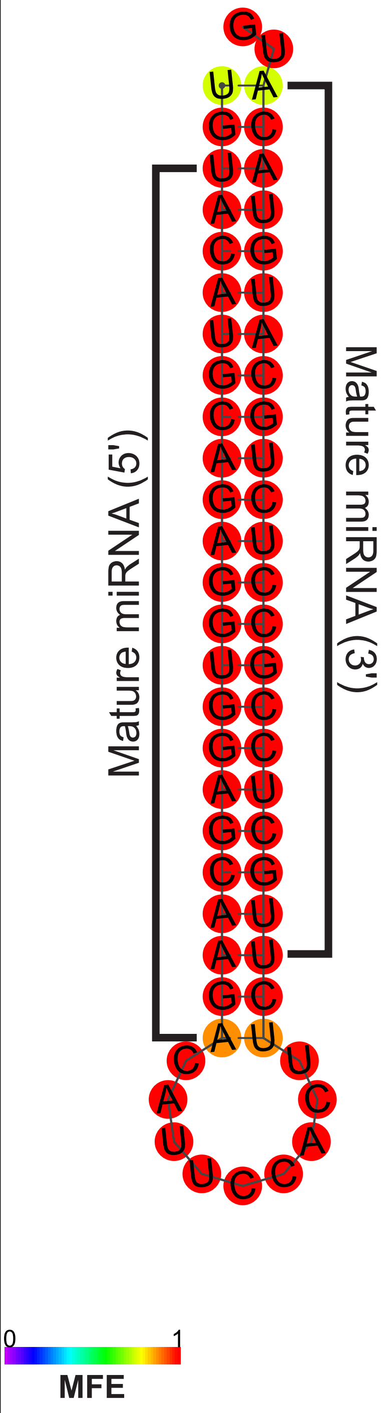

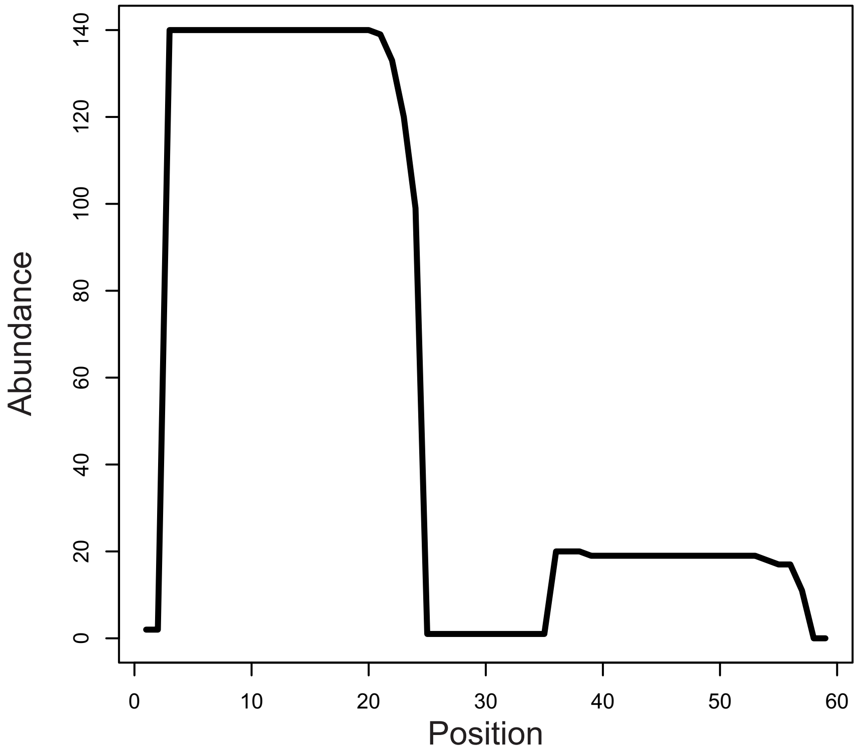


```
TGTACATGCAGAGGTGGAGCAAGACATTCCACTTCTTGCTCCGCCTCTGCATGTACATG       Raw reads
..TACATGCAGAGGTGGAGCAAGA...................................       98
..TACATGCAGAGGTGGAGCAAG....................................       21
..TACATGCAGAGGTGGAGCAA.....................................       12
...................................TTGCTCCGCCTCTGCATGTACA..       11
..TACATGCAGAGGTGGAGCA......................................       6
...................................TTGCTCCGCCTCTGCATGTAC...       6
..TACATGCAGAGGTGGAGC.......................................       1
TGTACATGCAGAGGTGGAGCA......................................       1
TGTACATGCAGAGGTGGAGCAA.....................................       1
.....................AGACATTCCACTTCTTG.....................       1
...................................TTGCTCCGCCTCTGCATG......       1
...................................TTGCTCCGCCTCTGCATGT.....       1

((((((((((((((((((((((((.........))))))))))))))))))))))))..       (-45.00 MFE
```

mir-n768

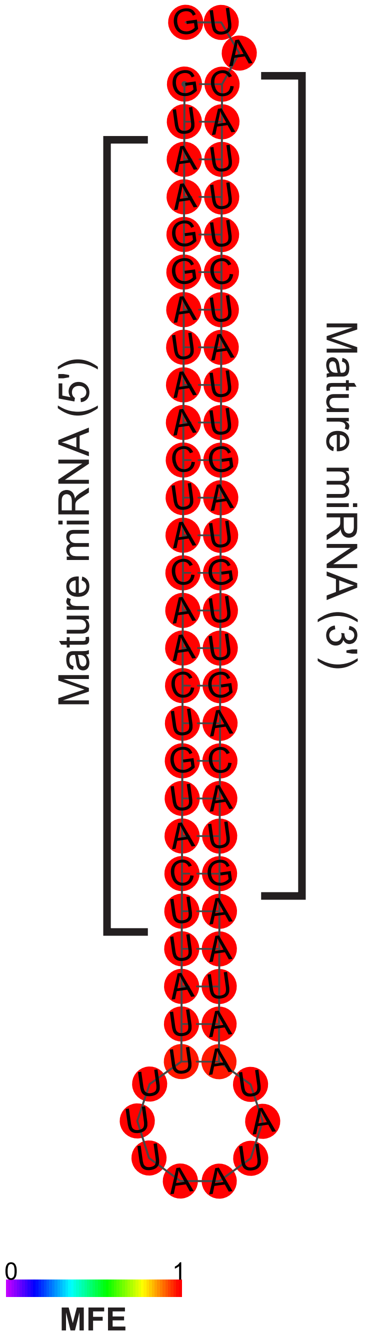

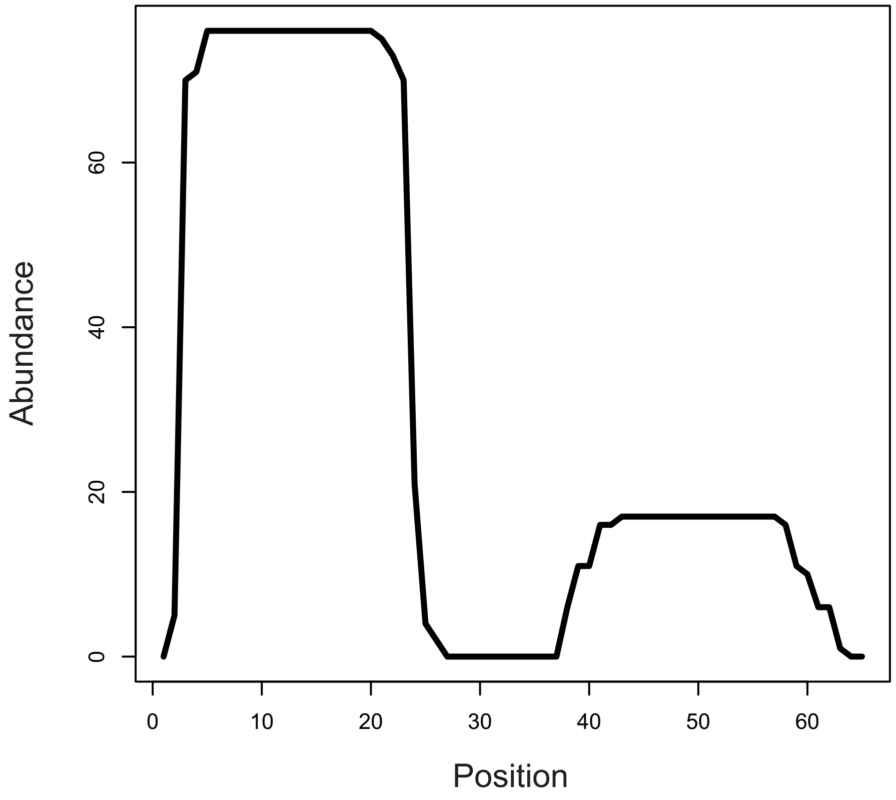


```
GTAAGGATAACTACAACTGTACTTATTTTTAATATAATAAGTACAGTTGTAGTTATCTTTACATG   Raw reads
..AAGGATAACTACAACTGTACT..........................................   44
..AAGGATAACTACAACTGTACTT.........................................   16
.TAAGGATAACTACAACTGTACT..........................................   5
........................................GTACAGTTGTAGTTATCTTTAC...   5
.....................................TAAGTACAGTTGTAGTTATCT.......   4
......................................AAGTACAGTTGTAGTTATCTTT.....   3
..AAGGATAACTACAACTGTA............................................   2
..AAGGATAACTACAACTGTAC...........................................   2
....GGATAACTACAACTGTACTTA........................................   2
....GGATAACTACAACTGTACTTAT.......................................   2
..AAGGATAACTACAACTGT.............................................   1
....GGATAACTACAACTGTAC...........................................   1
...AGGATAACTACAACTGTACTT.........................................   1
.....................................TAAGTACAGTTGTAGTTATC........   1
......................................AAGTACAGTTGTAGTTATCT.......   1
......................................AAGTACAGTTGTAGTTATCTT......   1
.....................................TAAGTACAGTTGTAGTTATCTTT.....   1
..........................................ACAGTTGTAGTTATCTTTACA..   1

(((((((((((((((((((((((((((........)))))))))))))))))))))))))))...   (-38.90 MFE)
```

mir-n771

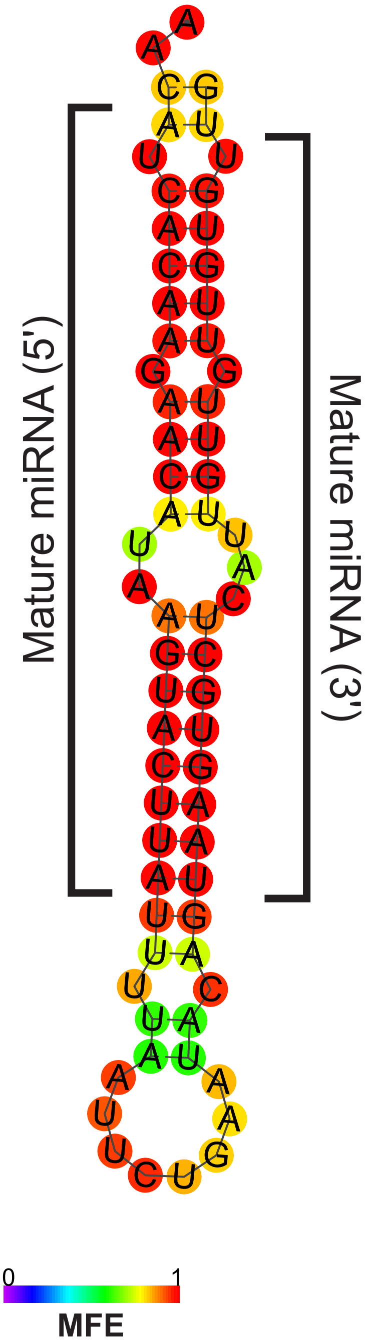

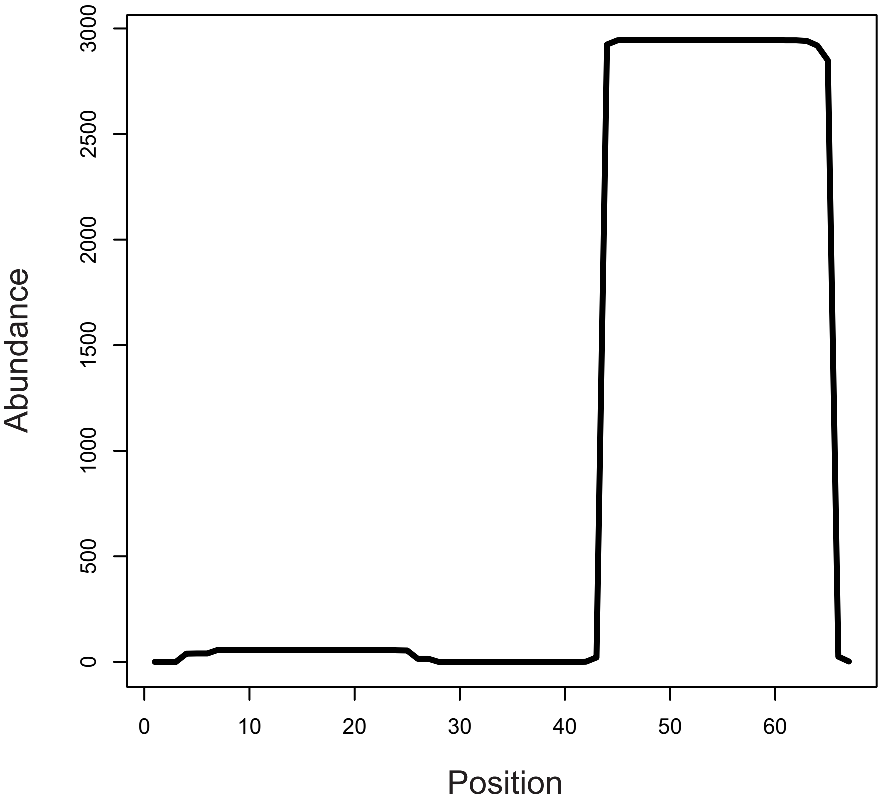


```
AACATCACAAGAACATAAGTACTTATTTTAATTCTGAATACAGTAAGTGCTCATTGTTGTTGTGTTG        Raw reads
...........................................TAAGTGCTCATTGTTGTTGTGT..        2785
...........................................TAAGTGCTCATTGTTGTTGTG...        70
...ATCACAAGAACATAAGTACTTA..........................................        36
...........................................TAAGTGCTCATTGTTGTTGTGTT.        23
...........................................TAAGTGCTCATTGTTGTTGT....        21
..........................................GTAAGTGCTCATTGTTGTTGTGT..        20
............................................AAGTGCTCATTGTTGTTGTGT..        18
......ACAAGAACATAAGTACTTATT........................................        15
...........................................TAAGTGCTCATTGTTGTTG.....        3
...ATCACAAGAACATAAGTACT............................................        2
......ACAAGAACATAAGTACTTA..........................................        2
...ATCACAAGAACATAAGTACTT...........................................        1
....TCACAAGAACATAAGTACTTA..........................................        1
...........................................TAAGTGCTCATTGTTGT.......        1
............................................AAGTGCTCATTGTTGTTGT....        1
.........................................AGTAAGTGCTCATTGTTGTTGTGT..        1
............................................AAGTGCTCATTGTTGTTGTGTTG        1
.............................................AGTGCTCATTGTTGTTGTGTTG        1

..((.(((((.((((..((((((((((.((........)).))))))))))...)))).))))).))        (-19.90 MFE)
```

mir-n780

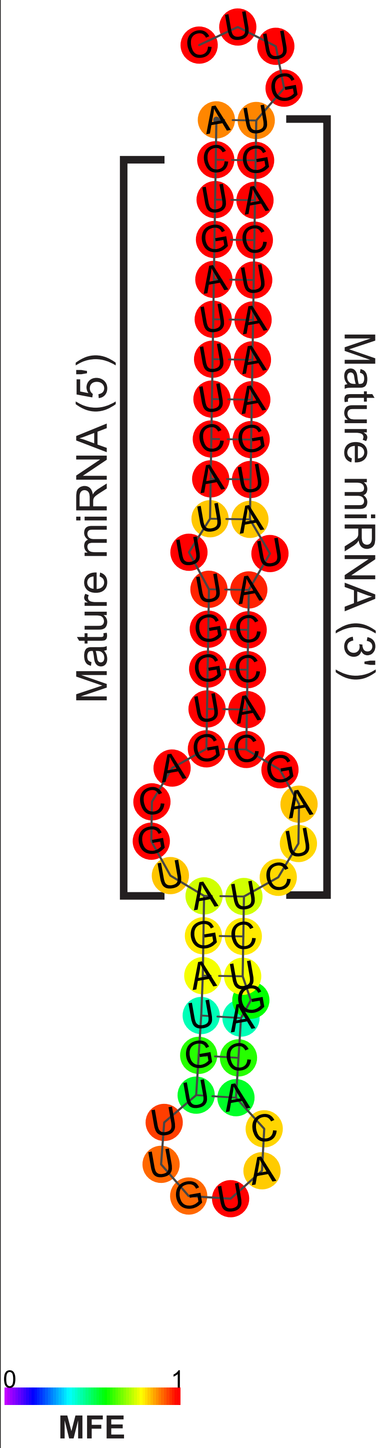

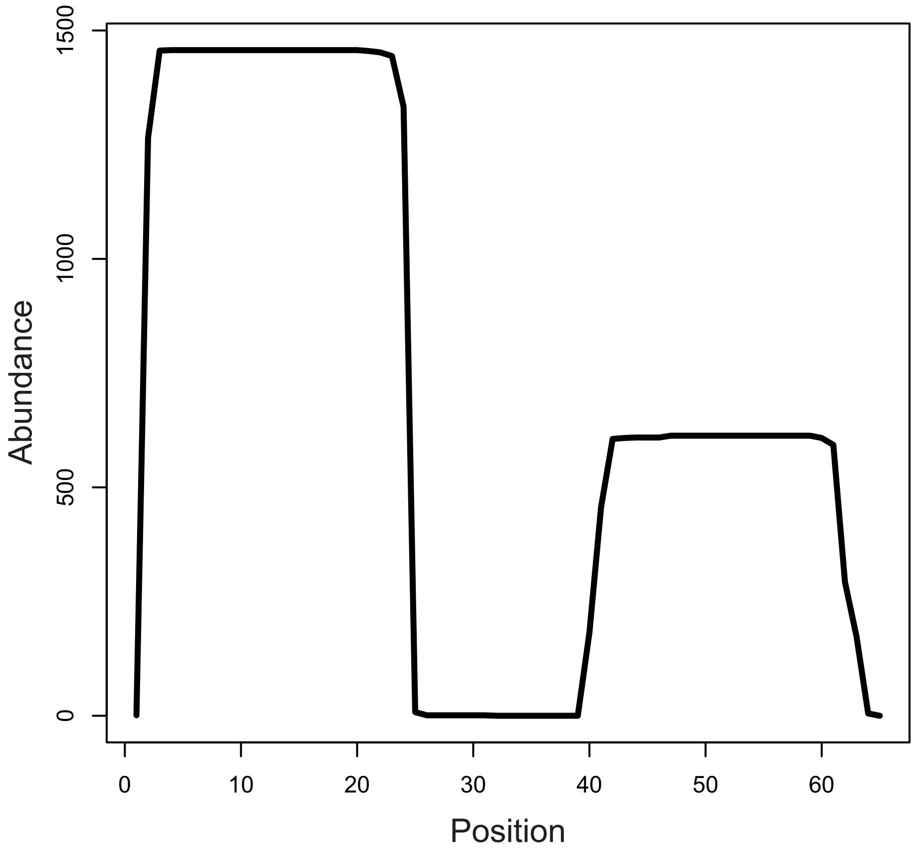


```
ACTGATTTCATTTGGTGACGTAGATGTTTGTACACAGTCTCTAGCACCATATGAAATCAGTGTTC Raw read
.CTGATTTCATTTGGTGACGTAGA......................................... 1138
..TGATTTCATTTGGTGACGTAGA......................................... 185
.......................................TCTAGCACCATATGAAATCAGT.... 157
........................................CTAGCACCATATGAAATCAGT.... 115
.CTGATTTCATTTGGTGACGTAG.......................................... 106
.........................................TAGCACCATATGAAATCAGTGT.. 94
........................................CTAGCACCATATGAAATCAGTG... 85
........................................CTAGCACCATATGAAATCAGTGT.. 64
.........................................TAGCACCATATGAAATCAGT.... 28
.........................................TAGCACCATATGAAATCAGTG... 20
.......................................TCTAGCACCATATGAAATCAGTG... 13
.CTGATTTCATTTGGTGACGTA........................................... 7
.CTGATTTCATTTGGTGACGTAGAT........................................ 7
.......................................TCTAGCACCATATGAAATCAGTGT.. 6
..TGATTTCATTTGGTGACGTAG.......................................... 5
........................................CTAGCACCATATGAAATCAG..... 5
.........................................TAGCACCATATGAAATCAG..... 5
........................................CTAGCACCATATGAAATCA...... 4
.......................................TCTAGCACCATATGAAATCAG..... 4
.CTGATTTCATTTGGTGACGT............................................ 3
.........................................TAGCACCATATGAAATCAGTGTT. 3
.CTGATTTCATTTGGTGACG............................................. 2
..............................................CCATATGAAATCAGTG... 2
..............................................CCATATGAAATCAGTGT.. 2
..TGATTTCATTTGGTGACGTA........................................... 1
ACTGATTTCATTTGGTGACGTAGA......................................... 1
...GATTTCATTTGGTGACGTAGA......................................... 1
.CTGATTTCATTTGGTGACGTAGATGTTTGT.................................. 1
.......................................TCTAGCACCATATGAAATCA...... 1
..........................................AGCACCATATGAAATCAG..... 1
..........................................AGCACCATATGAAATCAGTGT.. 1
...........................................GCACCATATGAAATCAGTGT.. 1
.......................................TCTAGCACCATATGAAATCAGTGTT. 1
........................................CTAGCACCATATGAAATCAGTGTT. 1

(((((((((((.(((((.(.((((.(.(((....))).))))))))))).)))))))))))).... (-25.60 MFE)
```

mir-n791

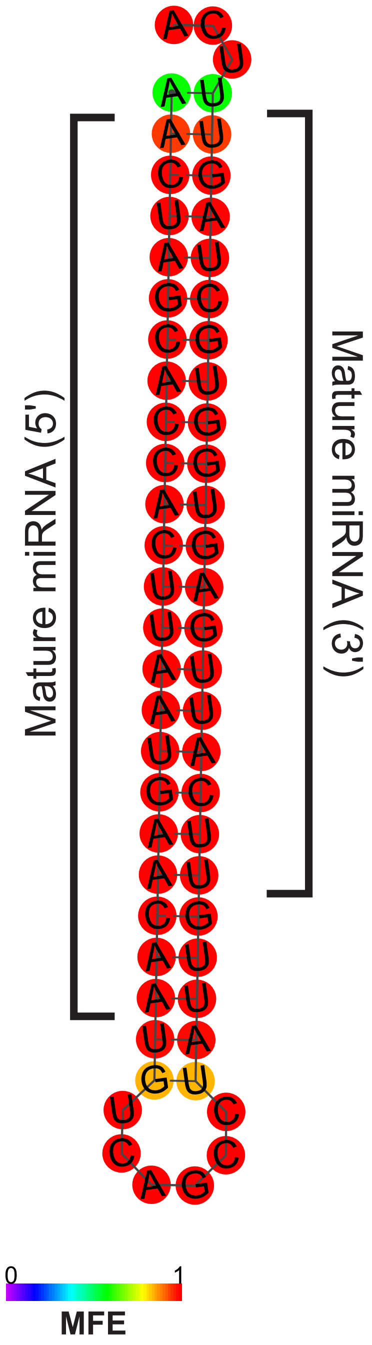

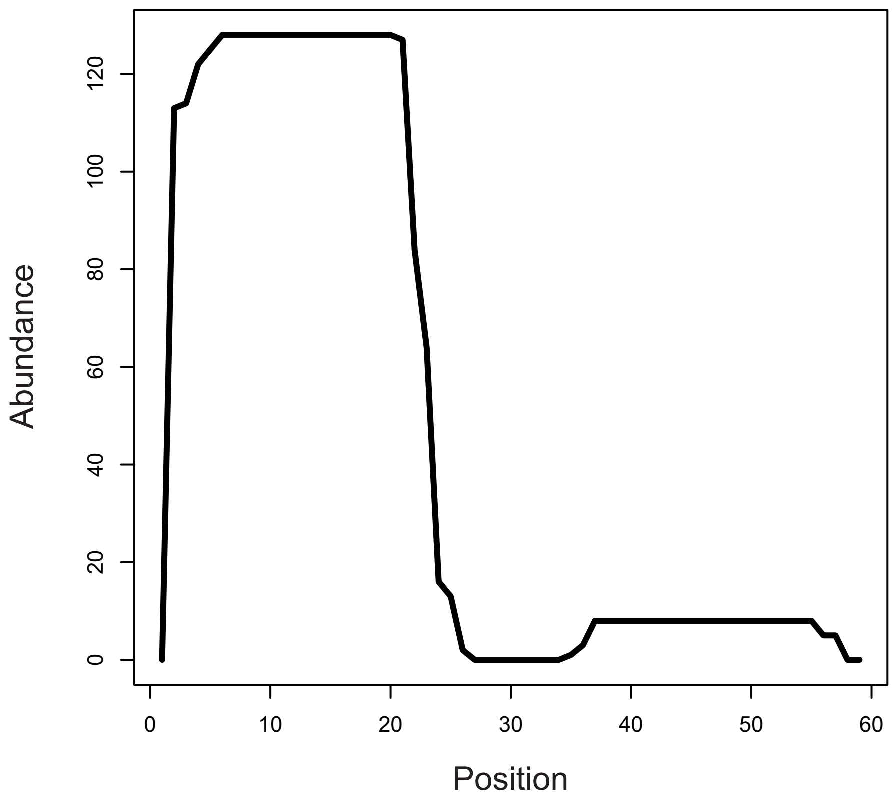


```
AACTAGCACCACTTAATGAACAATGTCAGCCTATTGTTCATTGAGTGGTGCTAGTTTCA      Raw reads
.ACTAGCACCACTTAATGAACAA....................................      48
.ACTAGCACCACTTAATGAAC......................................      42
.ACTAGCACCACTTAATGAACA.....................................      20
...TAGCACCACTTAATGAACAATG..................................      7
.....GCACCACTTAATGAACAATG..................................      3
....................................TTCATTGAGTGGTGCTAGT....      3
.ACTAGCACCACTTAATGAACAAT...................................      2
....AGCACCACTTAATGAACAATGT.................................      2
...................................GTTCATTGAGTGGTGCTAGTTT..      2
....................................TTCATTGAGTGGTGCTAGTTT..      2
.ACTAGCACCACTTAATGAA.......................................      1
..CTAGCACCACTTAATGAAC......................................      1
...TAGCACCACTTAATGAACAAT...................................      1
....AGCACCACTTAATGAACAATG..................................      1
..................................TGTTCATTGAGTGGTGCTAGTTT..      1

(((((((((((((((((((((((((......)))))))))))))))))))))))))...      (-37.50 MFE)
```

mir-n813-2

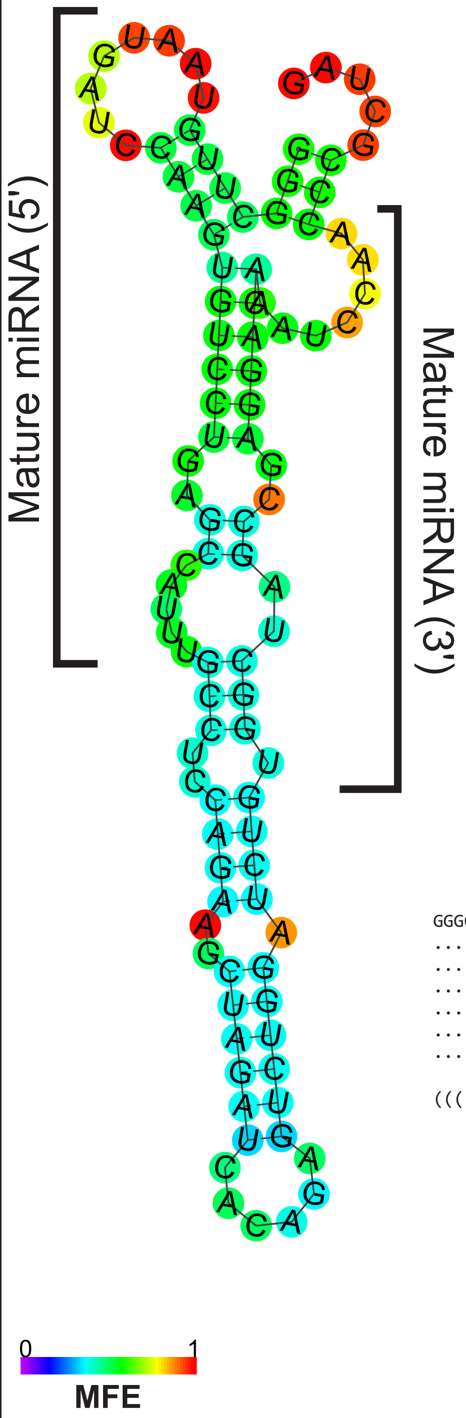

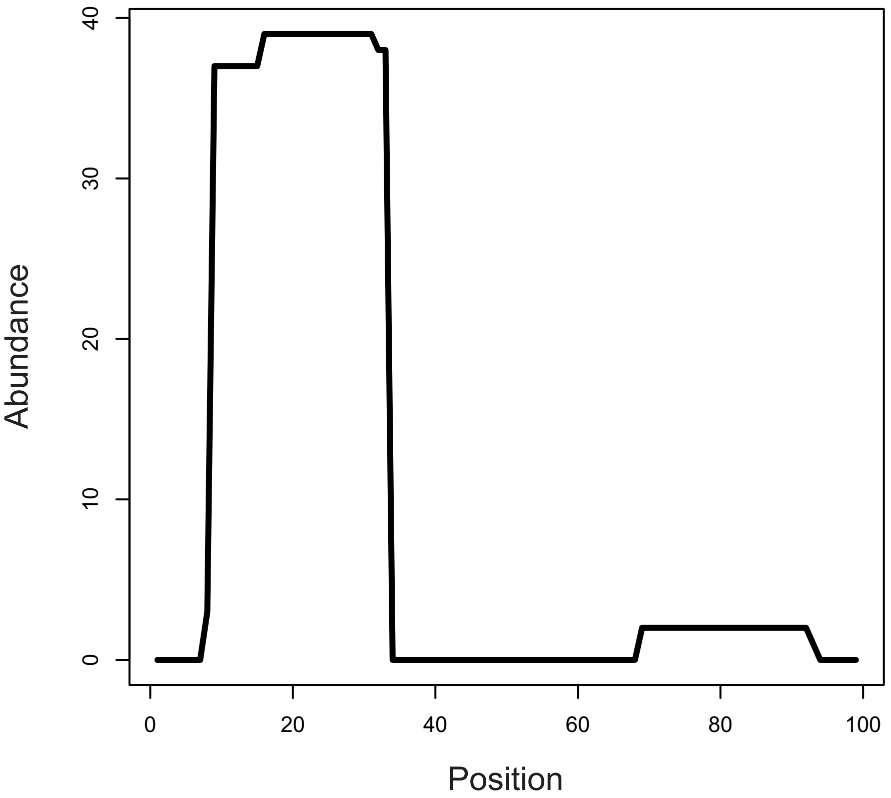


```
GGGCTTGTAATGATCCAAGTGTCCTGAGCCATTTGCCTCCAGAAGCTAGATCACAGAGTCTGGATCTGTGGCTAGCCGAGGACAAATCCAACCCGCTAG    Raw reads
........AATGATCCAAGTGTCCTGAGCCATT..................................................................    33
.......TAATGATCCAAGTGTCCTGAGCCATT..................................................................    3
...............CAAGTGTCCTGAGCCATT..................................................................    2
........AATGATCCAAGTGTCCTGAGCCA....................................................................    1
....................................................................TGGCTAGCCGAGGACAAATCCAAC.......    1
....................................................................TGGCTAGCCGAGGACAAATCCAACC......    1

(((.(((....(((.....((((((.((((((.....((((((..((.......))..))))))...)))))).....)))))).))))))))).....  (-30.70 MFE)
```

mir-n836

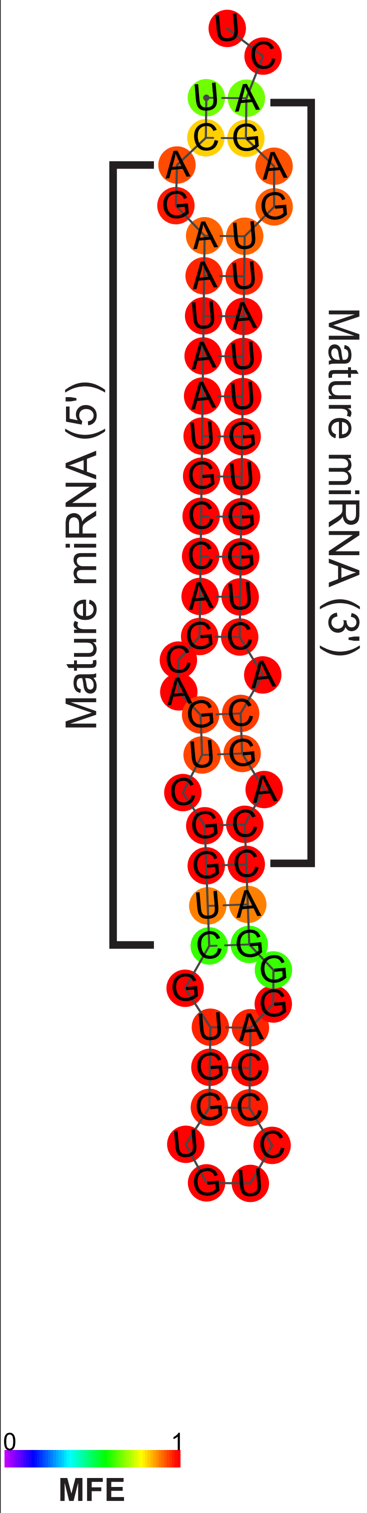

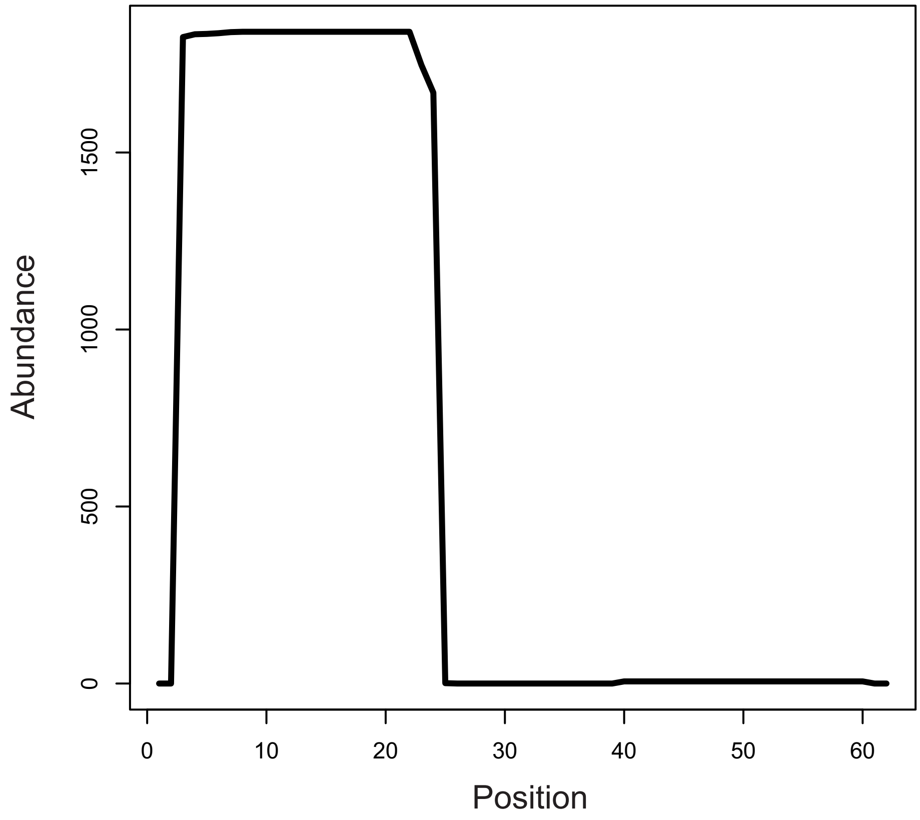


```
TCAGAATAATGCCAGCAGTCGGTCGTGGTGTCCCAGGGACCAGCACTGGTGTTATTGAGACT    Raw reads
..AGAATAATGCCAGCAGTCGGTC......................................    1656
..AGAATAATGCCAGCAGTCGG........................................    93
..AGAATAATGCCAGCAGTCGGT.......................................    76
...GAATAATGCCAGCAGTCGGTC......................................    6
.......................................CCAGCACTGGTGTTATTGAGA..    6
.....ATAATGCCAGCAGTCGGTC......................................    2
......TAATGCCAGCAGTCGGTC......................................    2
...GAATAATGCCAGCAGTCGG........................................    1
...GAATAATGCCAGCAGTCGGT.......................................    1
......TAATGCCAGCAGTCGGT.......................................    1
....AATAATGCCAGCAGTCGGTC......................................    1
.......AATGCCAGCAGTCGGTC......................................    1
..AGAATAATGCCAGCAGTCGGTCG.....................................    1

((..(((((((((((..((.((((.(((....))).).))).)).)))))))))))..))..    (-21.50 MFE)
```

mir-n843

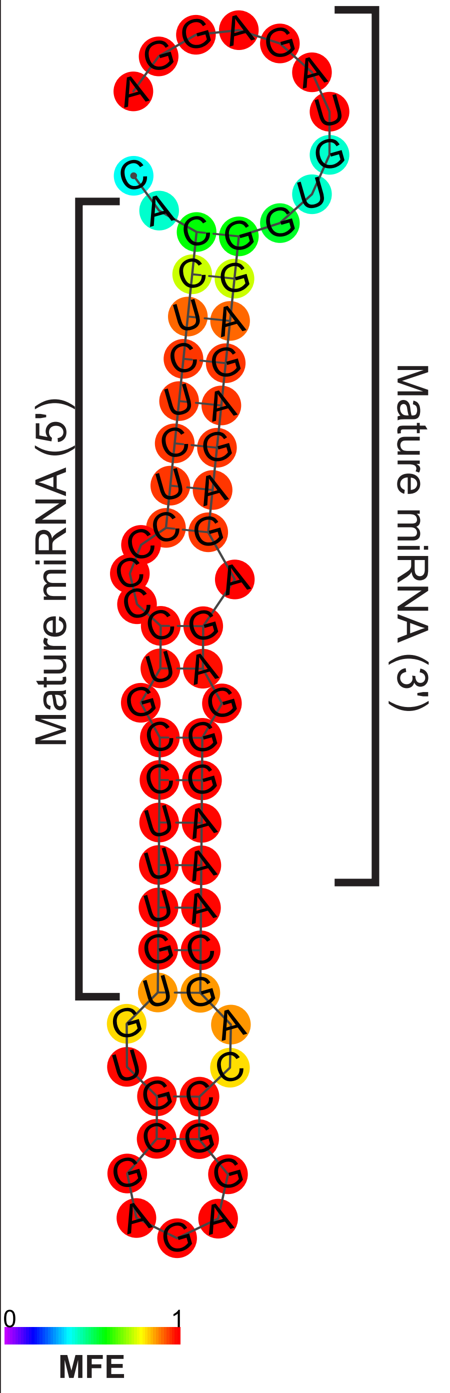

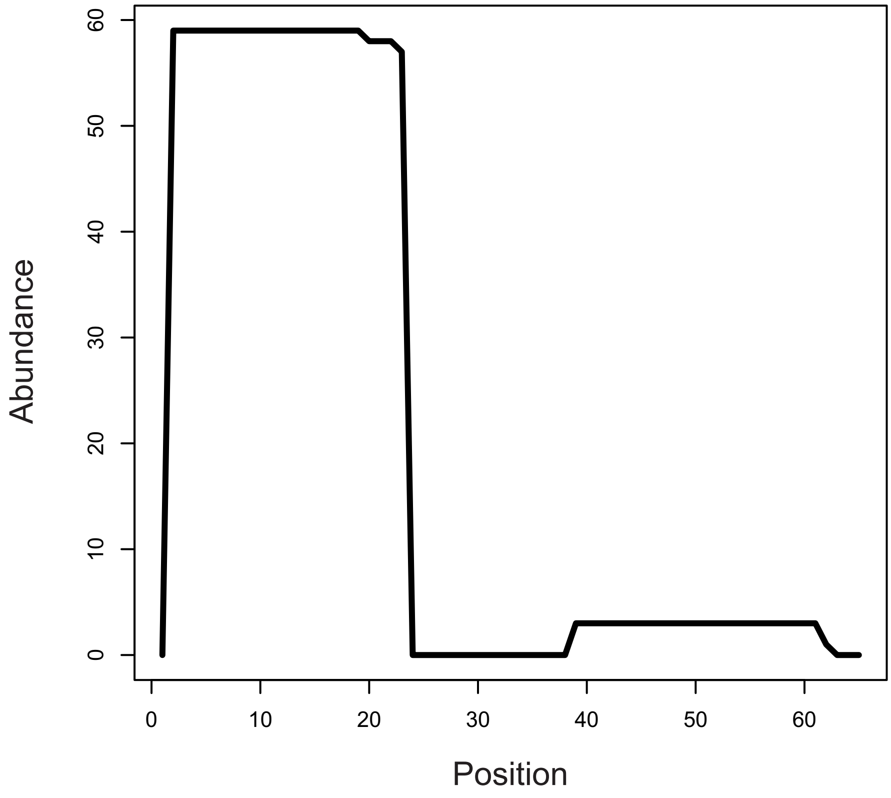


```
CACCTCTCTCCCCCTGCCTTTGTGTGCGAGAGGCCAGCAAAGGGAGAGAGAGAGGGTGTAGAGGA   Raw reads
.ACCTCTCTCCCCCTGCCTTTGT..........................................   57
......................................AAAGGGAGAGAGAGAGGGTGTAG....   2
.ACCTCTCTCCCCCTGCCT..............................................   1
.ACCTCTCTCCCCCTGCCTTTG...........................................   1
......................................AAAGGGAGAGAGAGAGGGTGTAGA...   1

((((.(((((.(.((.(((((((..((.....))..))))))).)).).))))))))).......   (-28.40 MFE)
```

mir-n847

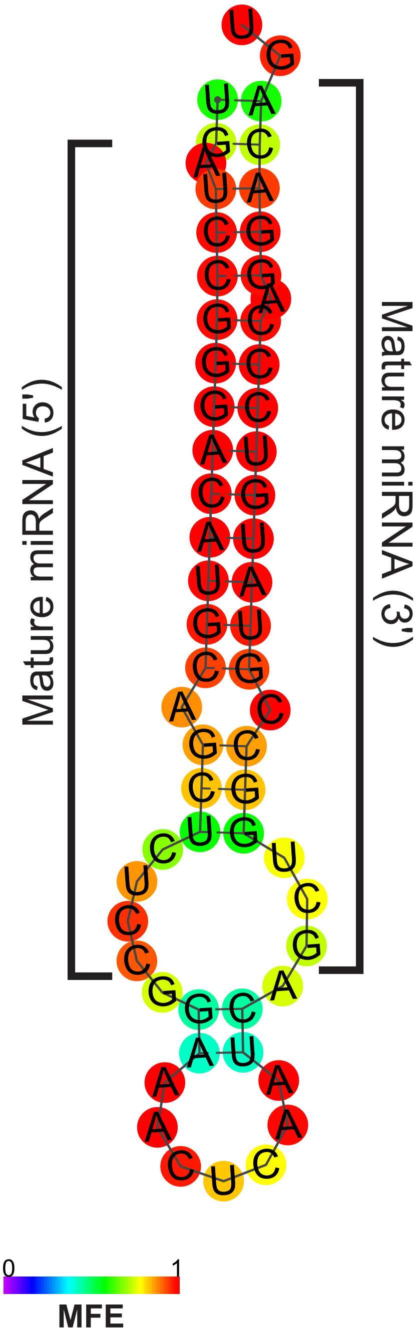

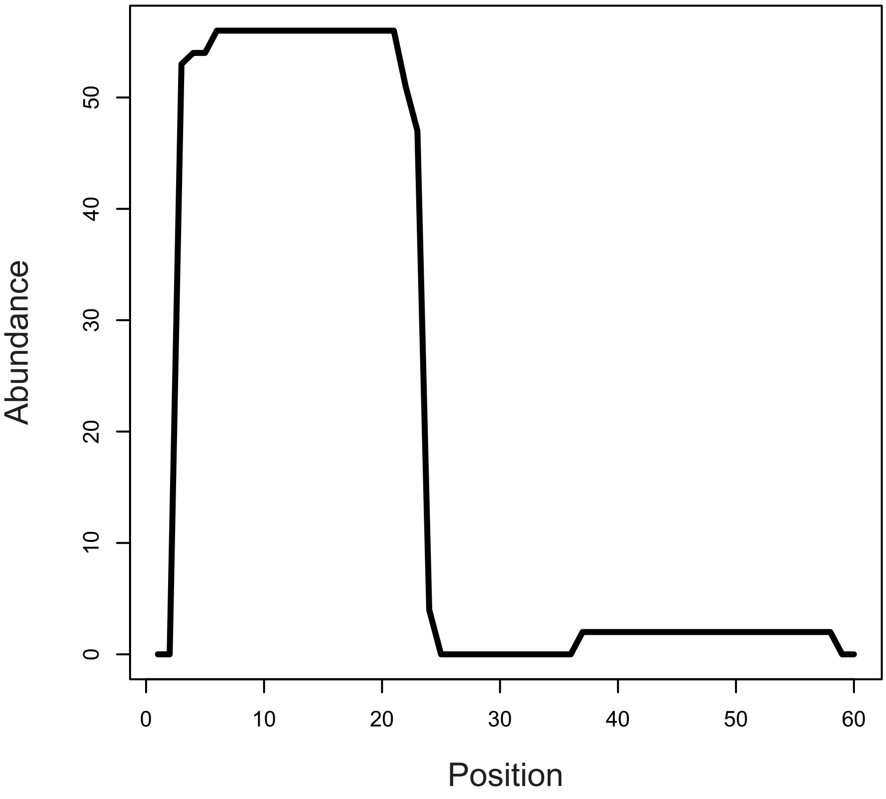


```
TGATCCGGGACATGCAGCTCTCCGGAAACTCAATCAGCTGGCCGTATGTCCCAGGACAGT      Raw reads
..ATCCGGGACATGCAGCTCTCC.....................................      41
..ATCCGGGACATGCAGCTCT.......................................      5
..ATCCGGGACATGCAGCTCTC......................................      4
..ATCCGGGACATGCAGCTCTCCG....................................      3
....................................GCTGGCCGTATGTCCCAGGACA..      2
...TCCGGGACATGCAGCTCTCC.....................................      1
.....CGGGACATGCAGCTCTCC.....................................      1
.....CGGGACATGCAGCTCTCCG....................................      1

((.((((((((((((.(((.....((.......))....))).))))))))).)))))..     (-22.40 MFE)
```

mir-n873

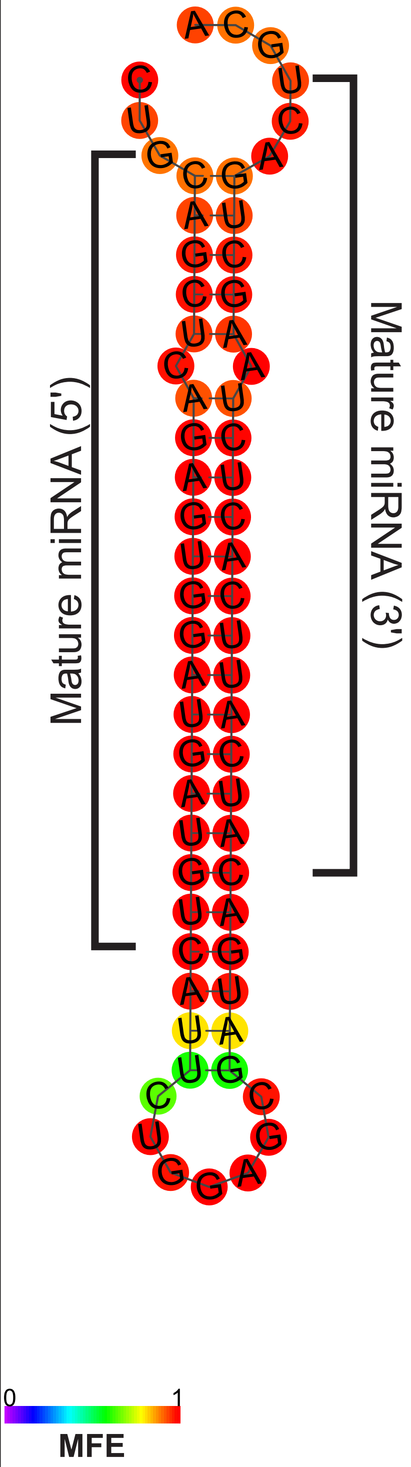

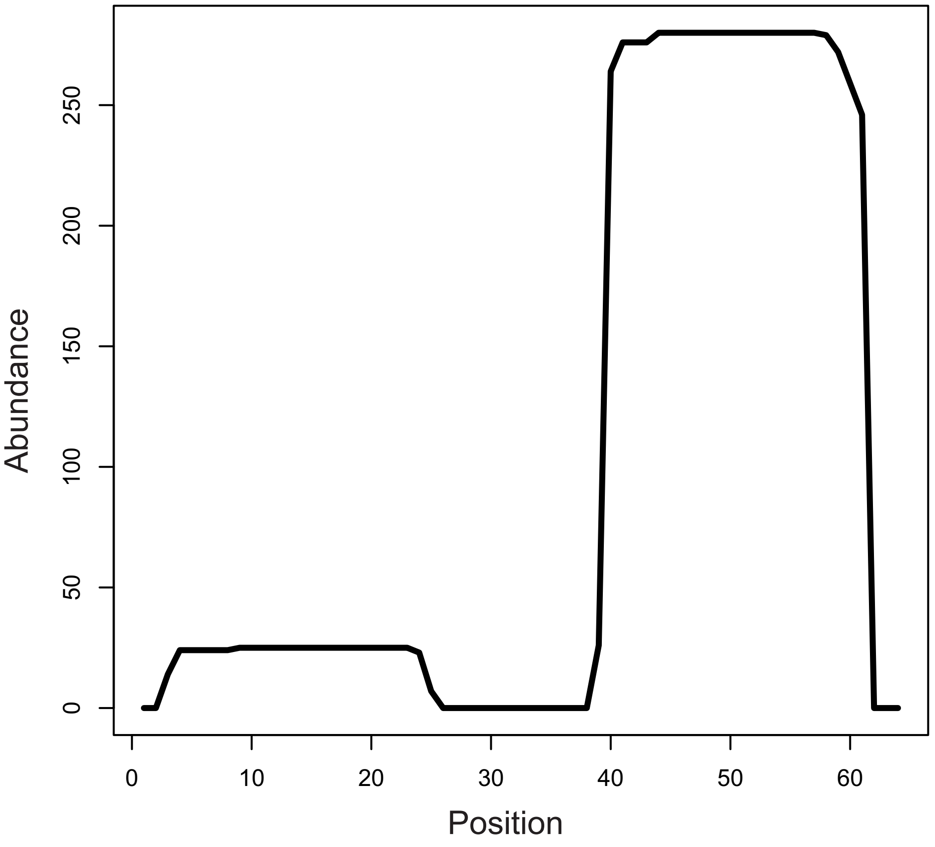


```
CTGCAGCTCAGAGTGGATGATGTCATTCTGGAGCGATGACATCATTCACTCTAAGCTGACTGCA  Raw reads
.......................................CATCATTCACTCTAAGCTGACT...  223
........................................ATCATTCACTCTAAGCTGACT...  11
..GCAGCTCAGAGTGGATGATGTC........................................  10
.......................................CATCATTCACTCTAAGCTGA.....  10
......................................ACATCATTCACTCTAAGCTGACT...  9
......................................ACATCATTCACTCTAAGCTG......  7
......................................ACATCATTCACTCTAAGCTGAC....  7
...CAGCTCAGAGTGGATGATGTC........................................  6
.......................................CATCATTCACTCTAAGCTGAC....  5
...CAGCTCAGAGTGGATGATGTCA.......................................  4
...........................................ATTCACTCTAAGCTGACT...  3
..GCAGCTCAGAGTGGATGATGT.........................................  2
..GCAGCTCAGAGTGGATGATGTCA.......................................  2
......................................ACATCATTCACTCTAAGCTGA.....  2
........CAGAGTGGATGATGTCA.......................................  1
......................................ACATCATTCACTCTAAGCT.......  1
...........................................ATTCACTCTAAGCTGA.....  1
........................................ATCATTCACTCTAAGCTGAC....  1

...(((((.((((((((((((((((((.......)))))))))))))))))).)))))...... (-35.10 MFE)
```

mir-n875

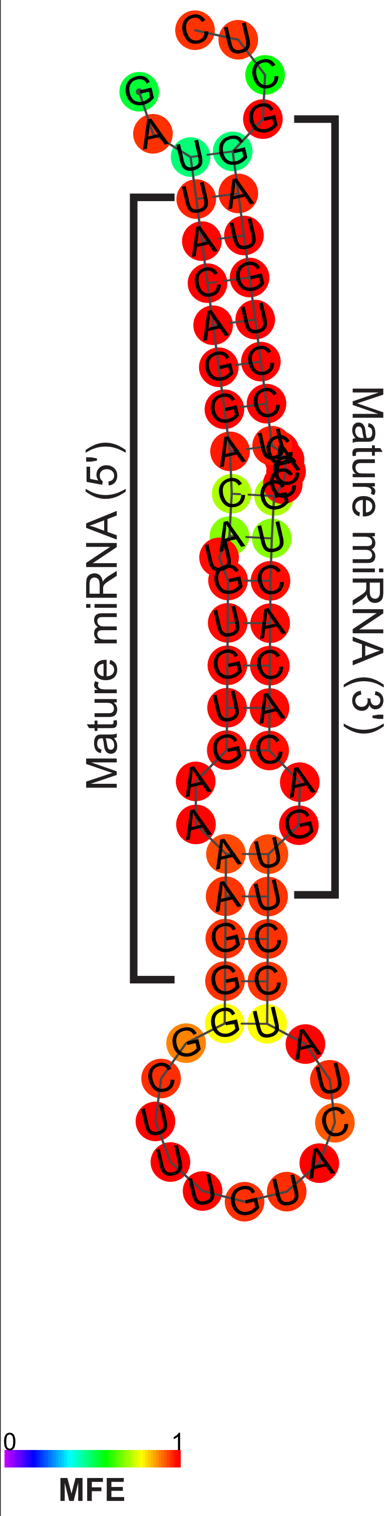

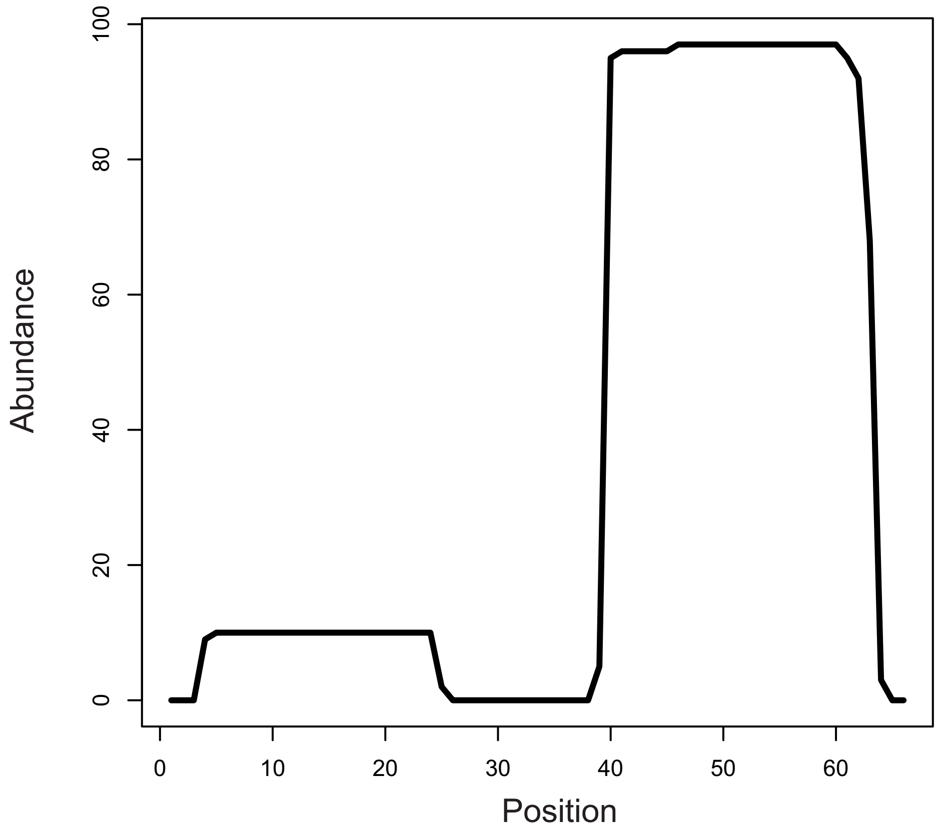


```
GATTACAGGACATGTGTGAAAAGGGGCTTTGTACTATCCTTGACACACTGCCACTCCTGTAGGCTC        Raw Read
.......................................TTGACACACTGCCACTCCTGTAGG...       61
.......................................TTGACACACTGCCACTCCTGTAG....       22
...TACAGGACATGTGTGAAAAGG............................................       7
......................................CTTGACACACTGCCACTCCTGTAGG...       4
.......................................TTGACACACTGCCACTCCTGTA.....       3
.......................................TTGACACACTGCCACTCCTGTAGGC..       3
...TACAGGACATGTGTGAAAAGGG...........................................       2
....ACAGGACATGTGTGAAAAGG............................................       1
.......................................TTGACACACTGCCACTCCTGT......       1
........................................TGACACACTGCCACTCCTGT......       1
......................................CTTGACACACTGCCACTCCTGTAG....       1
.............................................CACTGCCACTCCTGTAG....       1

..((((((((((.(((((..(((((...........)))))..)))))))....)))))))).... (-20.50 MFE)
```

mir-n878

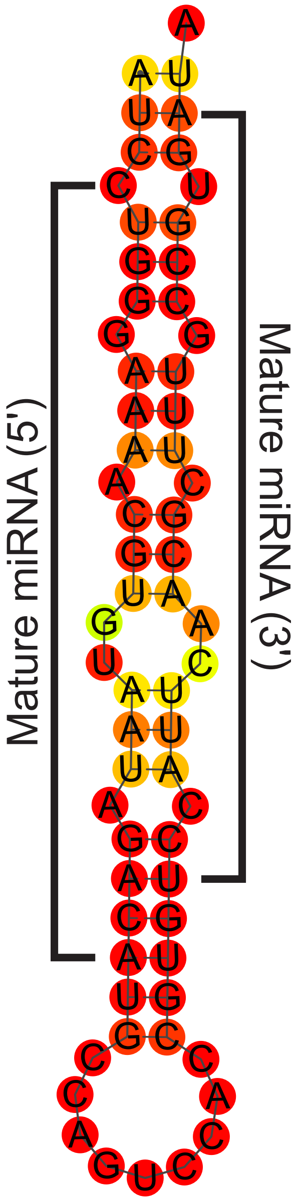

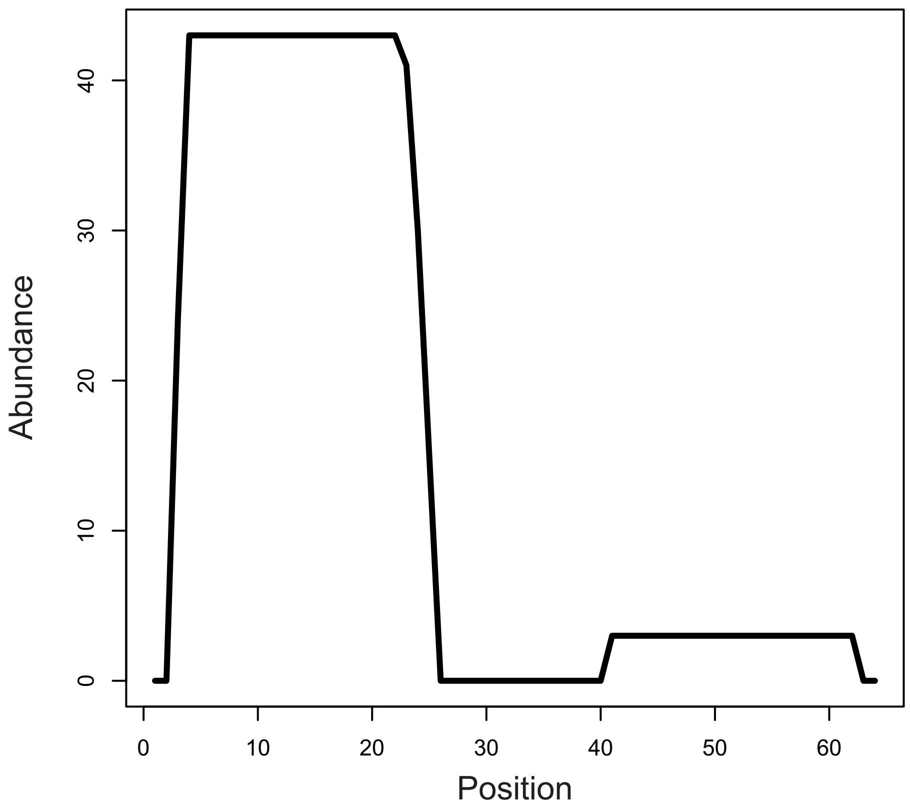


```
ATCCTGGGAAAACGTGTAATAGACATGCCAGTCCACCGTGTCCATTCAACGCTTTGCCGTGATA  Raw reads
...CTGGGAAAACGTGTAATAGACA.....................................  13
..CCTGGGAAAACGTGTAATAGAC......................................  12
..CCTGGGAAAACGTGTAATAGA.......................................  8
...CTGGGAAAACGTGTAATAGA.......................................  3
...CTGGGAAAACGTGTAATAGAC......................................  3
.....................................TCCATTCAACGCTTTGCCGTGA..  3
..CCTGGGAAAACGTGTAATAG........................................  2
..CCTGGGAAAACGTGTAATAGACA.....................................  2

(((.(((.(((.(((..(((.((((((.........)))))).)))..))).))).))).))). (-15.90 MFE)
```

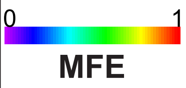

mir-n893

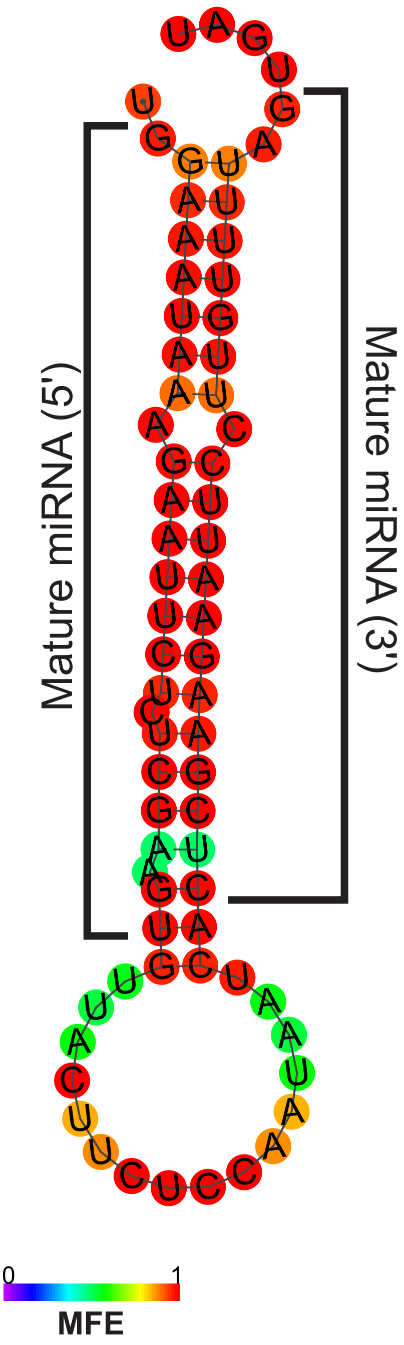

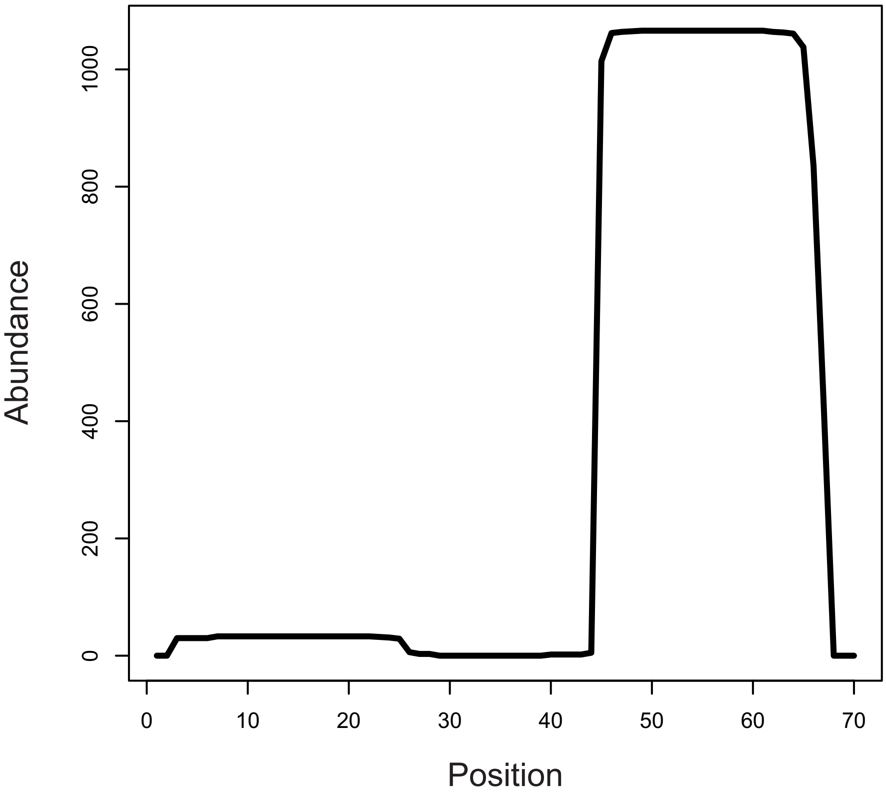


```
TGGAAATAAAGAATTCTCTCGAAGTGTTACTTCTCCAATAATCACTCGAAGAATTCCTTGTTTTAGTGAT    Raw reads
............................................CTCGAAGAATTCCTTGTTTTAG....    395
............................................CTCGAAGAATTCCTTGTTTTAGT...    394
............................................CTCGAAGAATTCCTTGTTTTA.....    197
.............................................TCGAAGAATTCCTTGTTTTAGT...    34
..GAAATAAAGAATTCTCTCGAAGT.............................................    23
............................................CTCGAAGAATTCCTTGTTTT......    21
.............................................TCGAAGAATTCCTTGTTTTAG....    10
..GAAATAAAGAATTCTCTCGAAGTG............................................    3
......TAAAGAATTCTCTCGAAGTGTT..........................................    3
.............................................TCGAAGAATTCCTTGTTTTA.....    3
..GAAATAAAGAATTCTCTCGAAG..............................................    2
.......................................AATCACTCGAAGAATTCCTTGT.........    2
...........................................ACTCGAAGAATTCCTTGTTTTA.....    2
..............................................CGAAGAATTCCTTGTTTTAGT...    2
..GAAATAAAGAATTCTCTCGA................................................    1
..GAAATAAAGAATTCTCTCGAA...............................................    1
............................................CTCGAAGAATTCCTTGTT........    1
............................................CTCGAAGAATTCCTTGTTT.......    1
.............................................TCGAAGAATTCCTTGTTT.......    1
...........................................ACTCGAAGAATTCCTTGTTTT......    1
...............................................GAAGAATTCCTTGTTTT......    1
................................................AAGAATTCCTTGTTTTA.....    1

..(((((((.(((((((.((((.(((................))))))))))))))).)))))))......   (-17.09 MFE)
```

mir-n896

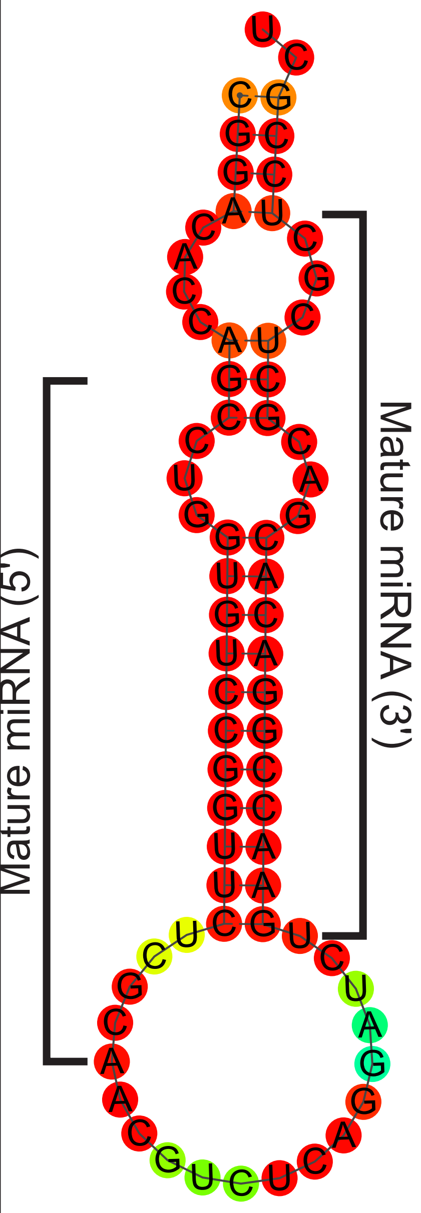

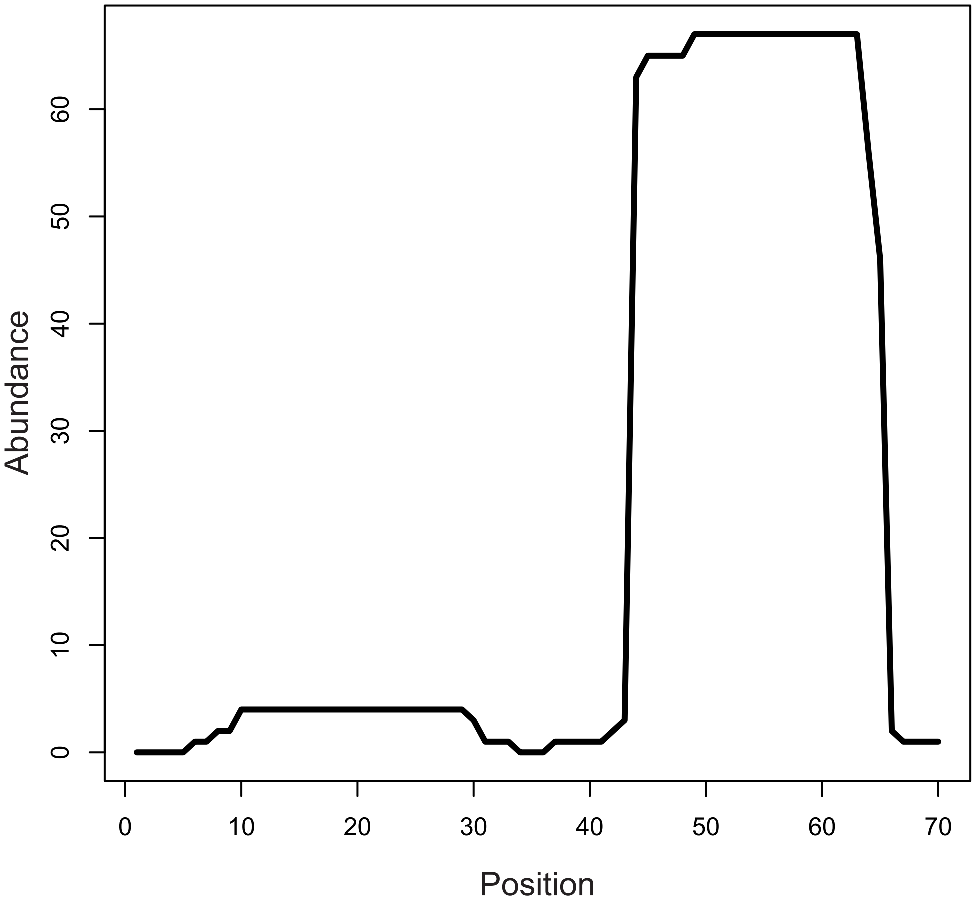


```
CGGACACCAGCCTGGTGTCCGGTTCTCGCAACGTCTCAGGATCTGAACCGGACACGACGCTCGCTCCGCT    Raw reads
...........................................TGAACCGGACACGACGCTCGCT.....    42
...........................................TGAACCGGACACGACGCTCG.......    10
...........................................TGAACCGGACACGACGCTCGC......    8
.........GCCTGGTGTCCGGTTCTCGCA........................................    2
................................................CGGACACGACGCTCGC......    2
.......CAGCCTGGTGTCCGGTTCTCGC.........................................    1
.....ACCAGCCTGGTGTCCGGTTCTCGCAACG.....................................    1
..........................................CTGAACCGGACACGACGCTCG.......    1
.........................................TCTGAACCGGACACGACGCTCGCT.....    1
............................................GAACCGGACACGACGCTCGCT.....    1
............................................GAACCGGACACGACGCTCGCTC....    1
....................................CAGGATCTGAACCGGACACGACGCTCGCTCCGCT    1

((((..(.(((.(.(((((((((((......(......).....))))))))))).).))).).)))).. (-28.70 MFE)
```

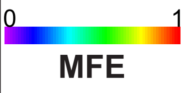

mir-n904

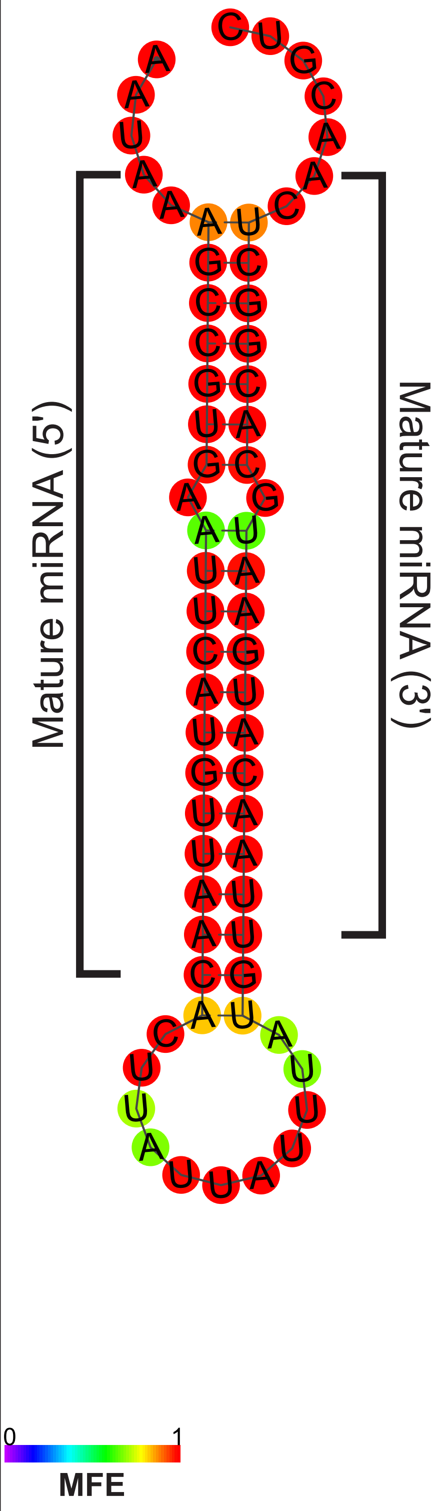

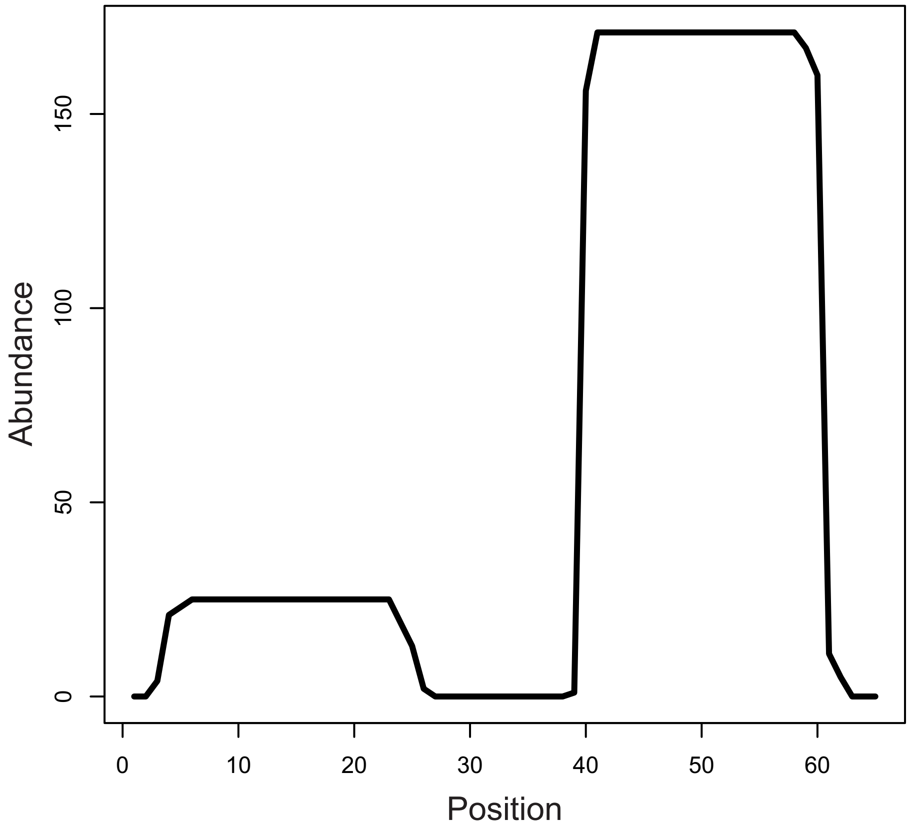


```
AATAAAGCCGTGAATTCATGTTAACACTTATTATTTATGTTAACATGAATGCACGGCTCAACGTC Raw reads
.......................................TTAACATGAATGCACGGCTCA..... 138
........................................TAACATGAATGCACGGCTCA..... 10
...AAAGCCGTGAATTCATGTTAAC........................................ 7
.......................................TTAACATGAATGCACGGCTC...... 7
...AAAGCCGTGAATTCATGTTA.......................................... 5
...AAAGCCGTGAATTCATGTTAA......................................... 5
.......................................TTAACATGAATGCACGGCT....... 4
.......................................TTAACATGAATGCACGGCTCAA.... 4
........................................TAACATGAATGCACGGCTCAAC... 3
..TAAAGCCGTGAATTCATGTTAAC........................................ 2
........................................TAACATGAATGCACGGCTCAA.... 2
.......................................TTAACATGAATGCACGGCTCAAC... 2
..TAAAGCCGTGAATTCATGTTA.......................................... 1
..TAAAGCCGTGAATTCATGTTAA......................................... 1
....AAGCCGTGAATTCATGTTAAC........................................ 1
.....AGCCGTGAATTCATGTTAAC........................................ 1
....AAGCCGTGAATTCATGTTAACA....................................... 1
.....AGCCGTGAATTCATGTTAACA....................................... 1
......................................GTTAACATGAATGCACGGCTCA..... 1

.....(((((((.(((((((((((((...........))))))))))))).))))))))....... (-27.10 MFE)
```

mir-n907

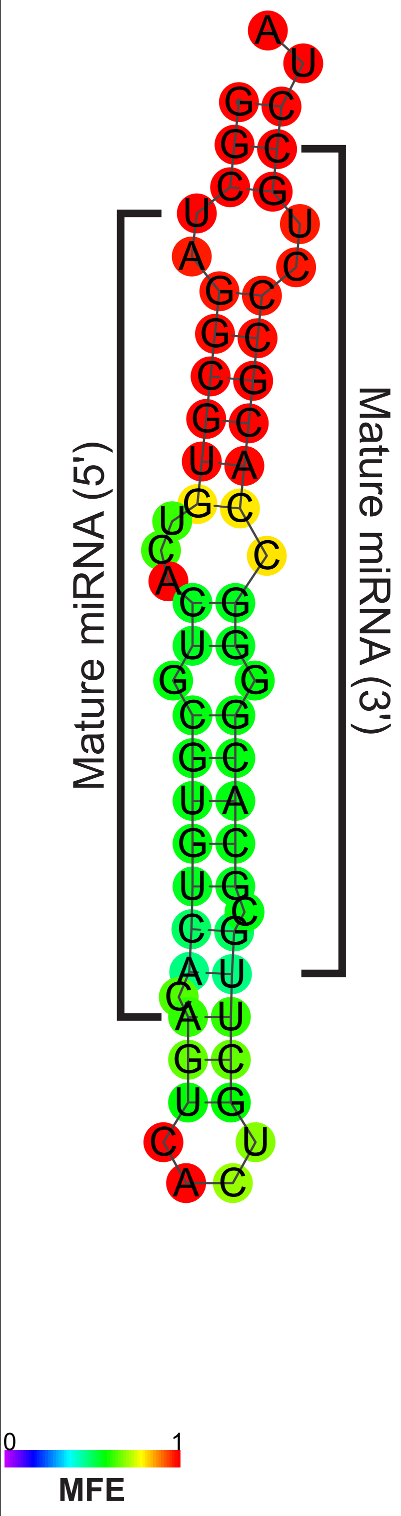

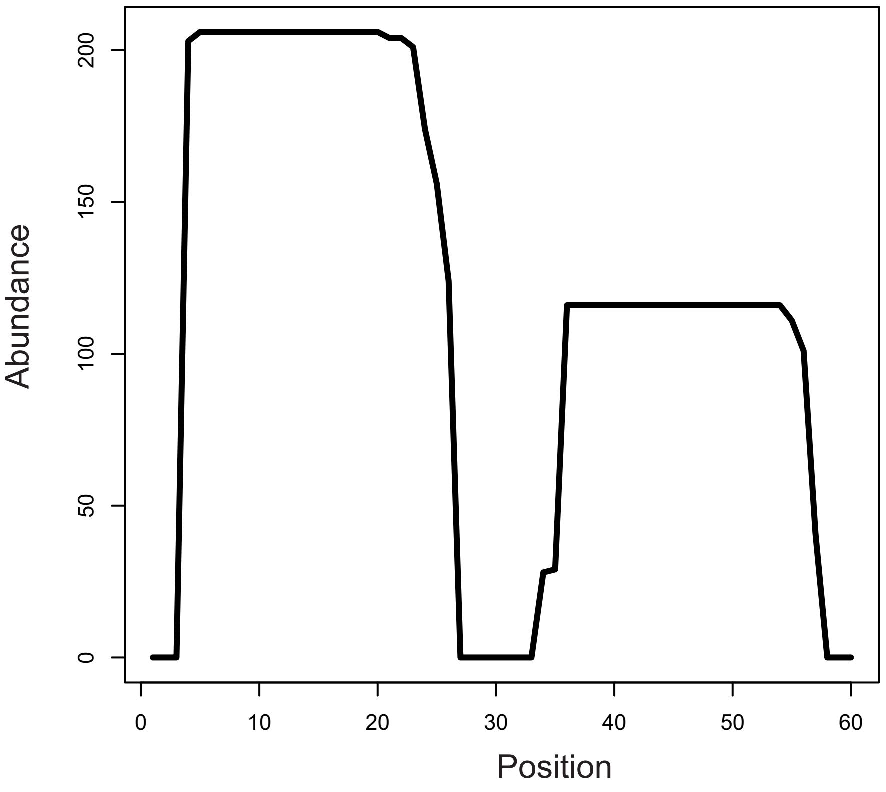


```
GGCTAGGCGTGTCACTGCGTGTCACAGTCACTGCTTGCGCACGGGGCCACGCCCTGCCTA      Raw reads
...TAGGCGTGTCACTGCGTGTCACA..................................      123
...................................TGCGCACGGGGCCACGCCCTGC...      39
...................................TGCGCACGGGGCCACGCCCTG....      38
...TAGGCGTGTCACTGCGTGTCAC...................................      32
...TAGGCGTGTCACTGCGTGTC.....................................      26
.................................CTTGCGCACGGGGCCACGCCCTG....      22
...TAGGCGTGTCACTGCGTGTCA....................................      17
...................................TGCGCACGGGGCCACGCCCT.....      6
...................................TGCGCACGGGGCCACGCCC......      4
...TAGGCGTGTCACTGCGTGT......................................      3
.................................CTTGCGCACGGGGCCACGCCCT.....      3
...TAGGCGTGTCACTGCGT........................................      2
.................................CTTGCGCACGGGGCCACGCCCTGC...      2
....AGGCGTGTCACTGCGTGTC.....................................      1
....AGGCGTGTCACTGCGTGTCA....................................      1
....AGGCGTGTCACTGCGTGTCACA..................................      1
.................................CTTGCGCACGGGGCCACGCCC......      1
..................................TTGCGCACGGGGCCACGCCCT.....      1

(((..((((((((.(((.((((..(((...)))...)))).))))).))))))..)))..      (-26.50 MFE)
```

mir-n932

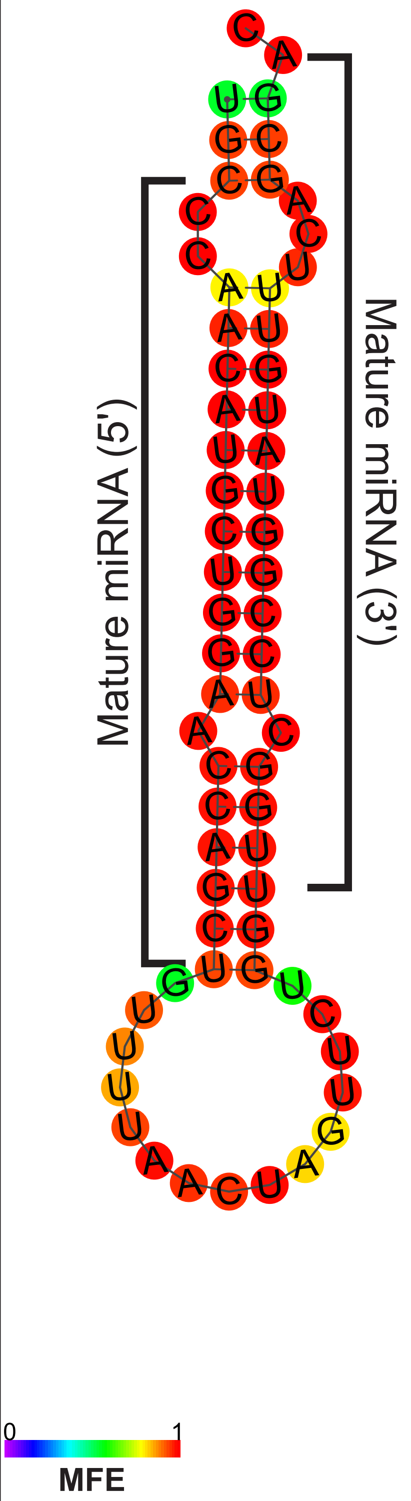

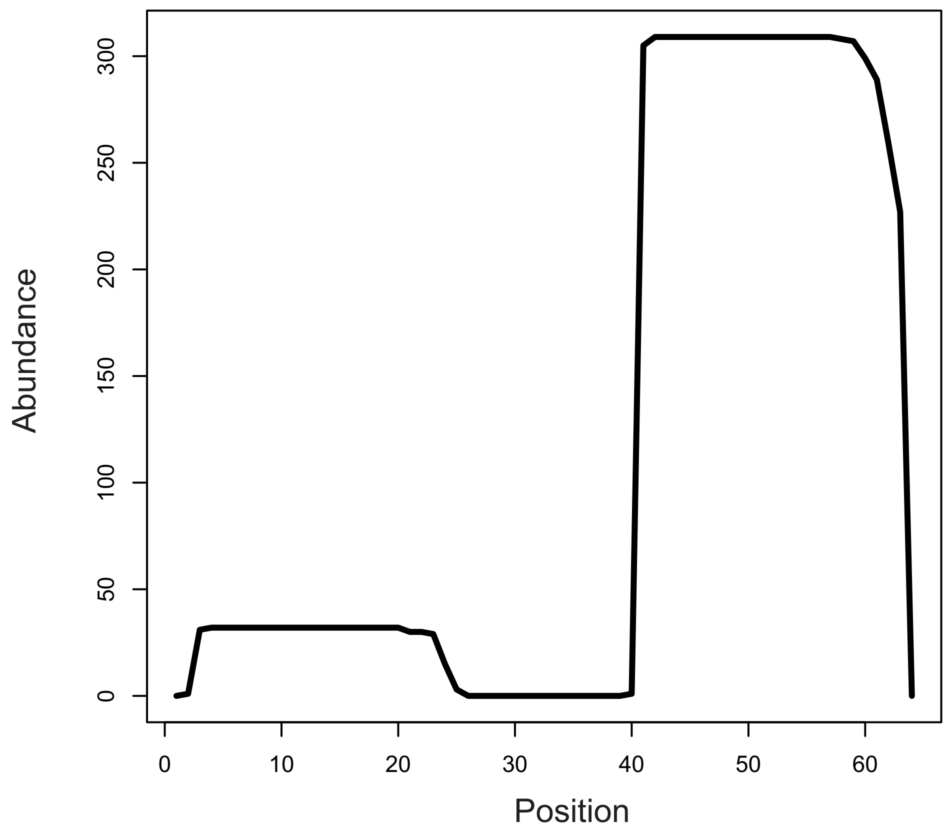


```
TGCCCAACATGCTGGAACCAGCTGTTTTAACTAGTTCTGGTTGGCTCCGGTATGTTTCAGCGAC  Raw reads
........................................TTGGCTCCGGTATGTTTCAGCGA.  222
........................................TTGGCTCCGGTATGTTTCAGCG..  32
........................................TTGGCTCCGGTATGTTTCAGC...  30
..CCCAACATGCTGGAACCAGCT.........................................  13
..CCCAACATGCTGGAACCAGCTG........................................  12
........................................TTGGCTCCGGTATGTTTCAG....  10
........................................TTGGCTCCGGTATGTTTCA.....  8
.........................................TGGCTCCGGTATGTTTCAGCGA.  4
..CCCAACATGCTGGAACCAGCTGT.......................................  3
.GCCCAACATGCTGGAACCA............................................  1
..CCCAACATGCTGGAACCA............................................  1
..CCCAACATGCTGGAACCAGC..........................................  1
...CCAACATGCTGGAACCAGCT.........................................  1
........................................TTGGCTCCGGTATGTTT.......  1
........................................TTGGCTCCGGTATGTTTC......  1
.......................................GTTGGCTCCGGTATGTTTCAGCGA.  1
.((..(((((((((((.((((((...............)))))).)))))))))))...))... (-24.36 MFE)
```

mir-n941

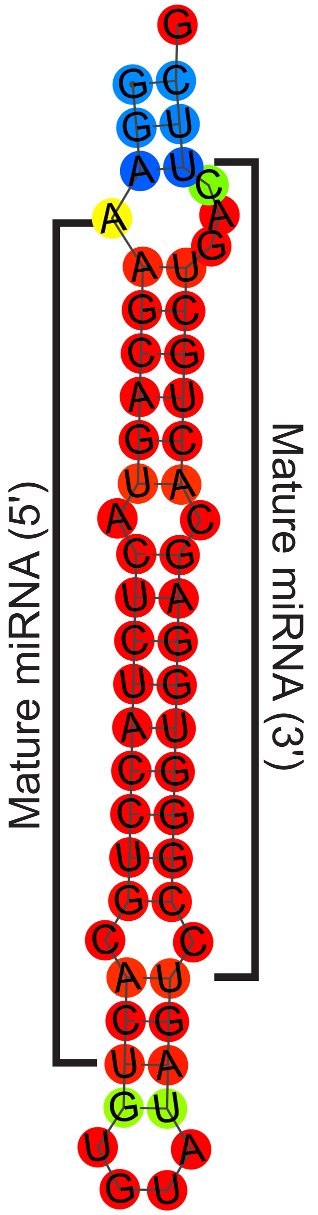

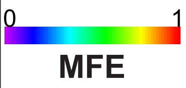

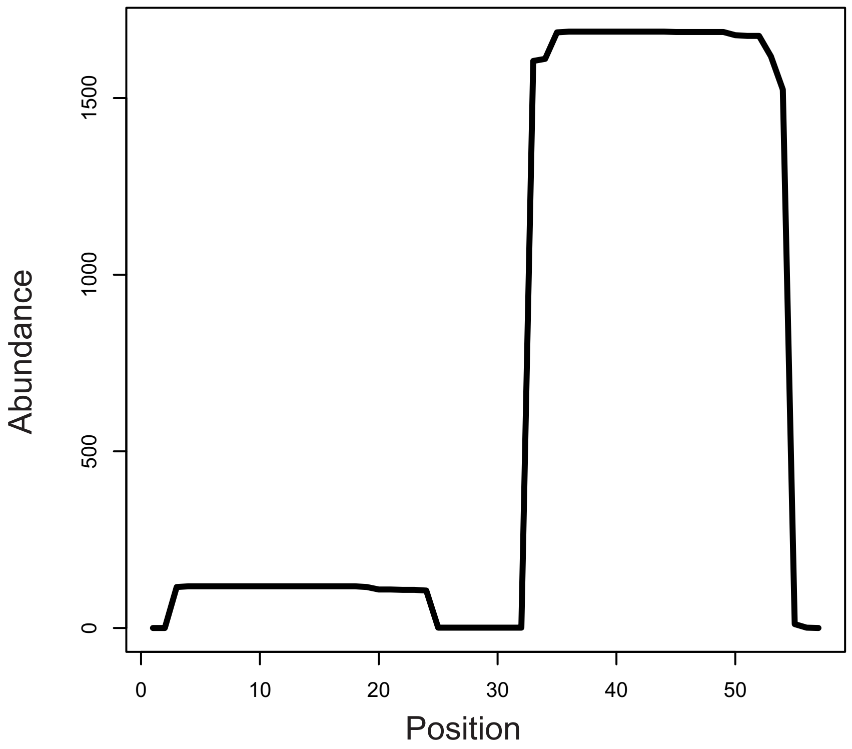


```
GGAAAGCAGTACTCTACCTGCACTGTGTATAGTCCGGGTGGAGCACTGCTGACTTCG   Raw reads
................................TCCGGGTGGAGCACTGCTGACT...   1437
..AAAGCAGTACTCTACCTGCACT.................................   103
................................TCCGGGTGGAGCACTGCTGAC....   91
..................................CGGGTGGAGCACTGCTGACT...   68
................................TCCGGGTGGAGCACTGCTGA.....   54
................................TCCGGGTGGAGCACTGCTGACTT..   10
................................TCCGGGTGGAGCACTGC........   9
..AAAGCAGTACTCTACCT.......................................   7
.................................CCGGGTGGAGCACTGCTGACT...   6
..................................CGGGTGGAGCACTGCTGA.....   4
..AAAGCAGTACTCTACC........................................   3
..................................CGGGTGGAGCACTGCTGAC....   3
..AAAGCAGTACTCTACCTGCAC..................................   2
...AAGCAGTACTCTACCTGCACT.................................   2
................................TCCGGGTGGAGCACTGCT.......   2
...................................GGGTGGAGCACTGCTGACT...   2
..AAAGCAGTACTCTACCTGC....................................   1
..................TGCACTGTGTATAGTCCGGGTGGAGC.............   1
................................TCCGGGTGGAGCACTGCTGACTTC.   1

(((.((((((.(((((((((.((((....)))).))))))))).))))))...))). (-27.80 MFE)
```

mir-n943

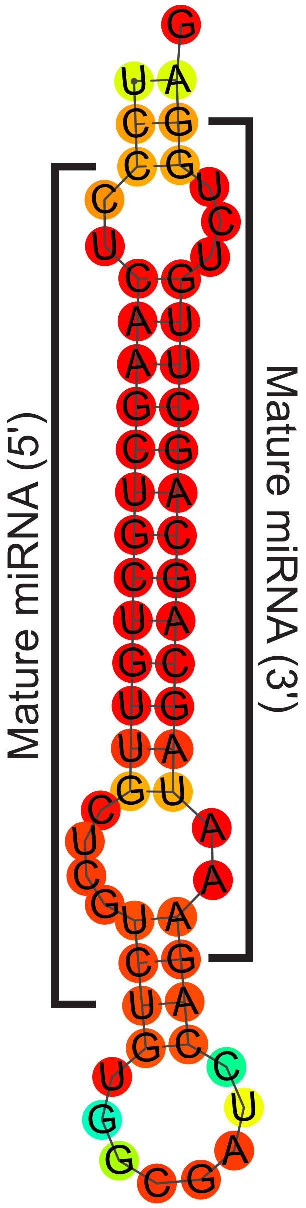

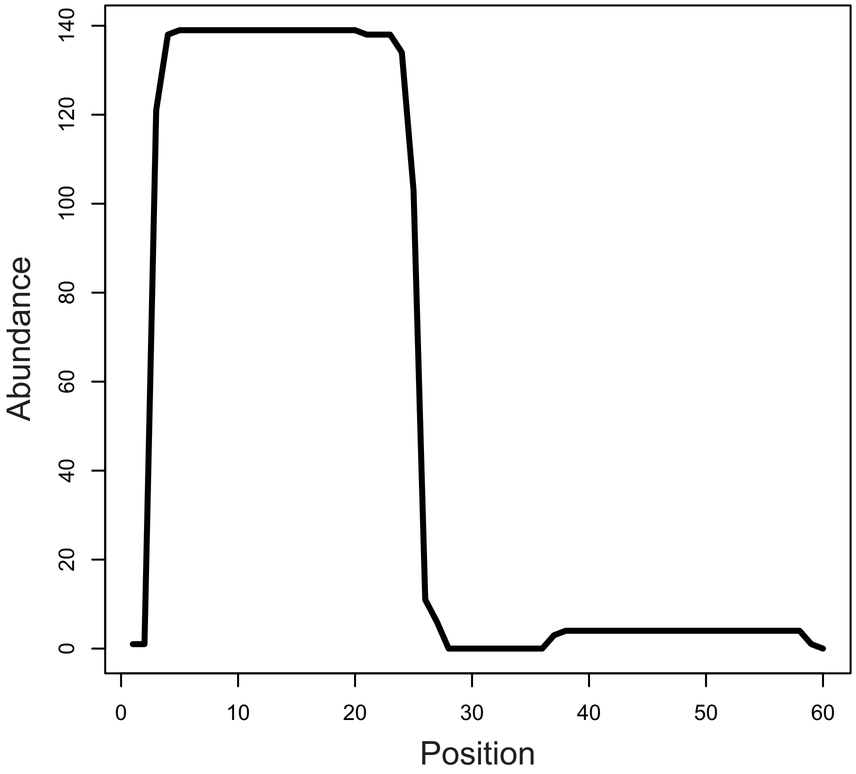


```
TCCCTCAAGCTGCTGTTGCTCGTCTGTGGCGATCCAGAAATAGCAGCAGCTTGTCTGGAG      Raw reads
..CCTCAAGCTGCTGTTGCTCGTCT...................................      80
..CCTCAAGCTGCTGTTGCTCGTC....................................      30
...CTCAAGCTGCTGTTGCTCGTCT...................................      12
..CCTCAAGCTGCTGTTGCTCGTCTG..................................      4
...CTCAAGCTGCTGTTGCTCGTCTGT.................................      4
..CCTCAAGCTGCTGTTGCTCGT.....................................      3
....................................GAAATAGCAGCAGCTTGTCTGG..      3
..CCTCAAGCTGCTGTTGCTCGTCTGT.................................      2
..CCTCAAGCTGCTGTTGCT........................................      1
TCCCTCAAGCTGCTGTTGCTCGT.....................................      1
....TCAAGCTGCTGTTGCTCGTC....................................      1
...CTCAAGCTGCTGTTGCTCGTCTG..................................      1
    .....................................AAATAGCAGCAGCTTGTCTGGA.     1

(((..((((((((((((....((((........))))..))))))))))))...))). (-27.50 MFE)
```

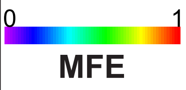

mir-n956

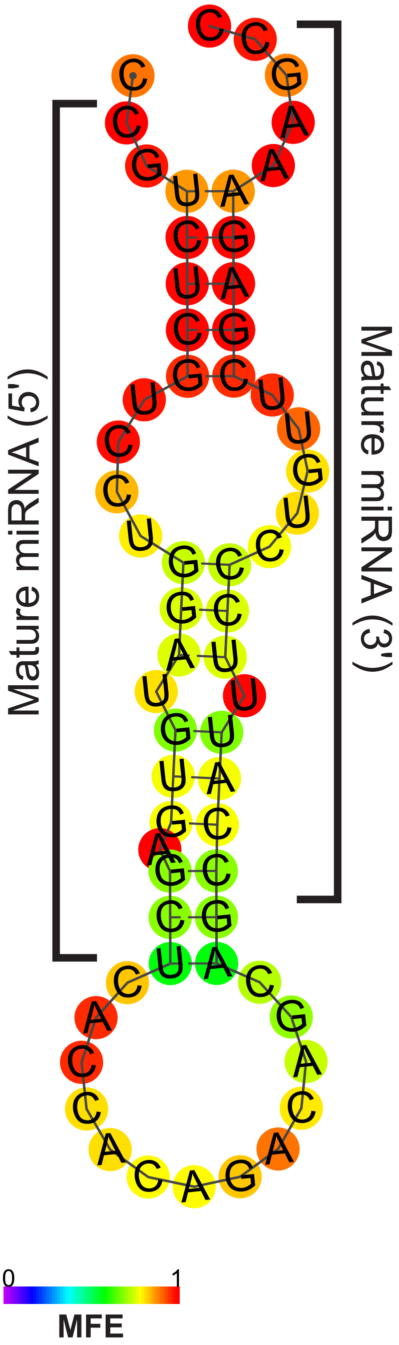

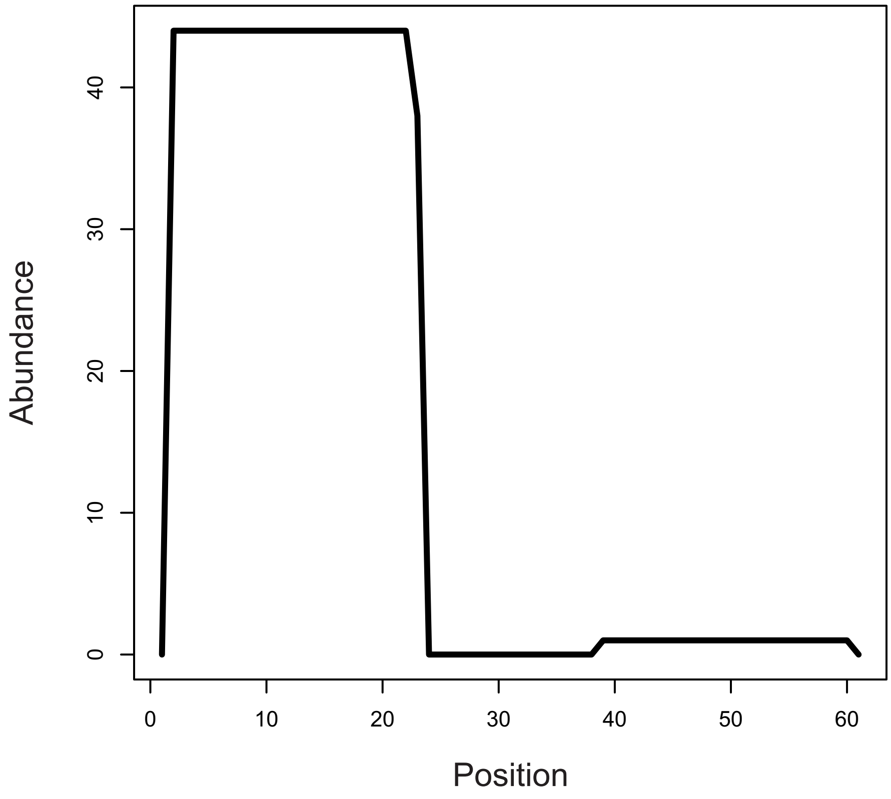


```
CCGTCTCGTCCTGGATGTGAGCTCACCACAGACAGCAGCCATTTCCCTGTTCGAGAAAGCC     Raw reads
.CGTCTCGTCCTGGATGTGAGCT.....................................     38
.CGTCTCGTCCTGGATGTGAGC......................................     6
......................................CCATTTCCCTGTTCGAGAAAGC.     1

...(((((..(.(((.(((.((((..........).)))))).)))..)..))))).....     (-15.30 MFE)
```

mir-n961

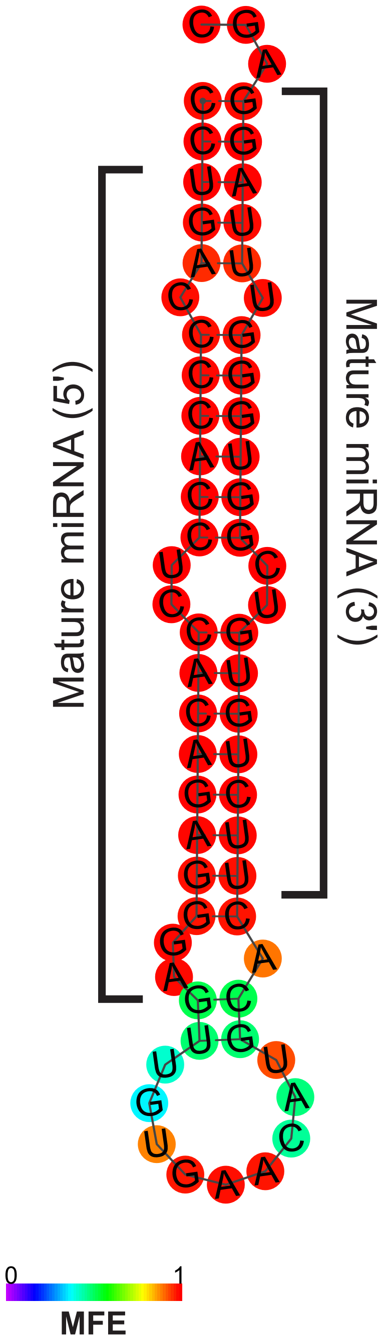

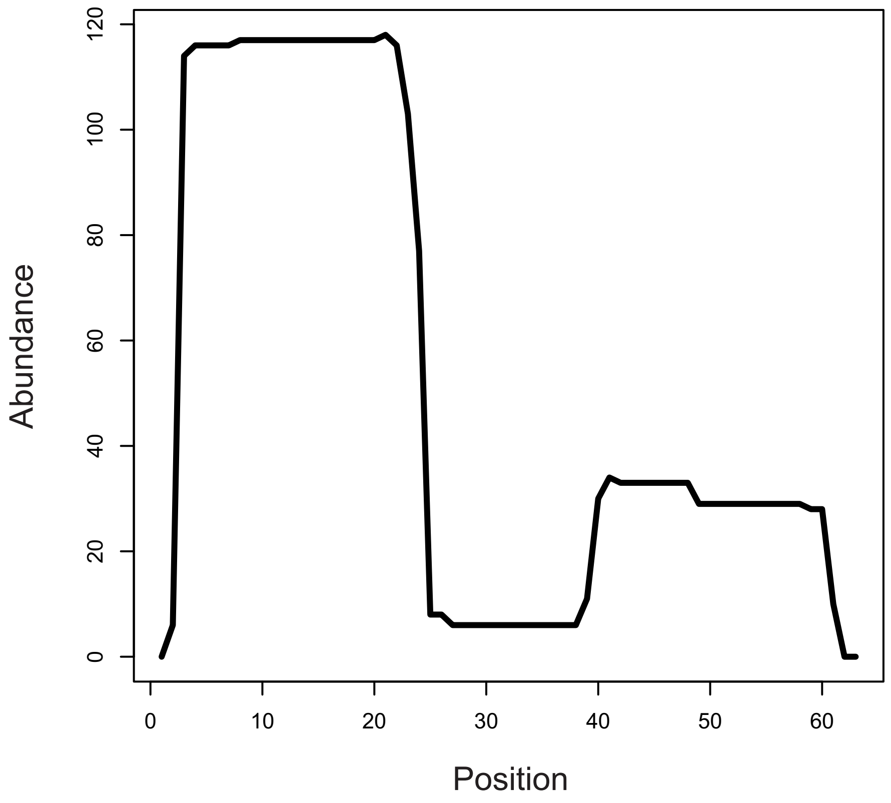


```
CCTGACCCCACCTCCACAGAGGGAGTTGTGAACATGCACTTCTGTGTCGGTGGGTTTAGGAGC  Raw reads
..TGACCCCACCTCCACAGAGGGA........................................  69
..TGACCCCACCTCCACAGAGGG.........................................  24
.......................................TTCTGTGTCGGTGGGTTTAGG...  11
..TGACCCCACCTCCACAGAGG..........................................  9
.......................................TTCTGTGTCGGTGGGTTTAGGA..  7
......................................CTTCTGTGTCGGTGGGTTTAGG...  5
.CTGACCCCACCTCCACAGAGG..........................................  4
..TGACCCCACCTCCACAGAGGGAGT.....................................  3
........................................TCTGTGTCGGTGGGTTTAGGA..  3
..TGACCCCACCTCCACAGAG...........................................  2
.CTGACCCCACCTCCACAGAGGG.........................................  2
...GACCCCACCTCCACAGAGGGA........................................  2
........................GTTGTGAACATGCACTTCTGTGTC...............  2
........................................TCTGTGTCGGTGGGTTTAGG...  2
.......CCACCTCCACAGAGGG.........................................  1
..TGACCCCACCTCCACAGAGGGAG......................................  1
....................GGGAGTTGTGAACATGCACT.......................  1
.......................AGTTGTGAACATGCACTT......................  1
.........................TTGTGAACATGCACTTCTGTGTC...............  1
..........................TGTGAACATGCACTTCTGTGTC...............  1
.......................................TTCTGTGTCGGTGGGTTTA.....  1

(((((.((((((..((((((((....((....))....))))))))..)))))).))))))...  (-34.00 MFE)
```

mir-n965

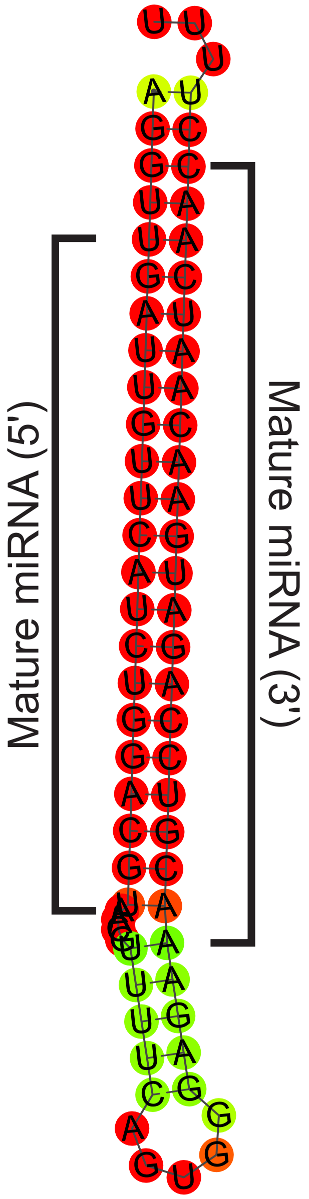

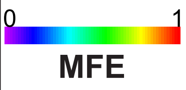

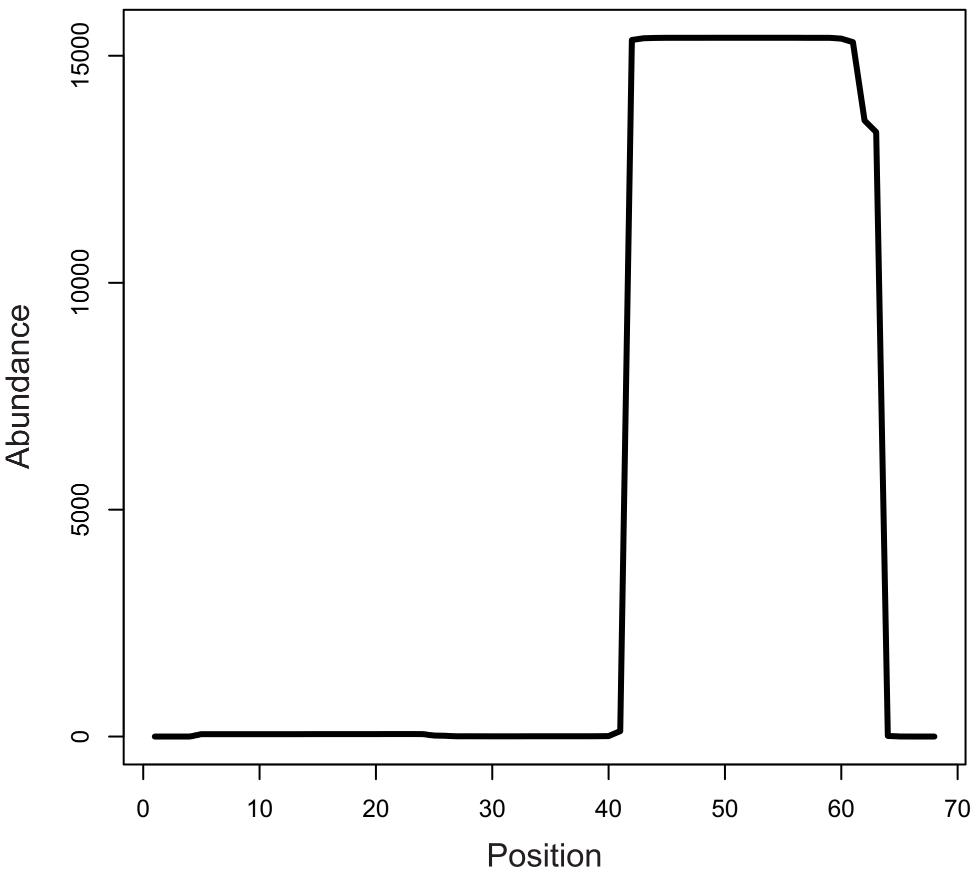


```
AGGTTGATTGTTCATCTGGACGTAACGTTTTCAGTGGGAGAAACGTCCAGATGAACAATCAACCTTTT      Raw read
.........................................AACGTCCAGATGAACAATCAAC.....      13202
.........................................AACGTCCAGATGAACAATCA.......      1678
.........................................AACGTCCAGATGAACAATCAA......      249
.........................................AACGTCCAGATGAACAATC........      68
........................................AAACGTCCAGATGAACAATCAAC.....      56
........................................AAACGTCCAGATGAACAATCA.......      37
..........................................ACGTCCAGATGAACAATCAAC.....      34
....TGATTGTTCATCTGGACGTA............................................      33
.........................................AACGTCCAGATGAACAAT.........      17
....TGATTGTTCATCTGGACGTAAC..........................................      13
.........................................AACGTCCAGATGAACAATCAACC....      13
........................................AAACGTCCAGATGAACAATC........      9
.......................................GAAACGTCCAGATGAACAATCA.......      7
...........................................CGTCCAGATGAACAATCAAC.....      7
....TGATTGTTCATCTGGACGTAA...........................................      4
........................................AAACGTCCAGATGAACAATCAA......      4
............................................GTCCAGATGAACAATCA.......      3
....TGATTGTTCATCTGGACGT.............................................      2
....................CGTAACGTTTTCAGTGGGA.............................      2
........................................AAACGTCCAGATGAAC............      2
..........................................ACGTCCAGATGAACAATCA.......      2
..........................................ACGTCCAGATGAACAATCAA......      2
................................AGTGGGAGAAACGTCCAGATGAACAATCAAC.....      2
....TGATTGTTCATCTGGACGTAACGT........................................      1
.............ATCTGGACGTAACGTT.......................................      1
..............TCTGGACGTAACGTTTTCAGTGGGA.............................      1
...........................TTTTCAGTGGGAGAAAC........................      1
......................................AGAAACGTCCAGATGAACAATC........      1
............................................GTCCAGATGAACAATCAAC.....      1
................................................AGATGAACAATCAACC....      1
.........................................AACGTCCAGATGAACAATCAACCT...      1

(((((((((((((((((((((((....(((((....))))).))))))))))))))))))))))))... (-41.40 MFE)
```

mir-n003-3

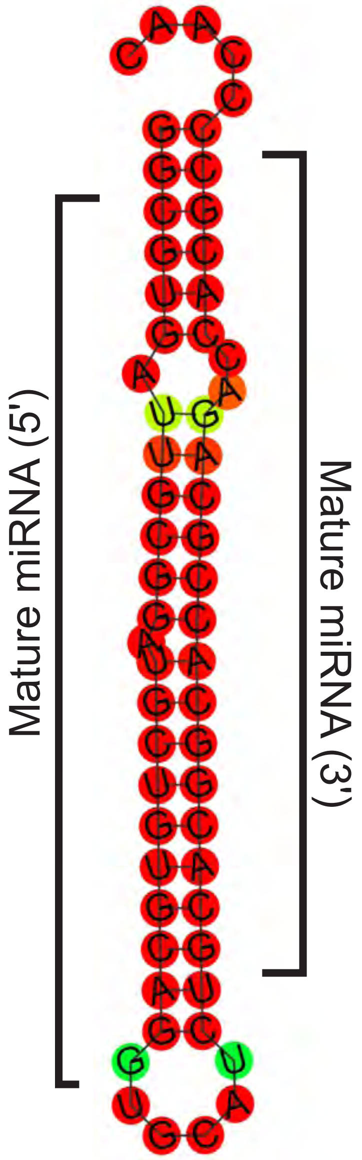

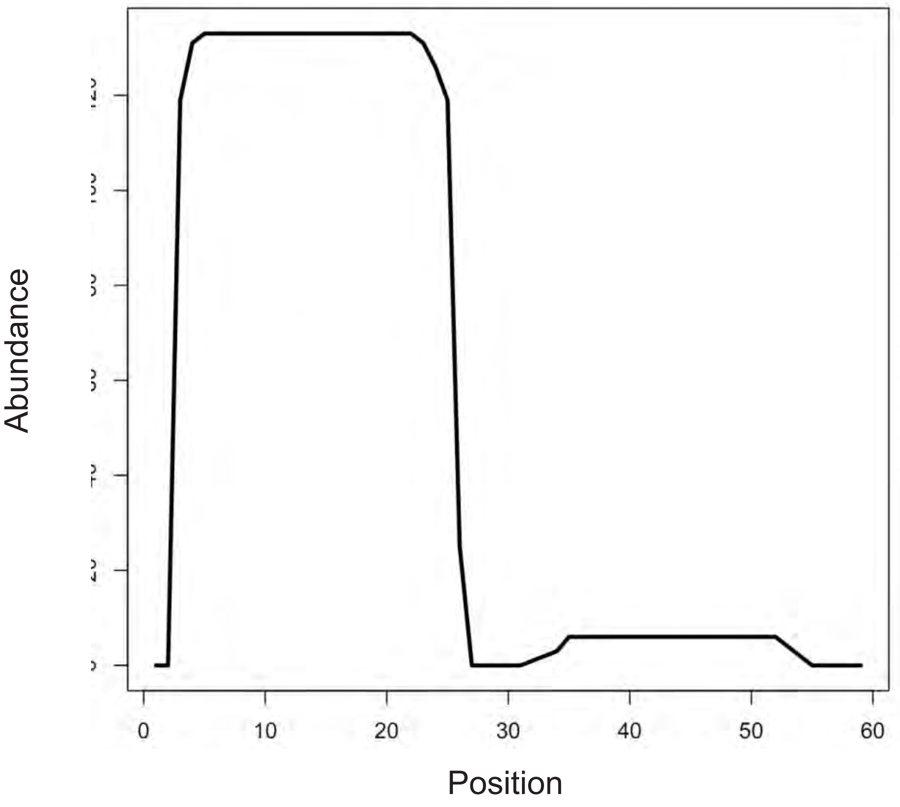


```
GGCGTGATTGCGGATGCTGTGCAGGTGCATCTGCACGGCACCGCAGACCACGCCCCAAC      Raw reads
..CGTGATTGCGGATGCTGTGCAGG..................................      85
..CGTGATTGCGGATGCTGTGCAGGT.................................      20
...GTGATTGCGGATGCTGTGCAGG..................................      9
..CGTGATTGCGGATGCTGTGCAG...................................      7
..CGTGATTGCGGATGCTGTGCA....................................      5
...GTGATTGCGGATGCTGTGCAGGT.................................      3
..CGTGATTGCGGATGCTGTGC.....................................      2
....TGATTGCGGATGCTGTGCAGGT.................................      2
...............................TGCACGGCACCGCAGACCACG.......      1
..................................ACGGCACCGCAGACCACG.......      1
.................................CACGGCACCGCAGACCACGC......      1
..................................ACGGCACCGCAGACCACGC......      1
................................GCACGGCACCGCAGACCACGCC.....      1
..................................ACGGCACCGCAGACCACGCC.....      1

((((((.((((((.(((((((((((....)))))))))))))))))..)))))).....  (-38.50 MFE)
```

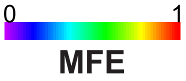

mir-n003-2

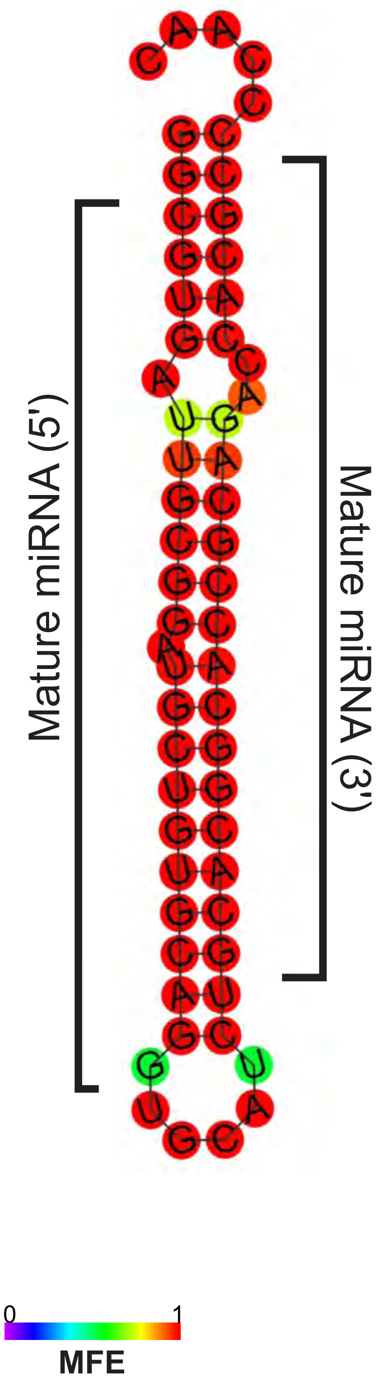

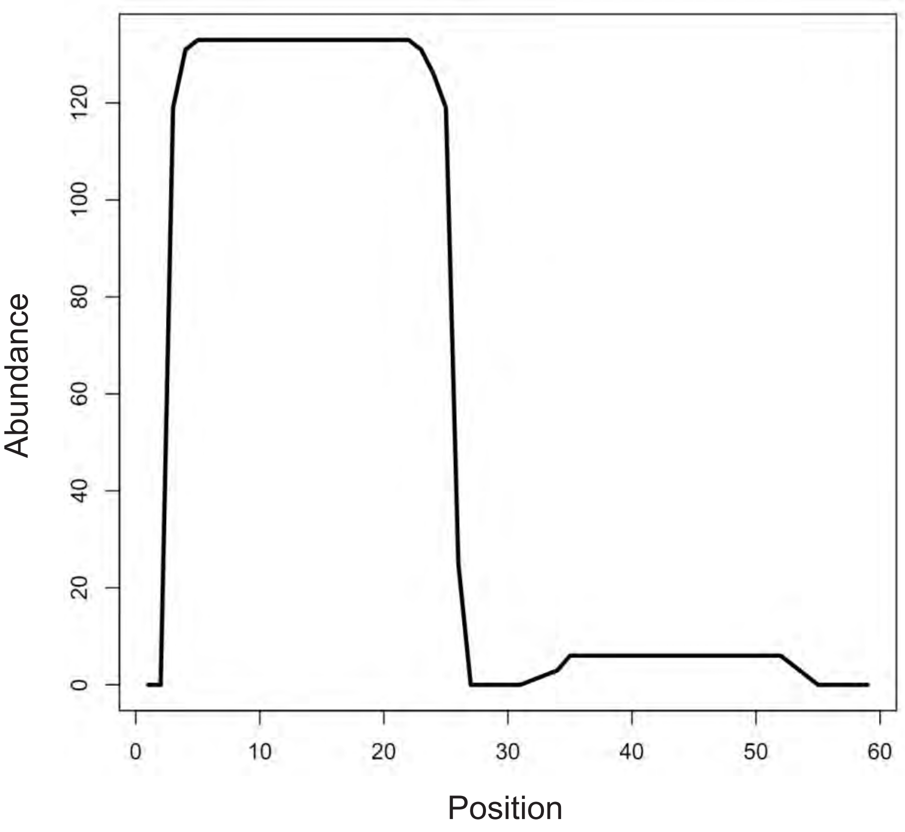


```
GGCGTGATTGCGGATGCTGTGCAGGTGCATCTGCACGGCACCGCAGACCACGCCCCAAC      Raw reads
..CGTGATTGCGGATGCTGTGCAGG..................................      85
..CGTGATTGCGGATGCTGTGCAGGT.................................      20
...GTGATTGCGGATGCTGTGCAGG..................................      9
..CGTGATTGCGGATGCTGTGCAG...................................      7
..CGTGATTGCGGATGCTGTGCA....................................      5
...GTGATTGCGGATGCTGTGCAGGT.................................      3
..CGTGATTGCGGATGCTGTGC.....................................      2
....TGATTGCGGATGCTGTGCAGGT.................................      2
...............................TGCACGGCACCGCAGACCACG.......      1
..................................ACGGCACCGCAGACCACG.......      1
.................................CACGGCACCGCAGACCACGC......      1
..................................ACGGCACCGCAGACCACGC......      1
................................GCACGGCACCGCAGACCACGCC.....      1
..................................ACGGCACCGCAGACCACGCC.....      1

((((((.((((((.(((((((((((....)))))))))))))))))..))))))..... (-38.50 MFE)
```

mir-n885-1

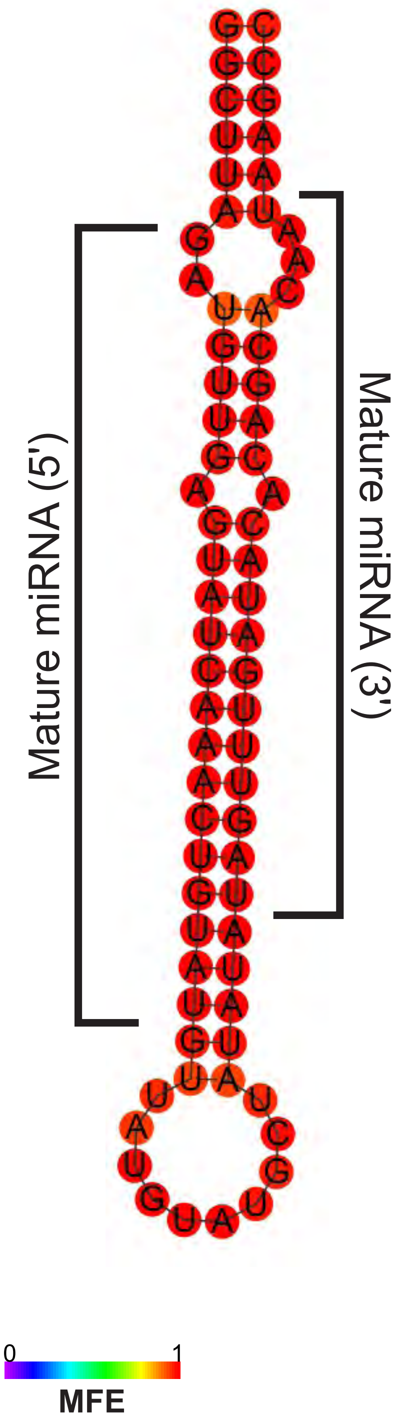

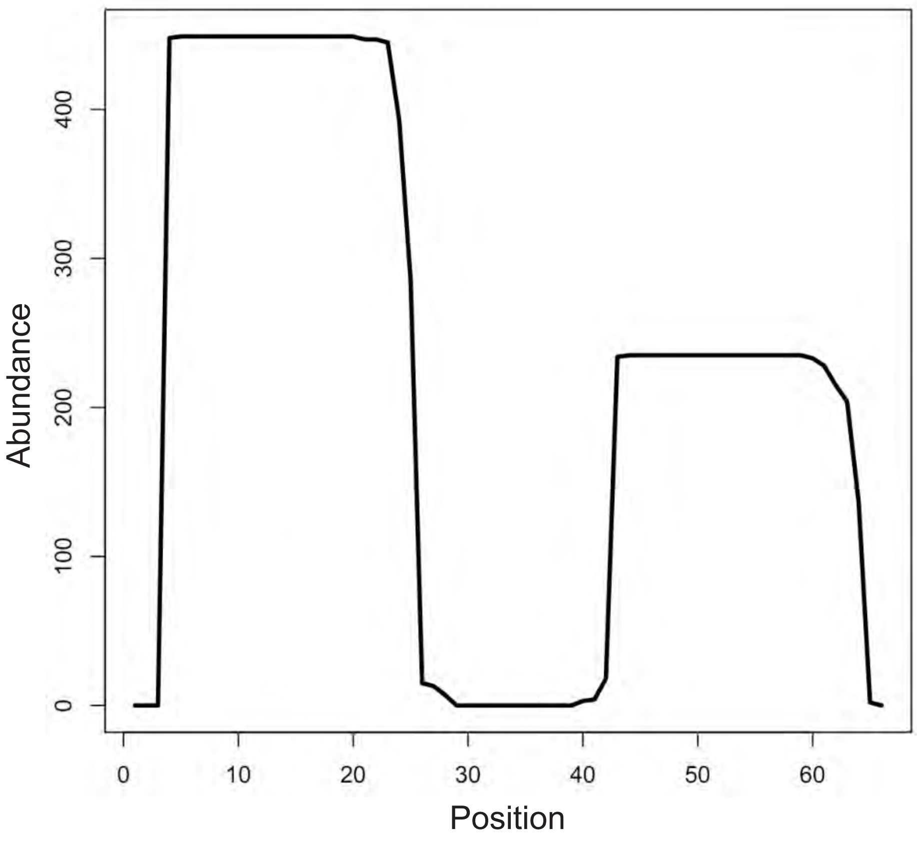


```
GGGCTTAGATGTTGAGTATCAAACTGTATGTTATGTATGCTATATATAGTTTGATACACAGCACAATAAGCC  Raw reads
......GATGTTGAGTATCAAACTGTAT............................................  270
..............................................TAGTTTGATACACAGCACAATA....  133
......GATGTTGAGTATCAAACTGTA.............................................  108
..............................................TAGTTTGATACACAGCACAAT.....  56
......GATGTTGAGTATCAAACTGT..............................................  52
..............................................TAGTTTGATACACAGCACA.......  10
..............................................TAGTTTGATACACAGCACAA......  10
.............................................ATAGTTTGATACACAGCACAAT.....  9
......GATGTTGAGTATCAAACTGTATGTT.........................................  7
......GATGTTGAGTATCAAACTGTATGT..........................................  6
..............................................TAGTTTGATACACAGCAC........  5
.............................................ATAGTTTGATACACAGCACA.......  3
......GATGTTGAGTATCAAACTG...............................................  2
......GATGTTGAGTATCAAACTGTATG...........................................  2
...........................................ATATAGTTTGATACACAGCA.........  2
.............................................ATAGTTTGATACACAGCACAATA....  2
..............................................TAGTTTGATACACAGCACAATAA...  2
......GATGTTGAGTATCAAAC.................................................  1
.......ATGTTGAGTATCAAAC.................................................  1
...........................................ATATAGTTTGATACACAGCACAA......  1
............................................TATAGTTTGATACACAGCACAAT.....  1
...............................................AGTTTGATACACAGCACAAT.....  1

((((((..(((((.((((((((((((((((...(....).)))))))))))))))).))))).)))...)))))) (-32.30 MFE)
```

mir-n885-2

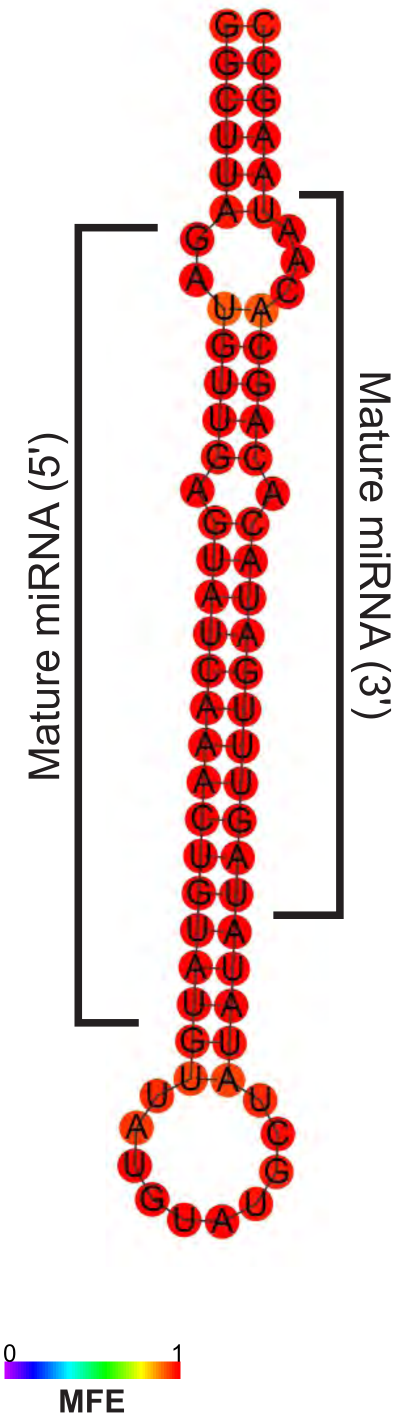

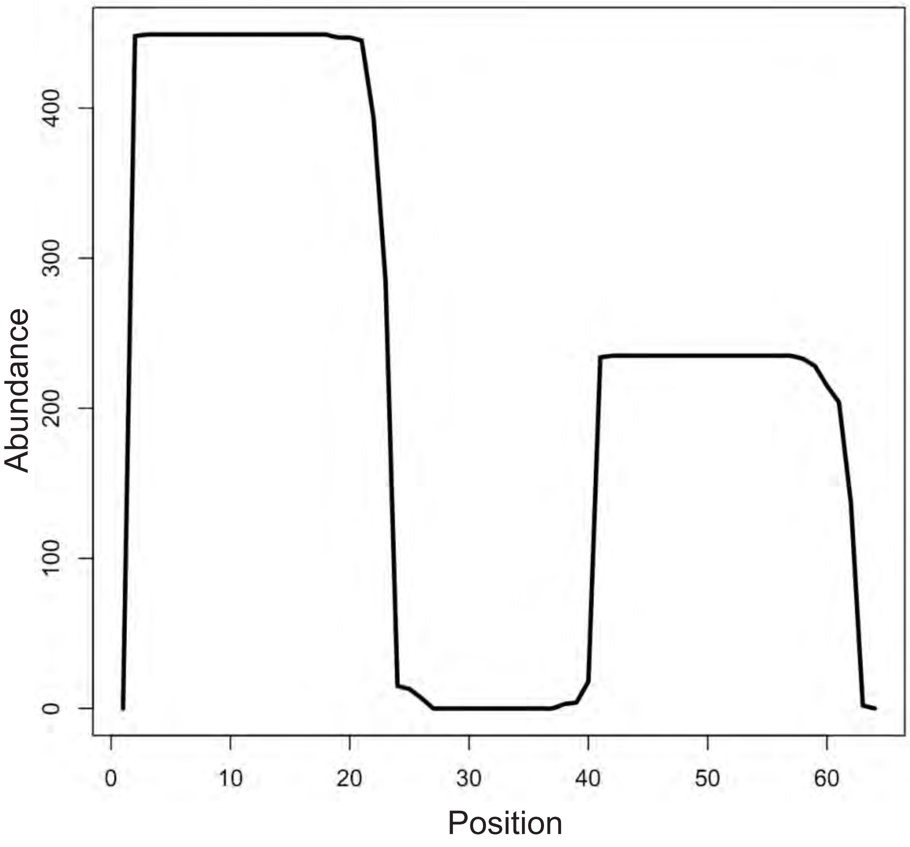


```
GGGCTTAGATGTTGAGTATCAAACTGTATGTTATGTATGCTATATATAGTTTGATACACAGCACAATAAGCC  Raw reads
......GATGTTGAGTATCAAACTGTAT...........................................  270
.............................................TAGTTTGATACACAGCACAATA....  133
......GATGTTGAGTATCAAACTGTA............................................  108
.............................................TAGTTTGATACACAGCACAAT.....  56
......GATGTTGAGTATCAAACTGT.............................................  52
.............................................TAGTTTGATACACAGCACA.......  10
.............................................TAGTTTGATACACAGCACAA......  10
............................................ATAGTTTGATACACAGCACAAT.....  9
......GATGTTGAGTATCAAACTGTATGTT........................................  7
......GATGTTGAGTATCAAACTGTATGT.........................................  6
.............................................TAGTTTGATACACAGCAC........  5
............................................ATAGTTTGATACACAGCACA.......  3
......GATGTTGAGTATCAAACTG..............................................  2
......GATGTTGAGTATCAAACTGTATG..........................................  2
..........................................ATATAGTTTGATACACAGCA.........  2
............................................ATAGTTTGATACACAGCACAATA....  2
.............................................TAGTTTGATACACAGCACAATAA...  2
......GATGTTGAGTATCAAAC................................................  1
.......ATGTTGAGTATCAAAC................................................  1
..........................................ATATAGTTTGATACACAGCACAA......  1
...........................................TATAGTTTGATACACAGCACAAT.....  1
..............................................AGTTTGATACACAGCACAAT.....  1

((((((..(((((.((((((((((((((((...(....).)))))))))))))))).)))))...)))))) (-32.30 MFE)
```

mir-n287-1

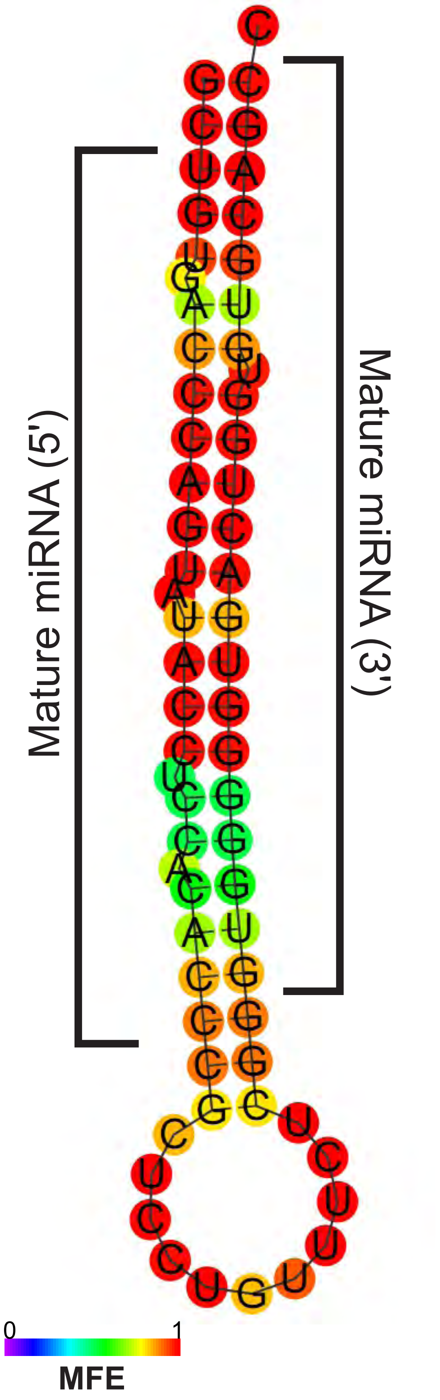

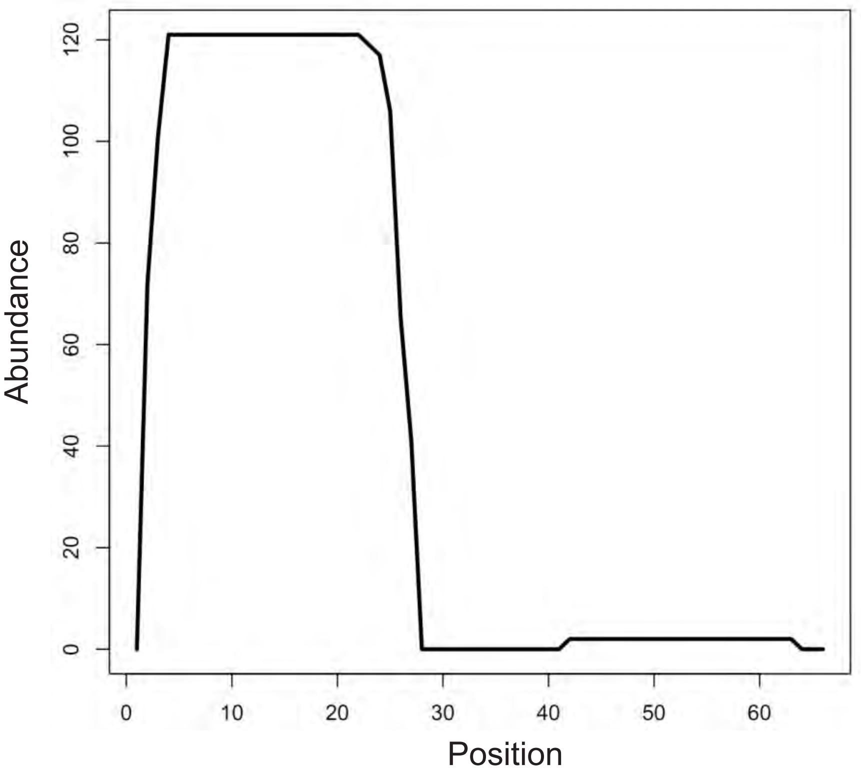


```
CTGTGACCCAGTATACCTCCACACCCGCTCCTGTTTCTCCGGTGGGGGTGACTGGTGTCCAGCCAC        Raw reads
.TGTGACCCAGTATACCTCCACACC.........................................        29
.TGTGACCCAGTATACCTCCACACCCG.......................................        24
..GTGACCCAGTATACCTCCACACCC........................................        11
...TGACCCAGTATACCTCCACACCCG.......................................        10
.TGTGACCCAGTATACCTCCACAC..........................................        8
..GTGACCCAGTATACCTCCACACC.........................................        8
.TGTGACCCAGTATACCTCCACACCC........................................        8
..GTGACCCAGTATACCTCCACACCCG.......................................        7
...TGACCCAGTATACCTCCACACCC........................................        5
...TGACCCAGTATACCTCCACACC.........................................        4
..GTGACCCAGTATACCTCCACAC..........................................        3
.TGTGACCCAGTATACCTCCAC............................................        2
.........................................GTGGGGGTGACTGGTGTCCAGC...        2
.TGTGACCCAGTATACCTCCACA...........................................        1
...TGACCCAGTATACCTCCACA...........................................        1

(((.((((((((.(((((((((....((....)).......)))))))))))))).)))))).... (-28.30 MFE)
```

mir-n287-2

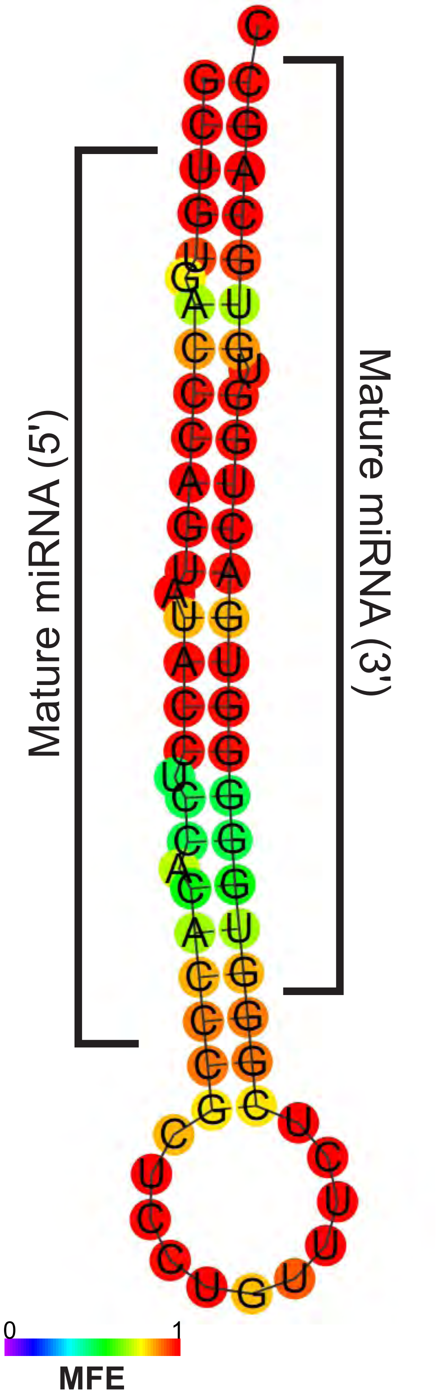

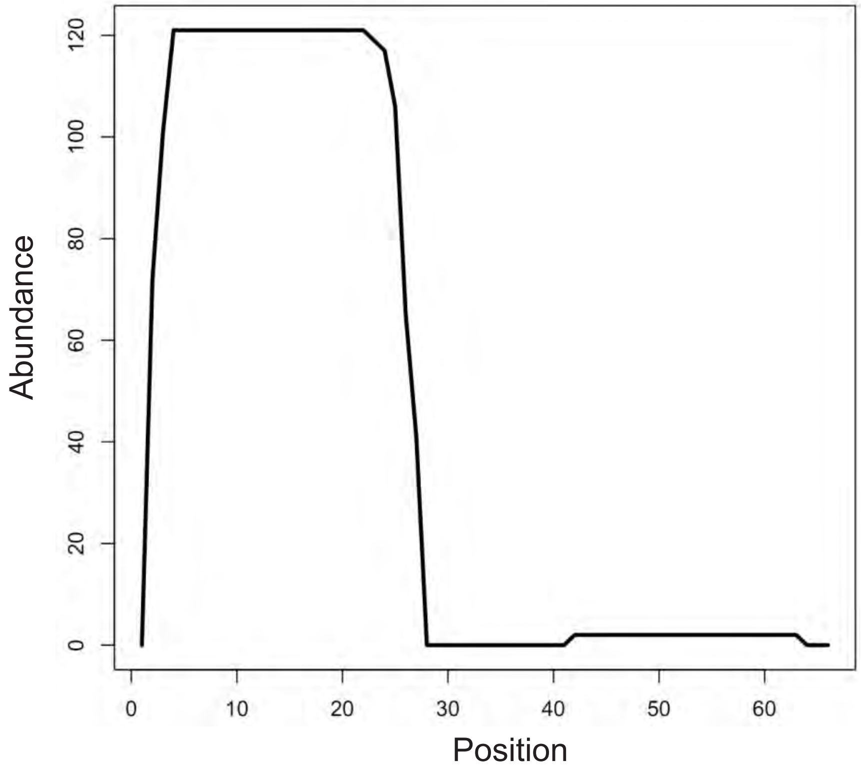


```
CTGTGACCCAGTATACCTCCACACCCGCTCCTGTTTCTCCGGTGGGGGTGACTGGTGTCCAGCCAC        Raw reads
.TGTGACCCAGTATACCTCCACACC.........................................        29
.TGTGACCCAGTATACCTCCACACCCG.......................................        24
..GTGACCCAGTATACCTCCACACCC........................................        11
...TGACCCAGTATACCTCCACACCCG.......................................        10
.TGTGACCCAGTATACCTCCACAC..........................................        8
..GTGACCCAGTATACCTCCACACC.........................................        8
.TGTGACCCAGTATACCTCCACACCC........................................        8
..GTGACCCAGTATACCTCCACACCCG.......................................        7
...TGACCCAGTATACCTCCACACCC........................................        5
...TGACCCAGTATACCTCCACACC.........................................        4
..GTGACCCAGTATACCTCCACAC..........................................        3
.TGTGACCCAGTATACCTCCAC............................................        2
.........................................GTGGGGGTGACTGGTGTCCAGC...        2
.TGTGACCCAGTATACCTCCACA...........................................        1
...TGACCCAGTATACCTCCACA...........................................        1

(((.((((((((.(((((((((....((....)).......)))))))))))))).)))))).... (-28.30 MFE)
```

mir-n287-3

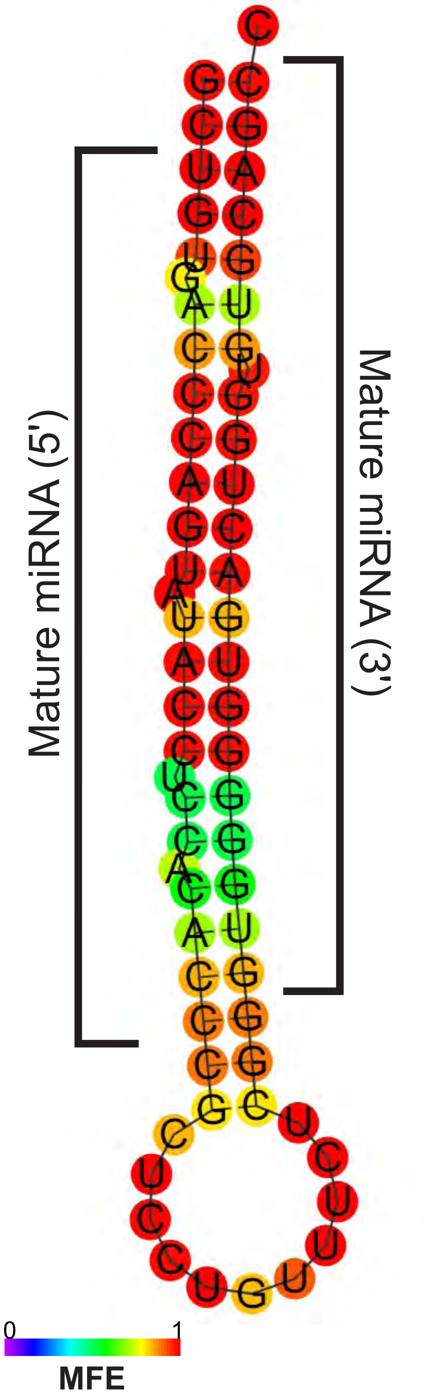

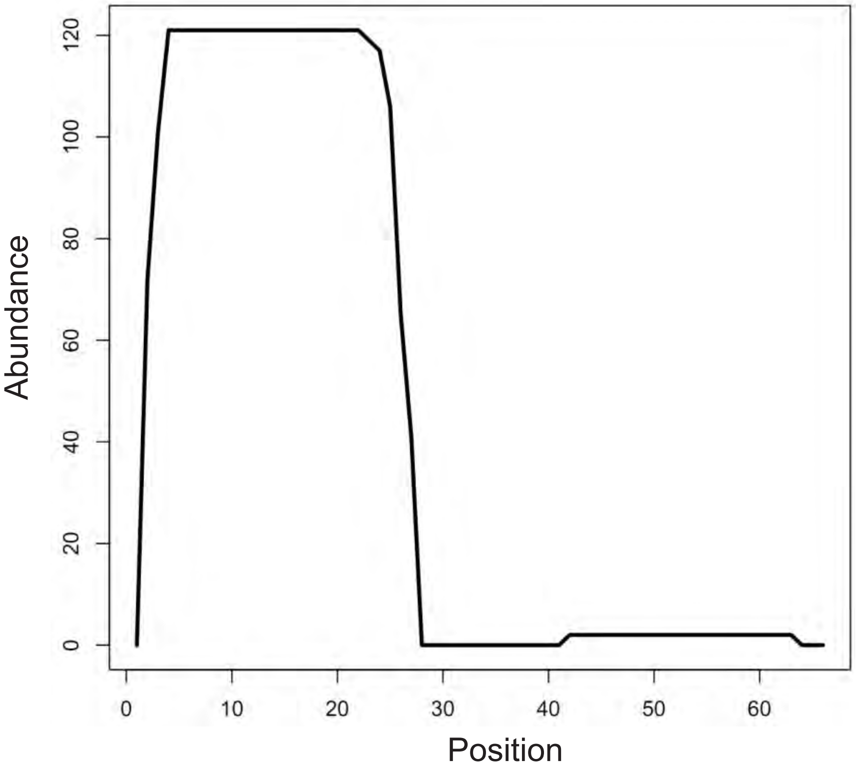


```
CTGTGACCCAGTATACCTCCACACCCGCTCCTGTTTCTCCGGTGGGGGTGACTGGTGTCCAGCCAC       Raw reads
.TGTGACCCAGTATACCTCCACACC.........................................       29
.TGTGACCCAGTATACCTCCACACCCG.......................................       24
..GTGACCCAGTATACCTCCACACCC........................................       11
...TGACCCAGTATACCTCCACACCCG.......................................       10
.TGTGACCCAGTATACCTCCACAC..........................................       8
..GTGACCCAGTATACCTCCACACC.........................................       8
.TGTGACCCAGTATACCTCCACACCC........................................       8
..GTGACCCAGTATACCTCCACACCCG.......................................       7
...TGACCCAGTATACCTCCACACCC........................................       5
...TGACCCAGTATACCTCCACACC.........................................       4
..GTGACCCAGTATACCTCCACAC..........................................       3
.TGTGACCCAGTATACCTCCAC............................................       2
.........................................GTGGGGGTGACTGGTGTCCAGC...       2
.TGTGACCCAGTATACCTCCACA...........................................       1
...TGACCCAGTATACCTCCACA...........................................       1

(((.((((((((.(((((((((....((....)).......)))))))))))))).))))))....  (-28.30 MFE)
```

mir-n987

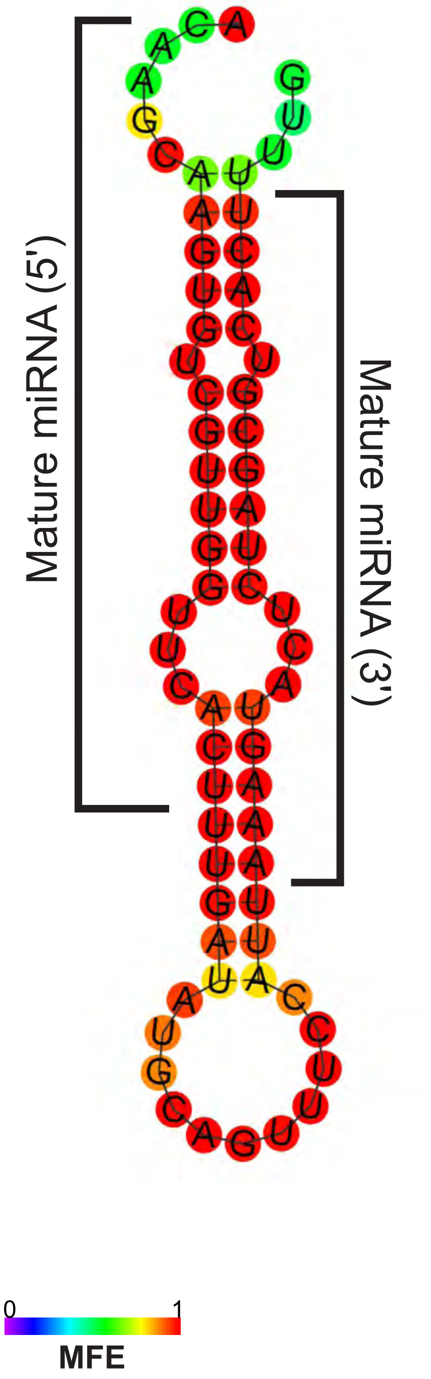

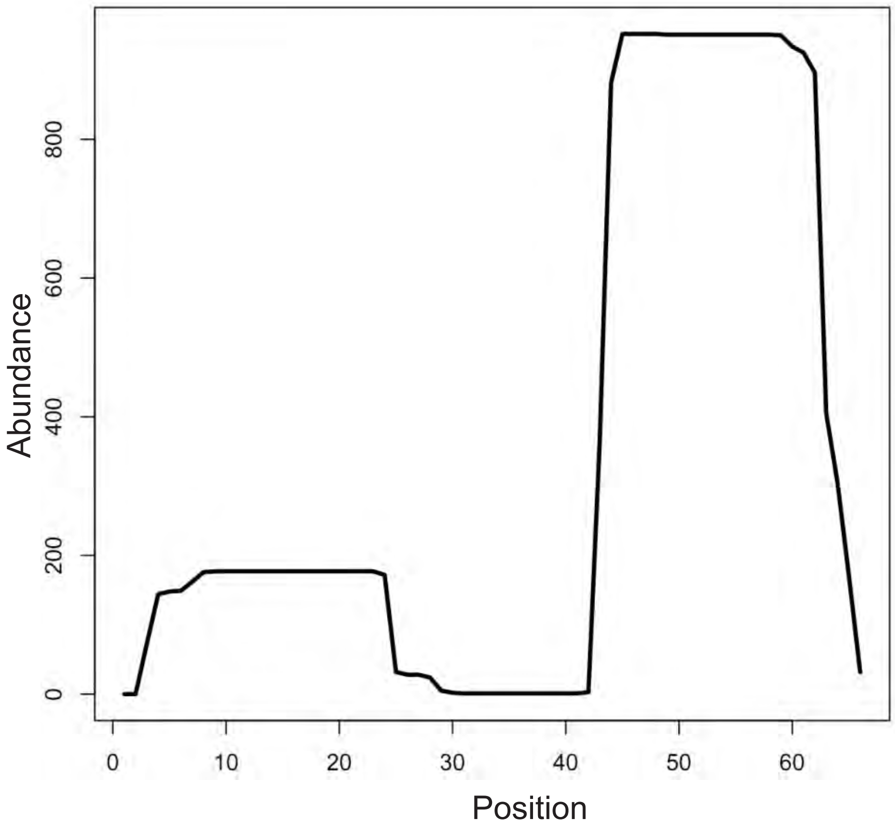


```
ACAAGCAAGTGTCGTTGGTTCACTTTGATATGCAGTTTCCATTAAAGTACTCTAGCGTCACTTTTG        Raw reads
...........................................AAAGTACTCTAGCGTCACT....        274
..........................................TAAAGTACTCTAGCGTCACT....        213
...........................................AAAGTACTCTAGCGTCACTTTT.        105
...........................................AAAGTACTCTAGCGTCACTTT..        79
..AAGCAAGTGTCGTTGGTTCACT..........................................        71
...AGCAAGTGTCGTTGGTTCACT..........................................        64
..........................................TAAAGTACTCTAGCGTCACTT...        62
..........................................TAAAGTACTCTAGCGTCACTTT..        53
...........................................AAAGTACTCTAGCGTCACTT...        37
............................................AAGTACTCTAGCGTCACTTTT.        32
............................................AAGTACTCTAGCGTCACTTTTG        32
..........................................TAAAGTACTCTAGCGTCAC.....        18
...........................................AAAGTACTCTAGCGTCAC.....        11
......AAGTGTCGTTGGTTCACTTTGA......................................        10
.......AGTGTCGTTGGTTCACTTTGA......................................        10
...........................................AAAGTACTCTAGCGTC.......        9
..........................................TAAAGTACTCTAGCGTC.......        7
..........................................TAAAGTACTCTAGCGTCA......        7
....GCAAGTGTCGTTGGTTCACT..........................................        4
...AGCAAGTGTCGTTGGTTCACTT.........................................        4
...AGCAAGTGTCGTTGGTTCAC...........................................        3
......AAGTGTCGTTGGTTCACTTTG.......................................        3
.......AGTGTCGTTGGTTCACTTTGAT.....................................        3
............................................AAGTACTCTAGCGTCACT....        3
..AAGCAAGTGTCGTTGGTTCAC...........................................        2
............................................AAGTACTCTAGCGTCACTTT..        2
..........................................TAAAGTACTCTAGCGTCACTTTT.        2
.....CAAGTGTCGTTGGTTCACT..........................................        1
........GTGTCGTTGGTTCACTTTG.......................................        1
.......AGTGTCGTTGGTTCACTTTGATA....................................        1
............................TATGCAGTTTCCATTAAAGT..................        1
..........................................TAAAGTACTCTAGCGT........        1
...........................................AAAGTACTCTAGCGTCA......        1
............................................AAGTACTCTAGCGTCA......        1
.........................................TTAAAGTACTCTAGCGTCACT....        1
.........................................TTAAAGTACTCTAGCGTCACTT...        1

......(((((.((((((...((((((((...........))))))))...)))))).)))))... (-17.70 MFE)
```

mir-n948

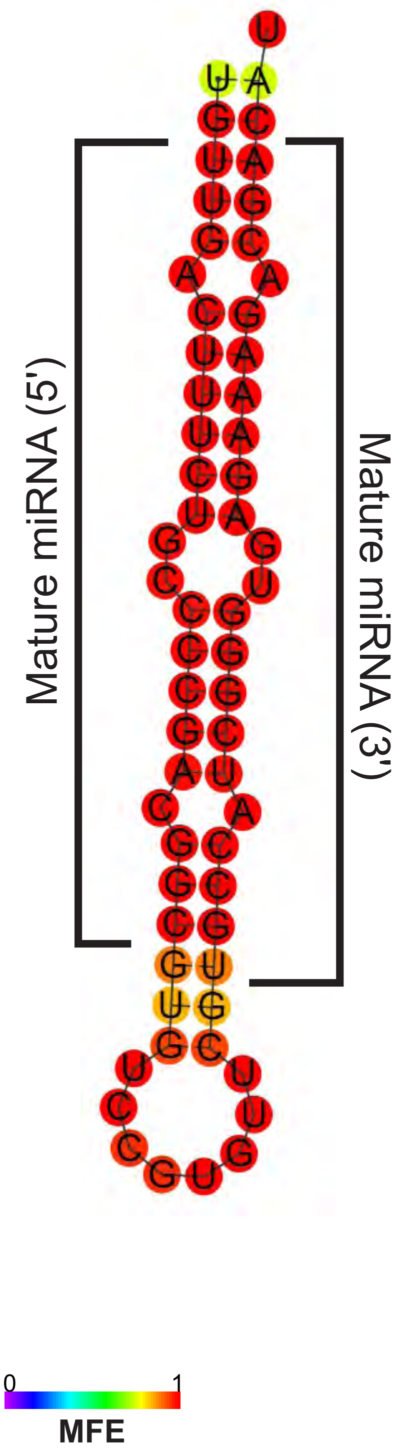

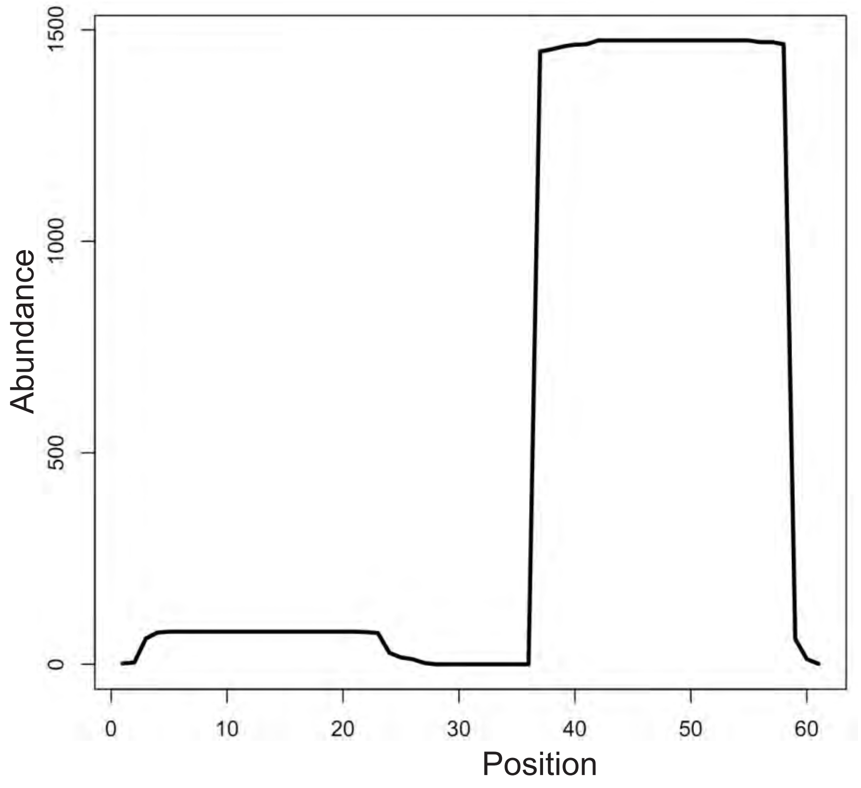


```
TGTTGACTTTCTGCCCCGACGGCGTGTCCGTGTTCGTGCCATCGGGTGAGAAAGACGACAT    Raw reads
....................................TGCCATCGGGTGAGAAAGACGA...    1383
....................................TGCCATCGGGTGAGAAAGACGAC..    48
..TTGACTTTCTGCCCCGACGGC......................................    44
..TTGACTTTCTGCCCCGACGGCG.....................................    9
.........................................TCGGGTGAGAAAGACGA...    9
....................................TGCCATCGGGTGAGAAAGACGACA.    9
...TGACTTTCTGCCCCGACGGCGTG...................................    8
......................................CCATCGGGTGAGAAAGACGA...    6
....................................TGCCATCGGGTGAGAAAGACG....    5
.....................................GCCATCGGGTGAGAAAGACGA...    5
....................................TGCCATCGGGTGAGAAAGA......    4
..TTGACTTTCTGCCCCGACGG.......................................    2
.GTTGACTTTCTGCCCCGACGGC......................................    2
...TGACTTTCTGCCCCGACGGCG.....................................    2
..TTGACTTTCTGCCCCGACGGCGT....................................    2
...TGACTTTCTGCCCCGACGGCGT....................................    2
...TGACTTTCTGCCCCGACGGCGTGT..................................    2
.......................................CATCGGGTGAGAAAGACGA...    2
TGTTGACTTTCTGCCCCGACG........................................    1
TGTTGACTTTCTGCCCCGACGGC......................................    1
....GACTTTCTGCCCCGACGGCGTG...................................    1
....GACTTTCTGCCCCGACGGCGTGT..................................    1
........................................ATCGGGTGAGAAAGACGA...    1
......................................CCATCGGGTGAGAAAGACGACA.    1
.......................................CATCGGGTGAGAAAGACGACA.    1
.......................................CATCGGGTGAGAAAGACGACAT    1

(((((.((((((..(((((.((((((........)))))).)))))..)))))).))))). (-28.30 MFE)
```

mir-n813-1

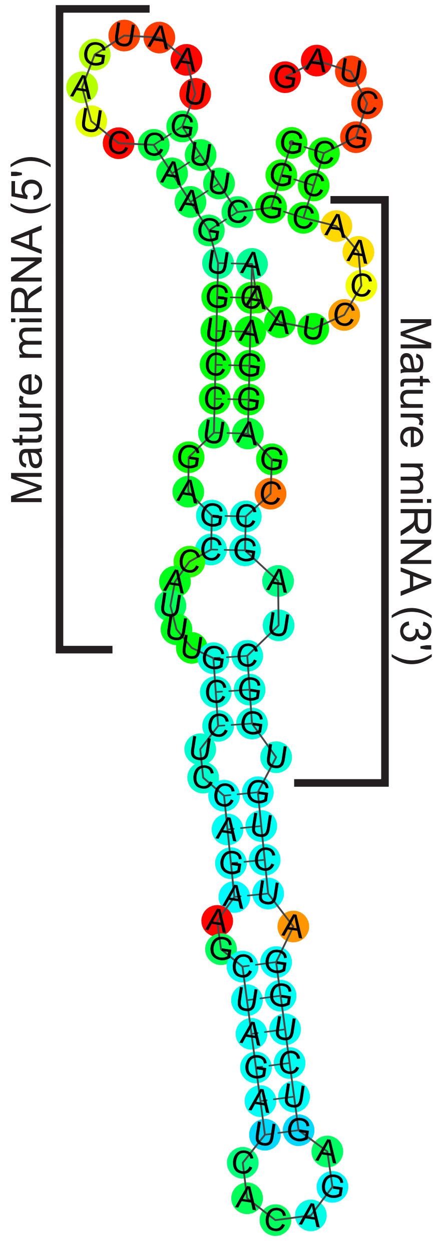

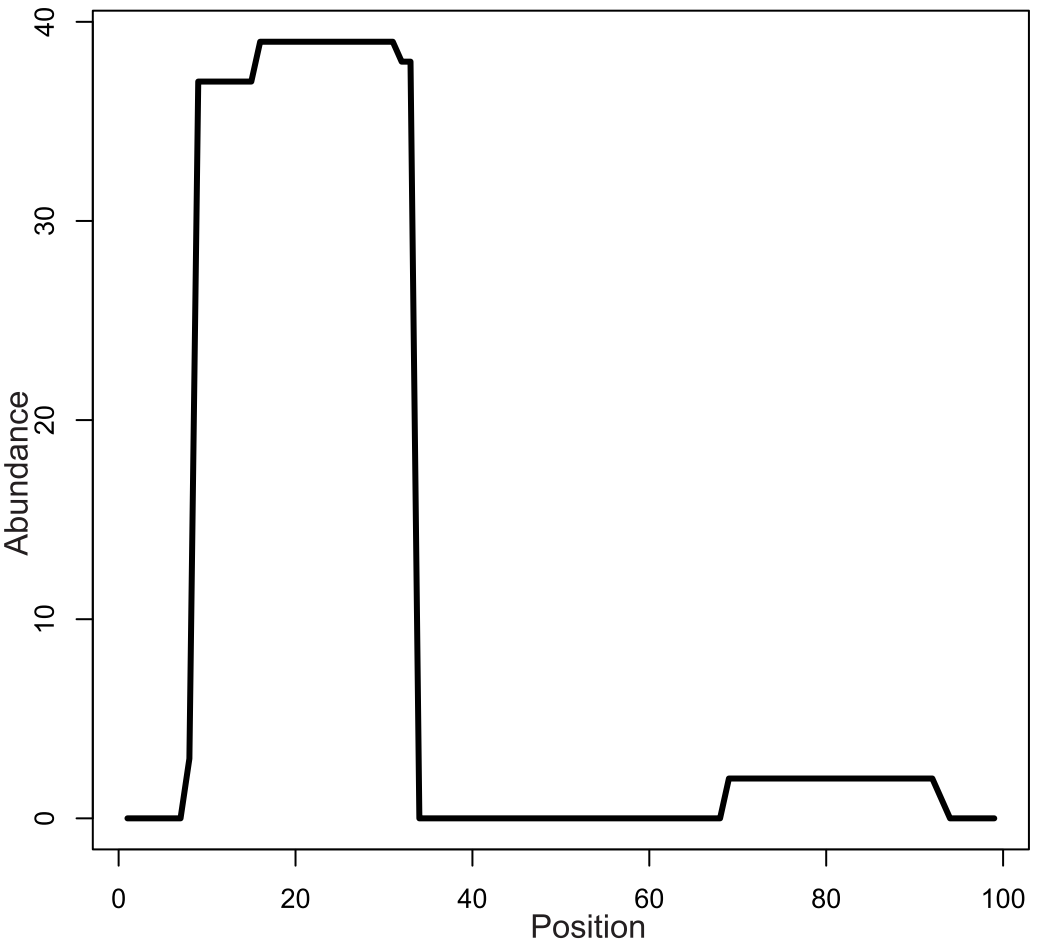


```
GGGCTTGTAATGATCCAAGTGTCCTGAGCCATTTGCCTCCAGAAGCTAGATCACAGAGTCTGGATCTGTGGCTAGCCGAGGACAAATCCAACCCGCTAG  Raw reads
........AATGATCCAAGTGTCCTGAGCCATT..................................................................   33
.......TAATGATCCAAGTGTCCTGAGCCATT..................................................................   3
...............CAAGTGTCCTGAGCCATT..................................................................   2
........AATGATCCAAGTGTCCTGAGCCA....................................................................   1
....................................................................TGGCTAGCCGAGGACAAATCCAAC.......   1
....................................................................TGGCTAGCCGAGGACAAATCCAACC......   1

(((.(((....(((.....((((((.((((((.....((((((..((.......))..))))))...)))))).....)))))).))))))))).....  (-30.70 MFE)
```

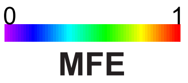

mir-n323

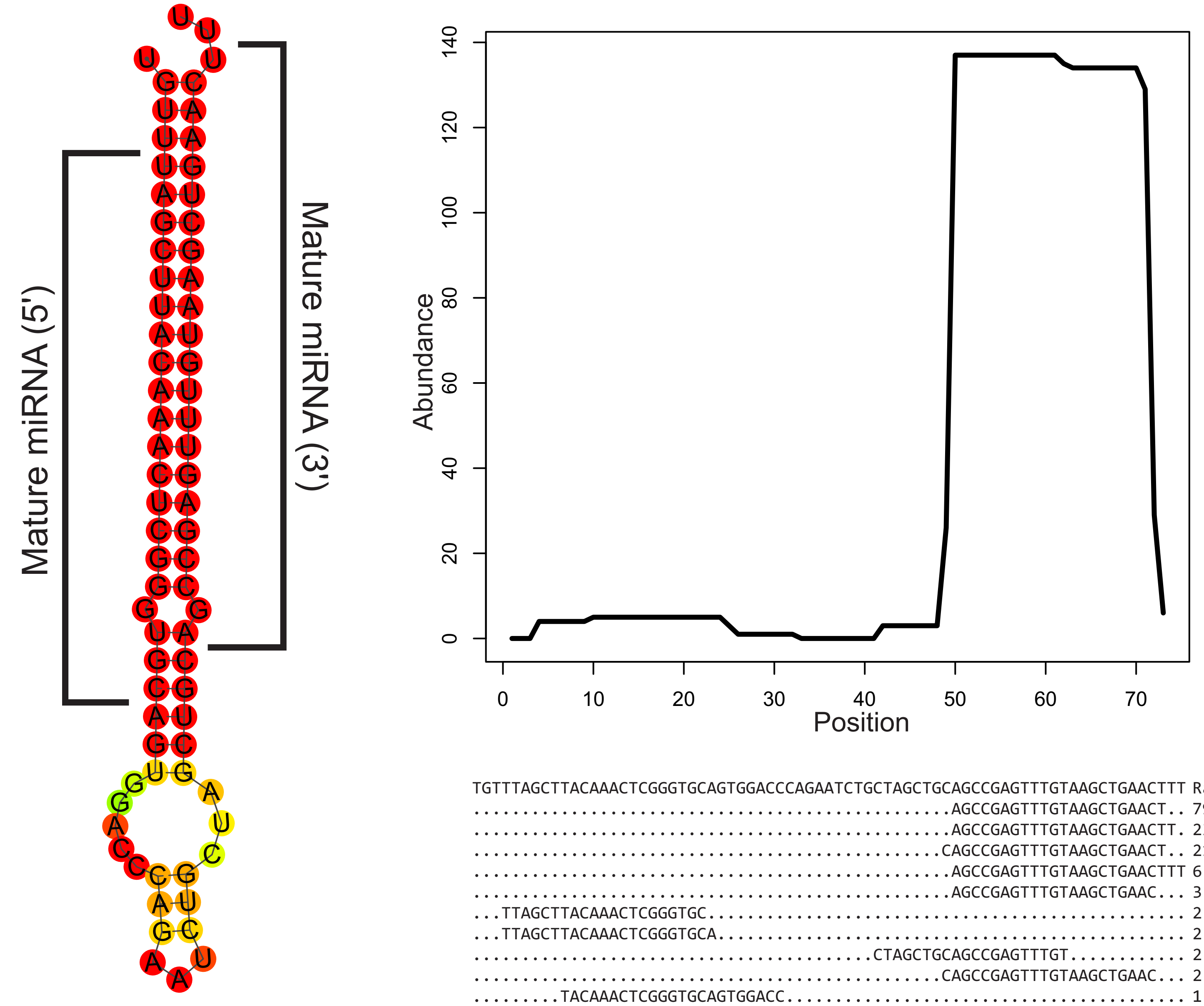


```
TGTTTAGCTTACAAACTCGGGTGCAGTGGACCCAGAATCTGCTAGCTGCAGCCGAGTTTGTAAGCTGAACTTT Raw reads
.................................................AGCCGAGTTTGTAAGCTGAACT.. 79
.................................................AGCCGAGTTTGTAAGCTGAACTT. 23
................................................CAGCCGAGTTTGTAAGCTGAACT.. 21
.................................................AGCCGAGTTTGTAAGCTGAACTTT 6
.................................................AGCCGAGTTTGTAAGCTGAAC... 3
...TTAGCTTACAAACTCGGGTGC................................................. 2
...TTAGCTTACAAACTCGGGTGCA................................................ 2
.........................................CTAGCTGCAGCCGAGTTTGT............ 2
................................................CAGCCGAGTTTGTAAGCTGAAC... 2
.........TACAAACTCGGGTGCAGTGGACC......................................... 1
.........................................CTAGCTGCAGCCGAGTTTGTA........... 1

.(((((((((((((((((((.((((((.....(((...)))...)))))).)))))))))))))))))))... (-43.70 MFE)
```

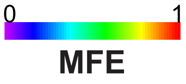

mir-n659

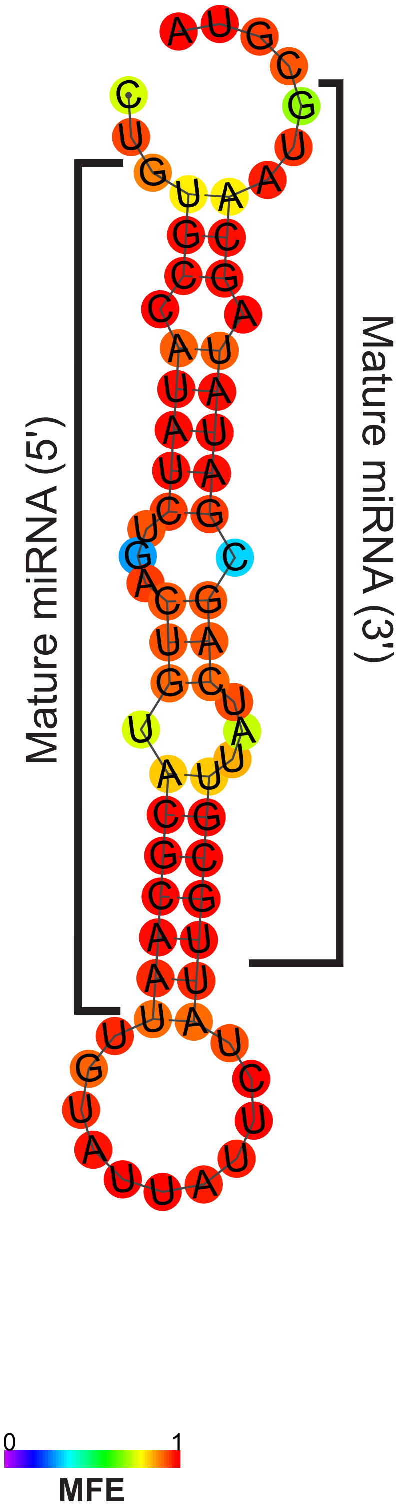

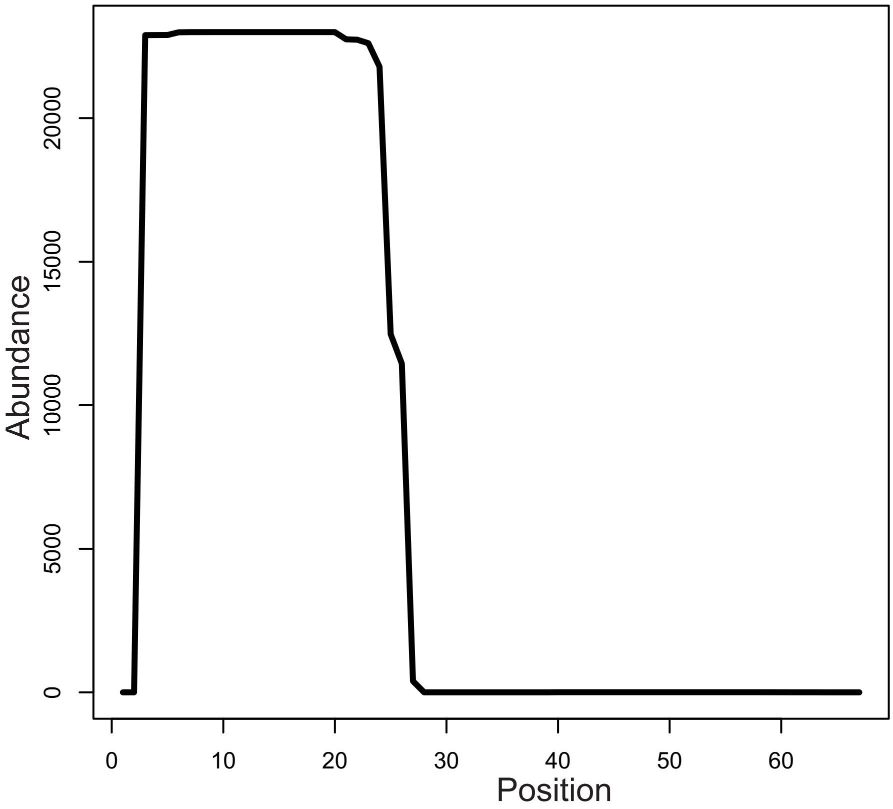


```
CTGTGCCATATCTGACTGTACGCAATTGTATTATTCTATTGCGTTATCAGCGATATAGCAATGCGTA     Raw reads
..GTGCCATATCTGACTGTACGCAAT.........................................     10967
..GTGCCATATCTGACTGTACGCA...........................................     9313
..GTGCCATATCTGACTGTACGCAA..........................................     1020
..GTGCCATATCTGACTGTACGC............................................     820
..GTGCCATATCTGACTGTACGCAATT.......................................      383
..GTGCCATATCTGACTGTA...............................................     250
..GTGCCATATCTGACTGTACG.............................................     123
.....CCATATCTGACTGTACGCAAT.........................................     87
..GTGCCATATCTGACTGTAC..............................................     14
.....CCATATCTGACTGTACGCAATT.......................................      6
......CATATCTGACTGTACGCAAT.........................................     3
....GCCATATCTGACTGTACGCA...........................................     2
.....CCATATCTGACTGTACGCAA..........................................     2
...TGCCATATCTGACTGTACGCAAT.........................................     2
.......................................TGCGTTATCAGCGATATAGCAATG....     2
..GTGCCATATCTGACTGT................................................     1
......CATATCTGACTGTACGCA...........................................     1
........TATCTGACTGTACGCA...........................................     1
......CATATCTGACTGTACGCAA..........................................     1
.TGTGCCATATCTGACTGTACGCAAT.........................................     1
....................CGCAATTGTATTATTCTAT............................     1
.......................................TGCGTTATCAGCGATATAG.........     1
.......................................TGCGTTATCAGCGATATAGC........     1
.......................................TGCGTTATCAGCGATATAGCA.......     1

...(((.(((((.(.(((.(((((((.(.......).)))))))...))))))))).)))....... (-17.80 MFE)
```

mir-n811

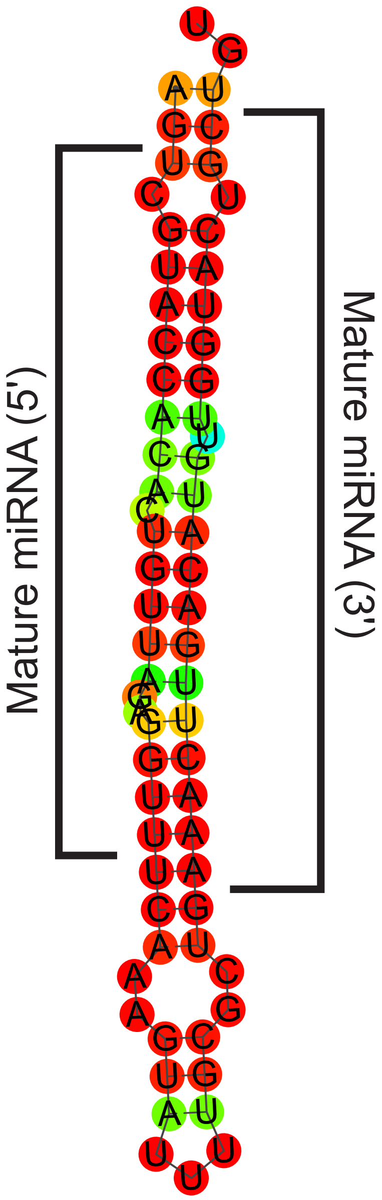

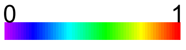

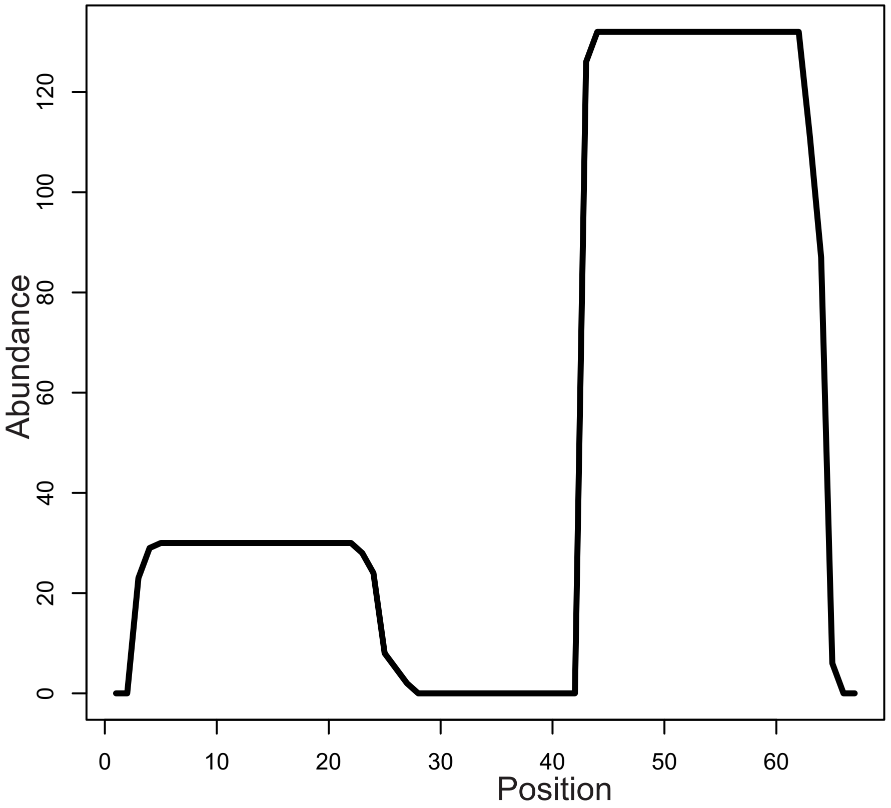


```
AGTCGTACCACACTGTTAGAGGTTTCAAAGTATTTTGCGCTGAAACTTGACATGTTGGTACTGCTGT        Raw reads
..........................................AAACTTGACATGTTGGTACTGC...        78
..........................................AAACTTGACATGTTGGTACTG....        24
..........................................AAACTTGACATGTTGGTACT.....        21
..TCGTACCACACTGTTAGAGGTT...........................................        14
..TCGTACCACACTGTTAGAGGT............................................        4
...........................................AACTTGACATGTTGGTACTGC...        3
..........................................AAACTTGACATGTTGGTACTGCT..        3
...........................................AACTTGACATGTTGGTACTGCT..        3
..TCGTACCACACTGTTAGAGG.............................................        2
...CGTACCACACTGTTAGAGGTT...........................................        2
..TCGTACCACACTGTTAGAGGTTT..........................................        2
...CGTACCACACTGTTAGAGGTTTC.........................................        2
...CGTACCACACTGTTAGAGGTTT..........................................        1
..TCGTACCACACTGTTAGAGGTTTC.........................................        1
...CGTACCACACTGTTAGAGGTTTCA........................................        1
....GTACCACACTGTTAGAGGTTTCA........................................        1

(((.((((((((.(((((..(((((((..((.....))..)))))))))))))).)))))).))).. (-22.50 MFE)
```

mir-n530

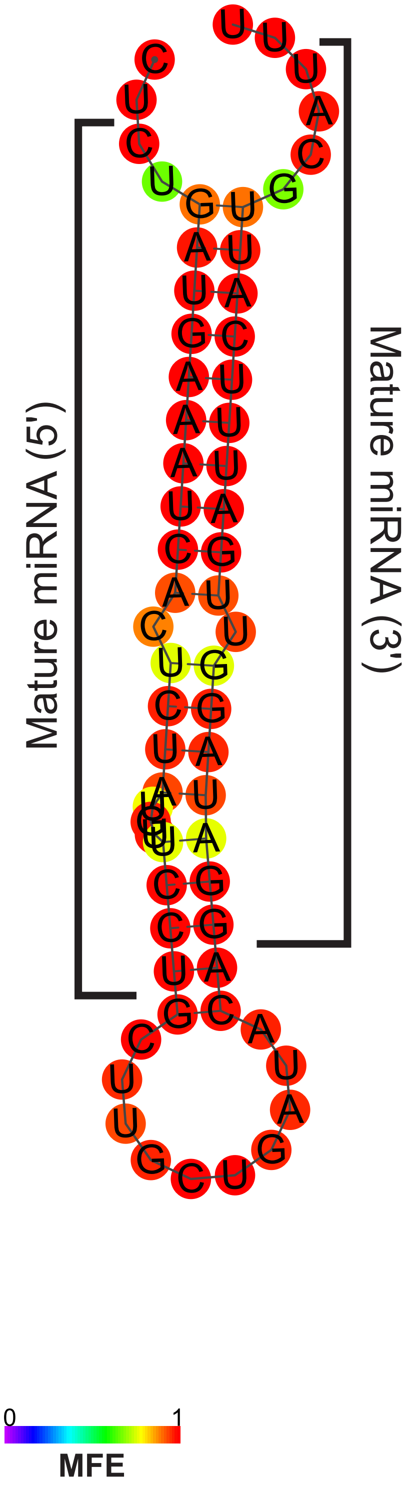

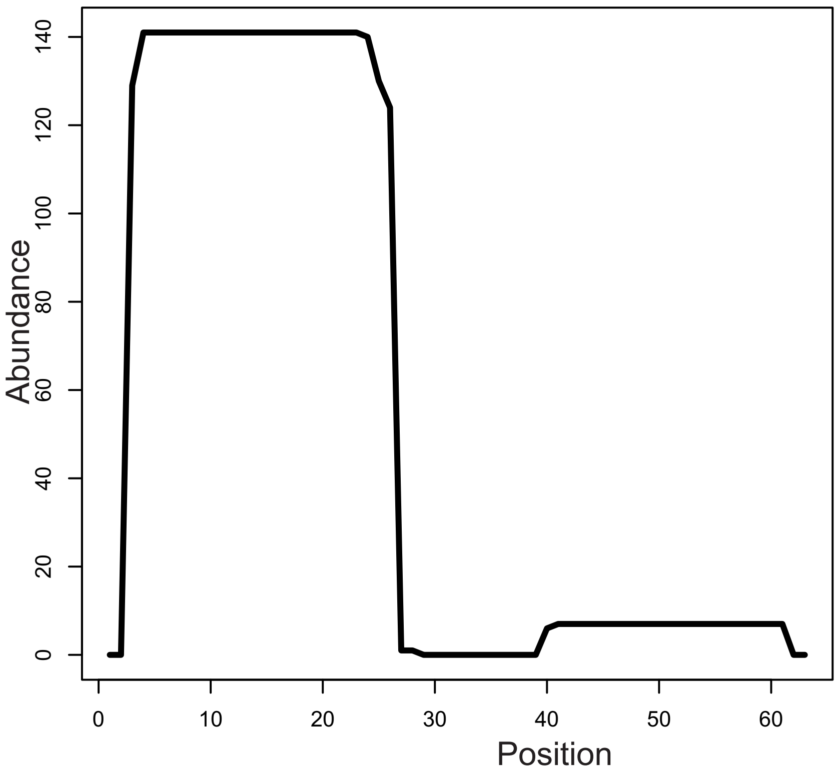


```
CTCTGATGAAATCACTCTATGTTCCTGCTTGCTGATACAGGATAGGTTGATTTCATTGCATTT  Raw reads
..CTGATGAAATCACTCTATGTTCCT.....................................  113
..CTGATGAAATCACTCTATGTTC.......................................  10
...TGATGAAATCACTCTATGTTCCT.....................................  10
.....................................GGATAGGTTGATTTCATTGCAT..  6
..CTGATGAAATCACTCTATGTTCC......................................  5
..CTGATGAAATCACTCTATGTT........................................  1
...TGATGAAATCACTCTATGTTCC......................................  1
...TGATGAAATCACTCTATGTTCCTGC...................................  1
......................................GATAGGTTGATTTCATTGCAT..  1

....((((((((((..(((...(((((..........))))))))..)))))))))).....  (-18.30 MFE)
```

mir-n734

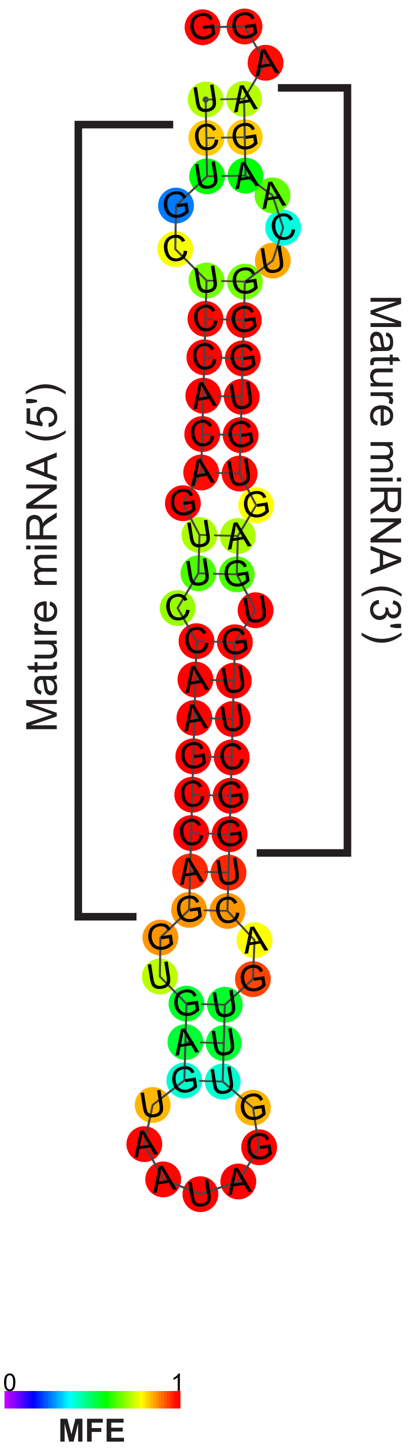

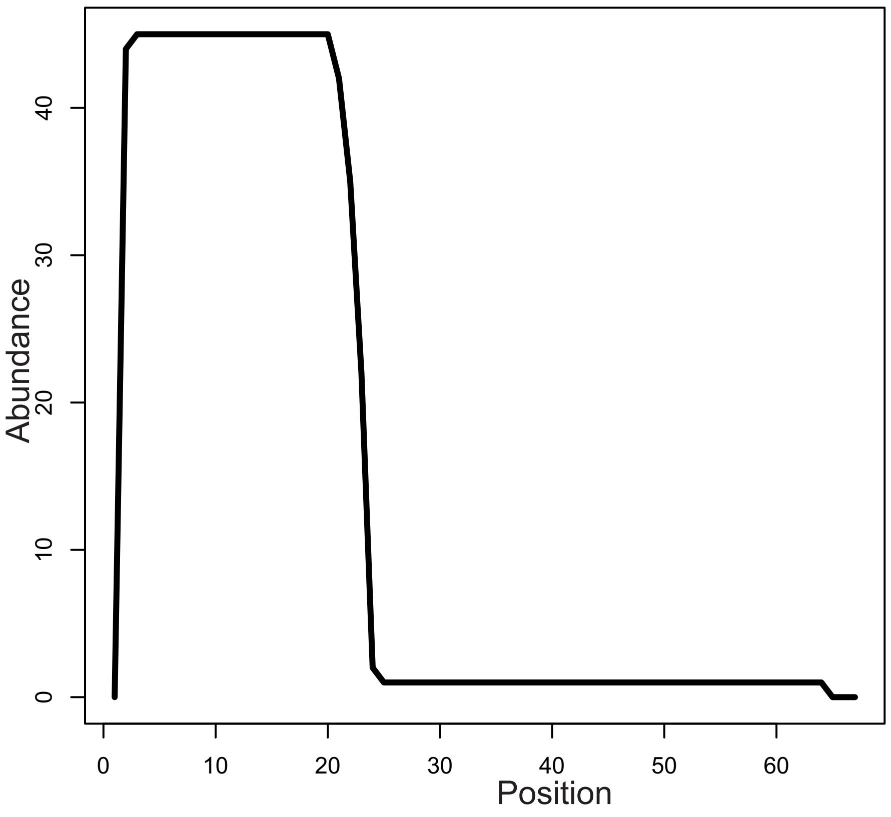


```
TCTGCTCCACAGTTCCAAGCCAGGTGAGTAATAGGTTTGACTGGCTTGTGAGTGTGGGTCAAGAAGG       Raw reads
.CTGCTCCACAGTTCCAAGCCAG............................................       21
.CTGCTCCACAGTTCCAAGCCA.............................................       13
.CTGCTCCACAGTTCCAAGCC..............................................       6
.CTGCTCCACAGTTCCAAGC...............................................       3
..TGCTCCACAGTTCCAAGCC..............................................       1
.CTGCTCCACAGTTCCAAGCCAGG...........................................       1
..........................................GGCTTGTGAGTGTGGGTCAAGA...       1
.......................GTGAGTAATAGGTTTGACT.........................       1

(((..((((((.(..((((((((..(((.......)))..))))))))..).))))))...)))... (-26.70 MFE)
```

mir-n631

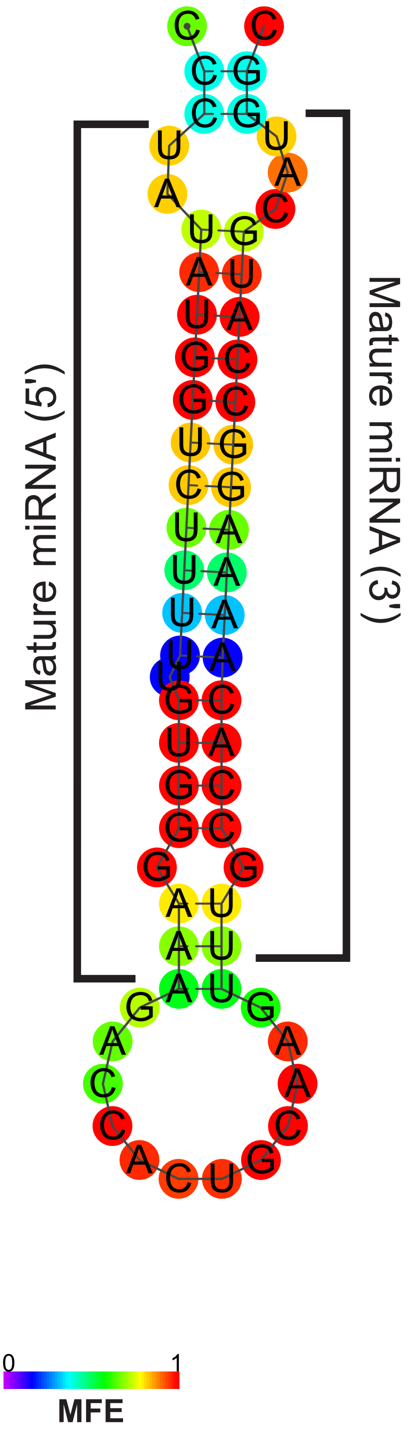

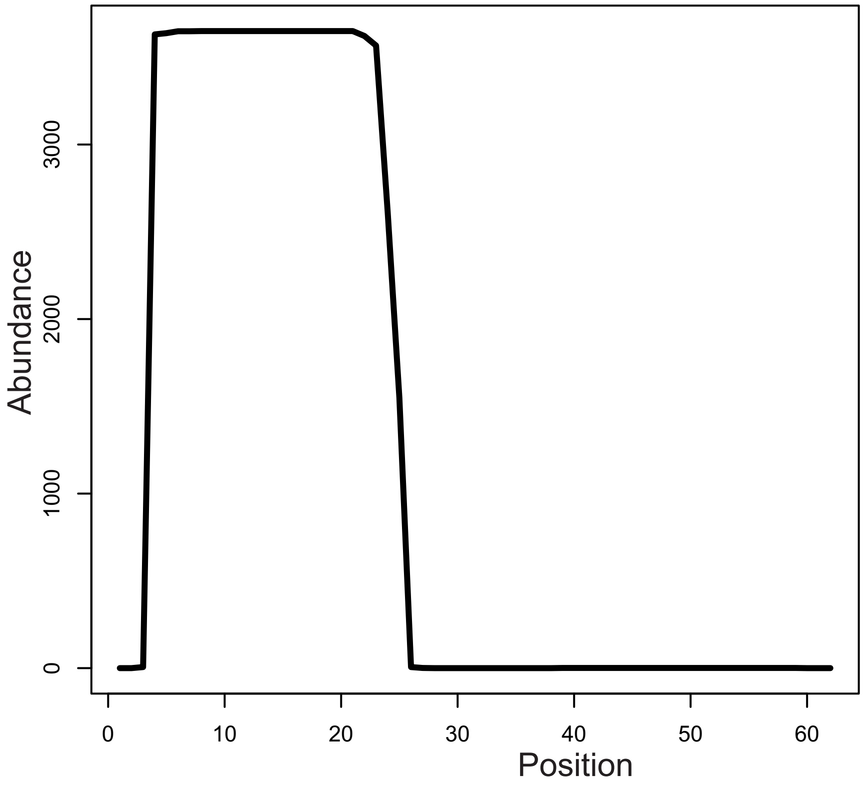


```
CCCTATATGGTCTTTTTGTGGGAAAGACCACTGCAAGTTTGCCACAAAAGGCCATGCATGGC    Raw reads
...TATATGGTCTTTTTGTGGGAAA.....................................    1537
...TATATGGTCTTTTTGTGGGAA......................................    1054
...TATATGGTCTTTTTGTGGGA.......................................    945
...TATATGGTCTTTTTGTGGG........................................    55
...TATATGGTCTTTTTGTGG.........................................    28
....ATATGGTCTTTTTGTGGGA.......................................    6
...TATATGGTCTTTTTGTGGGAAAG....................................    5
.....TATGGTCTTTTTGTGGGA.......................................    4
.....TATGGTCTTTTTGTGGGAAA.....................................    4
..CTATATGGTCTTTTTGTGGGA.......................................    3
.....TATGGTCTTTTTGTGGGAA......................................    3
..CTATATGGTCTTTTTGTGGGAA......................................    2
....ATATGGTCTTTTTGTGGGAA......................................    1
..CTATATGGTCTTTTTGTGGGAAA.....................................    1
.......TGGTCTTTTTGTGGGAAA.....................................    1
...TATATGGTCTTTTTGTGGGAAAGA...................................    1
......................................TTGCCACAAAAGGCCATGCAT...    1

.((..(((((((((((.((((...((((........)))).)))))))))))))))...)). (-21.10 MFE)
```

mir-n784

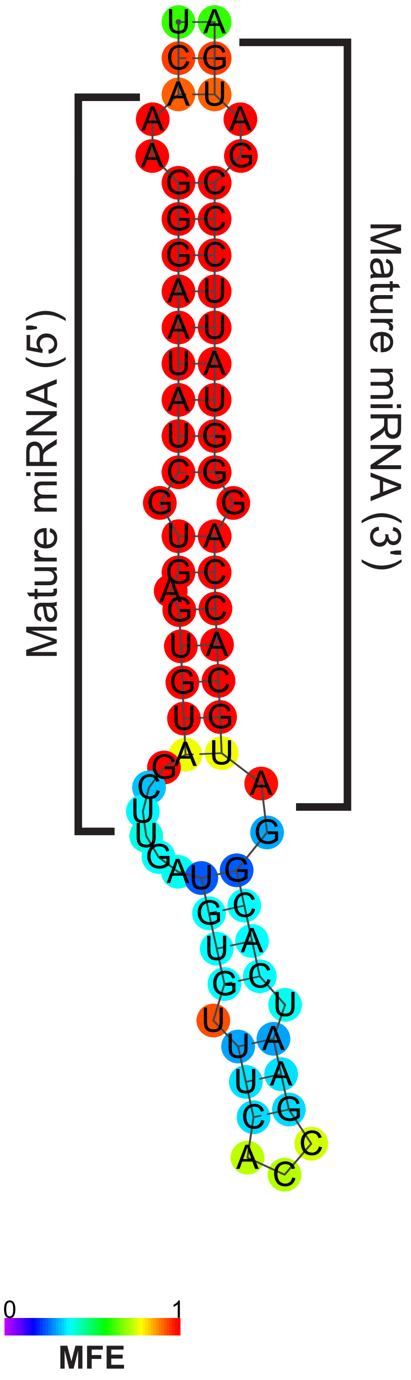

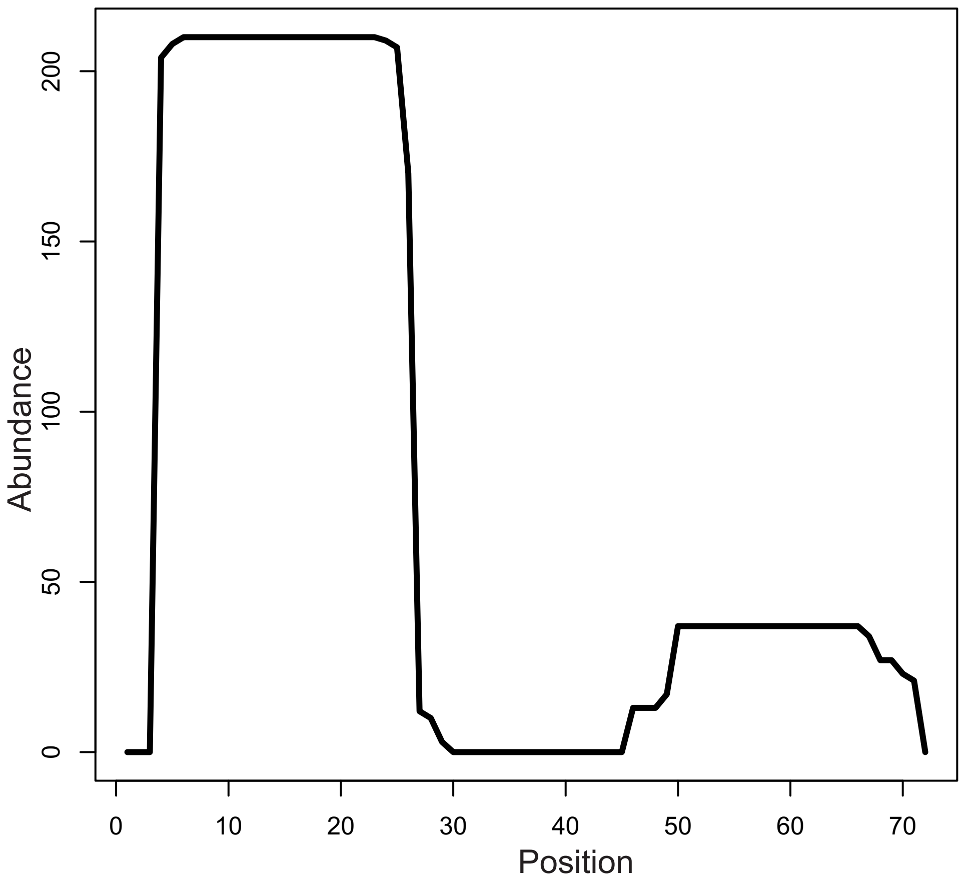


```
TCAAAGGGAATATCGTGAGTGTAGCTTGATGTGTTTCACCGAATCACGGATGCACCAGGGTATTCCCGATGA  Raw reads
...AAGGGAATATCGTGAGTGTAGCT..............................................  154
...AAGGGAATATCGTGAGTGTAGC...............................................  37
.................................................ATGCACCAGGGTATTCCCGATG.  18
...AAGGGAATATCGTGAGTGTAGCTTG............................................  7
.............................................ACGGATGCACCAGGGTATTCCC.....  7
....AGGGAATATCGTGAGTGTAGCT..............................................  4
.............................................ACGGATGCACCAGGGTATTCC......  3
.............................................ACGGATGCACCAGGGTATTCCCGA...  3
................................................GATGCACCAGGGTATTCCCGATG.  3
...AAGGGAATATCGTGAGTGTAG................................................  2
...AAGGGAATATCGTGAGTGTAGCTT.............................................  2
.....GGGAATATCGTGAGTGTAGCTTGA..........................................  2
.................................................ATGCACCAGGGTATTCCCGAT..  2
...AAGGGAATATCGTGAGTGTA.................................................  1
...AAGGGAATATCGTGAGTGTAGCTTGA..........................................  1
................................................GATGCACCAGGGTATTCCCGA...  1

(((..(((((((((.((.(((((......((((.(((...))).))))..))))))).)))))))))..))) (-27.40 MFE)
```

mir-n903

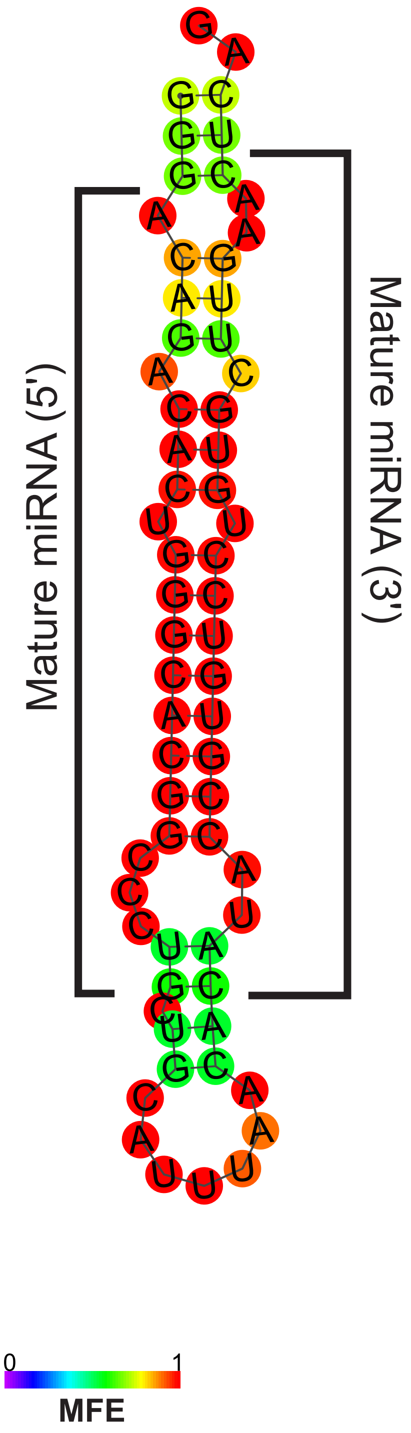

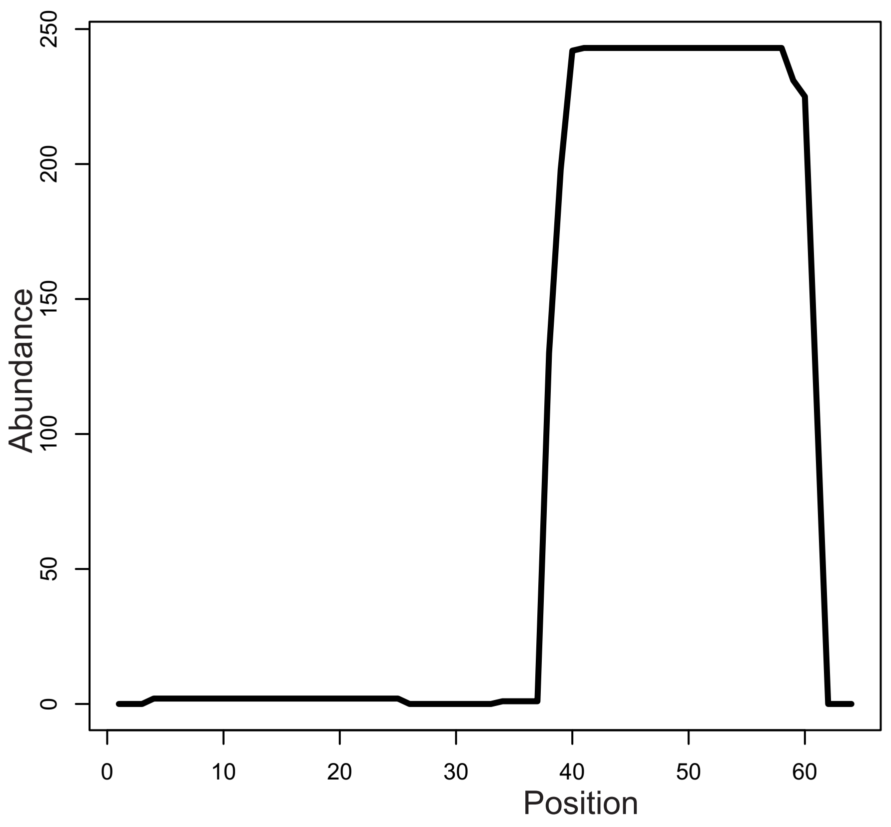


```
GGGACAGACACTGGGCACGGCCCTGCTGCATTTAACACATACCGTGTCCTGTGCTTGAACTCAG  Raw reads
.....................................CATACCGTGTCCTGTGCTTGAAC....  61
.....................................CATACCGTGTCCTGTGCTTGAACT...  56
......................................ATACCGTGTCCTGTGCTTGAAC....  32
......................................ATACCGTGTCCTGTGCTTGAACT...  30
.......................................TACCGTGTCCTGTGCTTGAACT...  25
.......................................TACCGTGTCCTGTGCTTGAAC....  19
.....................................CATACCGTGTCCTGTGCTTGA......  10
......................................ATACCGTGTCCTGTGCTTGAA.....  4
...ACAGACACTGGGCACGGCCCTG.......................................  2
......................................ATACCGTGTCCTGTGCTTGA......  2
.....................................CATACCGTGTCCTGTGCTTGAA.....  2
.................................AACACATACCGTGTCCTGTGCTTGAACT...  1
........................................ACCGTGTCCTGTGCTTGAACT...  1

(((.((..(((.((((((((...((.((.......))))..)))))))).))).))..))).. (-20.60 MFE)
```

mir-n615

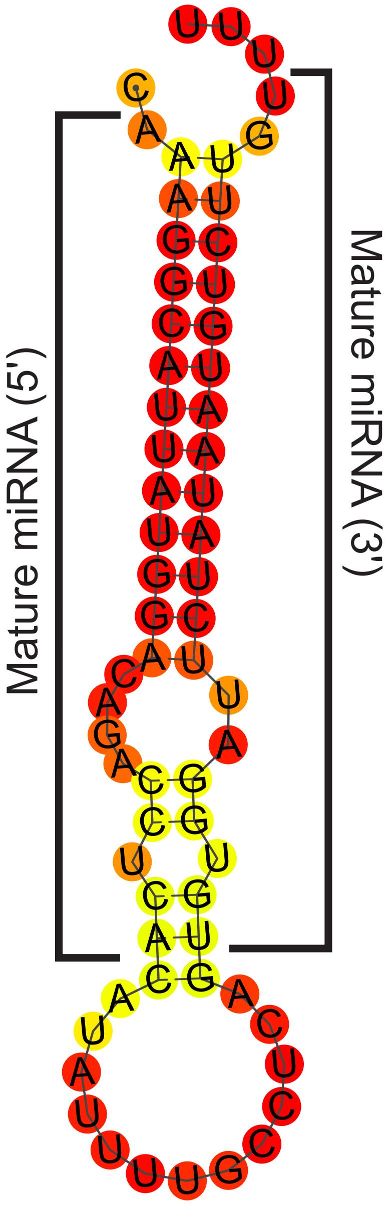

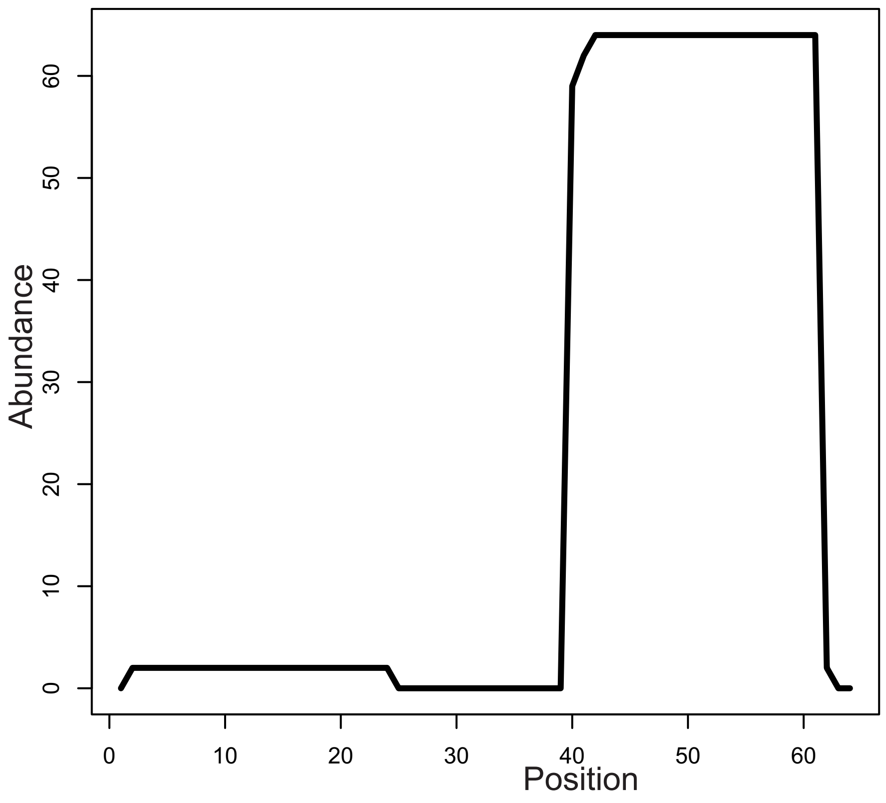


```
CAAAGGCATTATGGACAGACCTCACATATTTTGCCTCAGTGTGGATTCTATAATGTCTTGTTTT  Raw reads
.......................................TGTGGATTCTATAATGTCTTGT...  58
.AAAGGCATTATGGACAGACCTCA........................................  2
........................................GTGGATTCTATAATGTCTTGT...  2
.........................................TGGATTCTATAATGTCTTGT...  2
.......................................TGTGGATTCTATAATGTCTTGTT..  1
........................................GTGGATTCTATAATGTCTTGTT..  1

..(((((((((((((....((.(((.............))).))..))))))))))))).....  (-18.42 MFE)
```

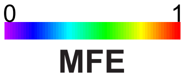

mir-n797

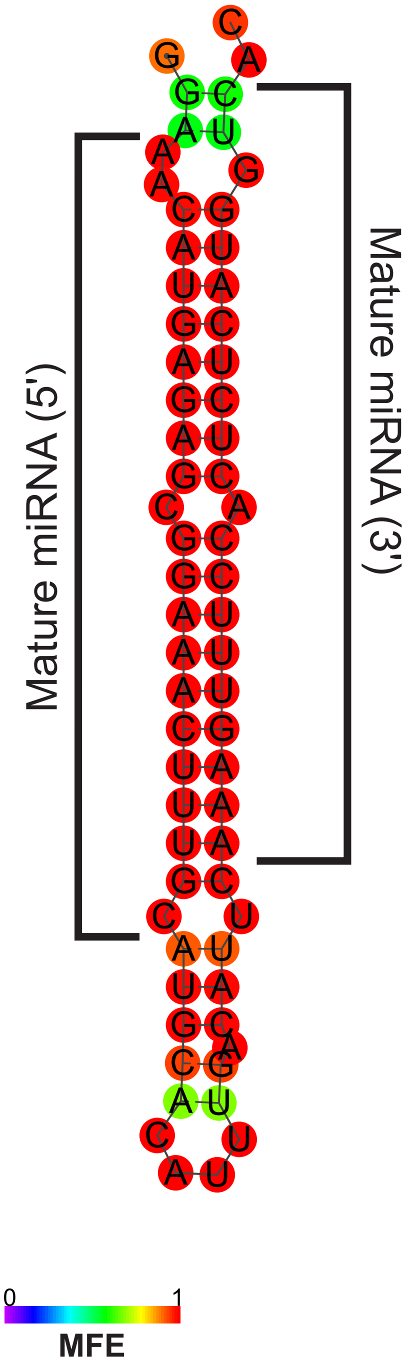

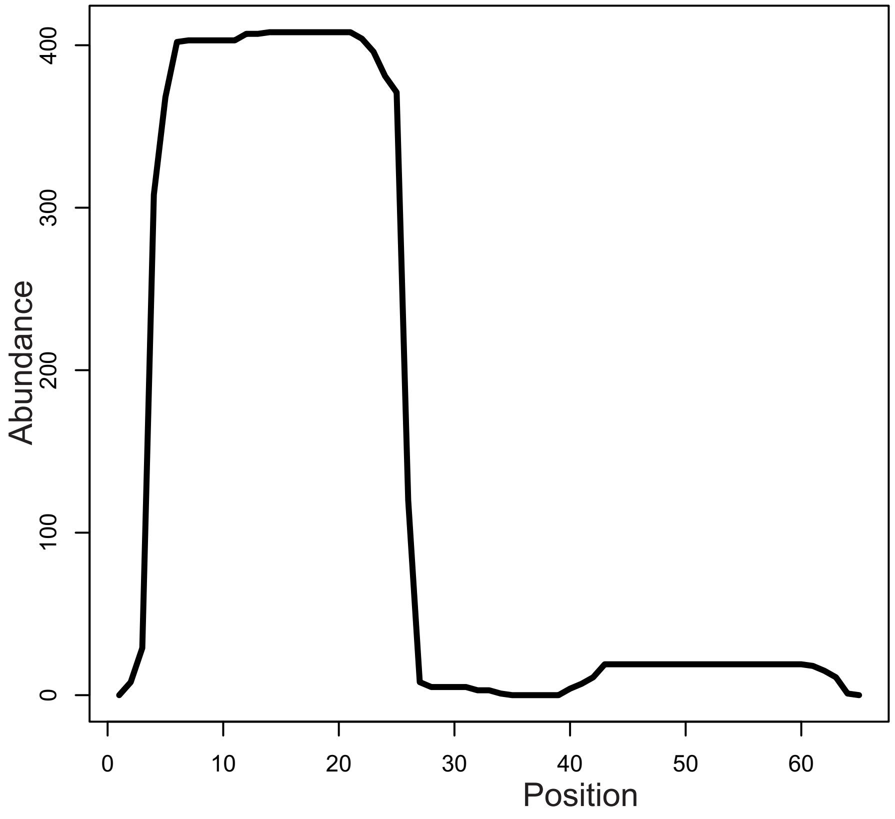


```
GGAAACATGAGAGCGGAAACTTTGCATGCACATTTGACATTCAAAGTTTCCACTCTCATGGTCAC Raw reads
...AACATGAGAGCGGAAACTTTGC........................................ 210
...AACATGAGAGCGGAAACTTTGCA....................................... 51
....ACATGAGAGCGGAAACTTTGCA....................................... 34
.....CATGAGAGCGGAAACTTTGCA....................................... 26
....ACATGAGAGCGGAAACTTTGC........................................ 24
.....CATGAGAGCGGAAACTTTGC........................................ 8
..AAACATGAGAGCGGAAACTTT.......................................... 7
..AAACATGAGAGCGGAAACTTTGC........................................ 7
..........................................AAAGTTTCCACTCTCATGGTC.. 6
...AACATGAGAGCGGAAACTT........................................... 5
..AAACATGAGAGCGGAAACTTTG......................................... 5
...AACATGAGAGCGGAAACT............................................ 4
.GAAACATGAGAGCGGAAACTTT.......................................... 4
...AACATGAGAGCGGAAACTTTG......................................... 4
.........................................CAAAGTTTCCACTCTCATGGTC.. 4
...AACATGAGAGCGGAAACTTT.......................................... 3
.......................................TTCAAAGTTTCCACTCTCATGG.... 3
........................................TCAAAGTTTCCACTCTCATGGT... 3
..AAACATGAGAGCGGAAACTT........................................... 2
.GAAACATGAGAGCGGAAACTTTGC........................................ 2
...AACATGAGAGCGGAAACTTTGCAT...................................... 2
...........AGCGGAAACTTTGCATGCACAT................................ 2
.GAAACATGAGAGCGGAAACTT........................................... 1
....ACATGAGAGCGGAAACTTT.......................................... 1
.GAAACATGAGAGCGGAAACTTTG......................................... 1
......ATGAGAGCGGAAACTTTGCA....................................... 1
....ACATGAGAGCGGAAACTTTGCAT...................................... 1
...........AGCGGAAACTTTGCATGCAC.................................. 1
.............CGGAAACTTTGCATGCAC.................................. 1
...........AGCGGAAACTTTGCATGCACATT............................... 1
..........................................AAAGTTTCCACTCTCATG..... 1
.......................................TTCAAAGTTTCCACTCTCATGGT... 1
..........................................AAAGTTTCCACTCTCATGGTCA. 1

((...((((((((.((((((((((.(((((....)).))).)))))))))).)))))))).)).. (-30.80 MFE)
```

mir-n162

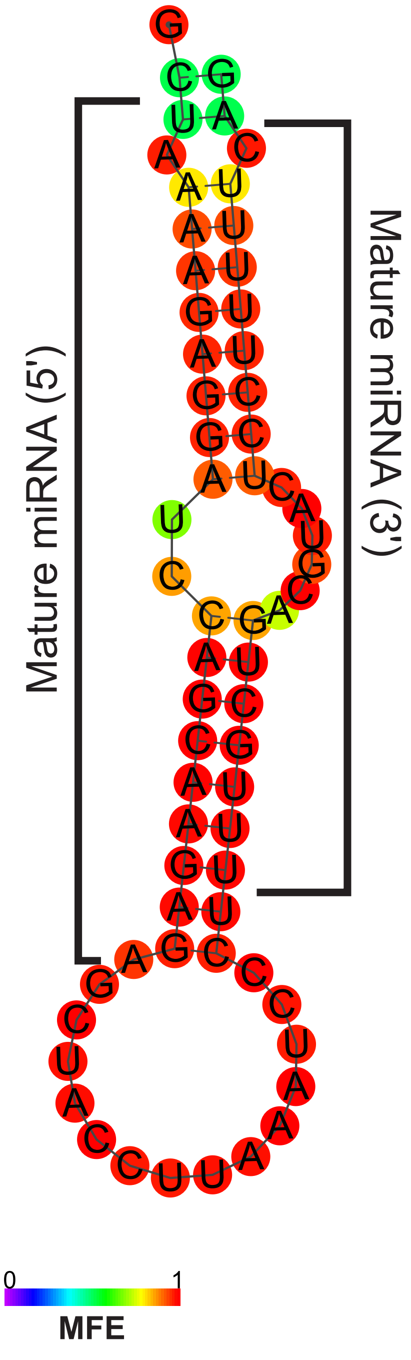

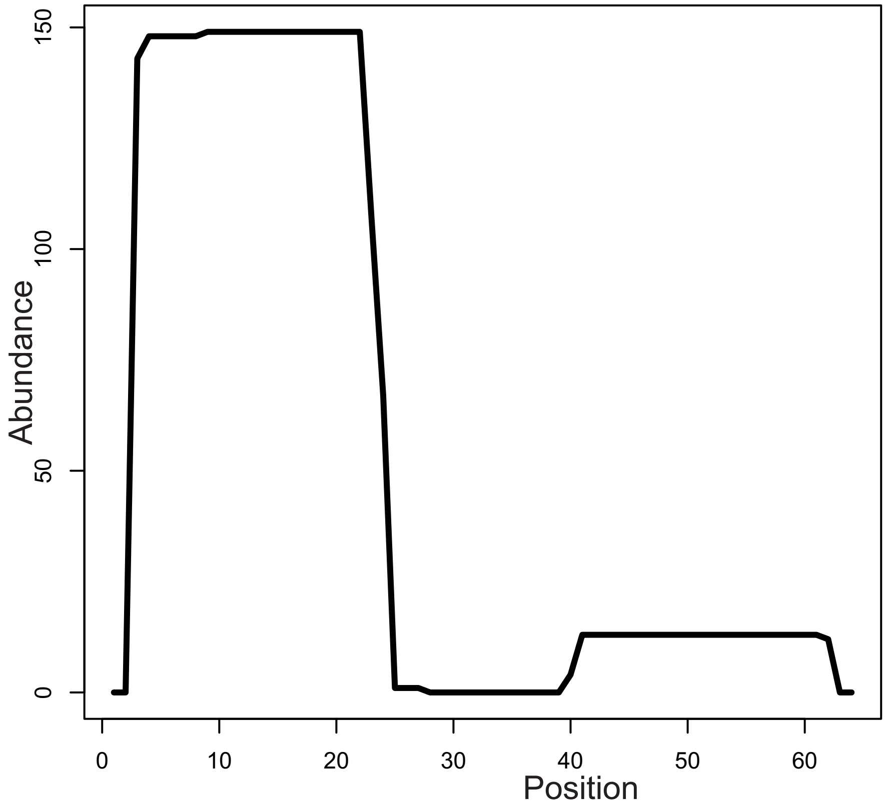


```
GCTAAAAGAGGATCCAGCAAGAGAGCTACCTTAAATCCCTTTTGCTGACGTACTCCTTTTTCAG  Raw reads
..TAAAAGAGGATCCAGCAAGAGA........................................  66
..TAAAAGAGGATCCAGCAAGA..........................................  39
..TAAAAGAGGATCCAGCAAGAG.........................................  38
........................................TTTGCTGACGTACTCCTTTTTC..  8
.......................................TTTTGCTGACGTACTCCTTTTTC..  4
...AAAAGAGGATCCAGCAAGA..........................................  3
...AAAAGAGGATCCAGCAAGAG.........................................  2
........AGGATCCAGCAAGAGAGCT.....................................  1
........................................TTTGCTGACGTACTCCTTTTT...  1

.((.((((((((..(((((((((...............)))))))))......)))))))).)) (-16.26 MFE)
```

mir-n760

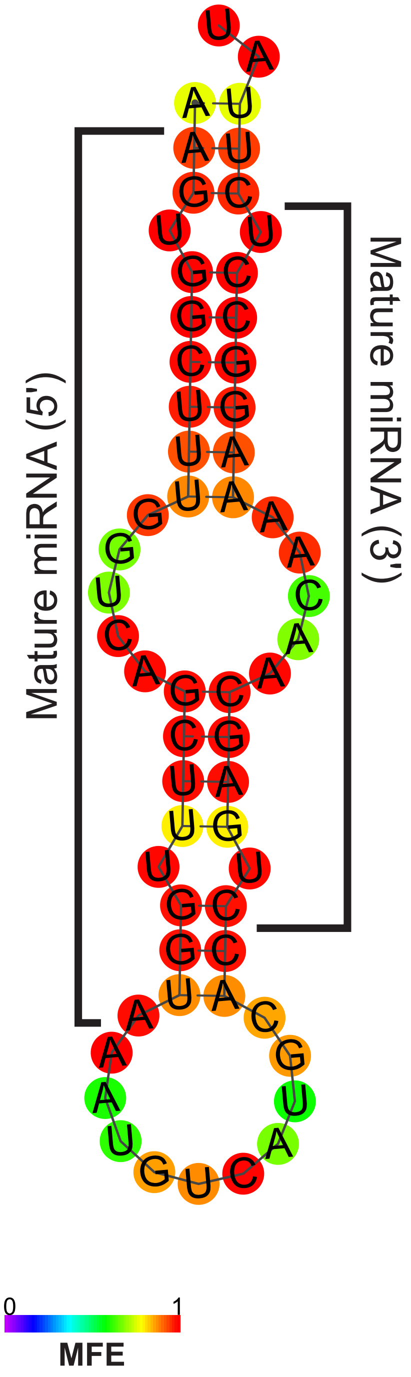

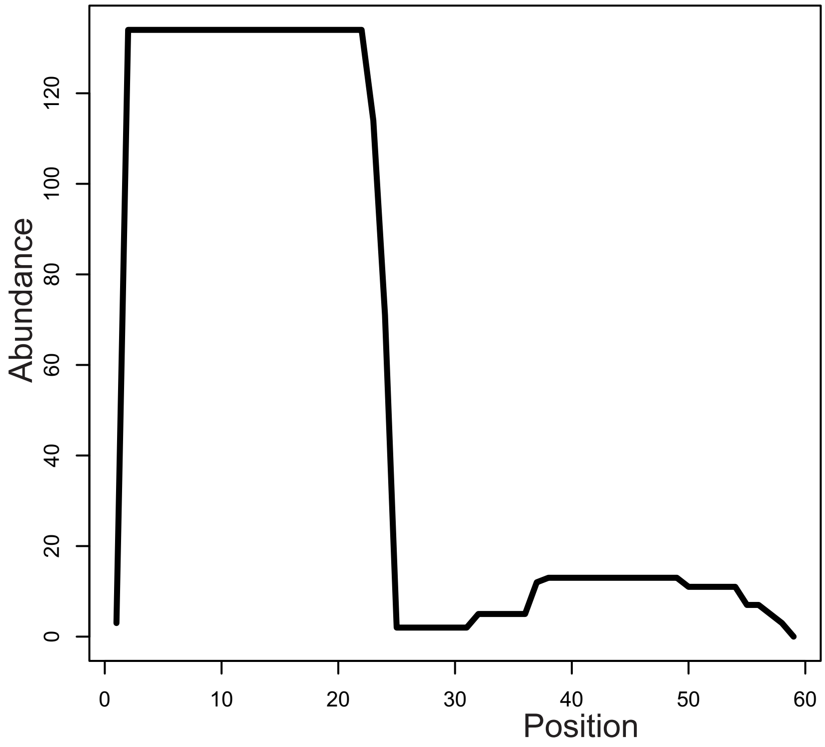


```
AAGTGGCTTTGGTCAGCTTTGGTAAATGTCATGCACCTGAGCAACAAAAGGCCTCTTAT      Raw reads
.AGTGGCTTTGGTCAGCTTTGGTA...................................      68
.AGTGGCTTTGGTCAGCTTTGGT....................................      45
.AGTGGCTTTGGTCAGCTTTGG.....................................      18
....................................CTGAGCAACAAAAGGCCT.....      3
....................................CTGAGCAACAAAAGGCCTCTTA.      3
AAGTGGCTTTGGTCAGCTTTGG.....................................      2
.......................AAATGTCATGCACCTGAGCAACAAAA..........      2
...............................TGCACCTGAGCAACAAAAGGCCTCT...      2
AAGTGGCTTTGGTCAGCTTTGGTA...................................      1
...............................TGCACCTGAGCAACAAAAGGCCT.....      1
....................................CTGAGCAACAAAAGGCCTCTT..      1
.....................................TGAGCAACAAAAGGCCTCTT..      1

(((.((((((.....((((.(((..........))).)))).....)))))).)))..  (-16.60 MFE)
```

mir-n682

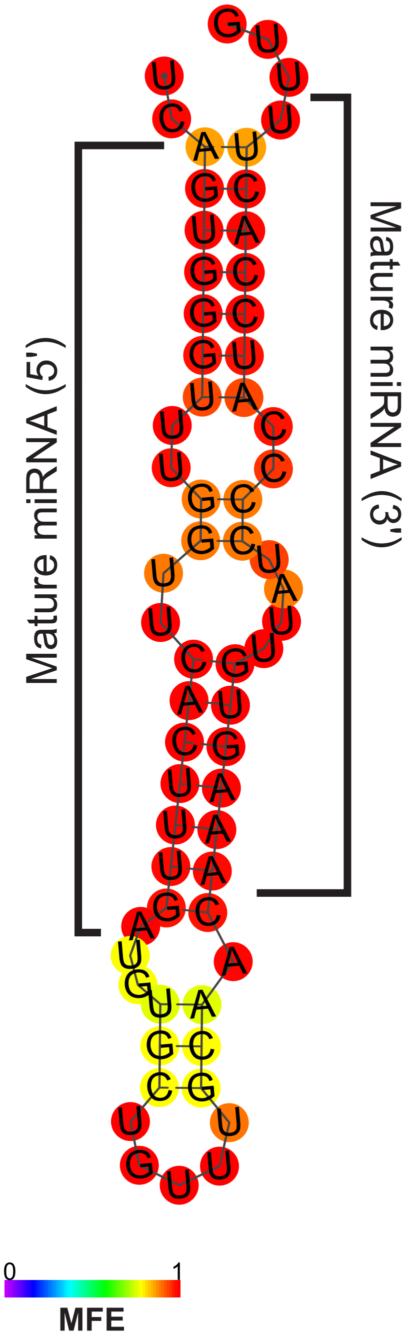

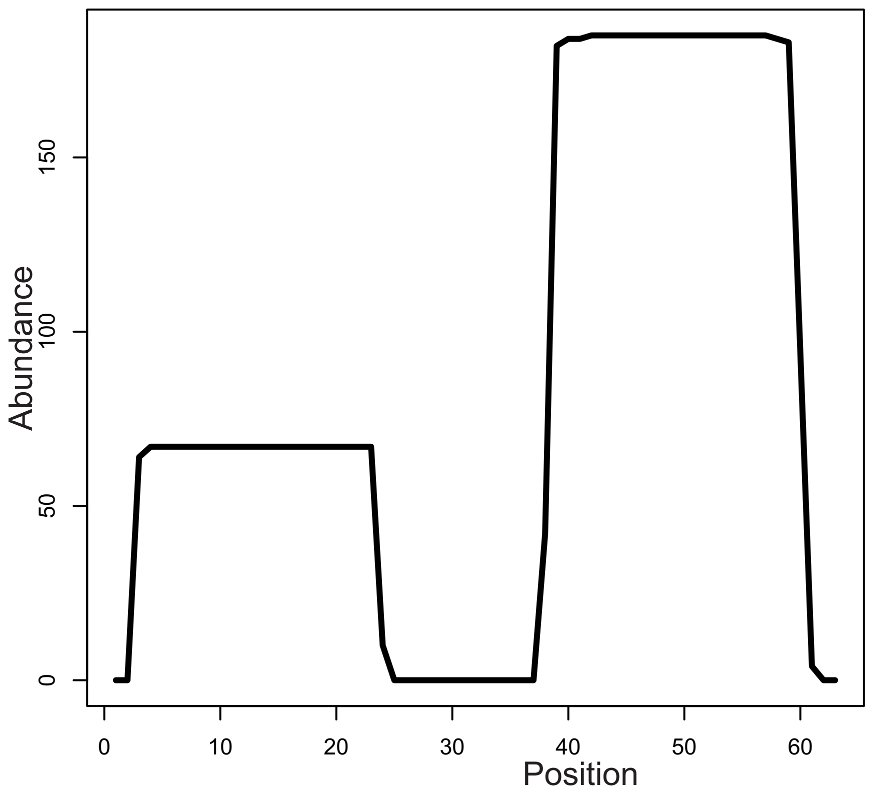


```
TCAGTGGGTTTGGTTCACTTTGATGTGCTGTTTGCAACAAAGTGTTATCCCCATCCACTTTTG  Raw reads
......................................AAAGTGTTATCCCCATCCACTT...  71
......................................AAAGTGTTATCCCCATCCACT....  65
..AGTGGGTTTGGTTCACTTTGA........................................  55
.....................................CAAAGTGTTATCCCCATCCACT....  23
.....................................CAAAGTGTTATCCCCATCCACTT...  18
..AGTGGGTTTGGTTCACTTTGAT.......................................  9
...GTGGGTTTGGTTCACTTTGA........................................  2
......................................AAAGTGTTATCCCCATCCACTTT..  2
...GTGGGTTTGGTTCACTTTGAT.......................................  1
......................................AAAGTGTTATCCCCATCCA......  1
......................................AAAGTGTTATCCCCATCCAC.....  1
.......................................AAGTGTTATCCCCATCCACT....  1
.........................................GTGTTATCCCCATCCACTT...  1
.....................................CAAAGTGTTATCCCCATCCACTTT..  1
.......................................AAGTGTTATCCCCATCCACTTT..  1

..(((((((..((..(((((((...(((.....))).)))))))....))..))))))).... (-22.00 MFE)
```

mir-n547

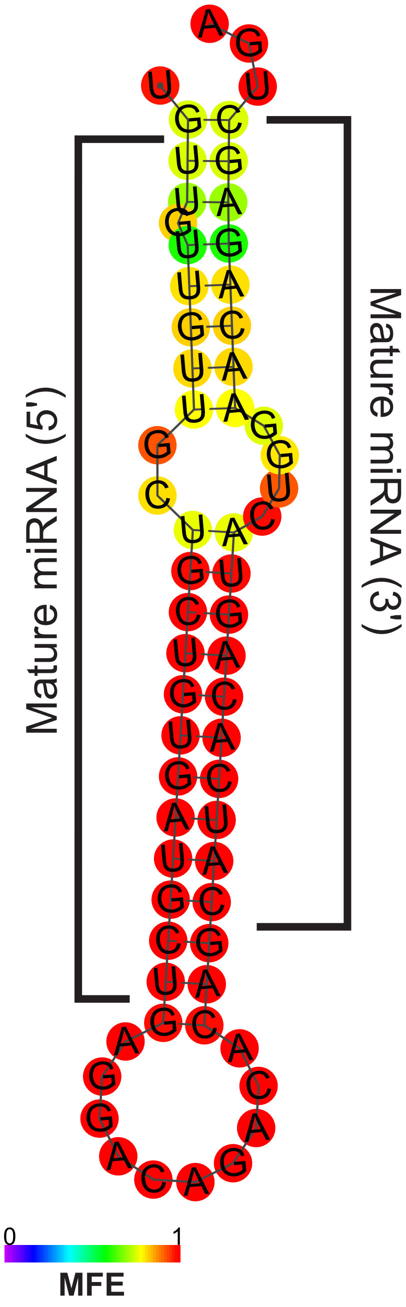

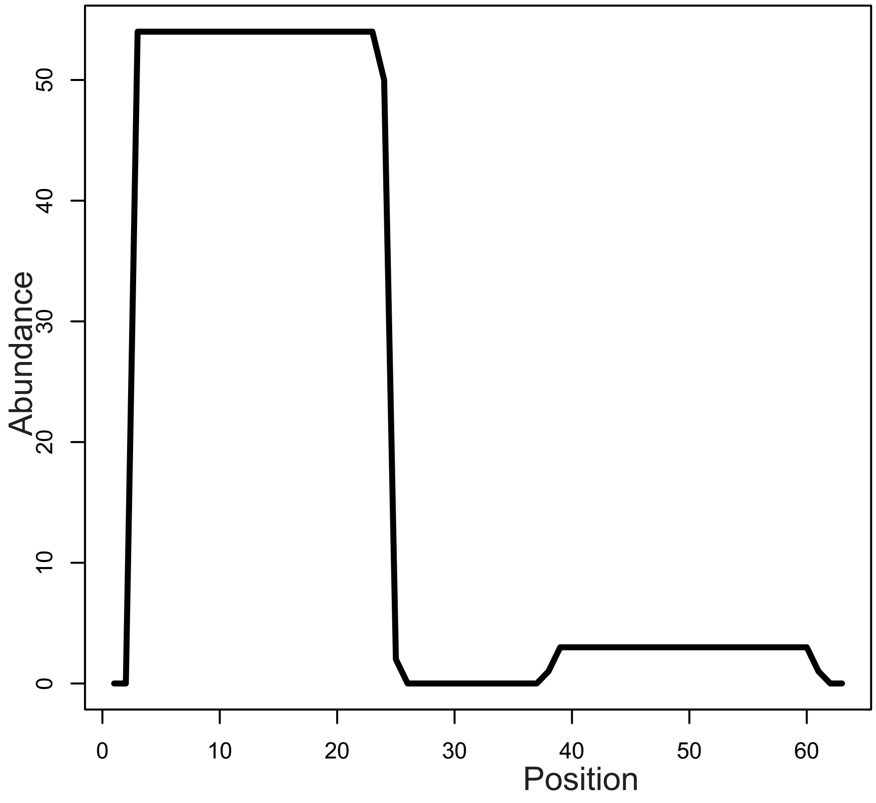


```
TGTTGTTGTTGCTGCTGTGATGCTGAGGACAGACACAGCATCACAGTACTGGAACAGAGCTGA   Raw reads
..TTGTTGTTGCTGCTGTGATGCT.......................................   48
..TTGTTGTTGCTGCTGTGATGC........................................   4
..TTGTTGTTGCTGCTGTGATGCTG......................................   2
......................................CATCACAGTACTGGAACAGAGC...   2
.....................................GCATCACAGTACTGGAACAGAGCT..   1

.(((.(((((.((((((((((((((.(......).)))))))))))))..).)))))))))... (-27.10 MFE)
```

mir-n072

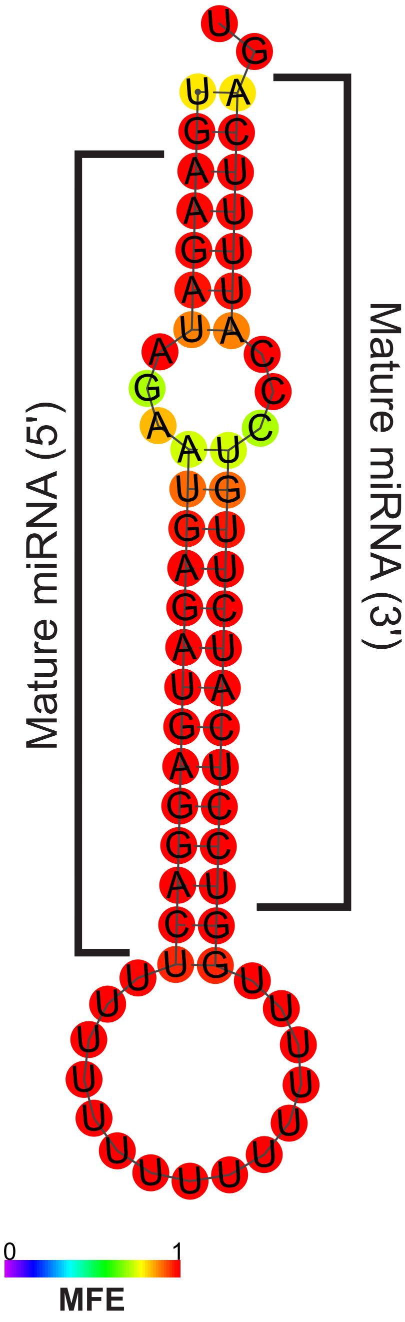

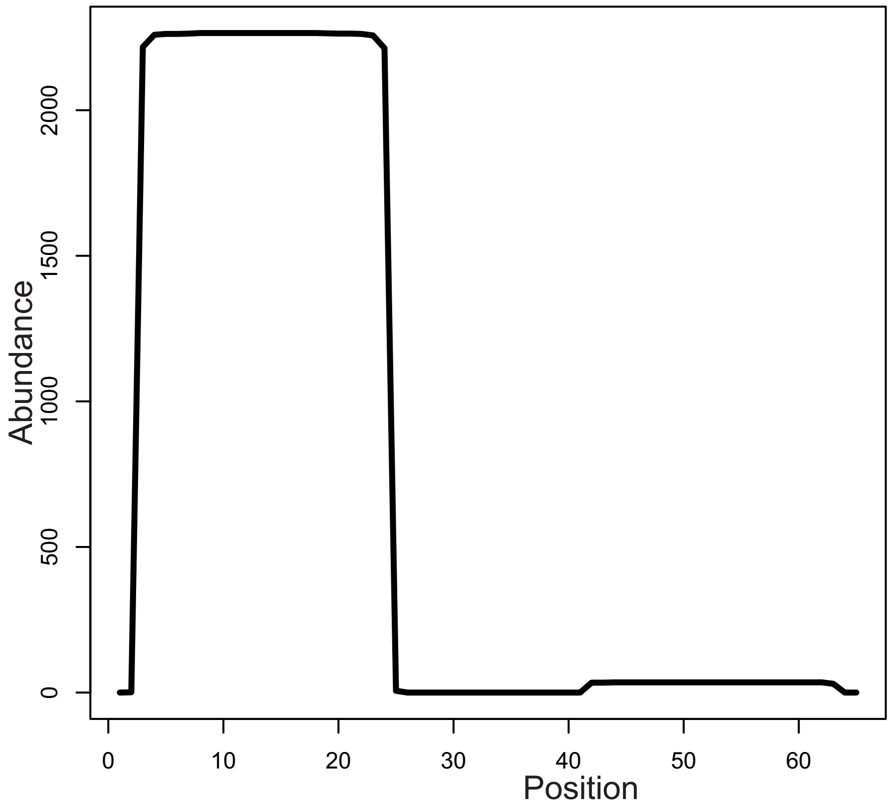


```
TGAAGATAGAATGAGATGAGGACTTTTTTTTTTTTTTTTGGTCCTCATCTTGTCCCATTTTCAGT Raw reads
..AAGATAGAATGAGATGAGGACT......................................... 2161
..AAGATAGAATGAGATGAGGAC.......................................... 42
...AGATAGAATGAGATGAGGACT......................................... 40
.........................................TCCTCATCTTGTCCCATTTTCA.. 29
..AAGATAGAATGAGATGAGGACTT........................................ 6
..AAGATAGAATGAGATGAGGA........................................... 5
.........................................TCCTCATCTTGTCCCATTTTC... 5
....GATAGAATGAGATGAGGACT......................................... 3
...AGATAGAATGAGATGAGGAC.......................................... 2
.......AGAATGAGATGAGGACT......................................... 2
..AAGATAGAATGAGATG............................................... 1
.GAAGATAGAATGAGATGA.............................................. 1
..AAGATAGAATGAGATGAGG............................................ 1
......TAGAATGAGATGAGGACT......................................... 1
...........................................CTCATCTTGTCCCATTTTCA.. 1

(((((((.(.(..(((((((((((...............)))))))))))..).).))))))).. (-25.06 MFE)
```

mir-n852

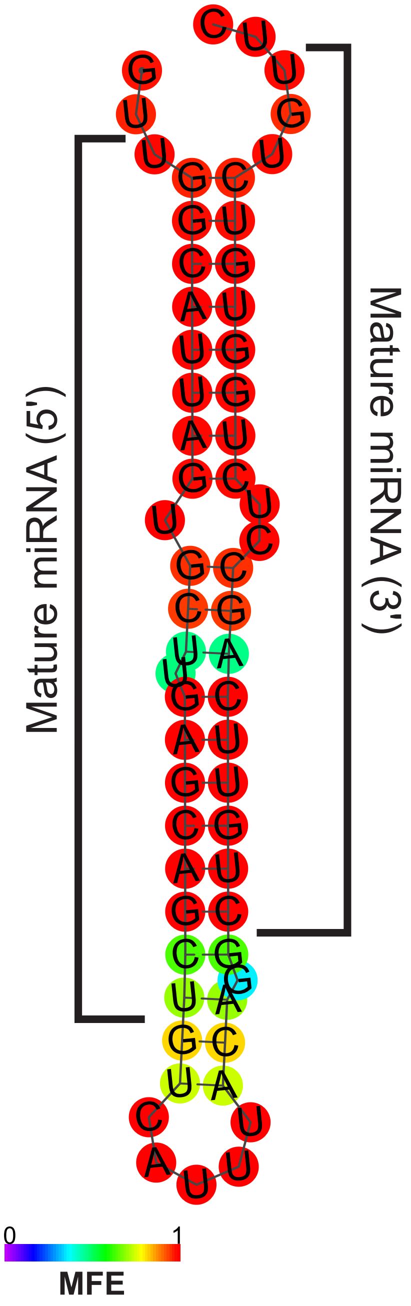

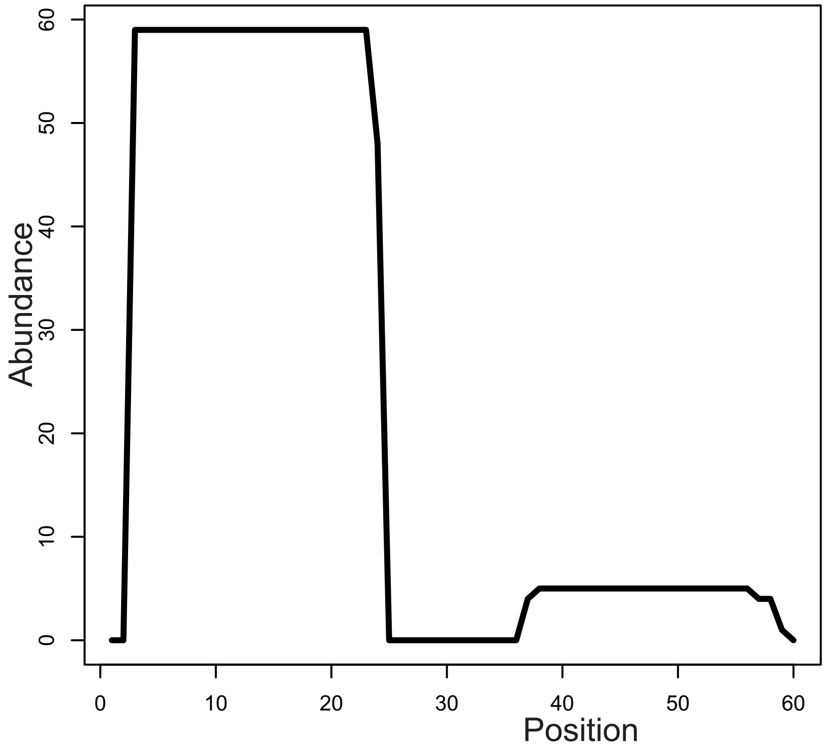


```
GTTGGCATTAGTGCTTGAGCAGCTGTCATTTACAGGCTGTTCAGCCTCTGGTGTCTGTTC      Raw reads
..TGGCATTAGTGCTTGAGCAGCT....................................      48
..TGGCATTAGTGCTTGAGCAGC.....................................      11
....................................CTGTTCAGCCTCTGGTGTCTGT..      2
....................................CTGTTCAGCCTCTGGTGTCT....      1
.....................................TGTTCAGCCTCTGGTGTCTGT..      1
....................................CTGTTCAGCCTCTGGTGTCTGTT.      1

...((((((((.(((.((((((((((.....))).))))))))))..)))))))).....  (-25.00 MFE)
```

mir-n015

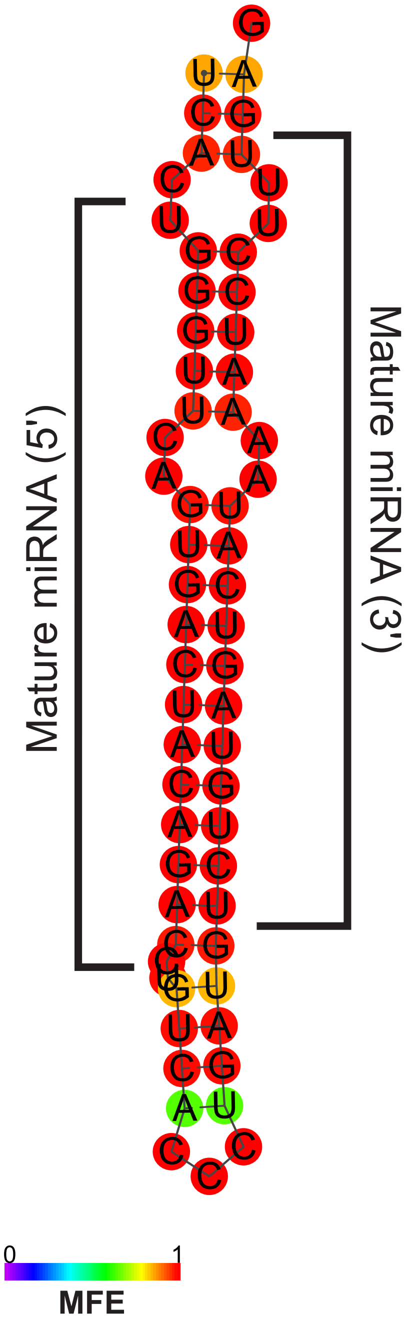

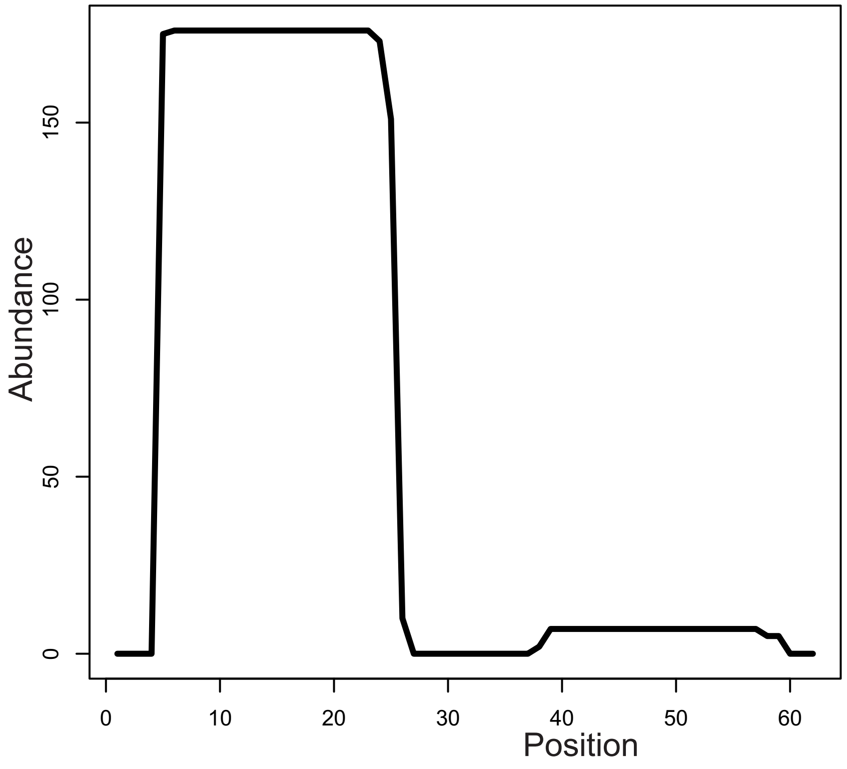


```
TCACTGGGTTCAGTGACTACAGACCTGTCACCCTGATGTCTGTAGTCATAAAATCCTTTGAG   Raw reads
....TGGGTTCAGTGACTACAGACC.....................................   141
....TGGGTTCAGTGACTACAGAC......................................   21
....TGGGTTCAGTGACTACAGACCT....................................   10
......................................TCTGTAGTCATAAAATCCTTT...   4
....TGGGTTCAGTGACTACAGA.......................................   3
.....GGGTTCAGTGACTACAGAC......................................   1
.....................................GTCTGTAGTCATAAAATCCT.....   1
......................................TCTGTAGTCATAAAATCCT.....   1
.....................................GTCTGTAGTCATAAAATCCTTT...   1

(((..(((((..((((((((((((..(((.....)))))))))))))))..)))))..))). (-27.90 MFE)
```

mir-n246

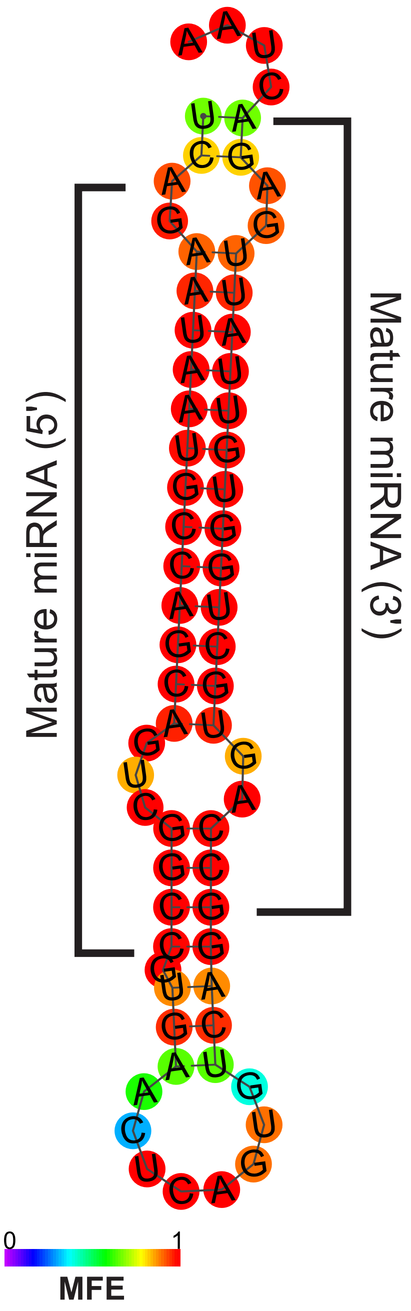

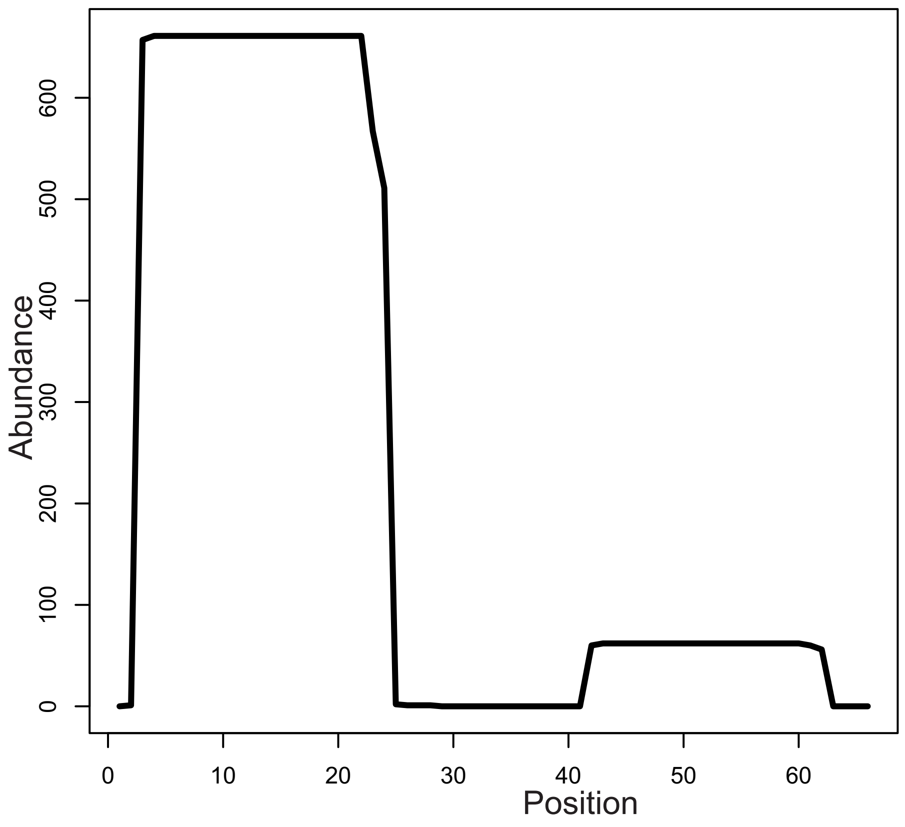


```
TCAGAATAATGCCAGCAGTCGGCCGTGAACTCAGTGTCAGGCCAGTGCTGGTGTTATTGAGACTAA       Raw reads
..AGAATAATGCCAGCAGTCGGCC..........................................       505
..AGAATAATGCCAGCAGTCGG............................................       93
..AGAATAATGCCAGCAGTCGGC...........................................       56
.........................................CCAGTGCTGGTGTTATTGAGA....       54
.........................................CCAGTGCTGGTGTTATTGAG.....       4
...GAATAATGCCAGCAGTCGGCC..........................................       3
.........................................CCAGTGCTGGTGTTATTGA......       2
..........................................CAGTGCTGGTGTTATTGAGA....       2
...GAATAATGCCAGCAGTCGG............................................       1
.CAGAATAATGCCAGCAGTCGGCC..........................................       1
..AGAATAATGCCAGCAGTCGGCCG.........................................       1
..AGAATAATGCCAGCAGTCGGCCGTGA......................................       1

((..((((((((((((.(.((((.(((.(.....))))))))).)))))))))))))..)).... (-28.10 MFE)
```

mir-n576

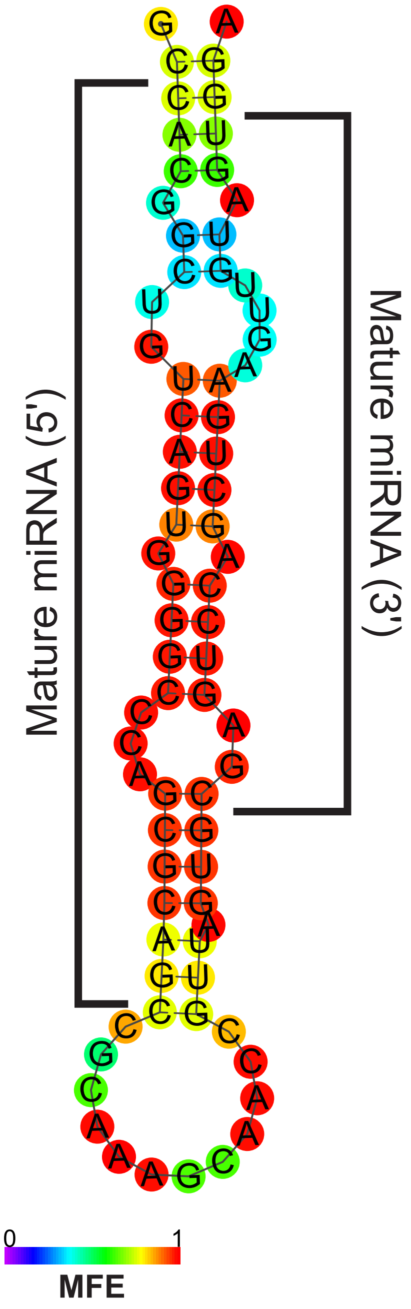

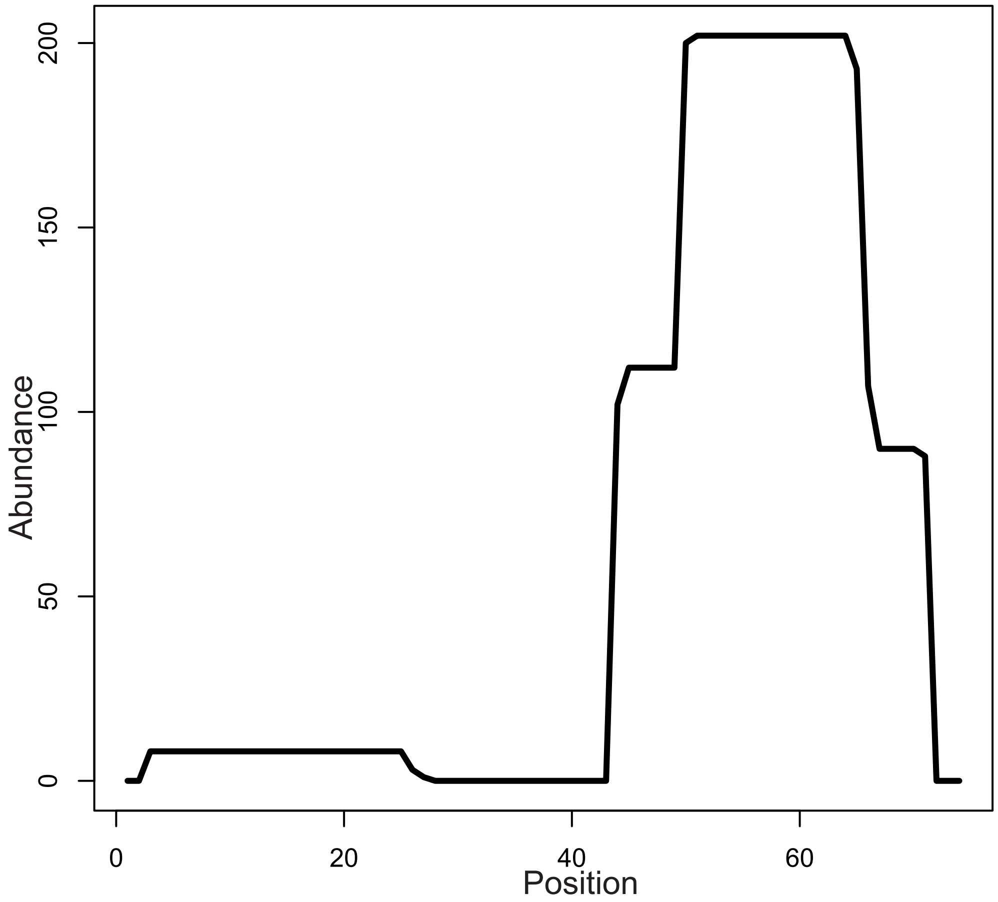


```
GCCACGGCTGTCAGTGGGGCCCAGCGCAGCCGCAAAGCAACCGTTAGTGCGAGTCCAGCTGAAGTTGTAGTGGA        Raw reads
.................................................CGAGTCCAGCTGAAGTTGTAGT...        87
...........................................TTAGTGCGAGTCCAGCTGAAGT.........        78
...........................................TTAGTGCGAGTCCAGCTGAAGTT........        15
...........................................TTAGTGCGAGTCCAGCTGAAG..........        9
............................................TAGTGCGAGTCCAGCTGAAGT.........        8
..CACGGCTGTCAGTGGGGCCCAGC.................................................        5
..CACGGCTGTCAGTGGGGCCCAGCG................................................        2
............................................TAGTGCGAGTCCAGCTGAAGTT........        2
..CACGGCTGTCAGTGGGGCCCAGCGC...............................................        1
.................................................CGAGTCCAGCTGAAGTTGTAG....        1
..................................................GAGTCCAGCTGAAGTTGTAG....        1
..................................................GAGTCCAGCTGAAGTTGTAGT...        1

.((((.((..(((((.((((...(((((((.(........).))).))))..)))).)))))....)).)))). (-26.30 MFE)
```

mir-n294

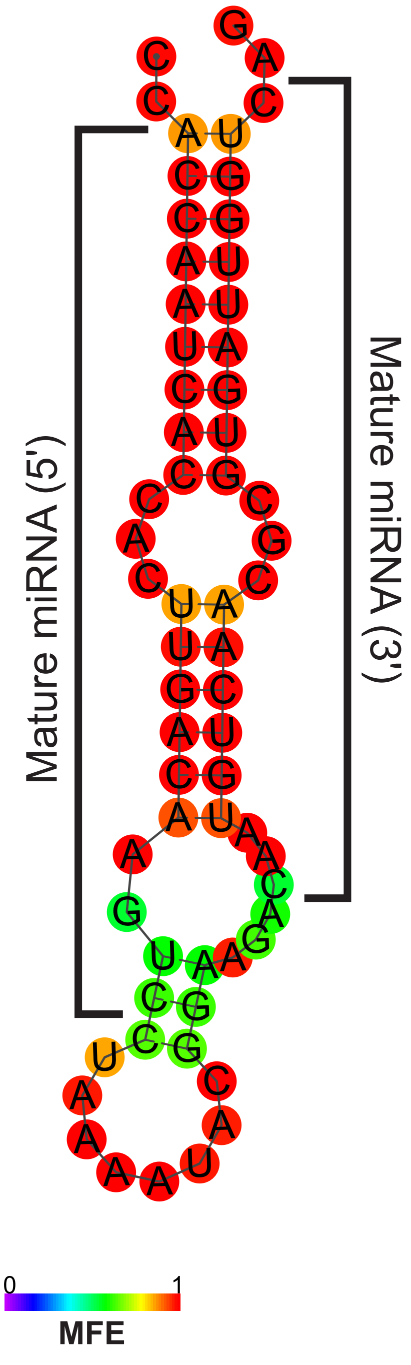

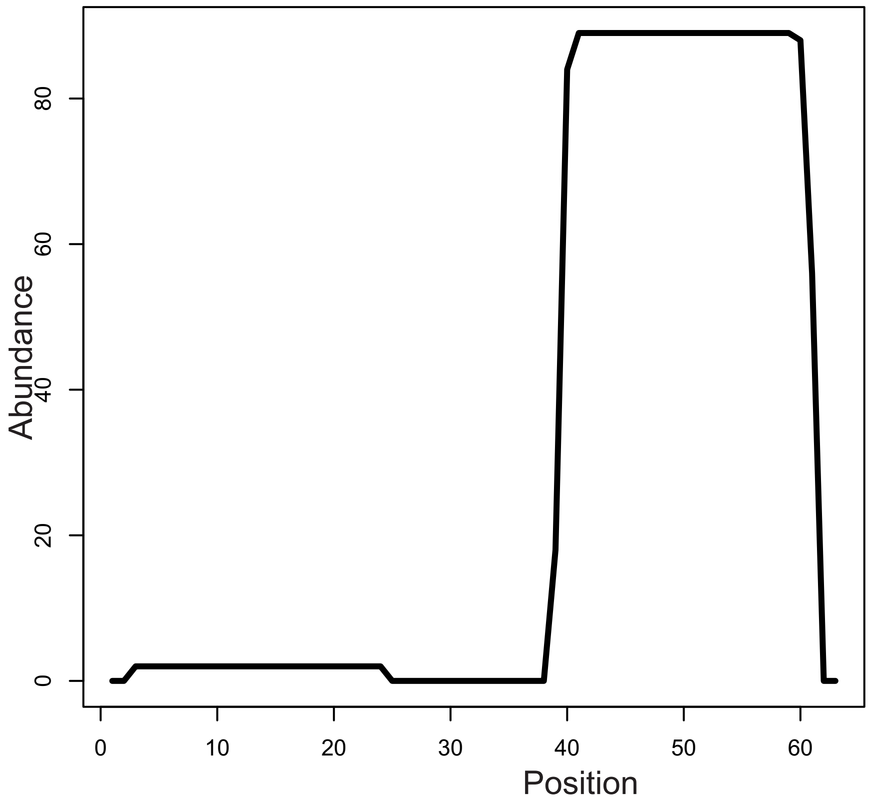


```
CCACCAATCACCACTTGACAAGTCCTAAAATACGGAAGACAATGTCAACGCGTGATTGGTCAG   Raw reads
.......................................CAATGTCAACGCGTGATTGGTC..   52
......................................ACAATGTCAACGCGTGATTGGT...   18
.......................................CAATGTCAACGCGTGATTGGT...   13
........................................AATGTCAACGCGTGATTGGTC..   4
..ACCAATCACCACTTGACAAGTC.......................................   2
.......................................CAATGTCAACGCGTGATTGG....   1
........................................AATGTCAACGCGTGATTGGT...   1

..(((((((((...((((((..(((........)))......))))))...)))))))))... (-20.10 MFE)
```

mir-n886

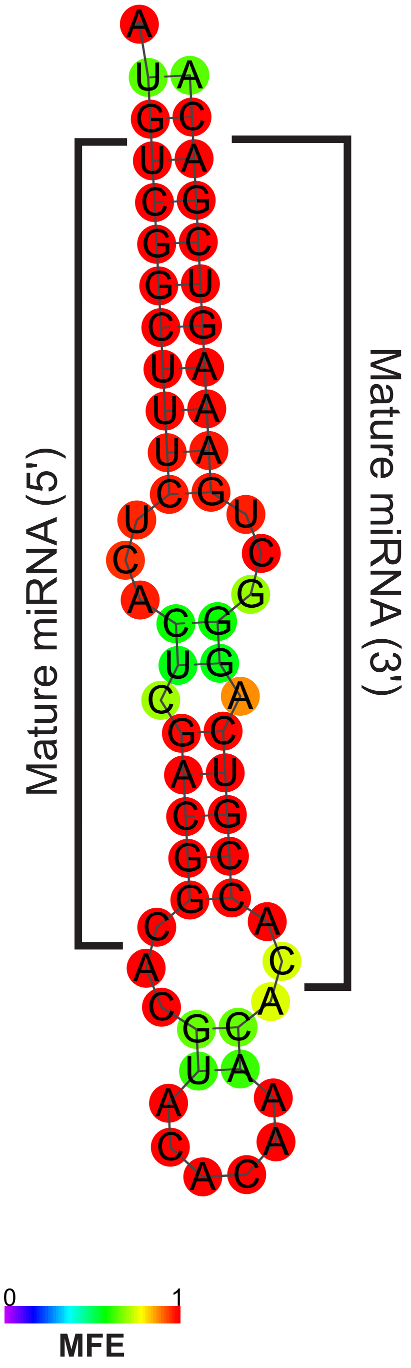

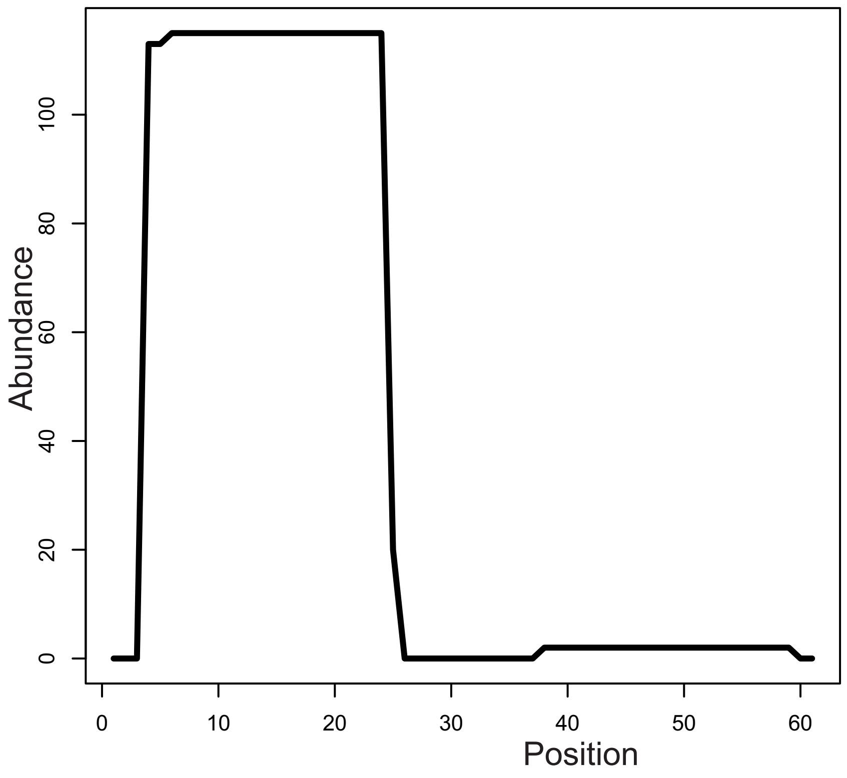


```
ATGTCGGCTTTCTCACTCGACGGCACGTACACAAACACACCGTCAGGGCTGAAAGTCGACA    Raw reads
...TCGGCTTTCTCACTCGACGGC.....................................    94
...TCGGCTTTCTCACTCGACGGCA....................................    19
.....................................CACCGTCAGGGCTGAAAGTCGA..    2
.....GGCTTTCTCACTCGACGGC.....................................    1
.....GGCTTTCTCACTCGACGGCA....................................    1

.(((((((((((...(..(((((...((......))...)))))..)...))))))))))) (-25.70)
```

mir-n801

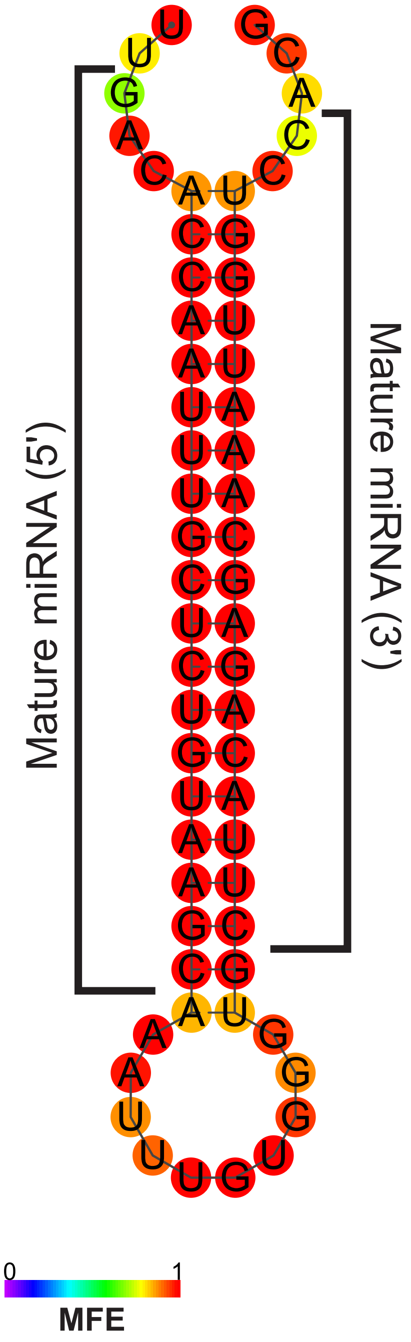

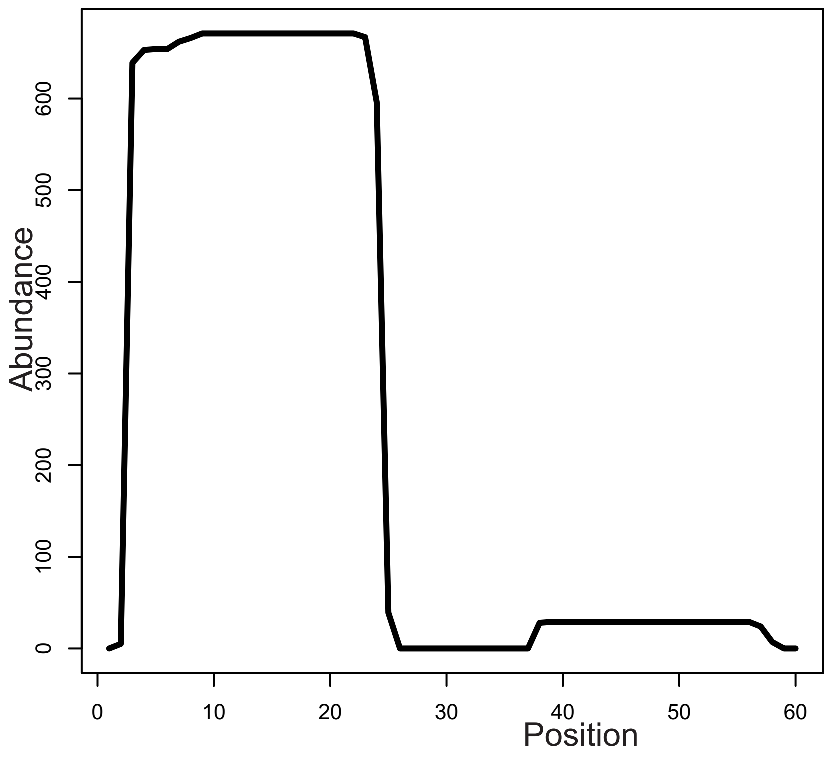


```
TTGACACCAATTTGCTCTGTAAGCAAATTTGTGGGTGCTTACAGAGCAAATTGGTCCACG      Raw reads
..GACACCAATTTGCTCTGTAAGC....................................      529
..GACACCAATTTGCTCTGTAAG.....................................      68
..GACACCAATTTGCTCTGTAAGCA...................................      33
.....................................CTTACAGAGCAAATTGGTCC...      17
...ACACCAATTTGCTCTGTAAGC....................................      8
......CCAATTTGCTCTGTAAGC....................................      7
.....................................CTTACAGAGCAAATTGGTCCA..      6
........AATTTGCTCTGTAAGC....................................      5
.....................................CTTACAGAGCAAATTGGTC....      5
..GACACCAATTTGCTCTGTAA......................................      4
.TGACACCAATTTGCTCTGTAAGC....................................      4
.......CAATTTGCTCTGTAAGC....................................      4
...ACACCAATTTGCTCTGTAAGCA...................................      4
...ACACCAATTTGCTCTGTAAG.....................................      2
....CACCAATTTGCTCTGTAAG.....................................      1
.TGACACCAATTTGCTCTGTAAGCA...................................      1
......CCAATTTGCTCTGTAAGCA...................................      1
......................................TTACAGAGCAAATTGGTCCA..      1

.....((((((((((((((((((((..(....)..)))))))))))))))))))))..... (-32.40 MFE)
```

mir-487

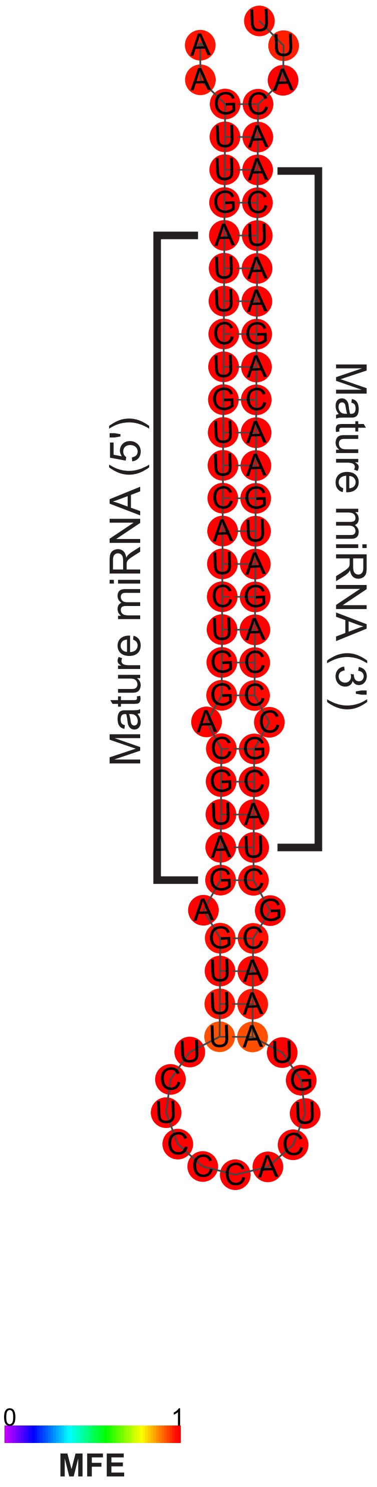

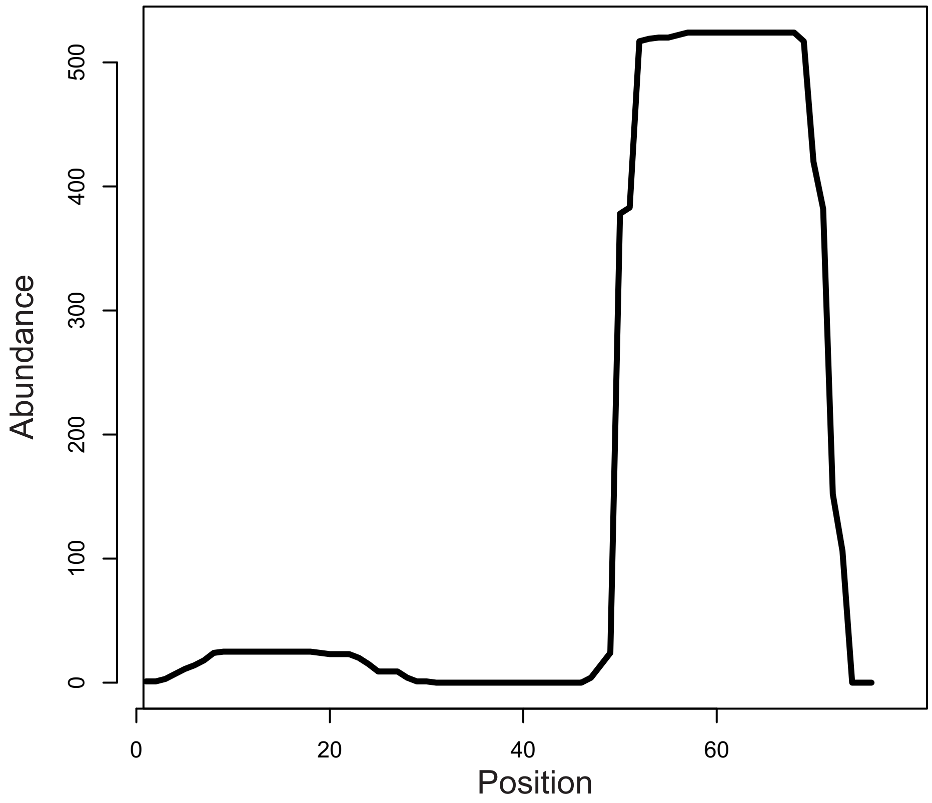


```
AAGTTGATTCTGTTCATCTGGACGTAGAGTTTTCTCCCACTGTAAACGCTACGCCCAGATGAACAGAATCAACATT          Raw reads
.................................................TACGCCCAGATGAACAGAATCA.....          225
...................................................CGCCCAGATGAACAGAATCAAC...          101
.................................................TACGCCCAGATGAACAGAAT.......          73
.................................................TACGCCCAGATGAACAGAATC......          38
...................................................CGCCCAGATGAACAGAATCAA....          31
.................................................TACGCCCAGATGAACAGAATCAA....          15
................................................CTACGCCCAGATGAACAGAAT.......          10
...............................................GCTACGCCCAGATGAACAGAAT.......          9
......ATTCTGTTCATCTGGACGTAG.................................................          3
....TGATTCTGTTCATCTGGACG....................................................          3
.......TTCTGTTCATCTGGACGTAGA................................................          3
..............................................CGCTACGCCCAGATGAACAGAA........          3
.................................................TACGCCCAGATGAACAGAA........          3
..................................................ACGCCCAGATGAACAGAAT.......          3
...TTGATTCTGTTCATCTGGAC.....................................................          2
.......TTCTGTTCATCTGGACGTAG.................................................          2
..................................................ACGCCCAGATGAACAGAATCA.....          2
...................................................CGCCCAGATGAACAGAATCA.....          2
....................................................GCCCAGATGAACAGAATCAAC...          2
........................................................AGATGAACAGAATCAAC...          2
AAGTTGATTCTGTTCATC..........................................................          1
...TTGATTCTGTTCATCT.........................................................          1
..GTTGATTCTGTTCATCTGGA......................................................          1
.....GATTCTGTTCATCTGGA......................................................          1
......ATTCTGTTCATCTGGA......................................................          1
..GTTGATTCTGTTCATCTGGAC.....................................................          1
....TGATTCTGTTCATCTGGAC.....................................................          1
.....GATTCTGTTCATCTGGAC.....................................................          1
...TTGATTCTGTTCATCTGGACG....................................................          1
.....GATTCTGTTCATCTGGACG....................................................          1
........TCTGTTCATCTGGACG....................................................          1
.......TTCTGTTCATCTGGACGTAGAGT..............................................          1
...............................................GCTACGCCCAGATGAACAGAA........          1
..............................................CGCTACGCCCAGATGAACAGAAT.......          1
.....................................................CCCAGATGAACAGAAT.......          1
.......................................................CAGATGAACAGAATCA.....          1
.......................................................CAGATGAACAGAATCAAC...          1
..(((((((((((((((((((.(((((.((((.(.......).)))).))))).)))))))))))))))))))...          (-43.40 MFE)
```

Supplementary Figure S2. Isoform sequence type statistics.

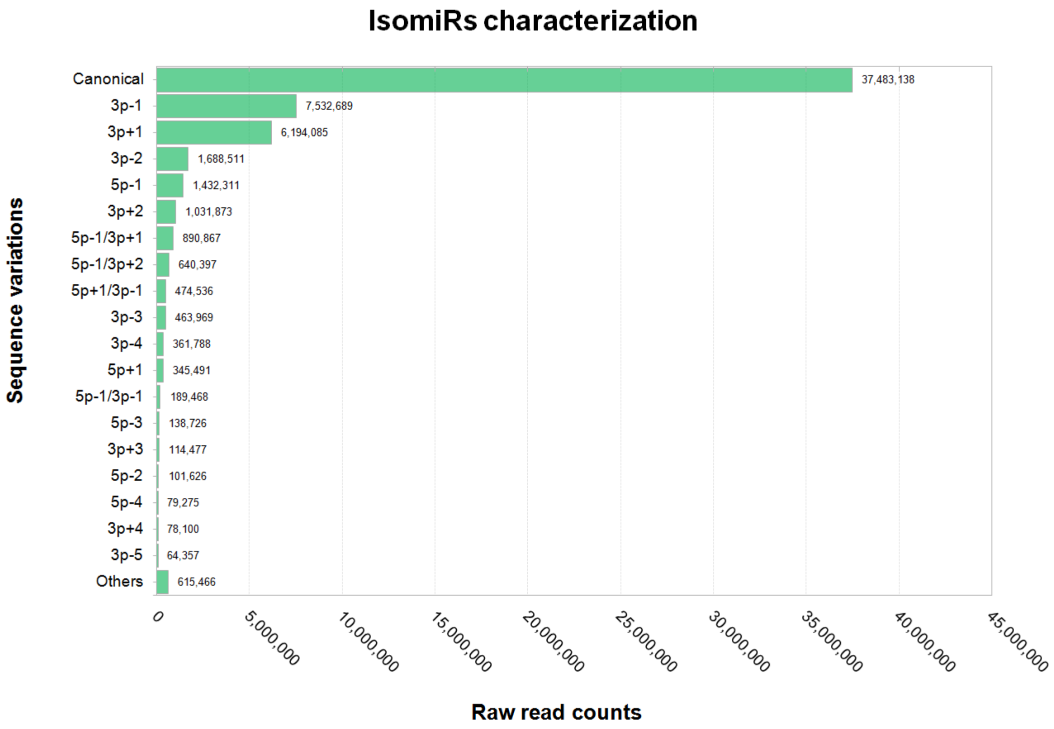

Isoform type distribution shows the raw read counts among variation sequence categories. 5p and 3p represents mature miRNA sequence end and +N or -N the type of variation. +N means increase of N nucleotides and -N reduction of N nucleotides in the mature sequence end. "Others" category include remaining categories and non-classified sequences, however non-template isoform count are not included.

Supplementary Figure S3. An in silico example of how 5' end isomiR affects target regulation processes.

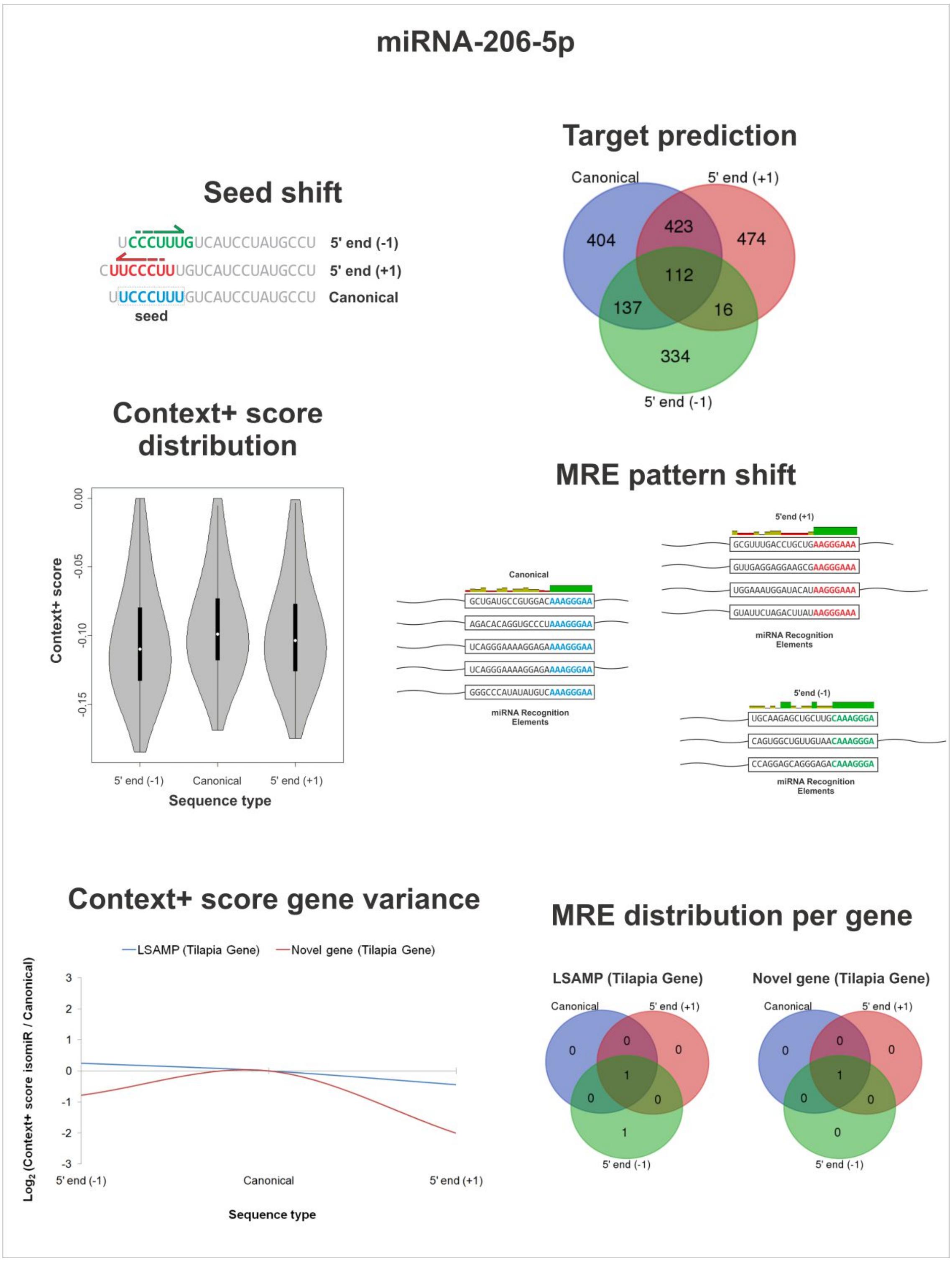

An in silico example of miRNA target prediction using three sequence types of miR-206-5p: canonical, 5' end with one nt reduction, and 5' end with one nt increases. (a) An example of seed shift region from canonical to isomiR sequences. (b) Veen-diagram of target gene prediction indicates that isomiRs can control several targets distinct from canonical, although a few number of 112 target gene (5.9%) can be controlled by all three isoform types. (c) Distribution of Context+Score[21sr, 22sr] indicates isomiRs affecting binding characteristics between miRNAs and miRNA Recognation Elements (MREs). (d) An example of 3' UTR MRE pattern shifts of predicted target genes among isoform types. In the most cases, alteration on seed region pattern is followed by MREs pattern alteration. (e) Context+Score variation of two genes (LSAMP and Novel) possible controlled by the three isoforms indicates that variant 5' end isoforms can result in target affinity with the same or additional MREs. (f) MRE counts of LSAMP and Novel gene indicates affinity changes either additional isoform MREs (LSAMP gene case) or characteristics of same MRE (novel gene case). We used TargetScan v6.0 as prediction tool [22sr].

Supplementary Figure S4. Z-score distribution of entropy values from miRNA expression among samples.

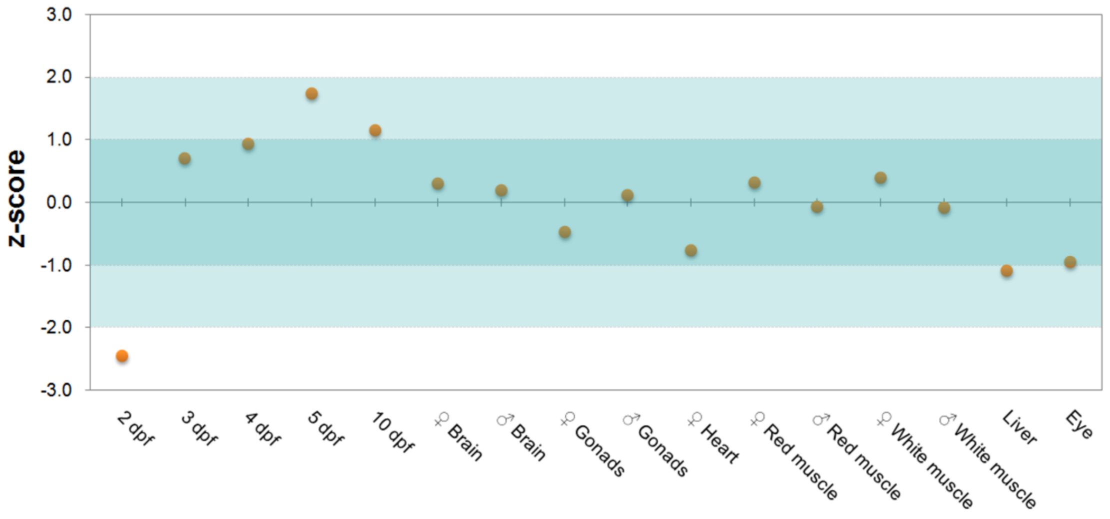

The z-score distribution values induce confidence intervals (standard deviations in x-axes) to entropy values among samples. 2 days post-fertilization (dpf), liver and eye samples show low entropy levels indicating low diversity of miRNAs. In the other hand, 5 and 10 dpf displayed high entropy values indicating high diversity of miRNAs expressed in this developmental stage period.

Supplementary Figure S5. Compilation of phylogenetic trees reconciled with species and gene trees of the novel miRNAs for ortholog and paralog gene annotation

oni-mir-n003-1/2/3

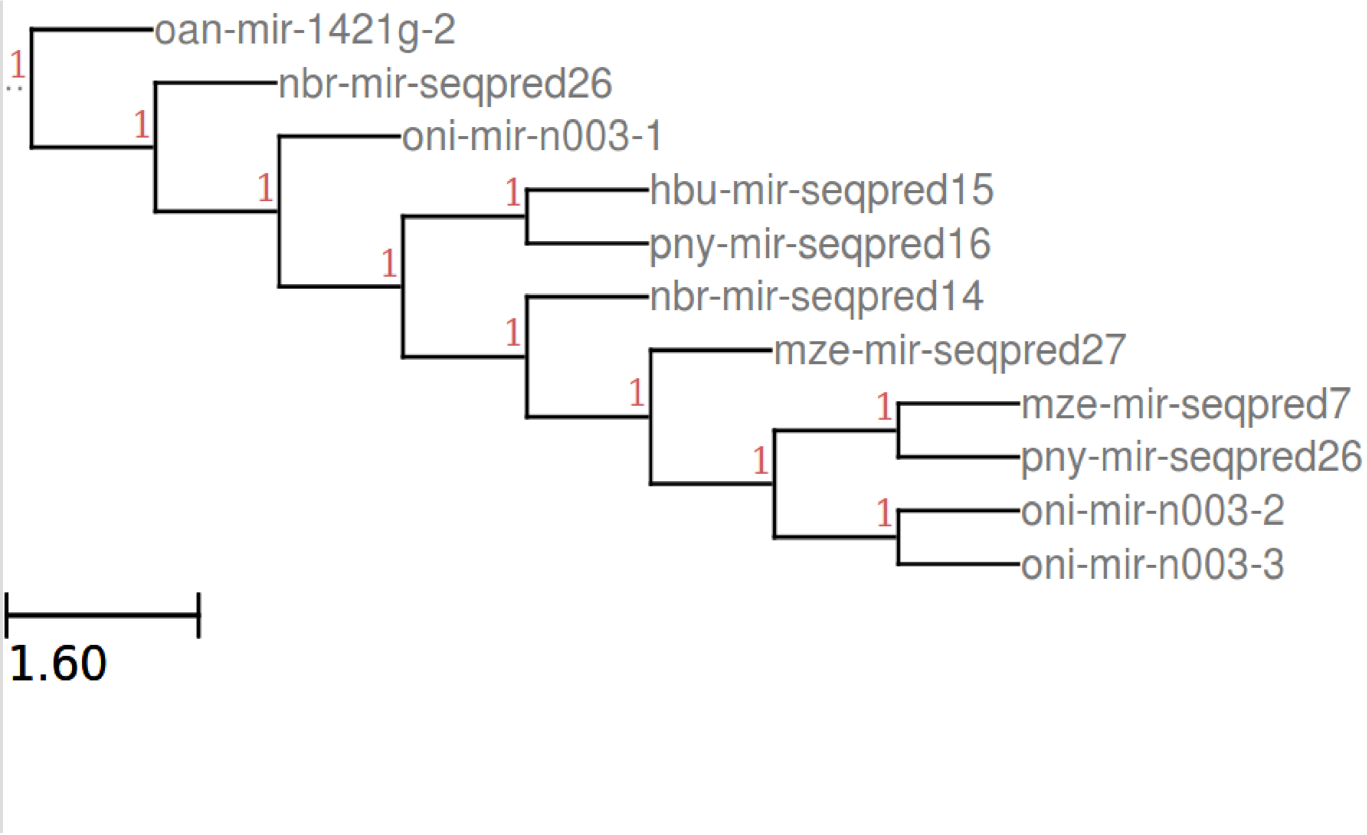

oni-mir-n012

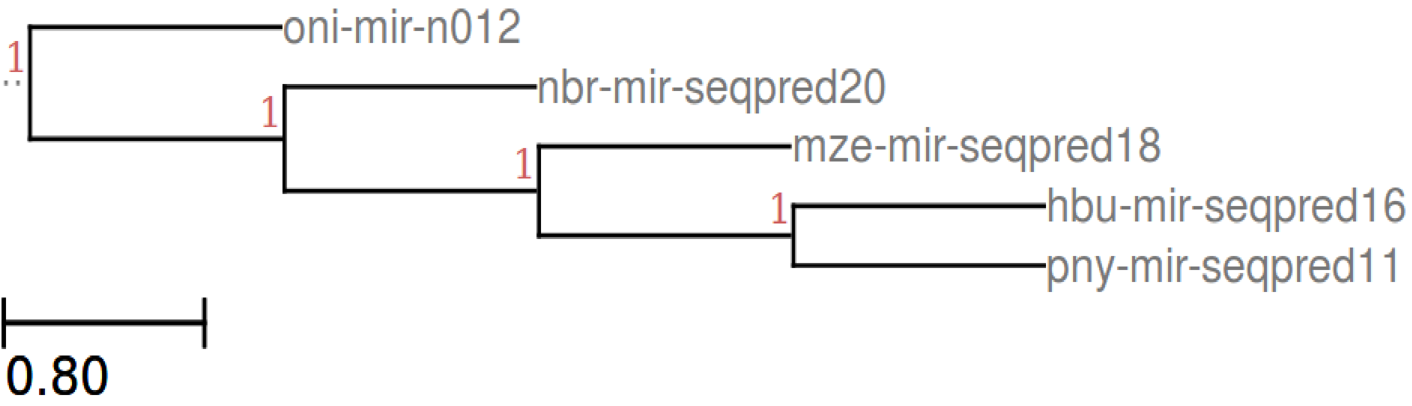

oni-mir-n012
nbr-mir-seqpred20
mze-mir-seqpred18
hbu-mir-seqpred16
pny-mir-seqpred11
1
1
1
1
0.80

oni-mir-n015

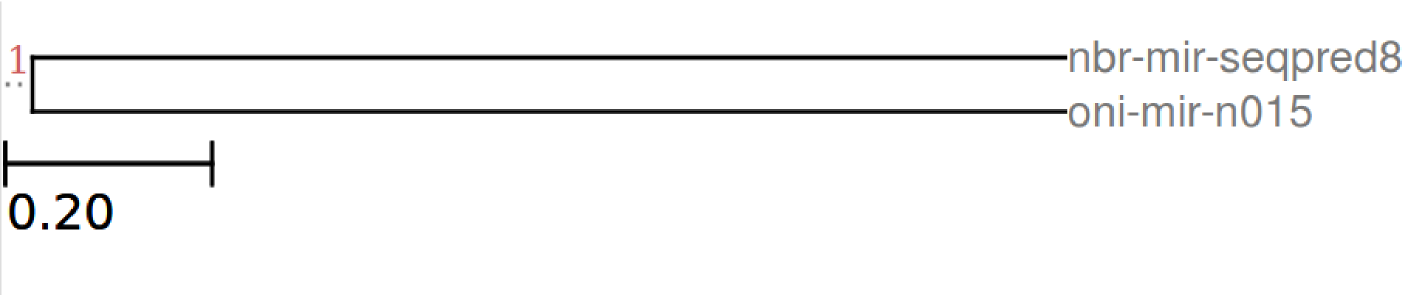

oni-mir-n024-1/2

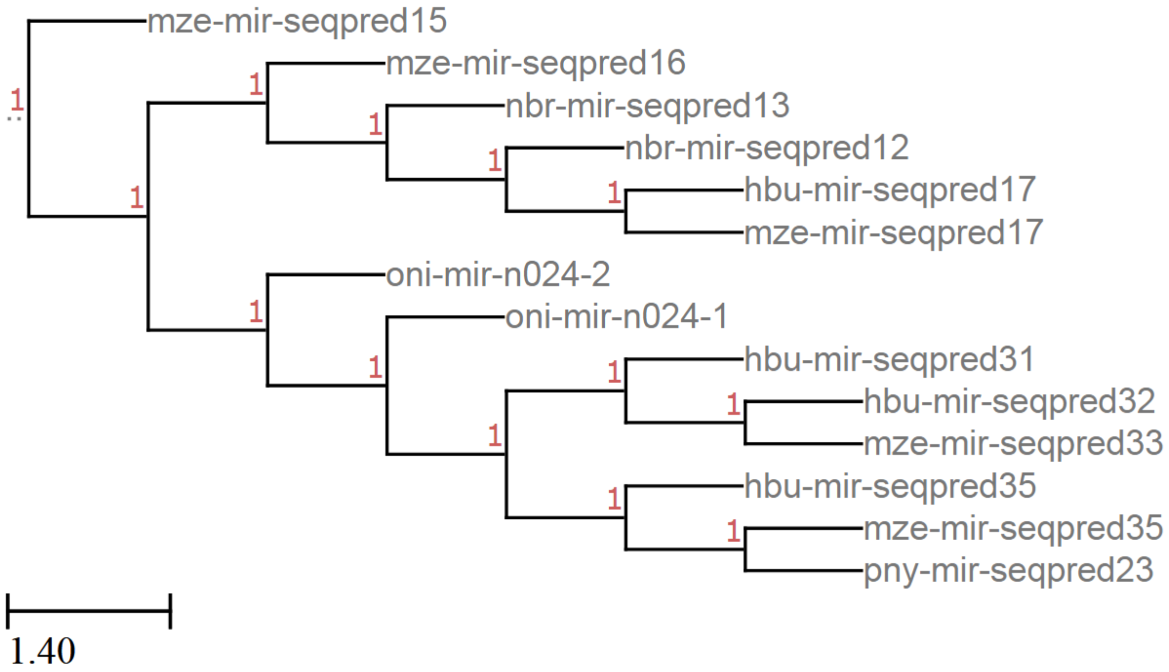

oni-mir-n057

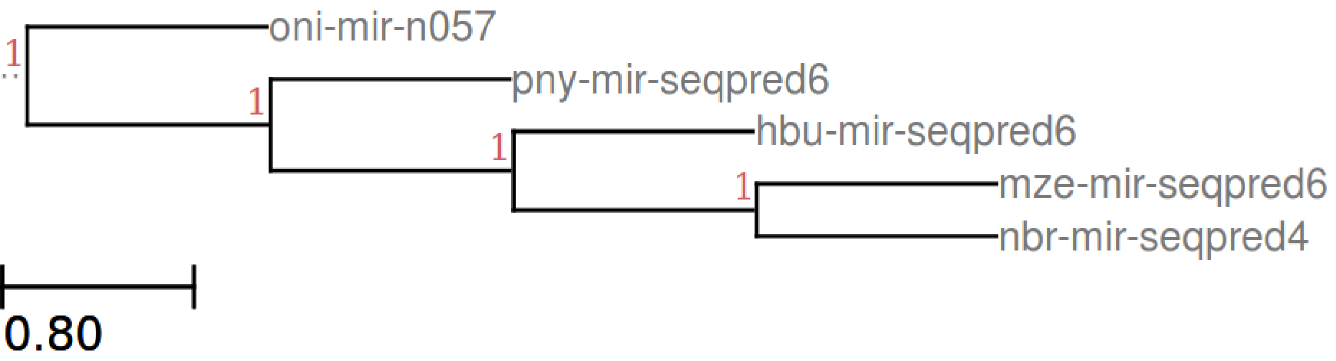
oni-mir-n057
pny-mir-seqpred6
hbu-mir-seqpred6
mze-mir-seqpred6
nbr-mir-seqpred4
1
1
1
1
0.80

oni-mir-n106

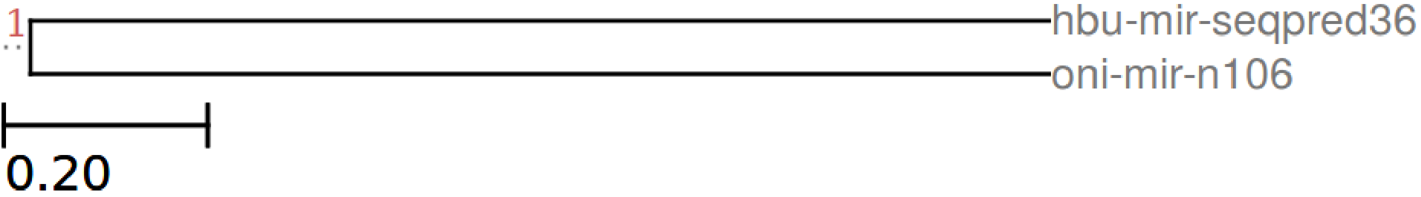

oni-mir-n260-1/2 – oni-mir-n885-1/2

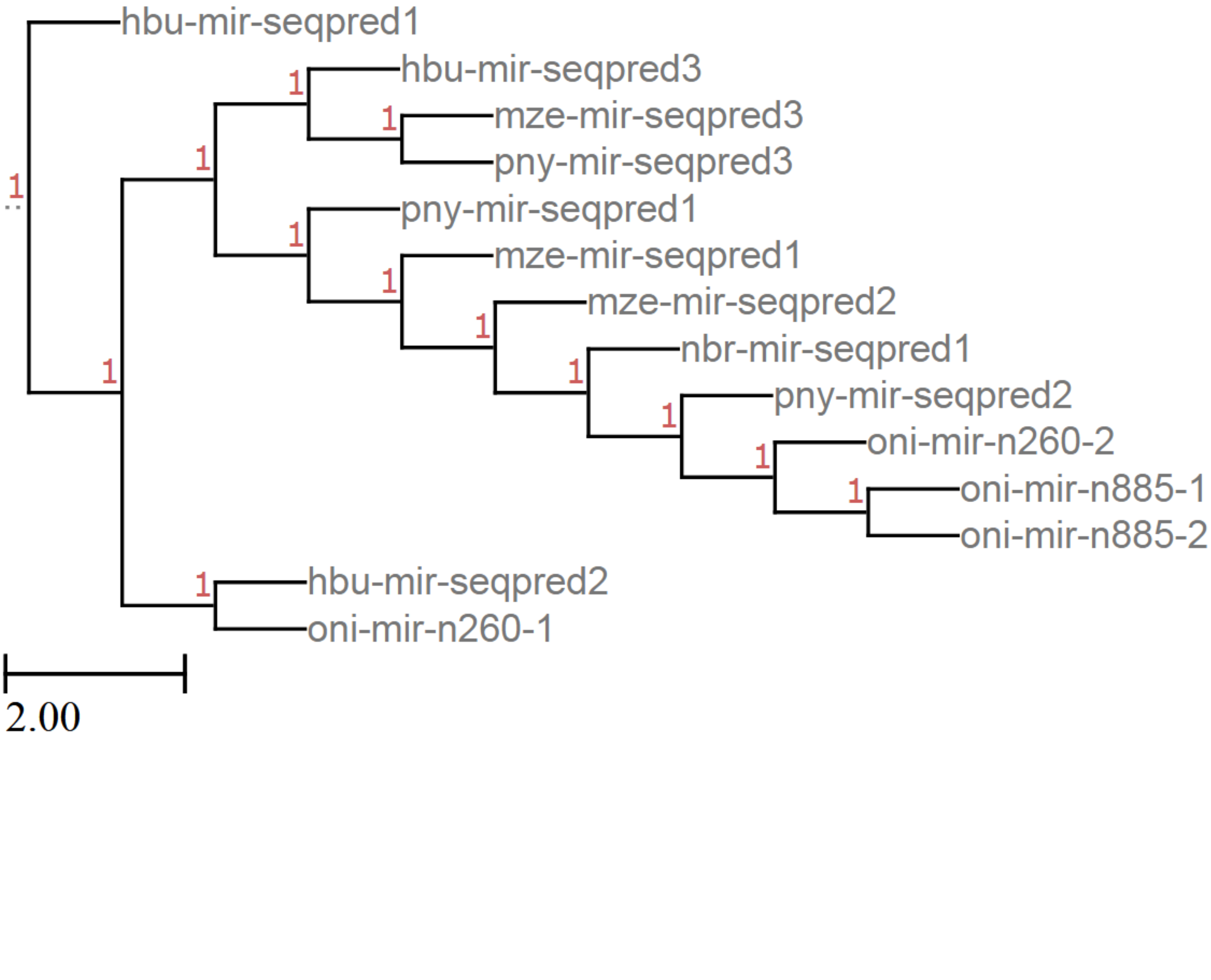

oni-mir-n287-1/2

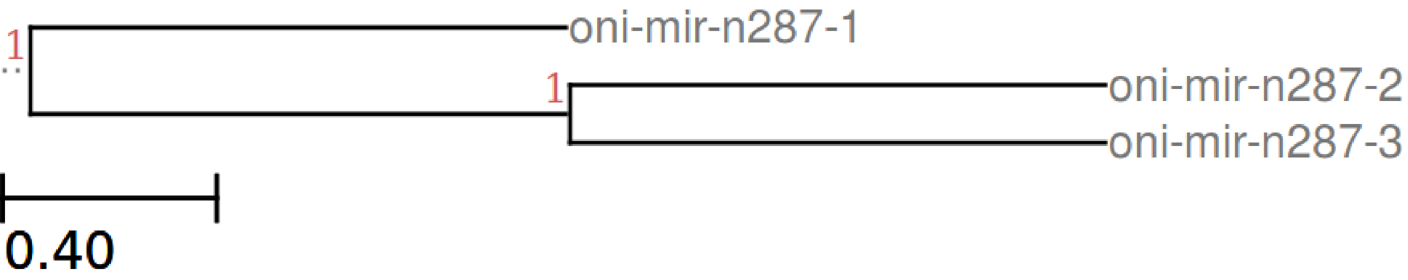

oni-mir-n329 – oni-mir-n487 – oni-mir-n965

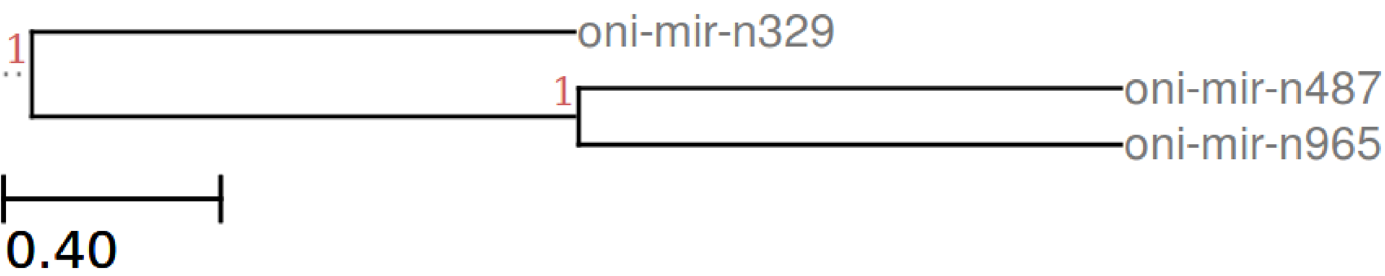

oni-mir-n363-1/2

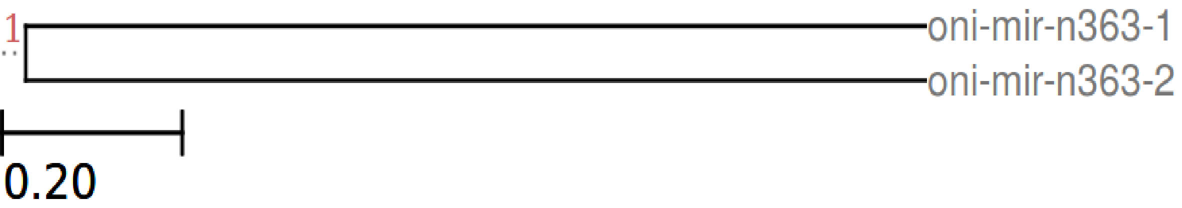

oni-mir-n437

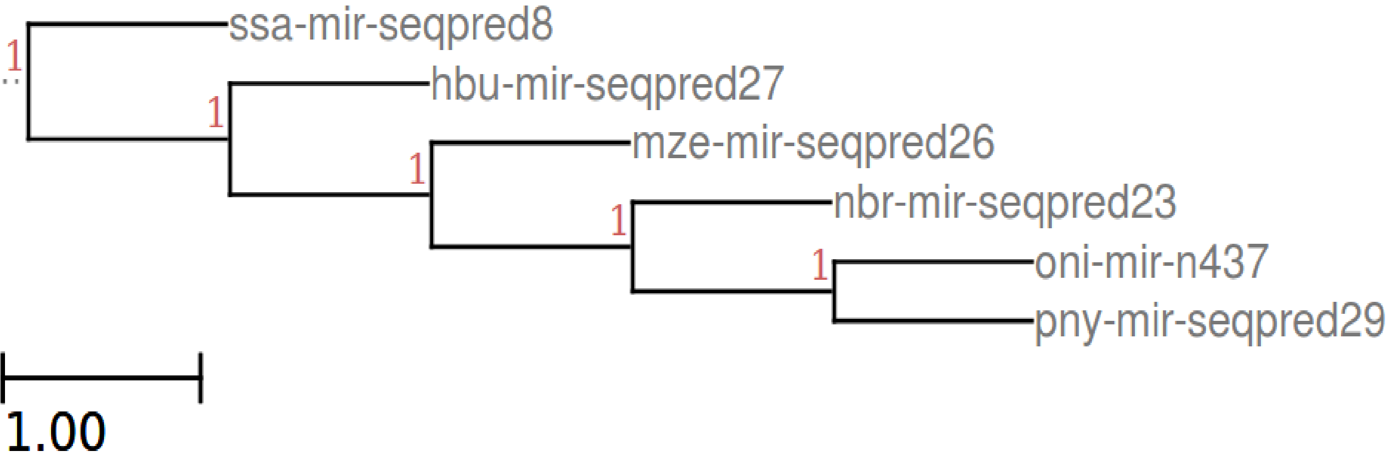

ssa-mir-seqpred8
hbu-mir-seqpred27
mze-mir-seqpred26
nbr-mir-seqpred23
oni-mir-n437
pny-mir-seqpred29
1
1
1
1
1
1.00

oni-mir-n456

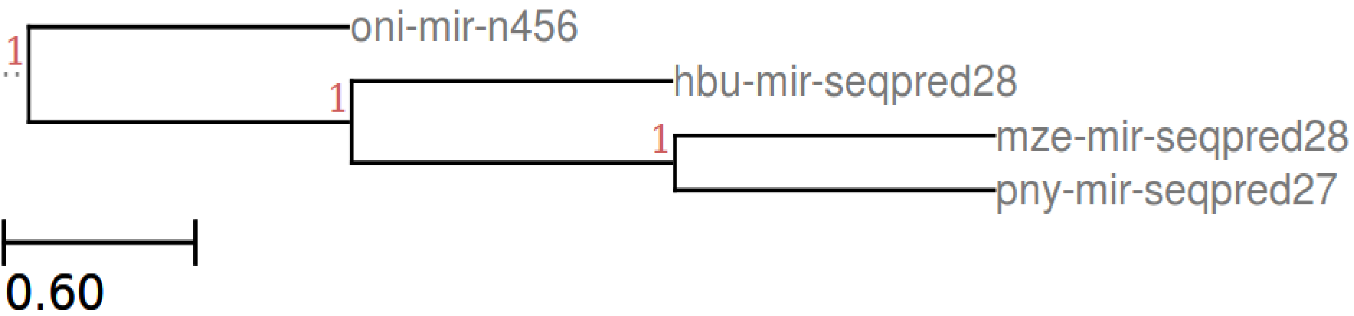
oni-mir-n456
hbu-mir-seqpred28
mze-mir-seqpred28
pny-mir-seqpred27
1
1
1
0.60

oni-mir-n470a/b

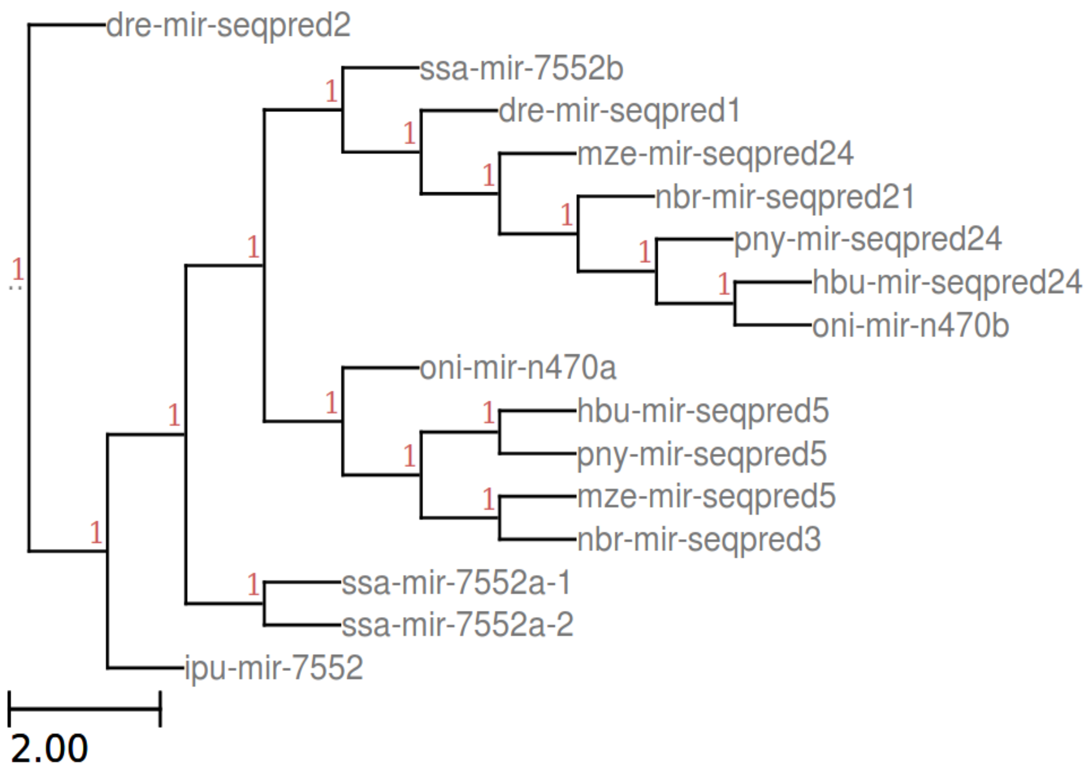

oni-mir-n483

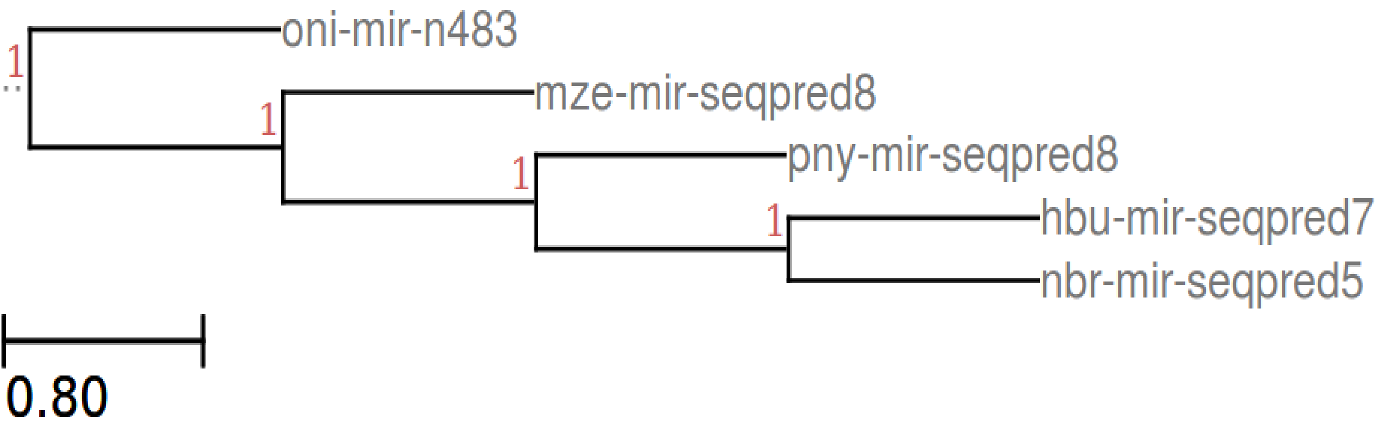

oni-mir-n483
mze-mir-seqpred8
pny-mir-seqpred8
hbu-mir-seqpred7
nbr-mir-seqpred5
1
1
1
1
0.80

oni-mir-n500

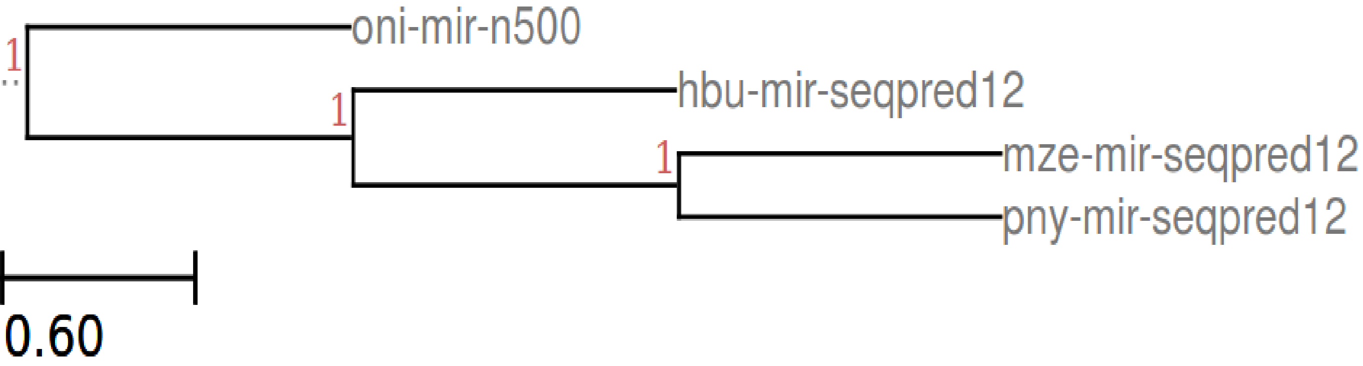

oni-mir-n500
hbu-mir-seqpred12
mze-mir-seqpred12
pny-mir-seqpred12
1
1
1
0.60

oni-mir-n518

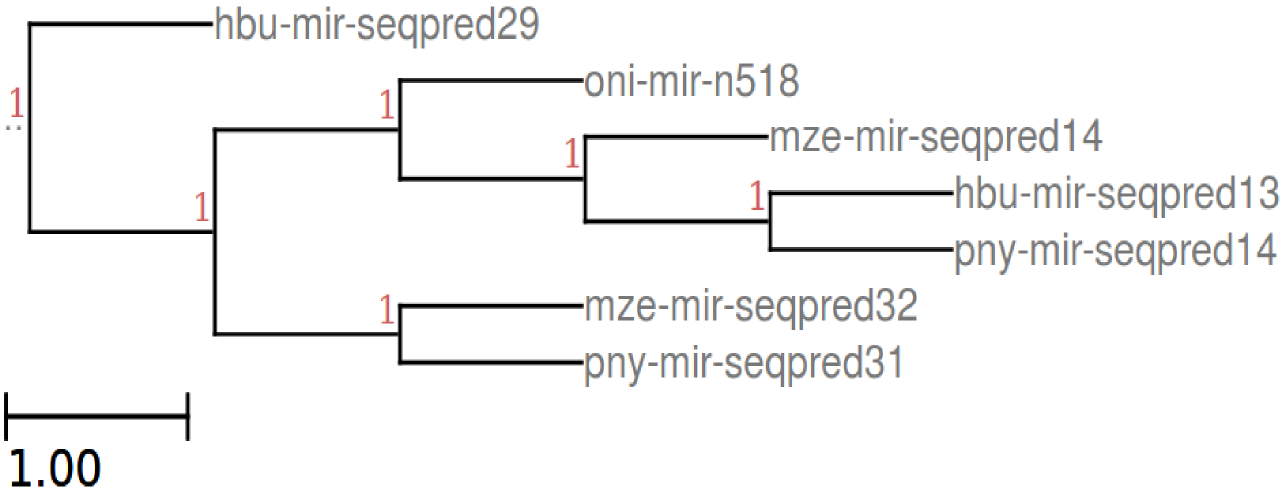

hbu-mir-seqpred29
oni-mir-n518
mze-mir-seqpred14
hbu-mir-seqpred13
pny-mir-seqpred14
mze-mir-seqpred32
pny-mir-seqpred31
1
1
1
1
1
1.00

oni-mir-n530

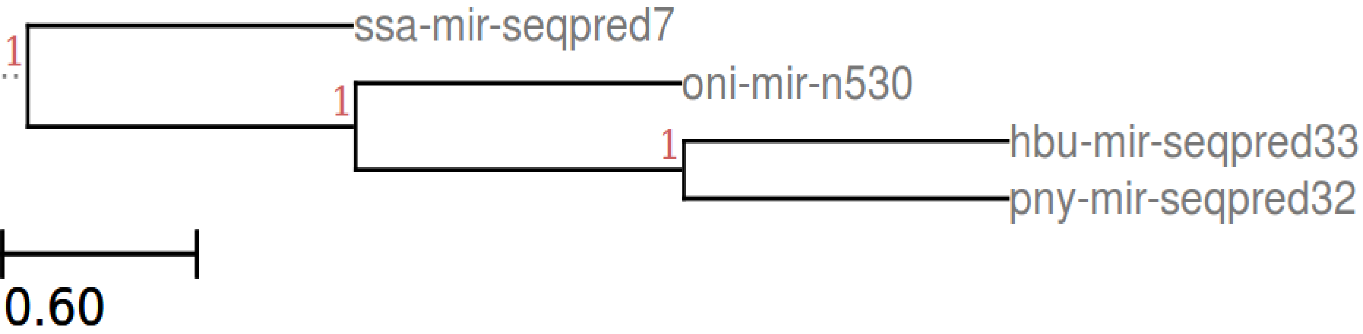

ssa-mir-seqpred7
oni-mir-n530
hbu-mir-seqpred33
pny-mir-seqpred32
1
1
1
0.60

oni-mir-547

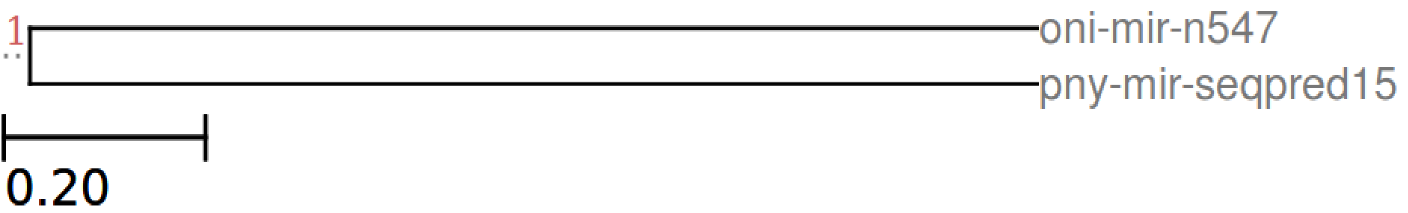

oni-mir-n579

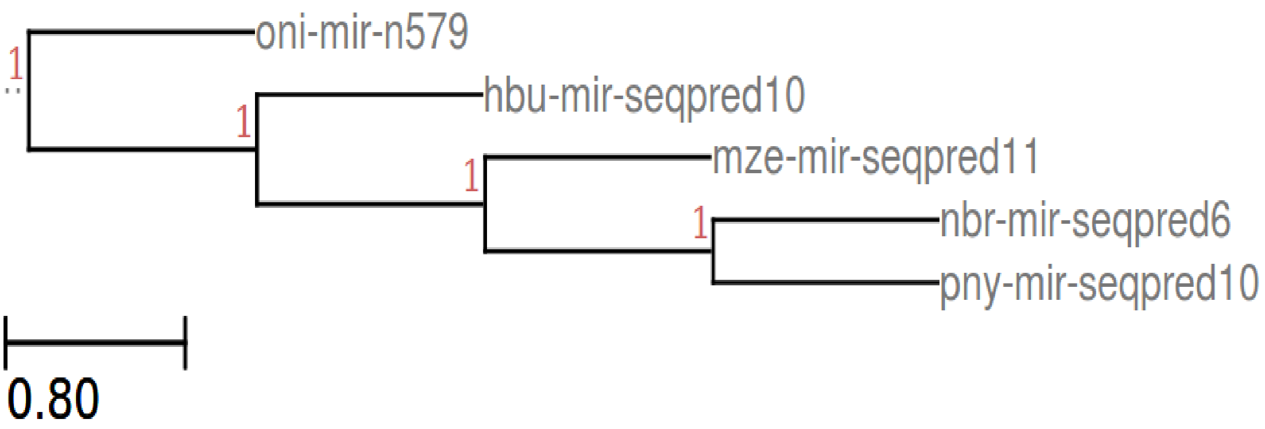

oni-mir-n579
hbu-mir-seqpred10
mze-mir-seqpred11
nbr-mir-seqpred6
pny-mir-seqpred10
1
1
1
1
0.80

oni-mir-n692

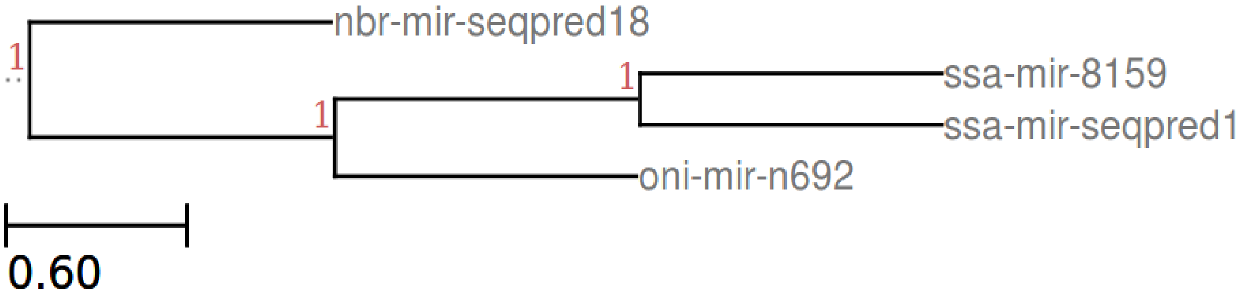

nbr-mir-seqpred18
ssa-mir-8159
ssa-mir-seqpred1
oni-mir-n692
1
1
1
0.60

oni-mir-n739

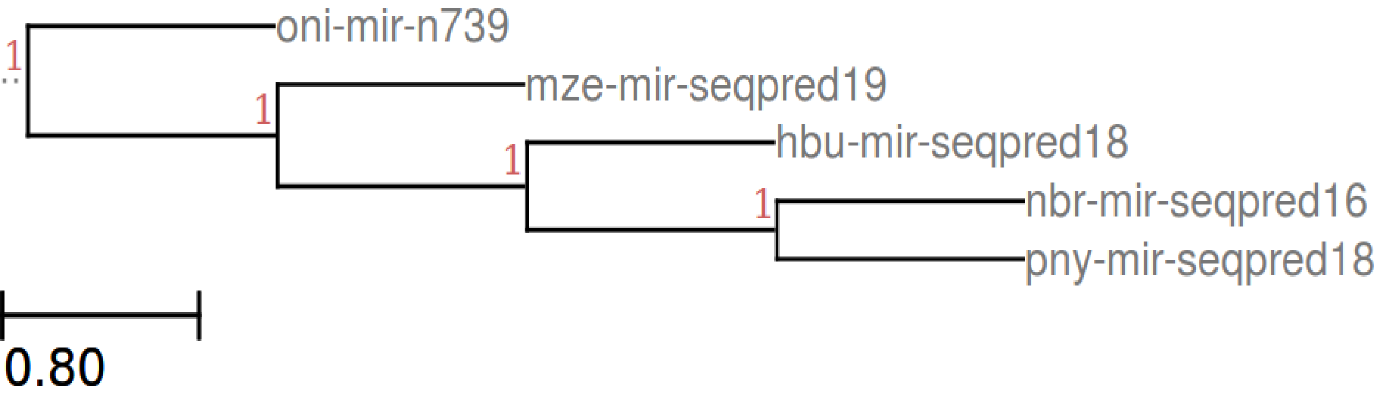

oni-mir-n739
mze-mir-seqpred19
hbu-mir-seqpred18
nbr-mir-seqpred16
pny-mir-seqpred18
1
1
1
1
0.80

oni-mir-n741

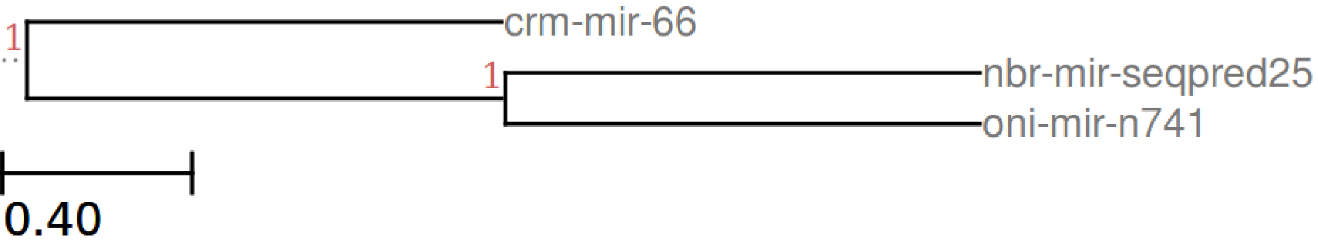

oni-mir-n771

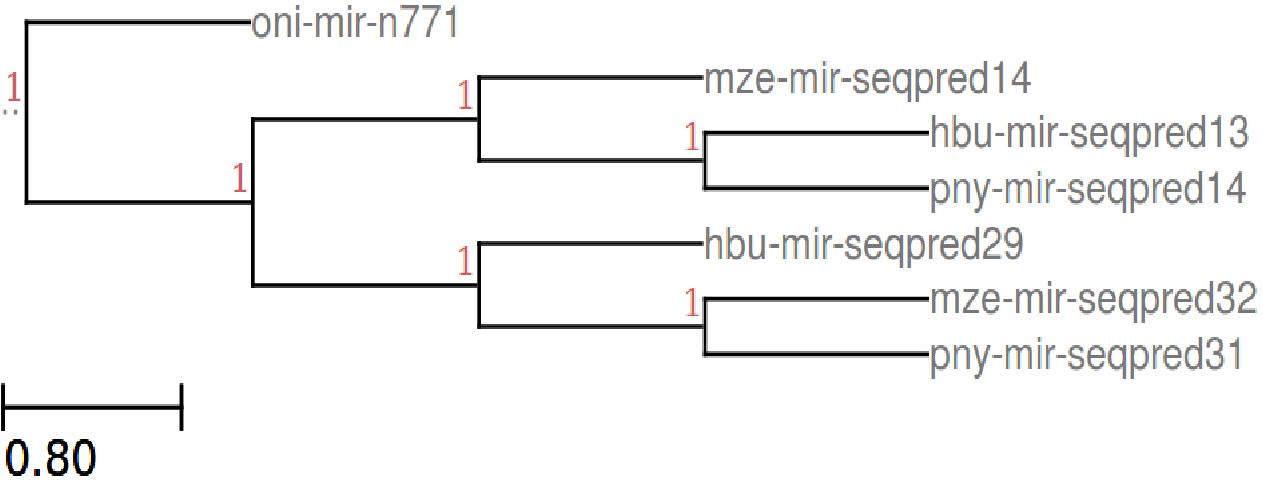

oni-mir-n771
mze-mir-seqpred14
hbu-mir-seqpred13
pny-mir-seqpred14
hbu-mir-seqpred29
mze-mir-seqpred32
pny-mir-seqpred31
1
1
1
1
1
1
0.80

oni-mir-n780

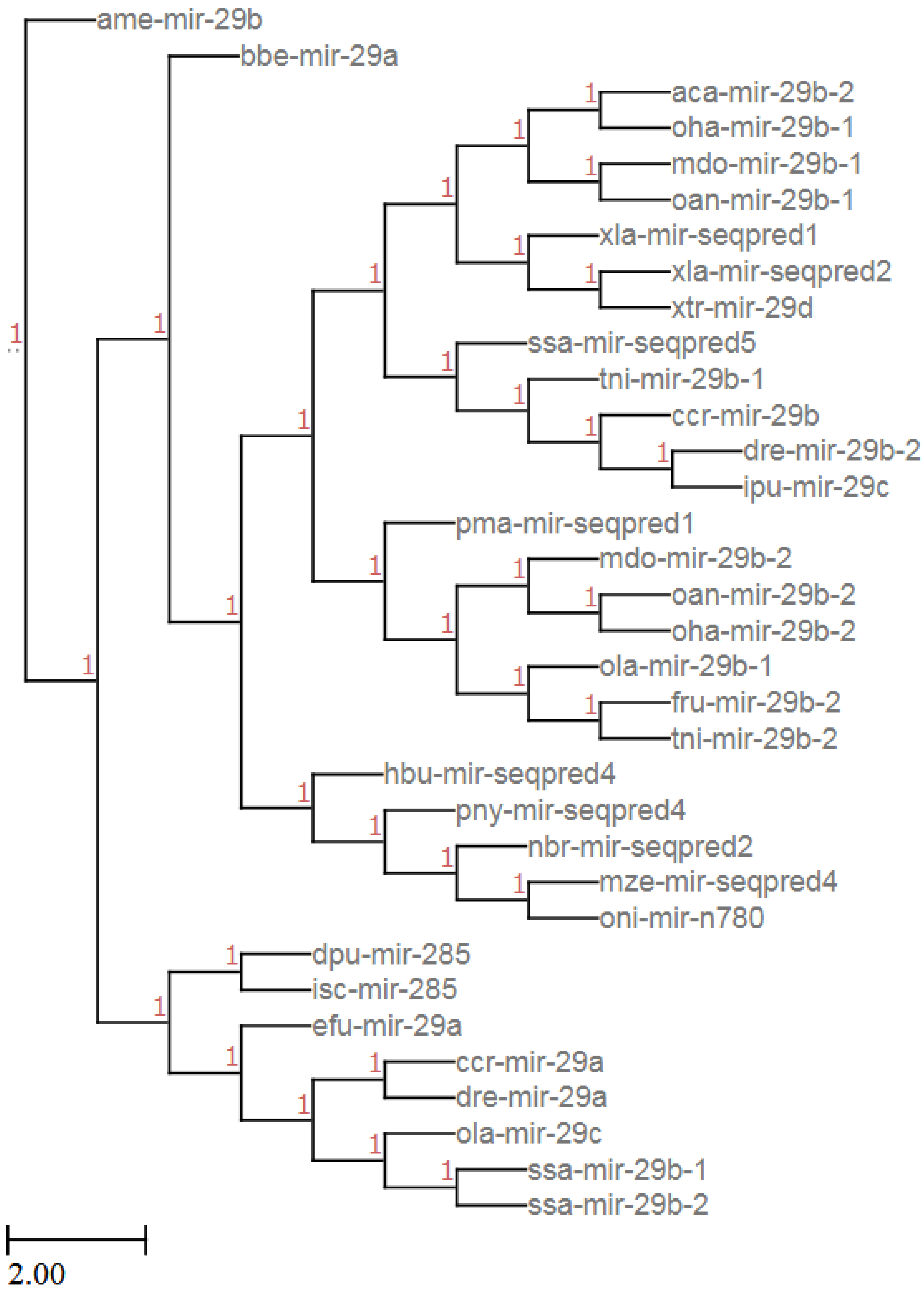

ame-mir-29b
bbe-mir-29a
aca-mir-29b-2
oha-mir-29b-1
mdo-mir-29b-1
oan-mir-29b-1
xla-mir-seqpred1
xla-mir-seqpred2
xtr-mir-29d
ssa-mir-seqpred5
tni-mir-29b-1
ccr-mir-29b
dre-mir-29b-2
ipu-mir-29c
pma-mir-seqpred1
mdo-mir-29b-2
oan-mir-29b-2
oha-mir-29b-2
ola-mir-29b-1
fru-mir-29b-2
tni-mir-29b-2
hbu-mir-seqpred4
pny-mir-seqpred4
nbr-mir-seqpred2
mze-mir-seqpred4
oni-mir-n780
dpu-mir-285
isc-mir-285
efu-mir-29a
ccr-mir-29a
dre-mir-29a
ola-mir-29c
ssa-mir-29b-1
ssa-mir-29b-2
2.00

oni-mir-n784

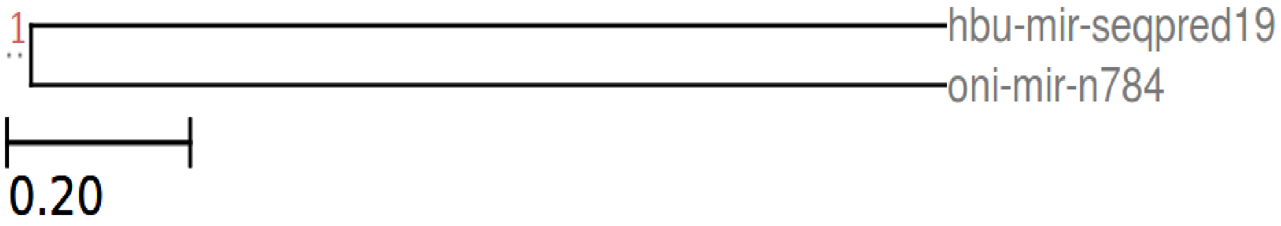

oni-mir-n813-1/2

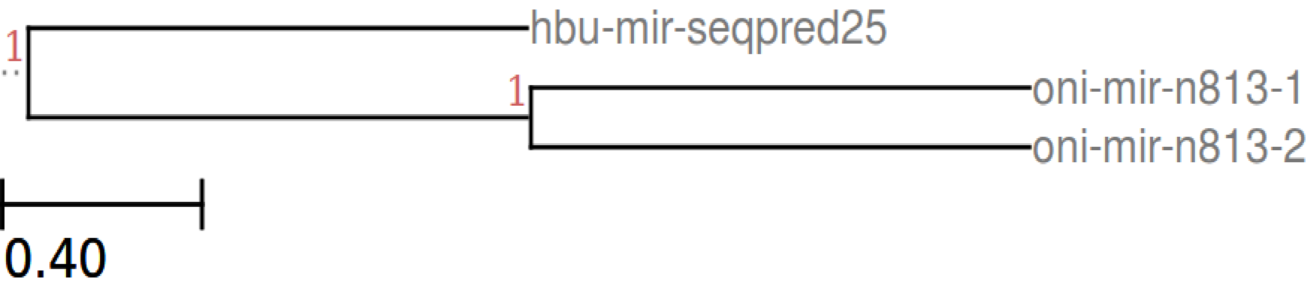

oni-mir-n246 – oni-mir-n836

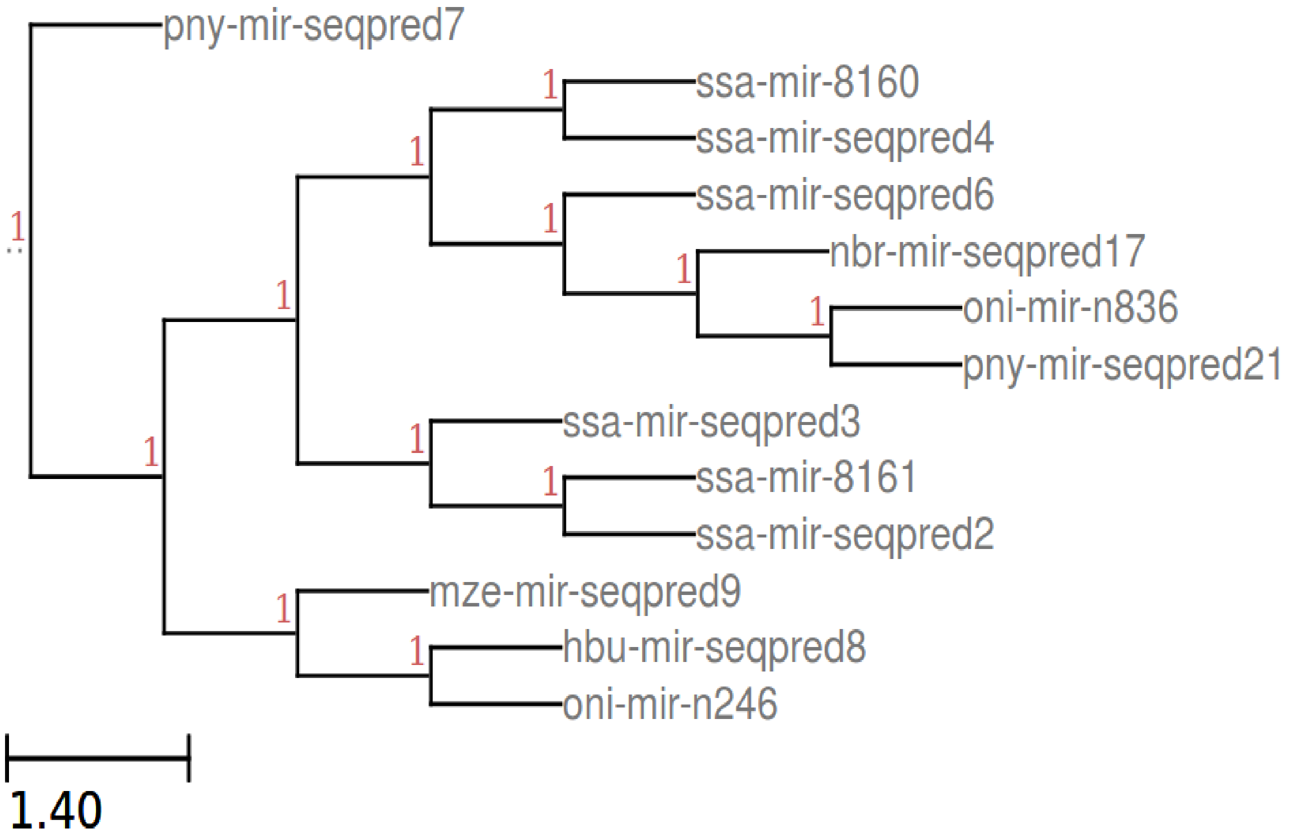

oni-mir-n852

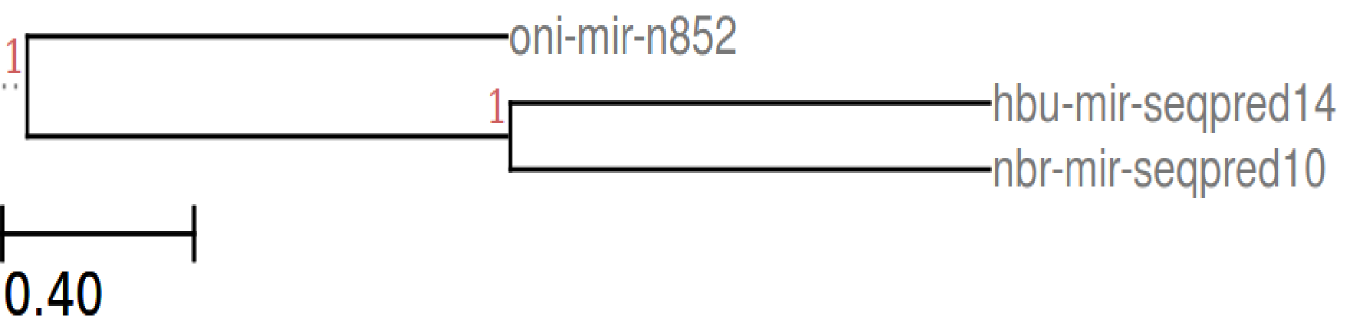

oni-mir-n875

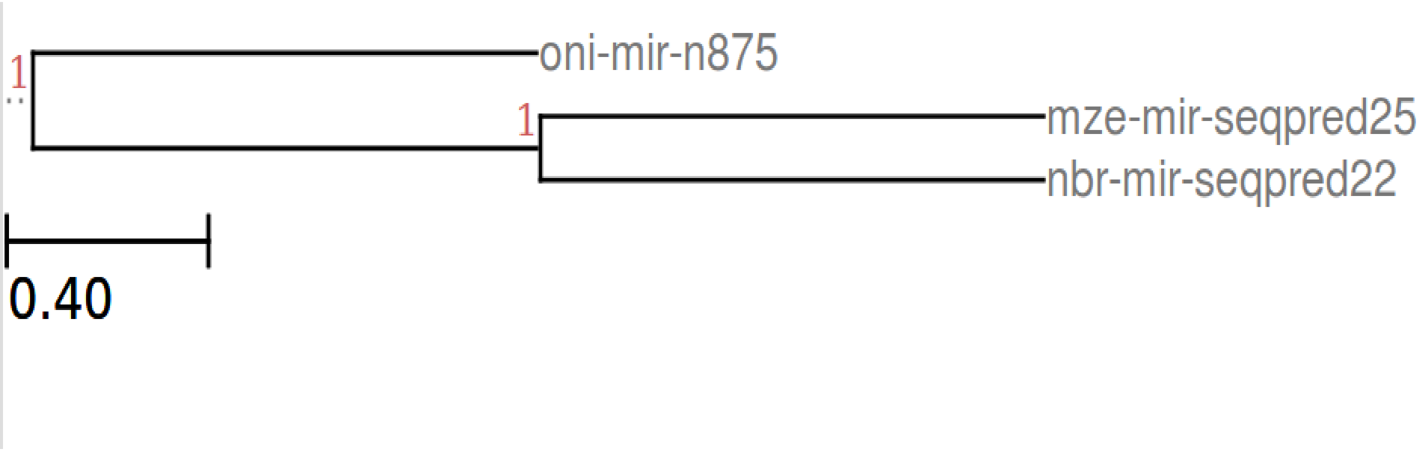

oni-mir-n878

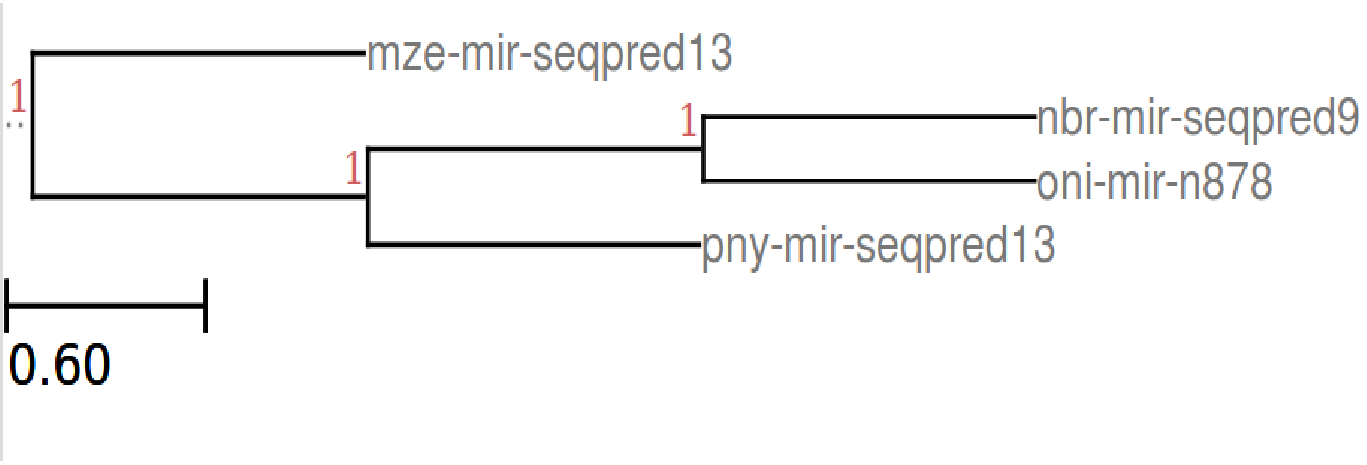

mze-mir-seqpred13
nbr-mir-seqpred9
oni-mir-n878
pny-mir-seqpred13
1
1
1
0.60

oni-mir-n886

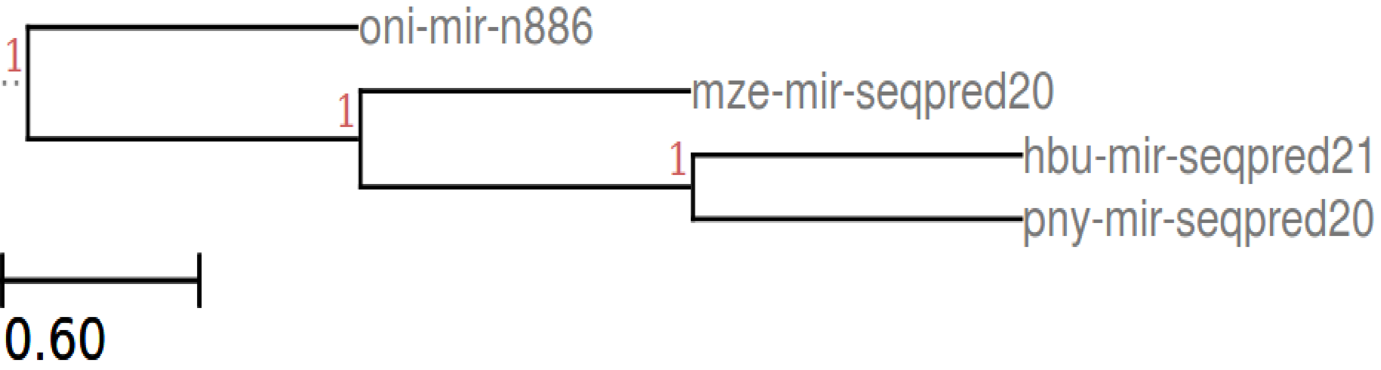

oni-mir-n886
mze-mir-seqpred20
hbu-mir-seqpred21
pny-mir-seqpred20
1
1
1
0.60

oni-mir-n893

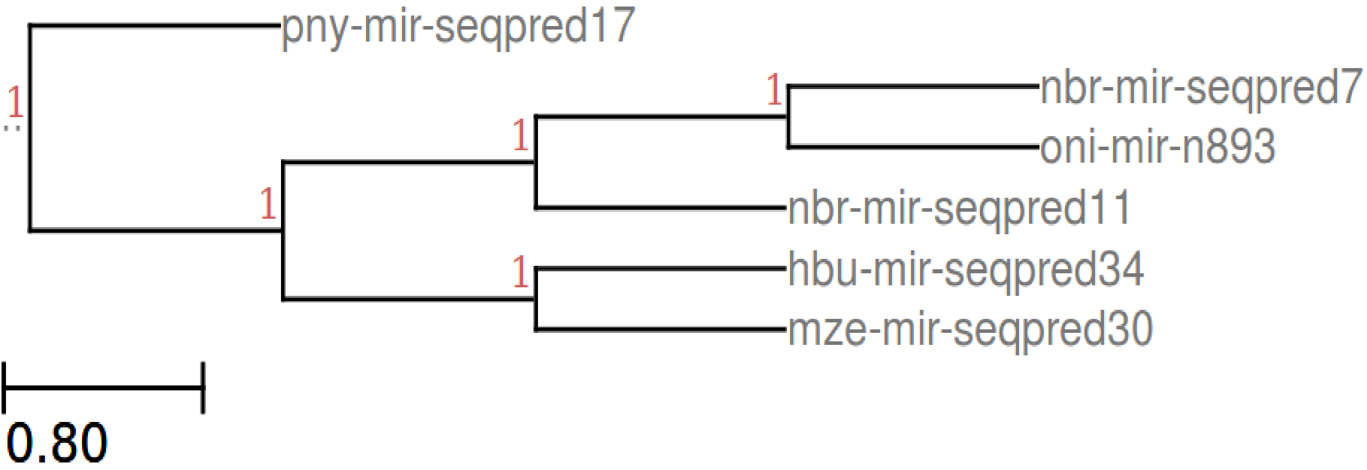

pny-mir-seqpred17
nbr-mir-seqpred7
oni-mir-n893
nbr-mir-seqpred11
hbu-mir-seqpred34
mze-mir-seqpred30
1
1
1
1
1
0.80

oni-mir-n896

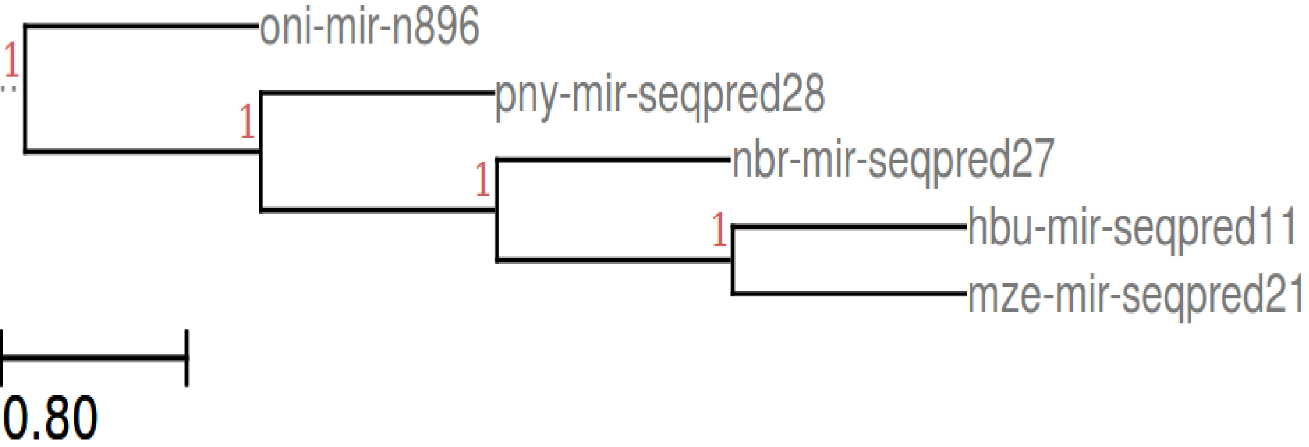
oni-mir-n896
pny-mir-seqpred28
nbr-mir-seqpred27
hbu-mir-seqpred11
mze-mir-seqpred21
1
1
1
1
0.80

oni-mir-n737 – oni-mir-n941

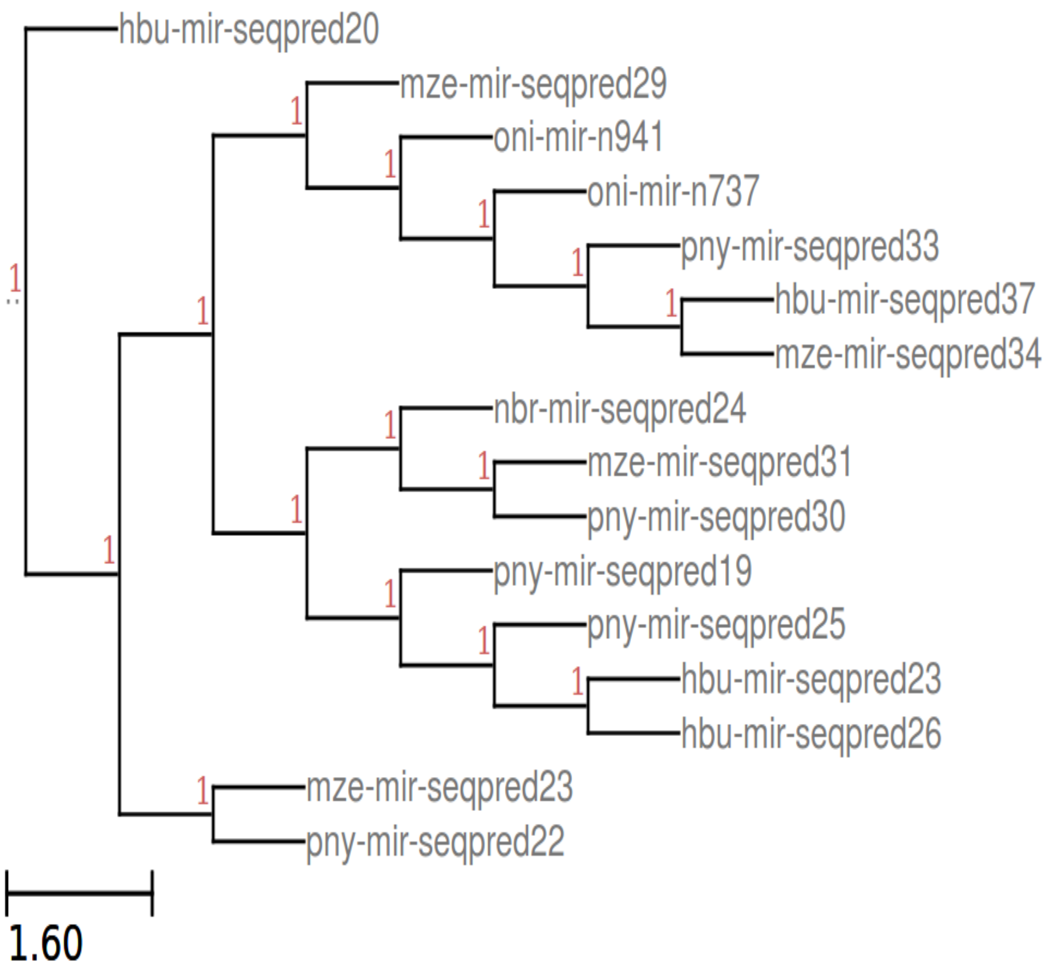

oni-mir-n943

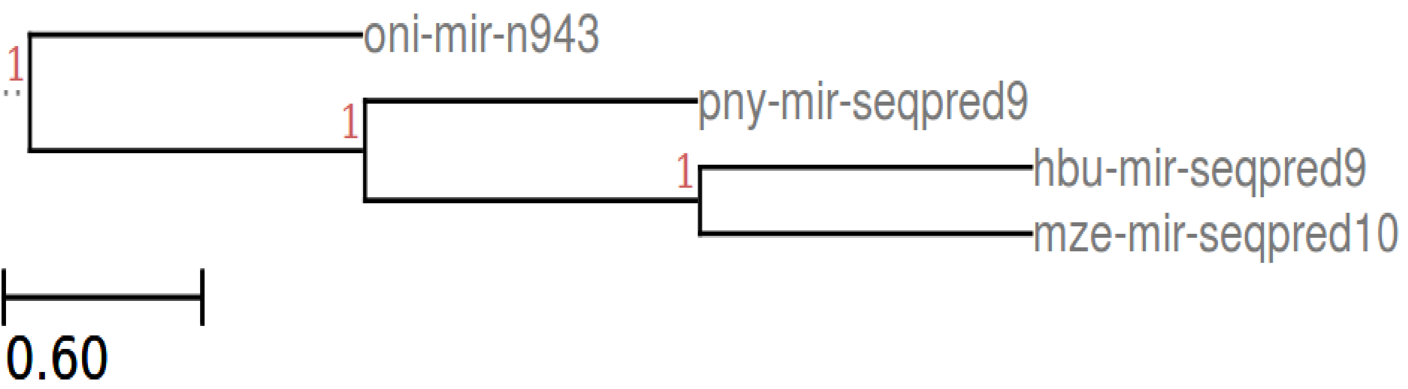
oni-mir-n943
pny-mir-seqpred9
hbu-mir-seqpred9
mze-mir-seqpred10
1
1
1
0.60

oni-mir-n948

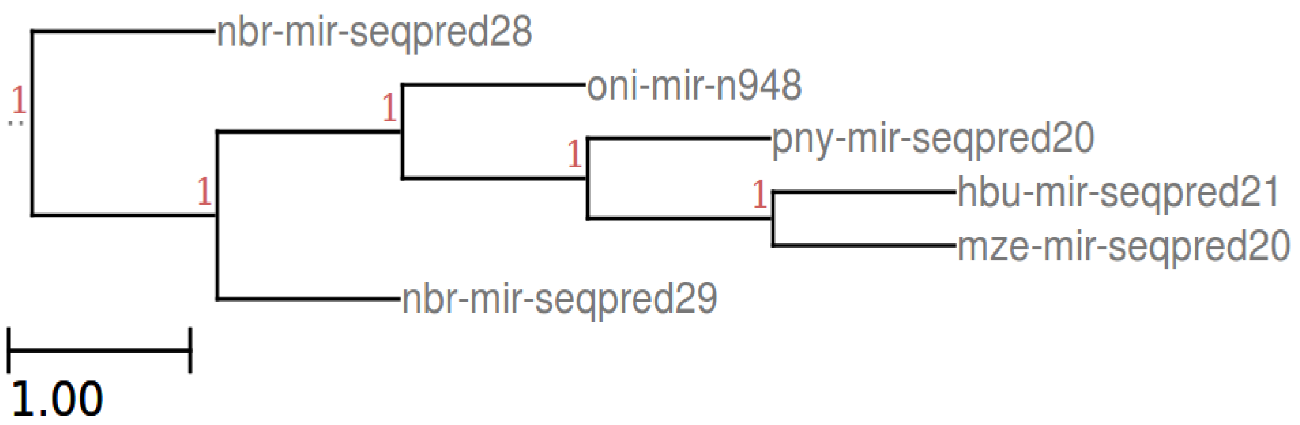

nbr-mir-seqpred28
oni-mir-n948
pny-mir-seqpred20
hbu-mir-seqpred21
mze-mir-seqpred20
nbr-mir-seqpred29
1
1
1
1
1
1.00

List of reconciled trees using RAxML and TreeBest to reconstructing the historical evolutive events among species of novel miRNAs.

Supplementary Figure S6. miRNA categories evolutionary rate distribution.

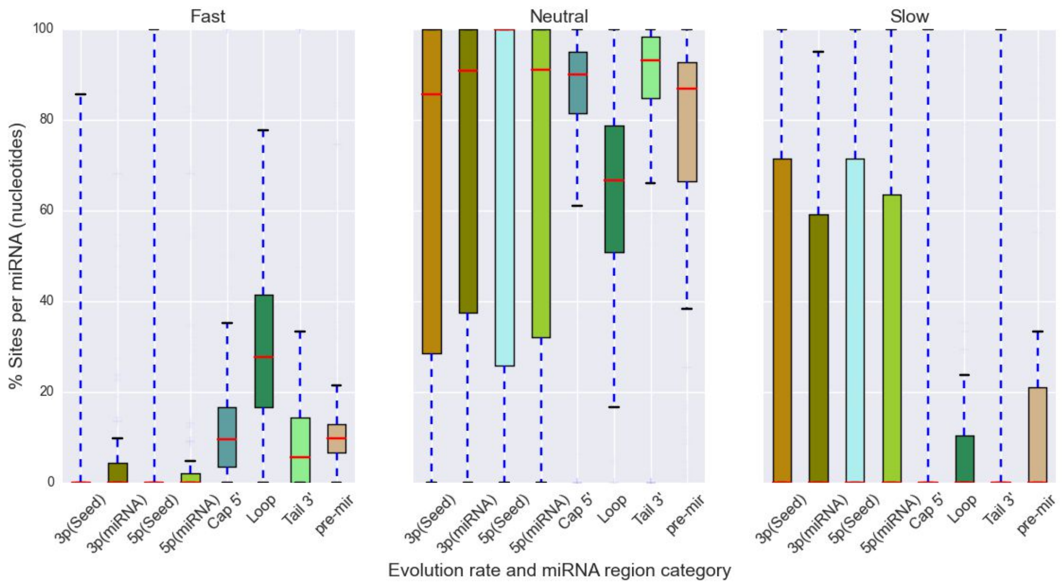

Distribution of evolution rate categories. Nucleotides were classified in slow, neutral and fast evolving categories based on phyloP scores (-log10(p-value)) computed by PhyloP algorithm from PHAST package[19sr] and count per miRNA region (see methods for more details). The majority regions were neutral or were suffering slow evolution (mature, seed, cap and tail pre-miRNA) except the loop region, the only miRNA region with a high density of fast evolving nucleotides.

Supplementary Figure S7. List of miRNAs containing fast evolving sites.

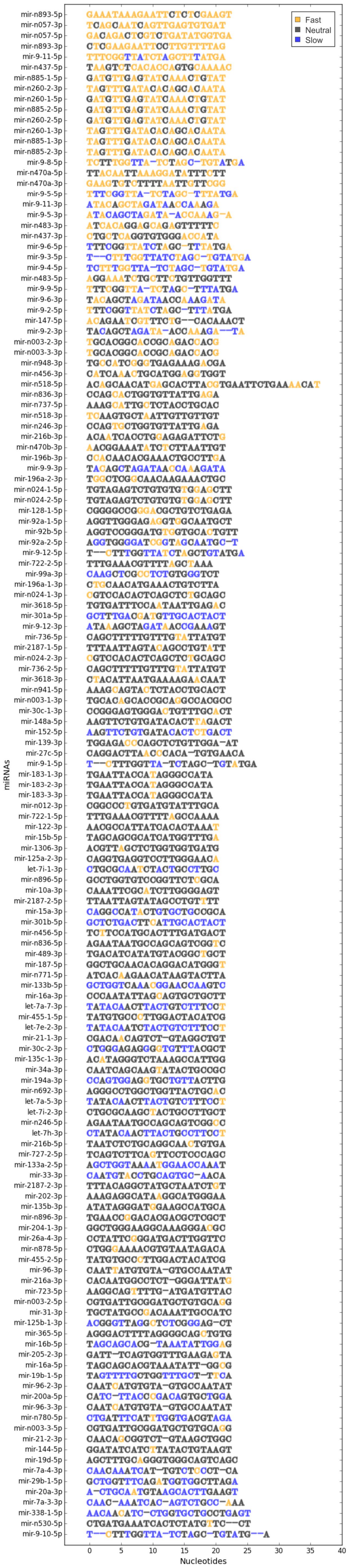

List of mature miRNAs containing fast evolving sites. Nucleotides were classified in slow (blue), neutral (black) and fast evolving (orange) based on phyloP scores computed by PhyloP algorithm from PHAST package (see methods for more details). Data are sorted from higher to lower density of fast evolving sites.

Supplementary Figure S8. List of miRNAs containing fast evolving sites and arm-switching event.

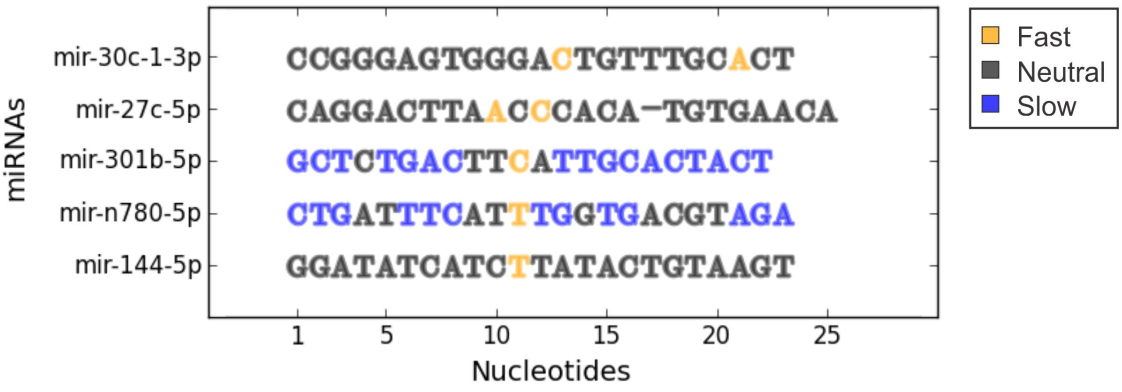

List of mature miRNAs containing fast evolving sites and detected in arm-switching event. Nucleotides were classified in slow (blue), neutral (black) and fast evolving (orange) based on phyloP scores computed by PhyloP algorithm from PHAST package (see methods for more details). Data are sorted from higher to lower density of fast evolving sites.

SUPPLEMENTARY FIGURES AND TABLES References

1sr. Kozomara, A. & Griffiths-Jones, S. miRBase: annotating high confidence microRNAs using deep sequencing data. *Nucleic acids research***42**, D68–D73 (2014).

2sr. Friedlander, M. R., Mackowiak, S. D., Li, N., Chen, W. & Rajewsky, N. miRDeep2 accurately identifies known and hundreds of novel microRNA genes in seven animal clades. *Nucleic acids research***40**, 37–52 (2012).

3sr. Stocks, M. B. et al. The UEA sRNA workbench: a suite of tools for analysing and visualizing next generation sequencing microRNA and small RNA datasets. *Bioinformatics***28**, 2059–2061 (2012).

4sr. Bonnet, E., Wuyts, J., Rouzé, P. & Van de Peer, Y. Evidence that microRNA precursors, unlike other non-coding RNAs, have lower folding free energies than random sequences. *Bioinformatics***20**, 2911–2917 (2004).

5sr. Tam, S., Tsao, M.-S. & McPherson, J. D. Optimization of miRNA-seq data preprocessing. *Briefings in bioinformatics***16**, 950–963 (2015).

6sr. Thatcher, E. J., Bond, J., Paydar, I. & Patton, J. G. Genomic organization of zebrafish microRNAs. *BMC genomics***9**, 1 (2008).

7sr. Juanchich, A. et al. Characterization of an extensive rainbow trout miRNA transcriptome by next generation sequencing. *BMC genomics***17**, 1 (2016).

8sr. Andreassen, R., Worren, M. M. & Høyheim, B. Discovery and characterization of miRNA genes in Atlantic salmon (*Salmo salar*) by use of a deep sequencing approach. *BMC genomics***14**, 1 (2013).

9sr. Bekaert, M. et al. Sequencing and characterisation of an extensive Atlantic salmon (*Salmo salar L.*) microRNA repertoire. *PLoS One***8**, e70136 (2013).

10sr. Hinske, L. C. G., Galante, P. A., Kuo, W. P. & Ohno-Machado, L. A potential role for intragenic miRNAs on their hosts' interactome. *BMC genomics***11**, 1 (2010).

11sr. Paczynska, P., Grzemski, A. & Szydlowski, M. Distribution of miRNA genes in the pig genome. *BMC genetics***16**, 1 (2015).

12sr. Tang, G.-Q. & Maxwell, E. S. Xenopus microRNA genes are predominantly located within introns and are differentially expressed in adult frog tissues via post-transcriptional regulation. *Genome research***18**, 104–112 (2008).

13sr. Xu, H., Wang, X., Du, Z. & Li, N. Identification of microRNAs from different tissues of chicken embryo and adult chicken. *FEBS letters***580**, 3610–3616 (2006).

14sr. Romao, J. M., Jin, W., He, M., McAllister, T. et al. MicroRNAs in bovine adipogenesis: genomic context, expression and function. *BMC genomics***15**, 1 (2014).

15sr. Liu, G. et al. Computational identification and microarray-based validation of microRNAs in *Oryctolagus cuniculus*. *Molecular biology reports***37**, 3575–3581 (2010).

16sr. Xu, F. et al. Identification of conserved and novel microRNAs in the pacific oyster *Crassostrea gigas* by deep sequencing. *PloS One***9**, e104371 (2014).

17sr. Isik, M., Korswagen, H. C. & Berezikov, E. Expression patterns of intronic microRNAs in *Caenorhabditis elegans*. *Silence***1**, 1 (2010).

18sr. Upton, G. J. Fisher's exact test. *Journal of the Royal Statistical Society*.**Series A** (Statistics in Society) 395–402 (1992).

19sr. Hubisz, M. J., Pollard, K. S. & Siepel, A. PHAST and RPHAST: phylogenetic analysis with space/time models. *Briefings in bioinformatics***12**, 41–51 (2010).

20sr. Lorenz, R. et al. ViennaRNA Package 2.0. *Algorithms for Molecular Biology***6**, 26 (2011).

21sr. Grimson, A. et al. MicroRNA targeting specificity in mammals: determinants beyond seed pairing. *Molecular cell***27**, 91–105 (2007).

22sr. Lewis, B. P., Burge, C. B. & Bartel, D. P. Conserved seed pairing, often flanked by adenosines, indicates that thousands of human genes are microRNA targets. *Cell***120**, 15–20 (2005).
